# Copula-Based Approach to Synthetic Population Generation

**DOI:** 10.1371/journal.pone.0159496

**Published:** 2016-08-04

**Authors:** Byungduk Jeong, Wonjoon Lee, Deok-Soo Kim, Hayong Shin

**Affiliations:** 1 Department of Industrial & Systems Engineering, KAIST (Korea Advanced Institute of Science and Technology), Daejeon, South Korea; 2 Department of Mechanical Engineering, Hanyang University, Seoul, South Korea; Université Toulouse 1 Capitole, FRANCE

## Abstract

Generating synthetic baseline populations is a fundamental step of agent-based modeling and simulation, which is growing fast in a wide range of socio-economic areas including transportation planning research. Traditionally, in many commercial and non-commercial microsimulation systems, the iterative proportional fitting (IPF) procedure has been used for creating the joint distribution of individuals when combining a reference joint distribution with target marginal distributions. Although IPF is simple, computationally efficient, and rigorously founded, it is unclear whether IPF well preserves the dependence structure of the reference joint table sufficiently when fitting it to target margins. In this paper, a novel method is proposed based on the copula concept in order to provide an alternative approach to the problem that IPF resolves. The dependency characteristic measures were computed and the results from the proposed method and IPF were compared. In most test cases, the proposed method outperformed IPF in preserving the dependence structure of the reference joint distribution.

## Introduction

Large-scale micro-simulations using agent-based models have gained wide popularity in recent years in various fields of socio-economic studies [1] including transportation planning [2] and land use [3]. Generating synthetic baseline populations is a key step in agent-based modeling and simulation. An agent in a microsimulation is described by a set of attributes such as age, income, residence type/region, and so on. These attributes are usually dependent on each other. Hence, the synthetic population generation can be considered as creating a set of agents with the attributes drawn from a joint distribution. However, a difficult element of the synthetic population generation is obtaining a relevant data set. As described in [4], there are two typical types of data sets available: disaggregated census data in form of PUMS (Public Use Micro Samples) and aggregated data in form of summary tables in census reports. PUMS contains individual samples of small size (typically less than 5% of population), which can be used to infer the joint distribution of the attributes. On the other hand, aggregated data in summary tables exhibits marginal distributions of attributes specific to each analysis zone of interest. As samples in PUMS data are chosen from a rather larger area (like state or nationwide) than target zones, the reference joint distribution from PUMS is often inconsistent with marginal distributions from the aggregate data of each zone. Beckman et al. [5] proposed to combine the disaggregated data with the aggregated data using IPF (Iterative Proportional Fitting) procedure. The primary concept of IPF is to maintain the dependence structure from the disaggregated data and alter the joint distribution to fit the marginal distribution of the attributes from the aggregated data. (We will use the term ‘*marginal distribution*’ and ‘*margin’* interchangeably.) Since the inception by Beckman et al, there has been much research following this path: see [4,6]. IPF, which is briefly reviewed in a later section, is a very efficient and powerful technique for constructing a joint distribution table from a reference joint distribution and target margins. (In population synthesis area, the term ‘contingency table’ is often used to refer a table with the frequency of population in each cell. Since a contingency table can be easily converted to a distribution table (a probability mass function of discrete random variables), we will use the concept of distribution table instead.) Although the IPF procedure is very popularly in a variety of applications including synthetic population generation, it has some limitations as well. In this paper, we propose a novel approach based on copula theory for the same problem of constructing a joint distribution in place of IPF. It should be noted that the proposed approach can deal with only ordinal variables, not categorical ones.

Recently, some research papers have used copula theory for microsimulation of traffic behavior (e.g., see [7,8], in which the copula was used in different contexts. Kao et al. [9] also proposed a copula based approach to synthesizing households in order to preserve the dependence structure. However, they combine target margins using Gaussian copula, whose covariance matrix is determined from the reference joint distribution (possibly represented by samples). A limitation of this approach is that some dependency information is lost because of the intermediate Gaussian copula. A similar approach can be found in [10], which utilizes Copula for representing temporal dependence structure among time series of stream flow in a geographic region. These literature uses some well-known copula functions such as Gaussian or Gumbel copula, then parameters of the copula function are chosen to fit the data. On the other hand, in this paper, we propose to directly use the empirical copula as explained in “Copula based approach to joint fitting problem” section.

## Problem Description

Though, in synthetic population generation, there are many research issues such as household–individual hierarchy and aggregation data inconsistency, the problem focused in this paper is the construction of a joint distribution from a given reference joint distribution and target margins. Although the approach is applicable to multi-dimensional distributions without significant modification, the description in this paper is confined to two dimensional setting, for simplicity. For the most part of this paper, we will assume that target margins are discrete distributions, then the procedure will be extended to continuous variables in “Distribution view of CBJF and extensions” section.

**Fig 1:**
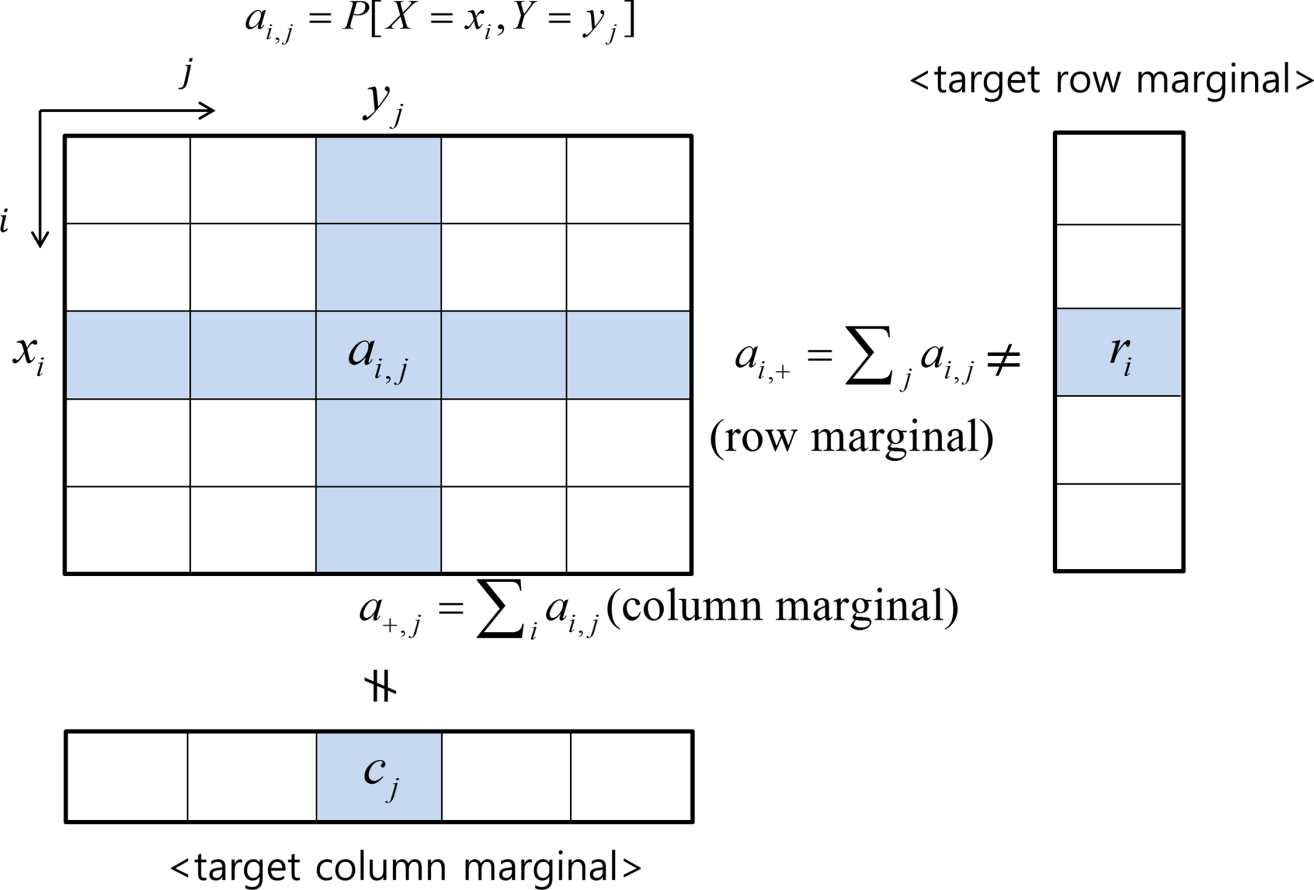
Joint fitting problem.

In order to formally describe the problem, the following notations are introduced. Let (X,Y) be a pair of discrete random variables that represent the attributes of the reference population. X and Y can have values from {*x*_1_,*x*_2_,…,*x*_*m*_} and {*y*_1_,*y*_2_,…,*y*_*n*_} respectively. We assume that X and Y are ordinal or interval variables, possible values of which have natural ordering. (For the variable types, readers are referred to [11].) The possibility of relaxing this assumption to handle categorical variables will be discussed in the conclusion section as a further research topic. Let $\left(\tilde{X},\tilde{Y}\right)$ be a pair of random variables representing the attributes of the target population. For simplicity, we assume that $\tilde{X}$ and $\tilde{Y}$ have the same values as X and Y, respectively. Note that this assumption can be easily removed in the proposed approach by introducing a mapping between them. We will use the following notations, illustrated in Fig 1.

- **a** = [*a*_*i*,*j*_]: an *m*×*n* matrix denoting the reference joint distribution of (X,Y),
  i.e. *a*_*i*,*j*_ = P[X = *x*_*i*_, Y = *y*_*j*_]
- **r** = [*r*_*i*_]: target marginal distribution of $\tilde{X}$, i.e. $r_{i}=P[\tilde{X}=x_{i}]$
- **c** = [*c*_*j*_]: target marginal distribution of $\tilde{Y}$, i.e. $c_{j}=P[\tilde{Y}=y_{j}]$
- **b** = [*b*_*i*,*j*_]: an *m*×*n* matrix denoting the target joint distribution of $\left(\tilde{X},\tilde{Y}\right)$,
  i.e. $b_{i,j}=P[\tilde{X}=x_{i},\tilde{Y}=y_{j}]$

The reference joint distribution **a** can be either given directly or obtained from the detailed disaggregated census data available in form of PUMS (by counting the samples in each cell), while the target margins **r** and **c** are obtained from the aggregated data. From the input data {**a**,**r**,**c**}, the goal is to find the target joint distribution **b** inheriting the dependence structure from **a** while fitting it to the margins **r** and **c**. We call this problem a *joint fitting problem*. By definition, *a*_*i*,*j*_, *r*_*i*_, *c*_*j*_, *b*_*i*,*j*_ are probabilities, meaning that they are nonnegative and sum to one. The following symbols are used to denote marginal summations:

- *a*_*i*,+_ = ∑_*j*_*a*_*i*,*j*_: row margin of **a**, i.e. *a*_*i*,+_ = P[X = *x*_*i*_]
- *a*_+,*j*_ = ∑_*i*_*a*_*i*,*j*_: column margin of **a**, i.e. *a*_+,*j*_ = P[Y = *y*_*j*_]
- *b*_*i*,+_ = ∑_*j*_*b*_*i*,*j*_: row margin of **b**
- *b*_+,*j*_ = ∑_*i*_*b*_*i*,*j*_: column margin of **b**

Using these symbols, the constraints of the problem are to satisfy *b*_*i*,+_ = *r*_*i*_ and *b*_+,*j*_ = *c*_*j*_. The goal of “*preserving the dependence structure*” in **a** may appear ambiguous. Quantitative measure of this goal is differently defined in each method described in the following sections.

## Brief Overview of IPF (Iterative Proportional Fitting)

IPF (Iterative Proportional Fitting) is a concise and efficient procedure to solve the joint fitting problem described in the previous section. IPF has many names, including RAS algorithm, matrix raking, matrix scaling, bi-proportional fitting, and so on. Since its introduction in by Deming & Stephan [12], the properties of IPF has been studied thoroughly and used widely in various fields including the synthetic population generation. Although there are some variations of IPF, its essence can be described using the following algorithm (Algorithm 1):

### Algorithm 1 (IPF)

*b*_*i*,*j*_ ← *a*_*i*,*j*_ (Initialization)

While (convergence criterion is not met)

        $b_{i,j}\;\leftarrow \;b_{i,j}\frac{r_{i}}{b_{i,+}}\;for\;\forall \;i,j\;(rowwise\;fitting)$

        ${\;b}_{i,j}\;\leftarrow \;b_{i,j}\frac{c_{j}}{b_{+,j}}\;for\;\forall \;i,j\;(columnwise\;fitting)$

A common choice of convergence criterion is to measure the maximum deviation ε from the given margins **r** and:

$$\text{Fitting error}={\text{max}}_{i}\;\left|b_{i,+}-r_{i}\right|+{\text{max}}_{j}\;\left|b_{+,j}-c_{j}\right|<\varepsilon$$ (1)

Ireland & Kullback [13] proved that if IPF procedure converges to a certain distribution table under the given constraints on the marginal, then the resulting table minimizes the relative entropy (called the ‘*discrimination information*’ in their paper), as defined below:

$$\text{RE}=\sum_{i,j}b_{i,j}\log\frac{b_{i,j}}{a_{i,j}}$$ (2)

Wong [14] investigated the reliability of IPF for use in geographical studies. Beckman et al. [5] proposes using IPF to combine disaggregated data with aggregated margins from different data sources. Following Beckman’s lead, much literature has been produced on adopting IPF for population generation, as summarized in [4]. Despite the popularity and rigorous mathematical analysis, IPF has some limitations, as follows:

- **Convergence problem:** Although this is rare in practical applications, IPF procedure may not converge. Pukelsheim & Simeone [15] showed conditions when IPF may fail to converge (i.e. when **a** can be permuted to a block diagonal structure in 2-dimensional case, as exemplified below). Though IPF converges very fast in general as explained in [16], it may take many iterations before reaching the desired level of fitting accuracy when there are cells with non-zero initial probability that are eventually wiped out to zero.
- **Zero margin / Zero cell problem:** If any of *a*_*i*,+_ (or *a*_+,*j*_) vanishes to zero while *r*_*i*_ (or *c*_*j*_, respectively) is not, the IPF procedure fails. This is called *zero margin problem*. Furthermore, the zero cells in **a** do not have a chance to obtain positive probability mass during IPF procedure. That is, if *a*_*i*,*j*_ = 0, then *b*_*i*,*j*_ = 0 always. A zero cell is not a problem in computational sense, however it poses a semantic problem because a zero cell can be a result of underrepresentation caused by the limited sample size. As a work-around, zero cells (and hence zero margins) are replaced with a very small number at the initialization step.
- **Dependence structure preserving:** Most importantly, it must be seriously considered whether IPF preserves well the dependence structure of the reference table **a**. Since there is no strict relationship between minimizing the discrimination information and preserving the dependence structure, minimum discrimination information does not guarantee the most similar dependence structure. Although IPF converges to the solution table **b** which minimizes the relative entropy subject to the marginal constraints, it does not necessarily mean that the dependence structure captured in **a** is best transferred to **b**. In this paper a new method is proposed to replace IPF and these methods are compared in terms of some dependence measures which can capture the strength of relationships between variables.

Tables 1 and 2 show a small example joint fitting problem and a solution obtained by IPF procedure. Inputs are the reference joint matrix **a** and the target margins **r**, **c** in Table 1. With the convergence tolerance ε = 10^−5^, the output of IPF procedure is shown in Table 2, obtained after more than 300 iterations. If *a*_5,3_ is changed to 0, the non-zero cells in **a** matrix are separated in two groups: upper-left group and lower-right group. In this case, IPF procedure does not come to convergence because probability mass in one group cannot be transferred to the other group due to the barricade of zero cells. If we replace zero cells with a very small number (10^−20^), IPF converges after 1000 iterations.

**Table 1:** Input matrix a and target margins r, c.

Ref	*y*_1_	*y*_2_	*y*_3_	*y*_4_	*y*_5_	[*a*_*+*,*i*_]	[*r*_*i*_]
*x*_1_	0.04	0	0	0	0	0.04	0.07
*x*_2_	0.08	0.04	0	0	0	0.12	0.13
*x*_3_	0	0.12	0.08	0	0	0.12	0.15
*x*_4_	0	0	0.16	0	0.04	0.20	0.25
*x*_5_	0	0	0.04	0.2	0	0.24	0.27
*x*_6_	0	0	0.04	0.04	0.04	0.12	0.07
*x*_7_	0	0	0.04	0.04	0	0.08	0.06
[*a*_*i*,*+*_]	0.12	0.16	0.36	0.28	0.08	1	1
[*c*_*j*_]	0.16	0.17	0.30	0.25	0.12	1	

**Table 2:** IPF output b_IPF_.

IPF	*y*_1_	*y*_2_	*y*_3_	*y*_4_	*y*_5_
*x*_1_	0.07	0	0	0	0
*x*_2_	0.09	0.04	0	0	0
*x*_3_	0	0.13	0.02	0	0
*x*_4_	0	0	0.167	0	0.083
*x*_5_	0	0	0.059	0.211	0
*x*_6_	0	0	0.019	0.014	0.037
*x*_7_	0	0	0.035	0.025	0

## Copula Based Approach to Joint Fitting Problem

*Copula*, which was first coined by Sklar in [17] from a Latin word *copulare* meaning “to connect or link”, is a popular tool for modeling dependence between random variables. This section begins by reviewing the foundation of the copula theory. Further details on the copula can be found in [18]. A copula C(*u*,*v*) is a joint cumulative distribution function whose margins are uniform (0,1) distributions, satisfying the following properties:

1. Uniform (0,1) margins:
  C(u,0)=0andC(0,v)=0
  C(u,1)=uandC(1,v)=v
2. Monotonously increasing:
  C(u,v)≤C(u+du,v)foranydu>0
  C(u,v)≤C(u,v+dv)foranydv>0
3. Rectangle inequality (non-negative probability for [*u*,*u* + *du*] × [*v*,*v* + *dv*]):
  C(u+du,v+dv)−C(u+du,v)−C(u,v+dv)+C(u,v)≥0foranydu,dv>0

A copula can also be obtained from a joint distribution. Let F_XY_(*x*,*y*) = P[X ≤ *x*, Y ≤ *y*] denote the joint cumulative distribution function of (X,Y). Furthermore, let F_X_(*x*) = P[X ≤ *x*] and F_Y_(*y*) = P[Y ≤ *y*] be the marginal cumulative distribution functions of X and Y respectively. The copula of F_XY_(*x*,*y*) is defined by a function C(*u*,*v*) that satisfies the following:

$${\text{F}}_{\text{XY}}\left(x,y\right)=\text{C}\left({\text{F}}_{\text{X}}\left(x\right),{\text{F}}_{\text{Y}}\left(y\right)\right)$$ (3)

Sklar’s theorem states that such a function C(*u*,*v*) exists, and if X and Y are continuous, C(*u*,*v*) is uniquely determined. That is, by letting *u* = F_X_(*x*) and *v* = F_Y_(*y*), the copula function associated with F_XY_(*x*,*y*) can be obtained as follows:

$$\text{C}\left(u,v\right)={\text{F}}_{\text{XY}}\left({\text{F}}_{\text{X}}^{-1}\left(u\right),{\text{F}}_{\text{Y}}^{-1}\left(v\right)\right)$$ (4)

However, if the variables are not continuous (like X and Y in this paper), then the copula C is not unique; in this case, the values of the copula are uniquely determined at points (x,y), and a copula C for which the properties (a)-(c) above holds can be obtained by interpolating the values at these points [19]. The proof for the general n-dimensional case is outlined in [20]. When we speak of the copula of variables X and Y, we will mean the copula whose existence is guaranteed using the bilinear interpolation, if one or both of the random variables are not continuous.

Using the bilinear interpolation (detail procedure is explained in the last part of this chapter), the copula function C(*u*,*v*) is obtained from the joint cumulative distribution function by removing the marginal information, meaning that C(*u*,*v*) extracts the dependence structure of the joint distribution without the information specific to margins. This forms the perfect basis of applying the copula to the joint fitting problem: the copula function derived from the reference joint distribution of (X,Y) can be combined with the new target margins of $\tilde{X}$ and $\tilde{Y}$ to form the target joint distribution of $\left(\tilde{X},\tilde{Y}\right)$. This is further elaborated with basic mathematical operations.

Let F_XY_(*x*,*y*) denote the joint cumulative distribution function of (X,Y), and let F_X_(*x*) and F_X_(*x*) denote the marginal distribution of X and Y, respectively. Let C(*u*,*v*) be the copula function obtained via bilinear interpolation. If we set U = F_X_(X) and V = F_Y_(Y), then C(*u*, *v*) is the cumulative distribution function of (U,V). Now, the goal is to find the joint distribution of $\left(\tilde{X},\tilde{Y}\right)$, whose marginal cumulative distributions are $F_{\tilde{X}}(x)$ and $F_{\tilde{Y}}(y)$. We would like to make the dependence structure of the joint distribution of $\left(\tilde{X},\tilde{Y}\right)$ as close to that of (X, Y) as possible. For this reason, the same copula C(*u*, *v*) is applied to the margins $F_{\tilde{X}}(x)$ and $F_{\tilde{Y}}(y)$ in order to achieve the goal. Let $\tilde{U}=F_{\tilde{X}}(\tilde{X})$ and $\tilde{V}=F_{\tilde{Y}}(\tilde{Y})$. Then, C(*u*, *v*) is used as the cumulative distribution function of $\left(\tilde{U},\tilde{V}\right)$.

$$\begin{array}{l} {\text{F}}_{\tilde{X}\tilde{Y}}\left(x_{i},y_{j}\right)=\text{P}\left[\tilde{X}\leq x_{i},\tilde{Y}\leq y_{j}\right]=\text{P}\left[{\text{F}}_{\tilde{X}}\left(\tilde{X}\right)\leq {\text{F}}_{\tilde{X}}\left(x_{i}\right),{\text{F}}_{\tilde{Y}}\left(\tilde{Y}\right)\leq {\text{F}}_{\tilde{Y}}\left(y_{j}\right)\right]\\ \;=\text{P}\left[\tilde{U}\leq {\text{F}}_{\tilde{X}}\left(x_{i}\right),\tilde{V}\leq {\text{F}}_{\tilde{Y}}\left(y_{j}\right)\right]=\text{C}\left({\text{F}}_{\tilde{X}}\left(x_{i}\right),{\text{F}}_{\tilde{Y}}\left(y_{j}\right)\right) \end{array}$$ (5)

The following symbols are introduced for denoting cumulative distributions:

- $A_{i,j}=\sum_{k=1}^{i}\sum_{l=1}^{j}a_{k,l}$: cumulative distribution of (X, Y), i.e. *A*_*i*,*j*_ = P[X ≤ *x*_*i*_, Y ≤ *y*_*j*_]
- $u_{i}=\sum_{k=1}^{i}a_{k,+}$: cumulative distribution of X, i.e. *u*_*i*_ = P[X ≤ *x*_*i*_] = F_X_(*x*_*i*_)
- $v_{j}=\sum_{l=1}^{j}a_{+,l}$: cumulative distribution of Y, i.e. *v*_*j*_ = P[Y ≤ *y*_*j*_] = F_Y_(*y*_*j*_)
- ${\tilde{u}}_{i}=\sum_{k=1}^{i}r_{k}$: cumulative distribution of $\tilde{X}$, i.e. ${\tilde{u}}_{i}=P[\tilde{X}\leq x_{i}]=F_{\tilde{X}}(x_{i})$
- ${\tilde{v}}_{j}=\sum_{l=1}^{j}c_{l}$: cumulative distribution of $\tilde{Y}$, i.e. ${\tilde{v}}_{j}=P[\tilde{Y}\leq y_{j}]=F_{\tilde{Y}}(y_{j})$
- $A_{i,0}=A_{0,j}=u_{0}=v_{0}={\tilde{u}}_{0}={\tilde{v}}_{0}=0$: for notational convenience

Because $F_{\tilde{X}}(x_{i})=P[\tilde{X}\leq x_{i}]={\tilde{u}}_{i}$ and $F_{\tilde{Y}}(y_{j})=P[\tilde{Y}\leq y_{j}]={\tilde{v}}_{j}$, we get $F_{\tilde{X}\tilde{Y}}(x_{i},y_{j})=C({\tilde{u}}_{i},\;{\tilde{v}}_{j})$. Therefore:

$$b_{i,j}=\text{P}\left[\tilde{X}=x_{i},\tilde{Y}=y_{j}\right]=\text{C}\left({\tilde{u}}_{i},{\tilde{v}}_{j}\right)-\text{C}\left({\tilde{u}}_{i-1},{\tilde{v}}_{j}\right)-\text{C}\left({\tilde{u}}_{i},{\tilde{v}}_{j-1}\right)+\text{C}\left({\tilde{u}}_{i-1},{\tilde{v}}_{j-1}\right)$$ (6)

**Fig 2:**
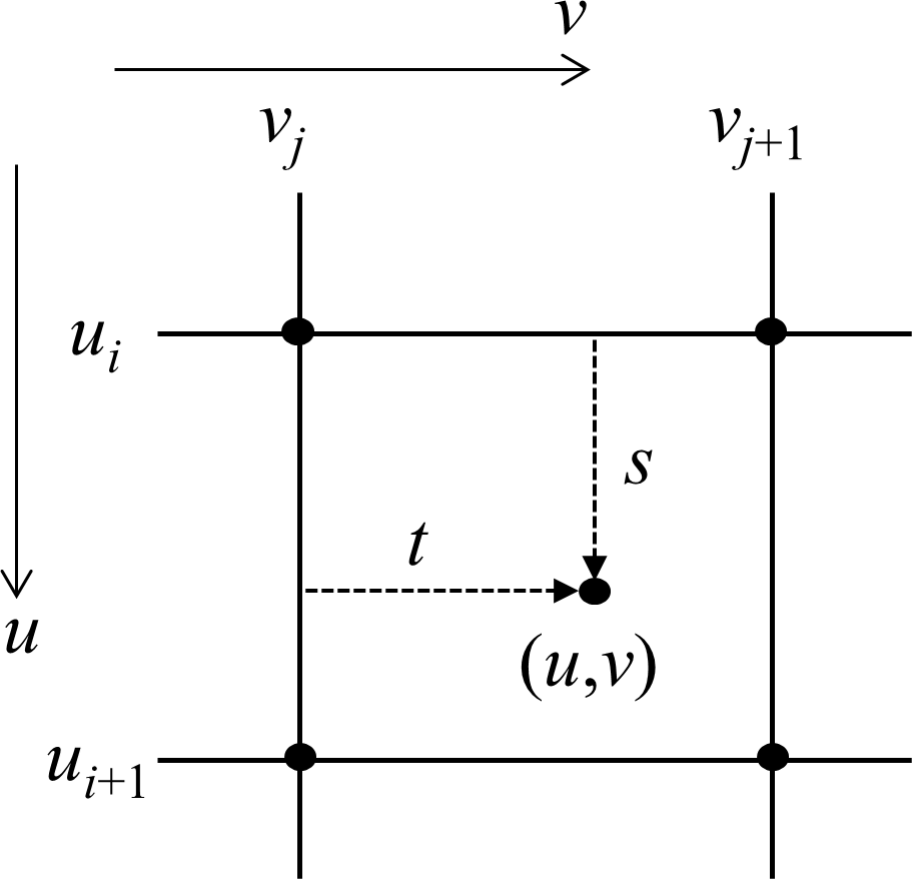
Bilinear interpolation.

Hence, the target joint distribution **b** can be computed, if we have the copula function C(*u*,*v*) obtained from **a**. Because *A*_*i*,*j*_ = P[X ≤ *x*_*i*_, Y ≤ *y*_*j*_] = P[U ≤ F_X_(*x*_*i*_), V ≤ F_Y_(*y*_*j*_)] = P[U ≤ *u*_*i*_, V ≤ *v*_*j*_] = C(*u*_*i*_,*v*_*j*_), the values of C(*u*,*v*) is uniquely defined only at each grid points (*u*_*i*_,*v*_*j*_). Since $\left(\tilde{u},\tilde{v}\right)$ does not necessarily coincide with (*u*,*v*), C(*u*,*v*) should be estimated from adjacent known grid points. For a given (*u*,*v*), the cell [*u*_*i*_,*u*_*i*+1_] × [*v*_*j*_,*v*_*j*+1_] containing (*u*,*v*) can be found. The local parameters *s* and *t* ∈ [0,1] are defined as shown in Eq (7) and Fig 2:

$$s=\frac{u-u_{i}}{u_{i+1}-u_{i}}\;\text{and}\;t=\frac{v-v_{j}}{v_{j+1}-v_{j}}$$ (7)

Then, C(*u*, *v*) is computed using a bilinear interpolation as below:

$$\text{C}\left(u,v\right)=\left(1-s\right)\left(1-t\right)A_{i,j}+\left(1-s\right)tA_{i,j+1}+s\left(1-t\right)A_{i+1,j}+stA_{i+1,j+1}$$ (8)

With C(*u*, *v*) as defined in Eq (8), the following lemmas can be easily proved. (Proofs can be found in S1 Text.)

**Lemma 1.** C(*u*, *v*) is at least *C*^0^ continuous at all locations in [0,1] × [0,1].

**Lemma 2.** C(*u*, *v*) satisfies the properties (a)~(c) of a copula function.

Hence, C(*u*, *v*) qualifies as a copula from the reference joint distribution **a**. The following algorithm (Algorithm 2) is presented based on the copula, which we call *copula based joint fitting* (CBJF).

### Algorithm 2 (CBJF)

Step 0 (Initialize) 

        $u_{0}=v_{0}={\tilde{u}}_{0}={\tilde{v}}_{0}=0;\;A_{i,0}=A_{0,j}=B_{i,0}=B_{0,j}=0$

Step 1 (Compute [*A*_*i*,*j*_]) 

        *A*_*i*,*j*_ = *a*_*i*,*j*_ + *A*_*i*−1,*j*_ + *A*_*i*,*j*−1_ − *A*_*i*−1,*j*−1_ for *i* = 1,…,*m* and *j* = 1,…,*n*

Step 2 (Compute $\left[u_{i}],\;[v_{j}],\;[{\tilde{u}}_{i}],\;and\;[{\tilde{v}}_{j}\right]$)

        *u*_*i*_ = *a*_*i*,+_ + *u*_*i*−1_ for *i* = 1,…,*m*

        *v*_*j*_ = *a*_+,*j*_ + *v*_*j*−1_ for *j* = 1,…,*n*

        ${\tilde{u}}_{k}=r_{k}+{\tilde{u}}_{k-1}\;for\;k=1,\ldots ,m$

        ${\tilde{v}}_{l}=c_{l}+{\tilde{v}}_{l-1}\;for\;l=1,\ldots ,n$

Step 3 (Compute $\left[B_{k,l}=C({\tilde{u}}_{k},\;{\tilde{v}}_{l})\right]$) 

        For each *k* = 1,…,*m* and *l* = 1,…,*n*

                Find the cell (*i*,*j*) such that $\left({\tilde{u}}_{k},\;{\tilde{v}}_{l})\in [u_{i},u_{i+1}]\times [v_{j},v_{j+1}\right]$

                $s=\frac{{\tilde{u}}_{k}-u_{i}}{u_{i+1}-u_{i}}\;and\;t=\frac{{\tilde{v}}_{l}-v_{j}}{v_{j+1}-v_{j}}$

                *B*_*k*,*l*_ = (1 − *s*)(1 − *t*)*A*_*i*,*j*_ + (1 − *s*)*tA*_*i*,*j*+1_ + *s*(1 − *t*)*A*_*i*+1,*j*_ + *stA*_*i*+1,*j*+1_

Step 4 (Compute [*b*_*k*,*l*_]) 

        For each *k* = 1,…,*m* and *l* = 1,…,*n*

                *b*_*k*.*l*_ = *B*_*k*,*l*_ − *B*_*k*−1,*l*_ − *B*_*k*,*l*−1_ + *B*_*k*−1,*l*−1_

Shown in Table 3 is the output of Algorithm 2 (CBJF) applied to the input condition in Table 1. It can be noted that some zero cells in **a** matrix get some probability mass by CBJF, unlike the result of IPF shown in Table 2. It can be interpreted as *diffusion of probability through the lens of copula upon the request of target margins*.

**Table 3:** CBJF output b_CBJF_.

CBJF	*y*_1_	*y*_2_	*y*_3_	*y*_4_	*y*_5_
*x*_1_	0.063	0.007	0	0	0
*x*_2_	0.073	0.043	0.013	0.001	0
*x*_3_	0.022	0.076	0.050	0.002	0
*x*_4_	0.002	0.028	0.142	0.033	0.045
*x*_5_	0	0.008	0.047	0.164	0.051
*x*_6_	0	0.004	0.022	0.024	0.020
*x*_7_	0	0.004	0.025	0.027	0.004

## Distribution View of CBJF and Extensions

The computational complexity of the above Algorithm 2 (CBJF) is O(*T*), where *T = mn* is the total number of cells in the joint distribution table. Though it is a very efficient algorithm with linear complexity proportional to the input/output size, the total number of cells *T* grows exponentially as the dimension of target margins increases, which can easily lead to prohibitively large matrices for storage and computation. However, in many practical high dimensional distributions, non-zero cells are very sparse and this is the clue to handle high dimensional cases. In order to exploit the sparsity of the joint distribution table, we need to re-organize Algorithm 2.

**Fig 3:**
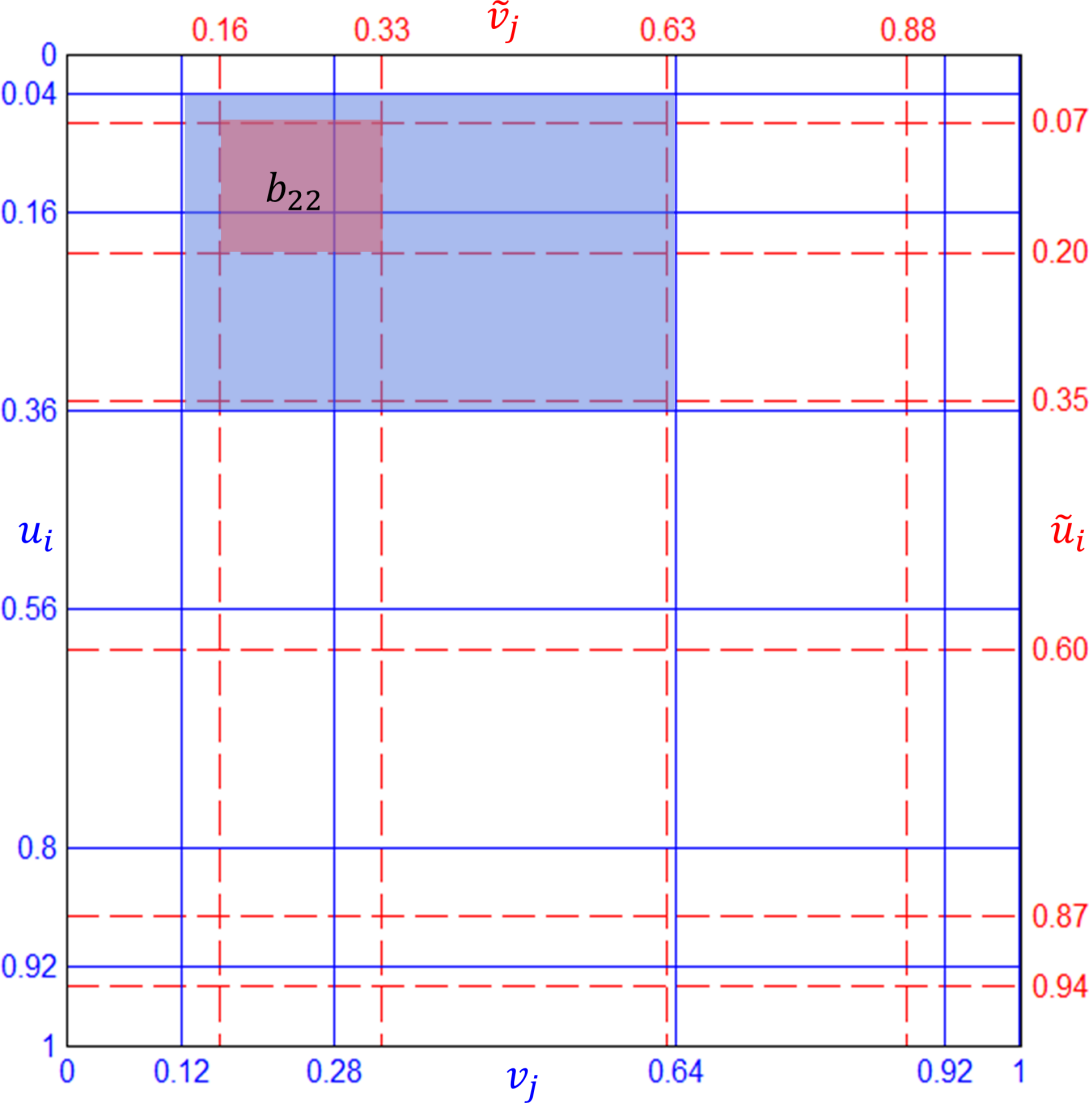
Collection view of CBJF (Partition lines are for the example in Table 1).

We will start with an interpretation of Algorithm 2 (CBJF). In step 2 of Algorithm 2, [*u*_*i*_] and [*v*_*j*_] define a partition *G* on [0,1]x[0,1] space, shown as solid (blue) lines in Fig 3. [*a*_*i*,*j*_] is the probability mass assigned to each cell *G*_*i*,*j*_ of the partition. The target margins **r** and **c** overlay a new partition $\tilde{G}$ defined by $\left[{\tilde{u}}_{i}\right]$ and $\left[{\tilde{v}}_{j}\right]$, shown as dashed (red) lines in Fig 3. Step 4 of Algorithm 2 can be interpreted as a “*collection view*”. In other words, the probability mass *b*_*i*,*j*_ for a cell ${\tilde{G}}_{i,j}$ in the new partition is computed by collecting the probability mass of cells in *G* overlapping with ${\tilde{G}}_{i,j}$. For example, the cell ${\tilde{G}}_{2,2}$ overlaps with *G*_2,2_, *G*_2,3_, *G*_3,2_, and *G*_3,3_. So, *b*_2,2_ is computed by collecting probability mass from *a*_2,2_, *a*_2,3_, *a*_3,2_, and *a*_3,3_ in proportion to the ratio of the overlapping area, which is achieved by the bilinear interpolation copula function in Eq (8). In this view, every cell in the partition $\tilde{G}$ should be visited and computed, since the algorithm does not know in advance whether the visit will result in a zero cell or not.

**Fig 4:**
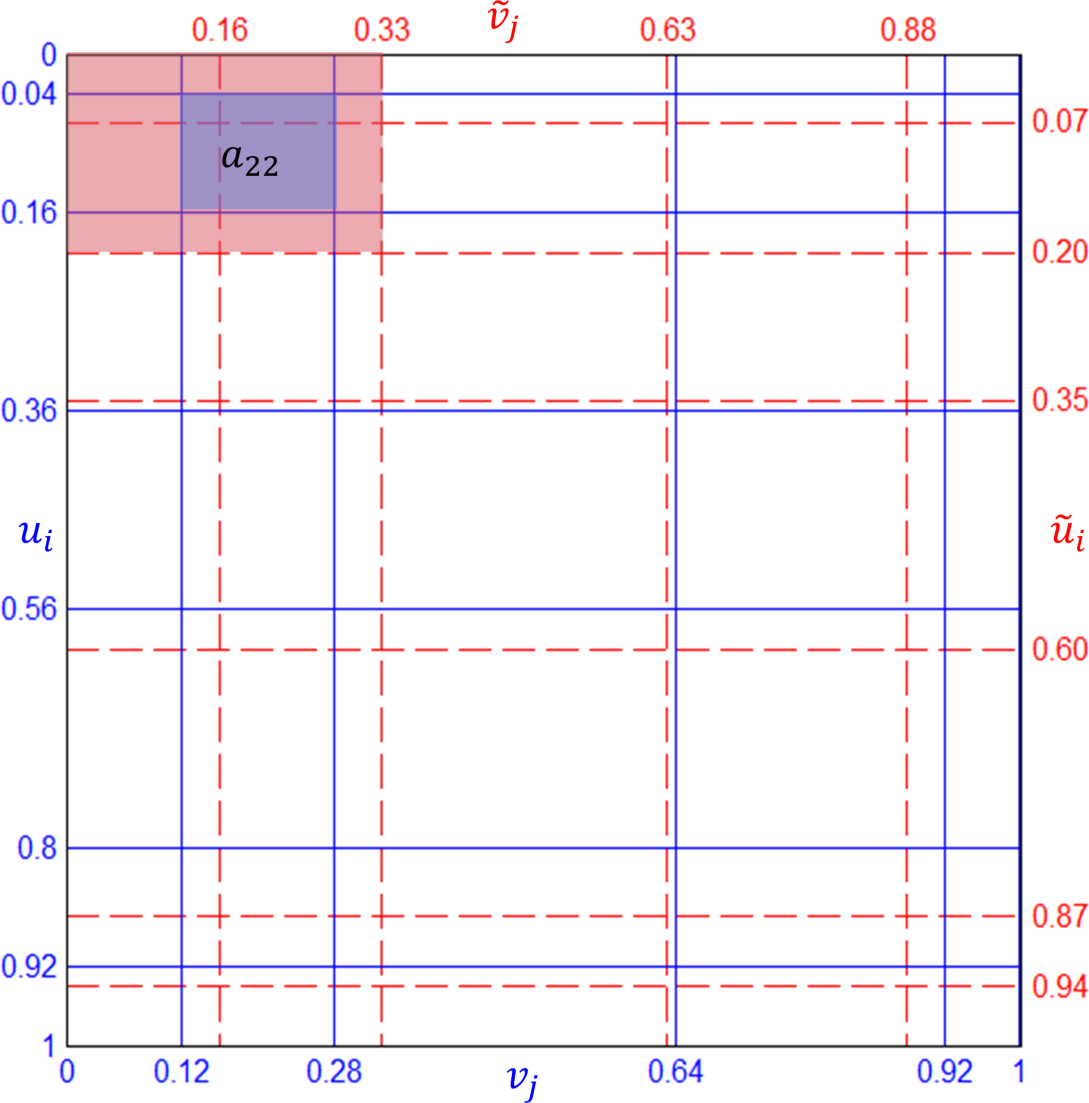
Distribution view of CBJF.

The same algorithm can be viewed from the other way around, which we call “*distribution view*”. Instead of collecting probability from the overlapping cells, we can visit each non-zero cell *G*_*i*,*j*_ and distribute the probability mass *a*_*i*,*j*_ to the overlapping cells in $\tilde{G}$, again in proportion to the ratio of the overlapping area. Fig 4 shows that *a*_2,2_ is distributed to *b*_1,1_, *b*_1,2_, *b*_2,1_, and *b*_2,2_, where *b*_*i*,*j*_ acts as an accumulator of probability mass incoming from each non-zero cell of *G*. Sparse matrix representation can be used to store **a** and **b**. This algorithm (Algorithm 3) is shown below.

### Algorithm 3 (CBJF-Distribution)

Step 0 (Initialize) 

            Define partition *G* by computing [*u*_*i*_], [*v*_*j*_]

            Define partition $\tilde{G}$ by computing $\left[{\tilde{u}}_{i}],\;[{\tilde{v}}_{j}\right]$

            [*b*_*i*,*j*_] is an empty zero martix

Step 1 (Distribute [*a*_*i*,*j*_]) 

            For each non-zero *a*_*i*,*j*_

                For each overlapping cell ${\tilde{G}}_{s,t}$

                        $p=area(G_{i,j}\cap {\tilde{G}}_{s,t})\;/\;area(G_{i,j})$

                        *b*_*s*,*t*_ = *b*_*s*,*t*_ + *p* * *a*_*i*,*j*_

Algorithm 3 (CBJF-Distribution) is simpler, faster, and storage-efficient, and hence, it can be applied to high dimensional case. On top of these benefits, it enables further extensions. Let us first look into an extension to resampling-based population generation, which is very common. In case that disaggregated micro-samples are given for the reference joint distribution, let ***p***^(*k*)^ = (*x*^(*k*)^,*y*^(*k*)^,***z***^(*k*)^) denote the k-th survey record in PUMS, which we will call a *PUMS entry* (*k*) hereafter in order to reduce confusion in the use of the term “sample”. *x*^(*k*)^ and *y*^(*k*)^ are attributes whose target margins are given, while ***z***^(*k*)^ is a vector of additional attributes. Let **P** = {***p***^(*k*)^; *k* = 1.*N*} denote the PUMS set. Once the target joint distribution **b** is obtained, it can be directly used for generating agents with attributes (*x*,*y*) drawn from **b**. However, we cannot set the additional attributes ***z***. This is the motivation of resampling based population generation, where synthetic population is generated by resampling **P**. In this case, a selection probability *w*^(*k*)^ is assigned to each PUMS entry ***p***^(*k*)^, so that resampling selects ***p***^(*k*)^ with probability *w*^(*k*)^. The goal of simple resampling is to compute *w*^(*k*)^ so as to meet the target margin requirement. Simple resampling can be efficiently combined with IPF procedure. However, in this case, the zero cell problem still persists, i.e. we cannot generate any population in a zero cell where no PUMS entry exists. This can be solved by viewing Algorithm 3 (CBJF-Distribution) at the granularity of PUMS entry. Algorithm 3 (CBJF-Distribution) allocates the probability mass of *a*_*i*,*j*_ to overlapping grids in $\tilde{G}$. On the same token, we can think a PUMS entry ***p***^(*k*)^ as a cell having the probability mass of 1/*N*. (When there are more than one PUMS entries in a cell, we can consider them as multiple layers overlaid on the same cell, each of which has the probability mass of 1/*N*.) For each ***p***^(*k*)^, we can find the overlapping cells ${\tilde{G}}_{s,t}$, and a copy of ***p***^(*k*)^ is added to the cell ${\tilde{G}}_{s,t}$ with the selection probability determined in proportion to the overlapping area ratio. Note that (*x*,*y*) attribute of ***p***^(*k*)^ is to be replaced by that of ${\tilde{G}}_{s,t}$. In this way, ***p***^(*k*)^ is duplicated into multiple copies, whose weights sum to 1/*N*, and each copy has different values of (*x*,*y*) attribute while retaining the same ***z***^(*k*)^ attribute. Details are given in Algorithm 4 (CBJF-Resampling). At the end of the algorithm, we get a set **Q** = {(***q***^(*k*)^,*w*^(*k*)^); *k* = 1.*M*}, which can be used as the source of resampling for synthetic population generation.

### Algorithm 4 (CBJF-Resampling)

Step 0 (Initialize) 

        Define partition *G* by computing [*u*_*i*_], [*v*_*j*_]

        Define partition $\tilde{G}$ by computing $\left[{\tilde{u}}_{i}],\;[{\tilde{v}}_{j}\right]$

        **Q** = an empty set

Step 1 (Distribute PUMS entry) 

        For each PUMS entry ***p***^(*k*)^ = (*x*^(*k*)^,*y*^(*k*)^,***z***^(*k*)^) ∈ **P**

                Find *G*_*i*,*j*_ (the cell where ***p***^(*k*)^ belongs)

                For each overlapping cell ${\tilde{G}}_{s,t}$

                        $w=area(G_{i,j}\cap {\tilde{G}}_{s,t})\;/\;area(G_{i,j})/N$

                        ***q*** = (*x*_*s*_,*y*_*t*_,***z***^(*k*)^)

                        Add (***q***,*w*) to **Q**

**Fig 5:**
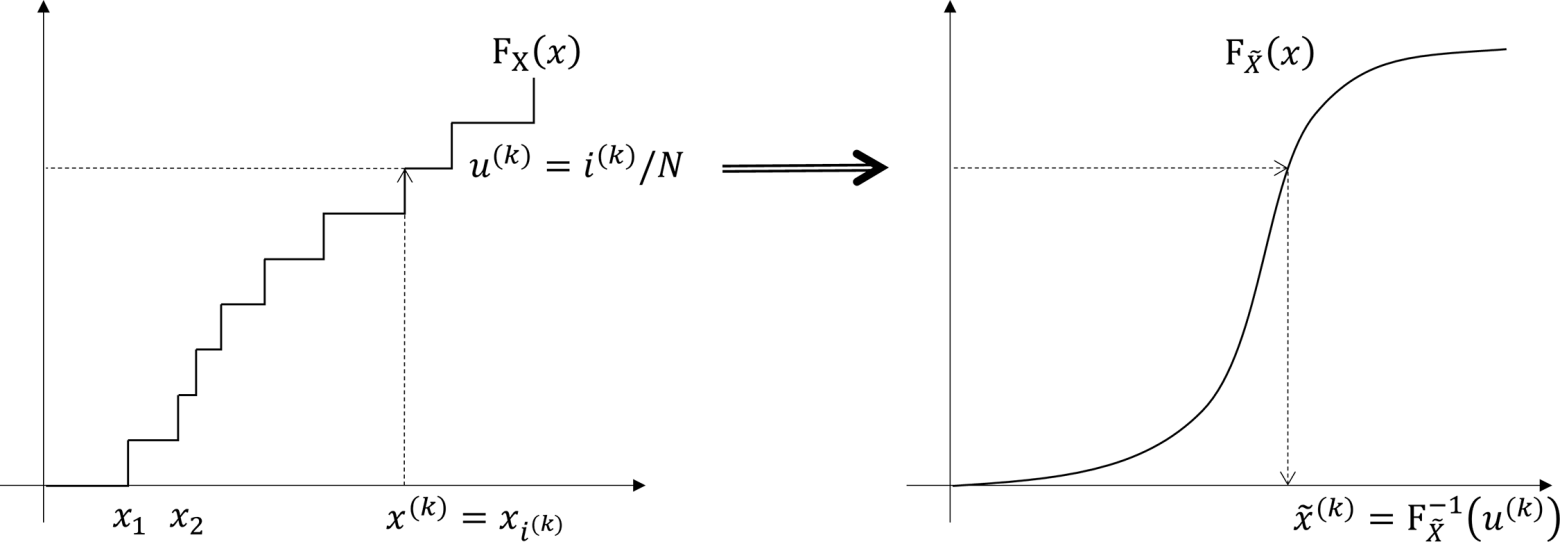
Mapping *x*^(*k*)^ to ${\tilde{x}}^{\left(k\right)}$.

The last extension is about handling continuous variables. For the continuous variable cases, it is natural to consider the reference joint is given as PUMS set **P** = {***p***^(*k*)^; *k* = 1.*N*} as above, while target margins are given in functional form $F_{\tilde{X}}(x)$ and $F_{\tilde{Y}}(y)$. We also assume that their inverses $F_{\tilde{X}}^{-1}(u)$ and $F_{\tilde{Y}}^{-1}(v)$ are available. In fact, continuous case is much simpler, at least conceptually, due to the fact that (*X*,*Y*) and $\left(F_{\tilde{X}}^{-1}(F_{X}(X)),F_{\tilde{Y}}^{-1}(F_{Y}(Y))\right)$ share the same copula [18]. For each PUMS entry ***p***^(*k*)^ = (*x*^(*k*)^,*y*^(*k*)^,***z***^(*k*)^), the corresponding point $q^{\left(k\right)}=({\tilde{x}}^{\left(k\right)},{\tilde{y}}^{\left(k\right)},z^{\left(k\right)})$ can be computed as shown in Fig 5: ${\tilde{x}}^{\left(k\right)}=F_{\tilde{X}}^{-1}(u^{\left(k\right)})$ and ${\tilde{y}}^{\left(k\right)}=F_{\tilde{Y}}^{-1}(v^{\left(k\right)})$, where *u*^(*k*)^ = F_X_(*x*^(*k*)^) and *v*^(*k*)^ = F_Y_(*y*^(*k*)^). Since the reference joint distribution is given as the PUMS set **P**, *u*^(*k*)^ = *i*^(*k*)^/*N* and *v*^(*k*)^ = *j*^(*k*)^/*N*, where *i*^(*k*)^ (or *j*^(*k*)^) is the number of PUMS entries ***p***^(*l*)^ with *x*^(*l*)^ ≤ *x*^(*k*)^ (or *y*^(*l*)^ ≤ *y*^(*k*)^). Computation of *u*^(*k*)^ (or *v*^(*k*)^) can be done more efficiently by sorting {*x*^(*l*)^} (or {*y*^(*l*)^}). This procedure is described in Algorithm 5 (CBJF-Continuous) below. The overall computational complexity of CBJF-Continuous is O(*N log N*), where *N* is the size of PUMS set. The new sample set **Q** = {***q***^(*k*)^; *k* = 1.*N*} is the source of resampling for synthetic population generation. In hybrid case where continuous and discrete variables are mixed, the above extensions can be easily combined and details are left to the readers.

### Algorithm 5 (CBJF-Continuous)

Step 0 (Initialize) 

        Sort {*x*^(*l*)^} and {*y*^(*l*)^}

        **Q** is an empty set

Step 1 (Transform PUMS set) 

        For each PUMS entry ***p***^(*k*)^ = (*x*^(*k*)^,*y*^(*k*)^,***z***^(*k*)^) ∈ **P**

                Obtain *i*^(*k*)^ and *j*^(*k*)^ from sorted sets

                *u*^(*k*)^ = *i*^(*k*)^/*N* and *v*^(*k*)^ = *j*^(*k*)^/*N*

                ${\tilde{x}}^{\left(k\right)}=F_{\tilde{X}}^{-1}(u^{\left(k\right)})$ and ${\tilde{y}}^{\left(k\right)}=F_{\tilde{Y}}^{-1}(v^{\left(k\right)})$

                Add $q^{\left(k\right)}=({\tilde{x}}^{\left(k\right)},{\tilde{y}}^{\left(k\right)},z^{\left(k\right)})$ to **Q**

## Numerical Experiments and Comparison

For comparison purpose, the joint fitting problem was also formulated as the following weighted least square formulation, which is an instance of a quadratic programming (QP Formulation) problem:

### QP Formulation

$Minimize\;\sum_{i,j}{w_{i,j}(b_{i,j}-a_{i,j})}^{2}$

Subject to

        *b*_*i*,+_ = r_i_ for i = 1,…,m,

        *b*_+,*j*_ = *c*_*j*_ for *j* = 1,…,*n*,

        *b*_*i*,*j*_ ≥ 0,

        where wi,j={1ai,jifai,j≠0Mifai,j=0},

        and M is a sufficiently large number.

Note that the objective function in the QP can be replaced with a weighted L_1_-norm:

$$Minimize\;\sum_{i,j}w_{i,j}|b_{i,j}-a_{i,j}|$$

Although the L_1_-norm is not a linear function, a standard technique can be applied to convert the optimization problem to a linear programming (LP) (see [21], for example), resulting in the following LP formulation, where the new variable $Z_{i,j}^{+}$ (or $Z_{i,j}^{-}$) represent the positive (or negative) part of *b*_*i*,*j*_ − *a*_*i*,*j*_. (The solution of this LP Formulation was also computed for the test cases listed in this section, however, LP results were discarded because the output quality was significantly inferior to other outputs. Though, we leave the formulation here for documentation purpose.)

### LP Formulation

$Minimize\;\sum_{i,j}w_{i,j}(Z_{i,j}^{+}+Z_{i,j}^{-})$

Subject to

        $b_{i,j}-a_{i,j}=Z_{i,j}^{+}-Z_{i,j}^{-}\;for\;\forall i,j$

        *b*_*i*,+_ = *r*_*i*_ for ∀*i*

        *b*_+,*j*_ = *c*_*j*_ for ∀*j*

        $b_{i,j}\geq 0,\;Z_{i,j}^{+}\geq 0,\;Z_{i,j}^{-}\geq 0\;for\;\forall i,j$

        where wi,j={1ai,jifai,j≠0Mifai,j=0},

        and M is a sufficiently large number.

Some quantitative measures will be introduced in order to compare the results in terms of preserving the dependence structure. The most popular measure of dependence is Pearson’s product moment correlation coefficient, which is defined as:

$$\rho \left(\text{X},\text{Y}\right):=\frac{\text{Cov}\left(\text{X},\text{Y}\right)}{\sqrt{\text{Var}\left(\text{X}\right)\text{Var}\left(\text{Y}\right)}}$$ (9)

Although it is simple and familiar, Pearson’s correlation coefficient measures only the linear dependence between X and Y. An alternative to Pearson’s correlation is a rank correlation, such as Spearman’s rho or Kendall’s tau. Spearman’s rho measures the Pearson’s correlation between the two uniform random variables F_X_(X) and F_Y_(Y):

$$\text{s}\left(\text{X},\text{Y}\right):=\rho \left({\text{F}}_{\text{X}}\left(\text{X}\right),{\text{F}}_{\text{Y}}\left(\text{Y}\right)\right)$$ (10)

For Kendall’s tau, consider the two independent samples (X_1_,Y_1_) and (X_2_,Y_2_) with the same joint distribution as (X,Y). (X_1_,Y_1_) and (X_2_,Y_2_) are *concordant* if (X_1_−X_2_)(Y_1_−Y_2_) > 0 or *discordant* if (X_1_−X_2_)(Y_1_−Y_2_) < 0. Then, Kendall’s tau is defined as follows:

$$\tau \left(\text{X},\text{Y}\right):=\text{E}\left[\text{sign}\left(\left({\text{X}}_{1}-{\text{X}}_{2}\right)\left({\text{Y}}_{1}-{\text{Y}}_{2}\right)\right)\right]=\text{P}\left[\left({\text{X}}_{1}-{\text{X}}_{2}\right)\left({\text{Y}}_{1}-{\text{Y}}_{2}\right)>0\right]-\text{P}\left[\left({\text{X}}_{1}-{\text{X}}_{2}\right)\left({\text{Y}}_{1}-{\text{Y}}_{2}\right)<0\right]$$ (11)

For the general types of dependence, the maximal information coefficient (MIC) [22] is also used. MIC is a measure of dependence which captures a wide range of (either functional or non-functional) associations between variables. In case that a functional relationship exists, MIC provides a score that roughly equals the coefficient of determination (R^2^) of the data relative to the regression function. (10,000 sample points were drawn from each joint distribution and used to calculate their MIC values.)

**Fig 6:**
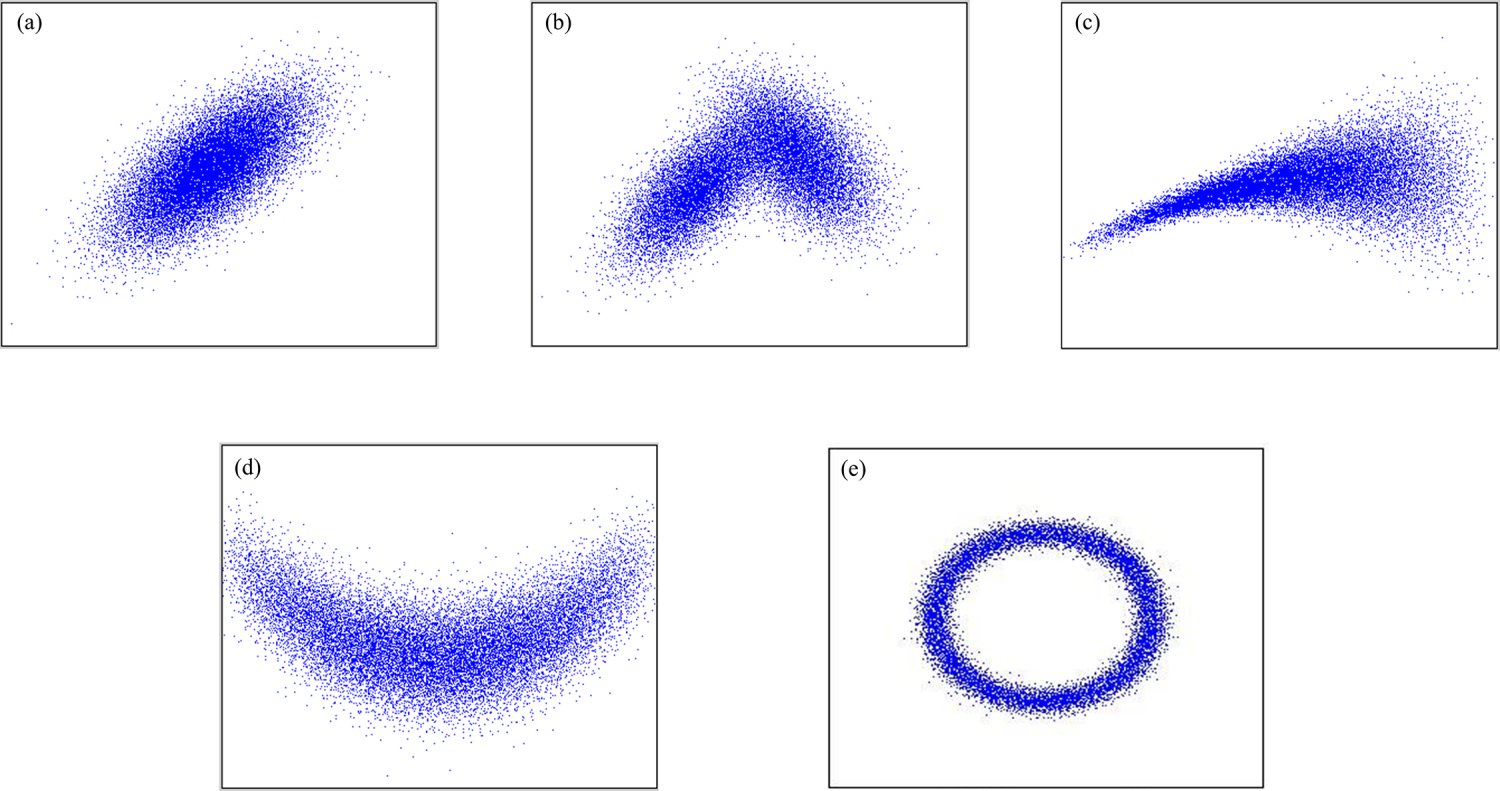
Reference joint types in the test set. (a) Bivariate normal, (b) Bimodal, (c) Lower tail dependence, (d) U-shape, (e) Circle.

In order to test the effectiveness of the proposed method, the following five different types of reference joint distribution types were chosen (shown in Fig 6):

- **Normal** (Fig 6(A)): bivariate normal with mean μ = (0.0, 0.0), standard deviation σ = 1 and correlation ρ = 0.7
- **Bimodal** (Fig 6(B)): mixture of two bivariate Gaussians
  - μ_1_ = (0.0, 0.0), σ_1_ = 1 and ρ_1_ = 0.7
  - μ_2_ = (3.0, 1.0), σ_2_ = 1 and ρ_2_ = -0.5
- **Tail dependent** (Fig 6(C)): a joint distribution showing strong tail dependence when X is low
- **U-shape** (Fig 6(D)): U-shaped distribution whose correlation coefficients are quite small
- **Circle** (Fig 6(E)): Circular joint distribution.

**Fig 7:**
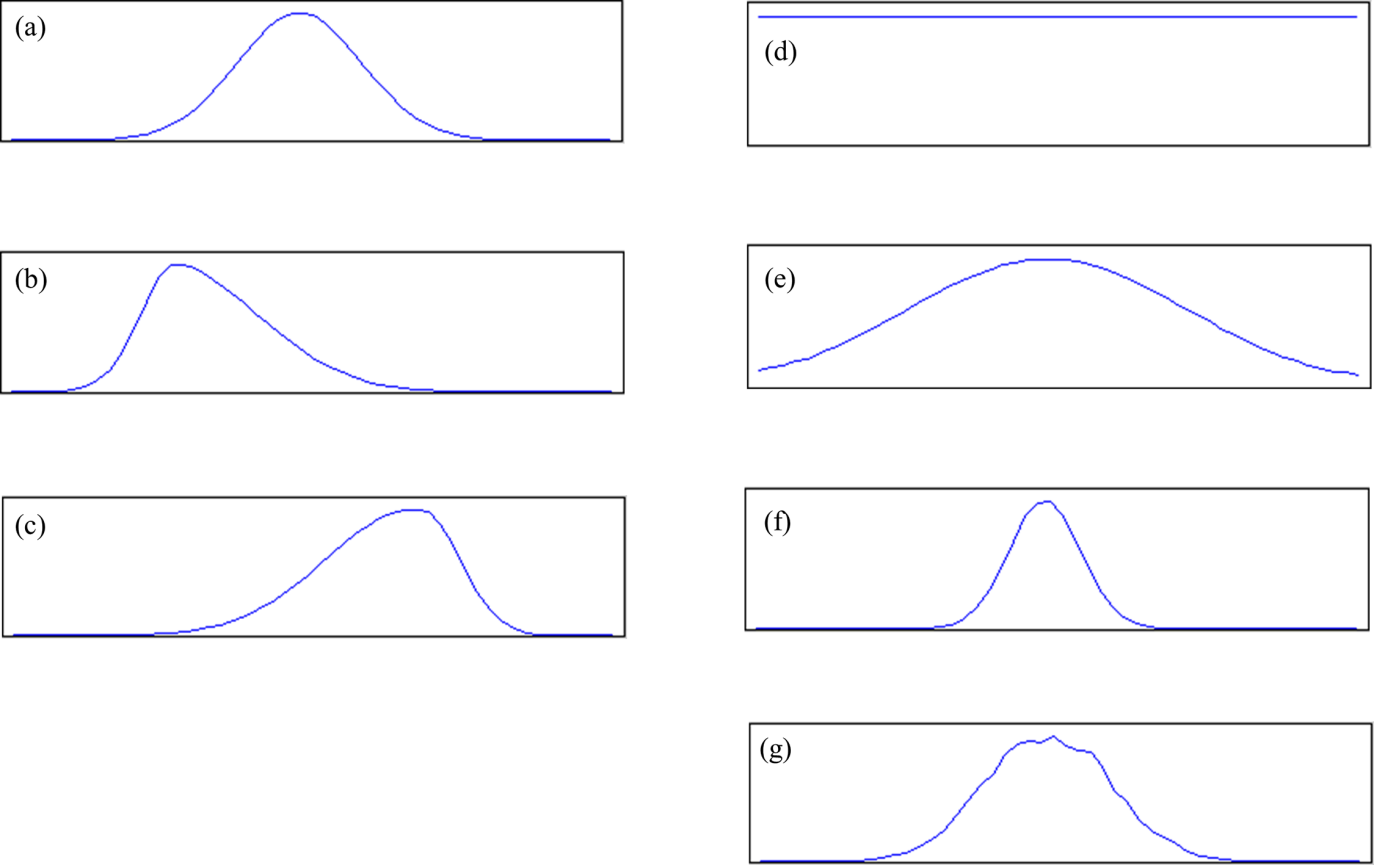
Target marginal modification operators. (a) Original marginal distribution, (b) Skew left, (c) Skew right, (d) Uniform, (e) Fat tail, (f) Thin tail, (g) Perturbation.

Then, in order to obtain target margins, the following eight types of marginal modification operators were applied to the margins of each reference joint distribution (see Fig 7):

- **Skew**: Left or right skew applied to the column margin and the row margin. There are 4 different combinations of skewed margins (LL, RR, LR, RL). For example, “Skew LR” indicates that the column margin [*c*_*j*_] is skewed left and the row margin [*r*_*i*_] is skewed right.
- **Uniform**: Uniform target margin
- **Fat tail**: To make the margin tails fatter
- **Thin tail**: To make the margin tails thinner
- **Perturb**: Add ±10% noise to each bin of the reference margins

Combining the five reference joint types with the eight marginal modification operators, a total of 40 combinations were used as the test set. The test set is not meant to be comprehensive, but rather some typical cases were selected in order to examine the effectiveness of the proposed methods in terms of dependency measures. Each margin was discretized into 100 bins, resulting in 100x100 cells in a joint table. For each combination of reference joint distribution and target margins, IPF procedure, QP method, and CBJF approach were applied in order to obtain the target joint distribution.

**Fig 8:**
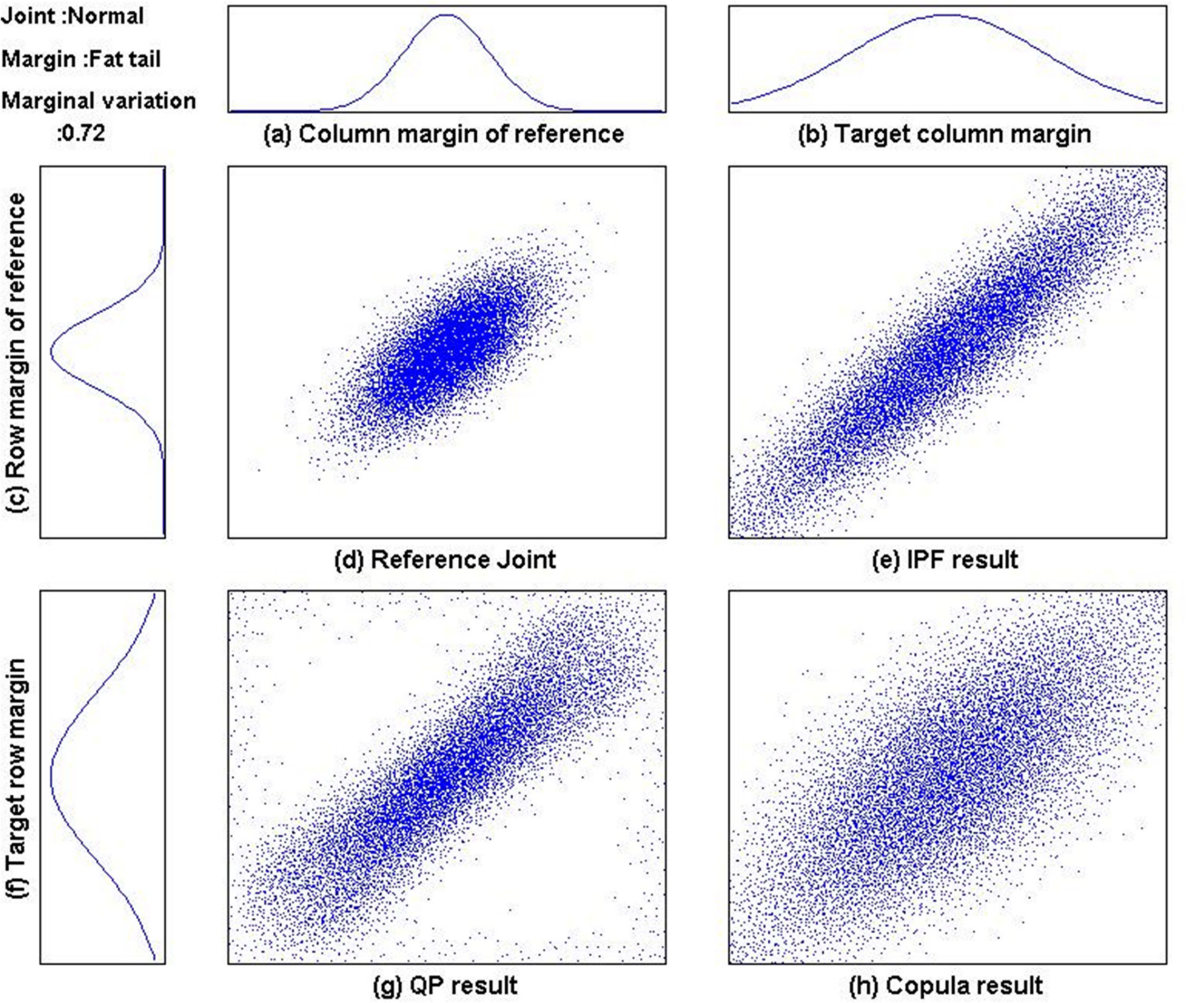
Output results for normal joint distribution and fat tail target margins.

Fig 8 shows the output distributions for one out of the 40 test cases: the normal joint + fat tail operator. Fig 8(D), 8(E), 8(G) and 8(H) are the scatter plots of the 10,000 sample points (for graphical presentation purpose) from the reference distribution, the IPF result, the QP result, and the CBJF result, respectively. While copula result seems to have similar dependent structure of reference joint, IPF and QP results look having more linearly correlated dependent structure. This is one of many cases where the output from CBJF approach outperforms the other methods. (The output plots for all 40 cases can be found in S1 Fig. Another numerical experiment of various changes on marginal distributions can be found in S2 Fig.)

IPF produces the smallest relative entropy (Eq (2)). However, relative entropy does not convey the same information as level of dependence structure preservation which can be measured from correlation coefficients. For the quantitative comparison of dependence structure preservation, the dependency measures stated in the previous section were computed: Pearson’s correlation, Spearman’s rho, Kendall’s tau, and the MIC. The results are shown in Tables 4–7 (rows marked as (a)), respectively. For each dependency measure, shown in Tables 4–7 (rows marked as (b)) are the deviation from the measure of the original reference joint table to that of the output joint table of each method. There, the smaller deviation implies the better preservation of dependence structure. The comparison results are summarized as follows:

- The perturbation operation (the second last columns of Tables 4–7) does not change the target margins significantly from those of the reference margins. For this small change, all three methods work well; showing almost no difference in the dependence measures.
- For the larger modifications, the copula based approach was superior to the other methods in almost all combinations. This indicates that the proposed method preserves the dependence structure of the reference joint distribution, while the other methods (IPF and QP) often fail to maintain the dependence structure when the target margins are significantly different from those of the reference.

**Table 4:** Pearson correlation coefficients ρ(X,Y).

Ref. Joint Type	Pearson ρ(Ref.)	Method	(a),(b)	Target marginal type								Sum
				Skew LL	Skew RR	Skew LR	Skew RL	Uni-form	Fat tail	Thin tail	Perturb	
Normal	0.685	IPF	(a)	0.714	0.712	0.683	0.660	0.950	0.910	0.370	0.684	
			(b)	0.030	0.027	0.001	0.025	0.266	0.226	0.314	0.001	0.889
		QP	(a)	0.581	0.633	0.784	0.844	0.738	0.797	0.290	0.684	
			(b)	0.104	0.052	0.099	0.159	0.054	0.113	0.395	0.001	0.976
		Copula	(a)	0.674	0.677	0.655	0.657	0.665	0.679	0.676	0.683	
			(b)	0.011	0.007	0.030	0.028	0.020	0.005	0.009	0.002	0.111
Bimodal	0.413	IPF	(a)	0.739	0.193	0.018	0.535	0.288	0.319	0.365	0.418	
			(b)	0.326	0.220	0.395	0.122	0.125	0.094	0.047	0.005	1.334
		QP	(a)	0.652	0.131	0.147	0.744	0.276	0.332	0.379	0.418	
			(b)	0.239	0.282	0.265	0.331	0.137	0.081	0.034	0.005	1.376
		Copula	(a)	0.308	0.437	0.379	0.361	0.419	0.407	0.418	0.407	
			(b)	0.105	0.024	0.034	0.052	0.006	0.006	0.006	0.006	0.238
Tail depen-dent	0.449	IPF	(a)	-0.450	0.489	-0.615	-0.077	0.088	0.317	0.207	0.447	
			(b)	0.899	0.041	1.063	0.526	0.361	0.132	0.241	0.002	3.264
		QP	(a)	-0.340	0.599	-0.631	0.214	0.135	0.272	0.164	0.447	
			(b)	0.789	0.150	1.079	0.234	0.314	0.177	0.284	0.002	3.029
		Copula	(a)	0.429	0.433	0.471	0.355	0.454	0.452	0.447	0.446	
			(b)	0.020	0.016	0.022	0.094	0.005	0.003	0.002	0.003	0.164
U-shape	-0.001	IPF	(a)	-0.159	0.107	0.091	-0.114	0.025	0.013	-0.002	0.000	
			(b)	0.155	0.111	0.095	0.110	0.029	0.017	0.002	0.003	0.522
		QP	(a)	-0.282	0.095	0.192	-0.103	0.021	0.012	-0.001	0.000	
			(b)	0.279	0.098	0.196	0.100	0.024	0.015	0.003	0.003	0.718
		Copula	(a)	0.047	-0.038	-0.042	0.041	-0.003	-0.003	-0.004	-0.001	
			(b)	0.051	0.035	0.039	0.045	0.000	0.000	0.000	0.002	0.172
Saddle	0.681	IPF	(a)	0.680	0.682	0.681	0.682	0.680	0.680	0.681	0.678	
			(b)	0.000	0.002	0.000	0.002	0.000	0.000	0.001	0.003	0.008
		QP	(a)	0.680	0.683	0.681	0.682	0.680	0.680	0.681	0.678	
			(b)	0.000	0.002	0.000	0.002	0.000	0.000	0.001	0.002	0.008
		Copula	(a)	0.680	0.680	0.680	0.680	0.680	0.680	0.680	0.681	
			(b)	0.000	0.001	0.000	0.001	0.000	0.000	0.000	0.000	0.002

(a) = Observed Pearson correlation ρ(Observed)

(b) = Absolute deviation |ρ(Reference)—ρ(Observed) |

**Table 5:** Spearman’s rank correlation coefficients s(X,Y).

Ref. Joint Type	Spear-man s(Ref.)	Method	(a),(b)	Target marginal type								Sum
				Skew LL	Skew RR	Skew LR	Skew RL	Uni-form	Fat tail	Thin tail	Perturb	
Normal	0.667	IPF	(a)	0.665	0.684	0.699	0.669	0.950	0.911	0.355	0.668	
			(b)	0.002	0.016	0.032	0.001	0.283	0.244	0.313	0.001	0.892
		QP	(a)	0.485	0.578	0.842	0.902	0.738	0.839	0.312	0.668	
			(b)	0.183	0.089	0.175	0.234	0.071	0.172	0.355	0.001	1.280
		Copula	(a)	0.663	0.663	0.663	0.663	0.665	0.665	0.658	0.666	
			(b)	0.004	0.004	0.004	0.004	0.002	0.003	0.009	0.002	0.032
Bimodal	0.430	IPF	(a)	0.770	0.154	0.023	0.579	0.288	0.344	0.360	0.440	
			(b)	0.340	0.276	0.407	0.148	0.142	0.086	0.070	0.010	1.480
		QP	(a)	0.693	0.115	0.210	0.822	0.276	0.358	0.371	0.440	
			(b)	0.262	0.315	0.220	0.392	0.154	0.072	0.059	0.010	1.485
		Copula	(a)	0.433	0.427	0.427	0.433	0.419	0.426	0.426	0.429	
			(b)	0.003	0.003	0.003	0.003	0.012	0.004	0.004	0.001	0.033
Tail depen-dent	0.452	IPF	(a)	-0.599	0.568	-0.774	-0.210	0.088	0.387	0.173	0.451	
			(b)	1.050	0.116	1.226	0.661	0.364	0.065	0.279	0.000	3.761
		QP	(a)	-0.435	0.705	-0.815	0.202	0.135	0.344	0.150	0.451	
			(b)	0.886	0.254	1.266	0.250	0.317	0.107	0.302	0.000	3.382
		Copula	(a)	0.448	0.445	0.448	0.445	0.454	0.453	0.435	0.450	
			(b)	0.004	0.006	0.003	0.007	0.002	0.001	0.016	0.001	0.040
U-shape	-0.010	IPF	(a)	-0.210	0.146	0.123	-0.178	0.025	0.003	-0.007	-0.009	
			(b)	0.200	0.156	0.134	0.167	0.036	0.013	0.003	0.001	0.710
		QP	(a)	-0.327	0.154	0.219	-0.195	0.021	0.003	-0.006	-0.009	
			(b)	0.317	0.164	0.230	0.185	0.031	0.013	0.004	0.001	0.945
		Copula	(a)	-0.009	-0.010	-0.010	-0.009	-0.003	-0.006	-0.015	-0.010	
			(b)	0.001	0.000	0.000	0.001	0.007	0.004	0.005	0.000	0.019
Saddle	0.681	IPF	(a)	0.680	0.683	0.681	0.682	0.680	0.680	0.681	0.678	
			(b)	0.000	0.002	0.000	0.002	0.000	0.000	0.001	0.003	0.009
		QP	(a)	0.680	0.683	0.681	0.682	0.680	0.680	0.681	0.678	
			(b)	0.000	0.002	0.000	0.002	0.000	0.000	0.001	0.003	0.009
		Copula	(a)	0.680	0.680	0.680	0.680	0.680	0.680	0.680	0.680	
			(b)	0.000	0.000	0.000	0.000	0.000	0.000	0.000	0.000	0.002

(a) = Observed Spearman's rank correlation s(Observed)

(b) = Absolute deviation | s(Reference)–s(Observed) |

**Table 6:** Kendall’s rank correlation coefficients τ(X,Y).

Ref. Joint Type	Kendall τ(Ref.)	Method	(a),(b)	Target marginal type								Sum
				Skew LL	Skew RR	Skew LR	Skew RL	Uni-form	Fat tail	Thin tail	Perturb	
Normal	0.478	IPF	(a)	0.481	0.488	0.500	0.473	0.791	0.736	0.239	0.479	
			(b)	0.003	0.010	0.021	0.005	0.313	0.258	0.239	0.001	0.851
		QP	(a)	0.341	0.405	0.723	0.766	0.577	0.661	0.205	0.479	
			(b)	0.137	0.073	0.244	0.288	0.099	0.183	0.273	0.001	1.300
		Copula	(a)	0.474	0.475	0.475	0.474	0.477	0.477	0.467	0.477	
			(b)	0.004	0.003	0.003	0.004	0.001	0.001	0.011	0.001	0.029
Bimodal	0.287	IPF	(a)	0.568	0.077	0.017	0.403	0.123	0.205	0.240	0.294	
			(b)	0.282	0.209	0.270	0.116	0.163	0.081	0.046	0.007	1.175
		QP	(a)	0.505	0.055	0.117	0.666	0.106	0.203	0.247	0.294	
			(b)	0.219	0.231	0.170	0.379	0.180	0.083	0.040	0.008	1.310
		Copula	(a)	0.284	0.286	0.285	0.285	0.286	0.286	0.283	0.286	
			(b)	0.002	0.001	0.001	0.002	0.000	0.000	0.003	0.001	0.011
Tail depen-dent	0.350	IPF	(a)	-0.371	0.458	-0.545	-0.055	0.187	0.446	0.123	0.350	
			(b)	0.721	0.108	0.895	0.405	0.163	0.096	0.227	0.000	2.615
		QP	(a)	-0.264	0.587	-0.603	0.198	0.209	0.426	0.115	0.350	
			(b)	0.615	0.237	0.953	0.152	0.141	0.076	0.235	0.000	2.409
		Copula	(a)	0.345	0.344	0.346	0.343	0.349	0.349	0.332	0.349	
			(b)	0.005	0.007	0.005	0.007	0.001	0.001	0.019	0.001	0.046
U-shape	-0.003	IPF	(a)	-0.145	0.106	0.091	-0.121	0.010	0.001	-0.001	-0.002	
			(b)	0.142	0.109	0.094	0.118	0.013	0.004	0.002	0.001	0.483
		QP	(a)	-0.232	0.121	0.162	-0.145	0.010	0.002	0.000	-0.002	
			(b)	0.229	0.124	0.165	0.143	0.012	0.005	0.002	0.001	0.681
		Copula	(a)	-0.003	-0.003	-0.003	-0.003	-0.003	-0.003	-0.003	-0.003	
			(b)	0.000	0.000	0.000	0.000	0.000	0.000	0.000	0.000	0.001
Saddle	0.481	IPF	(a)	0.491	0.493	0.491	0.492	0.490	0.491	0.492	0.488	
			(b)	0.000	0.002	0.000	0.001	0.000	0.000	0.001	0.002	0.007
		QP	(a)	0.491	0.493	0.491	0.492	0.490	0.491	0.492	0.488	
			(b)	0.000	0.002	0.000	0.002	0.000	0.000	0.001	0.002	0.008
		Copula	(a)	0.491	0.491	0.491	0.491	0.491	0.491	0.491	0.491	
			(b)	0.000	0.000	0.000	0.000	0.000	0.000	0.000	0.000	0.002

(a) = Observed Kendall's rank correlation τ(Observed)

(b) = Absolute deviation |τ(Reference)—τ(Observed) |

**Table 7:** MIC (Maximal Information Coefficient).

Ref. Joint Type	MIC (Ref.)	Method	(a),(b)	Target marginal type								Sum
				Skew LL	Skew RR	Skew LR	Skew RL	Uni-form	Fat tail	Thin tail	Perturb	
Normal	0.328	IPF	(a)	0.346	0.344	0.344	0.343	0.829	0.669	0.134	0.336	
			(b)	0.018	0.016	0.016	0.015	0.501	0.341	0.194	0.008	1.109
		QP	(a)	0.212	0.223	0.692	0.688	0.534	0.590	0.122	0.334	
			(b)	0.116	0.105	0.364	0.360	0.206	0.262	0.206	0.006	1.625
		Copula	(a)	0.329	0.329	0.329	0.326	0.326	0.330	0.327	0.330	
			(b)	0.001	0.001	0.001	0.002	0.002	0.002	0.001	0.002	0.012
Bimodal	0.325	IPF	(a)	0.395	0.225	0.283	0.369	0.603	0.515	0.189	0.327	
			(b)	0.070	0.100	0.042	0.044	0.278	0.190	0.136	0.002	0.862
		QP	(a)	0.694	0.507	0.527	0.686	0.633	0.569	0.193	0.328	
			(b)	0.369	0.182	0.202	0.361	0.308	0.244	0.132	0.003	1.801
		Copula	(a)	0.322	0.323	0.321	0.323	0.325	0.322	0.328	0.328	
			(b)	0.003	0.002	0.004	0.002	0.000	0.003	0.003	0.003	0.020
Tail depen-dent	0.198	IPF	(a)	0.345	0.351	0.362	0.396	0.563	0.608	0.075	0.192	
			(b)	0.147	0.153	0.164	0.198	0.365	0.410	0.123	0.006	1.566
		QP	(a)	0.275	0.631	0.632	0.361	0.457	0.756	0.086	0.194	
			(b)	0.077	0.433	0.434	0.163	0.259	0.558	0.112	0.004	2.040
		Copula	(a)	0.195	0.197	0.197	0.194	0.192	0.195	0.197	0.195	
			(b)	0.003	0.001	0.001	0.004	0.006	0.003	0.001	0.003	0.022
U-shape	0.259	IPF	(a)	0.276	0.293	0.279	0.286	0.601	0.516	0.105	0.253	
			(b)	0.017	0.034	0.020	0.027	0.342	0.257	0.154	0.006	0.857
		QP	(a)	0.519	0.632	0.519	0.634	0.484	0.483	0.103	0.252	
			(b)	0.260	0.373	0.260	0.375	0.225	0.224	0.156	0.007	1.880
		Copula	(a)	0.260	0.258	0.259	0.259	0.258	0.254	0.255	0.253	
			(b)	0.001	0.001	0.000	0.000	0.001	0.005	0.004	0.006	0.018
Saddle	0.457	IPF	(a)	0.532	0.521	0.540	0.533	0.521	0.468	0.436	0.455	
			(b)	0.075	0.064	0.083	0.076	0.064	0.011	0.021	0.002	0.396
		QP	(a)	0.577	0.555	0.566	0.569	0.219	0.509	0.493	0.455	
			(b)	0.120	0.098	0.109	0.112	0.238	0.052	0.036	0.002	0.767
		Copula	(a)	0.455	0.456	0.456	0.456	0.458	0.460	0.454	0.453	
			(b)	0.002	0.001	0.001	0.001	0.001	0.003	0.003	0.004	0.016

(a) = Observed MIC

(b) = Absolute deviation | MIC(Reference)—MIC(Observed)

Since not only the type of marginal modification operator but also the amount of marginal change affects the result, skew, fat tail and thin tail operators are applied to each of the reference joint types with various levels of marginal change. And for each test combination, the MIC is calculated to see how the marginal change affects the dependence structure. Marginal changes are controlled by marginal variation which we define as the summation of the total variation distance of row margins and that of column margins where total variation distance between distribution P and Q is:

$$\delta \left(\text{P},\text{Q}\right)=\frac{1}{2}{\left\|P-Q\right\|}_{1}=\frac{1}{2}\underset{x}{\sum }\left|P\left(x\right)-Q\left(x\right)\right|$$ (12)

**Fig 9:**
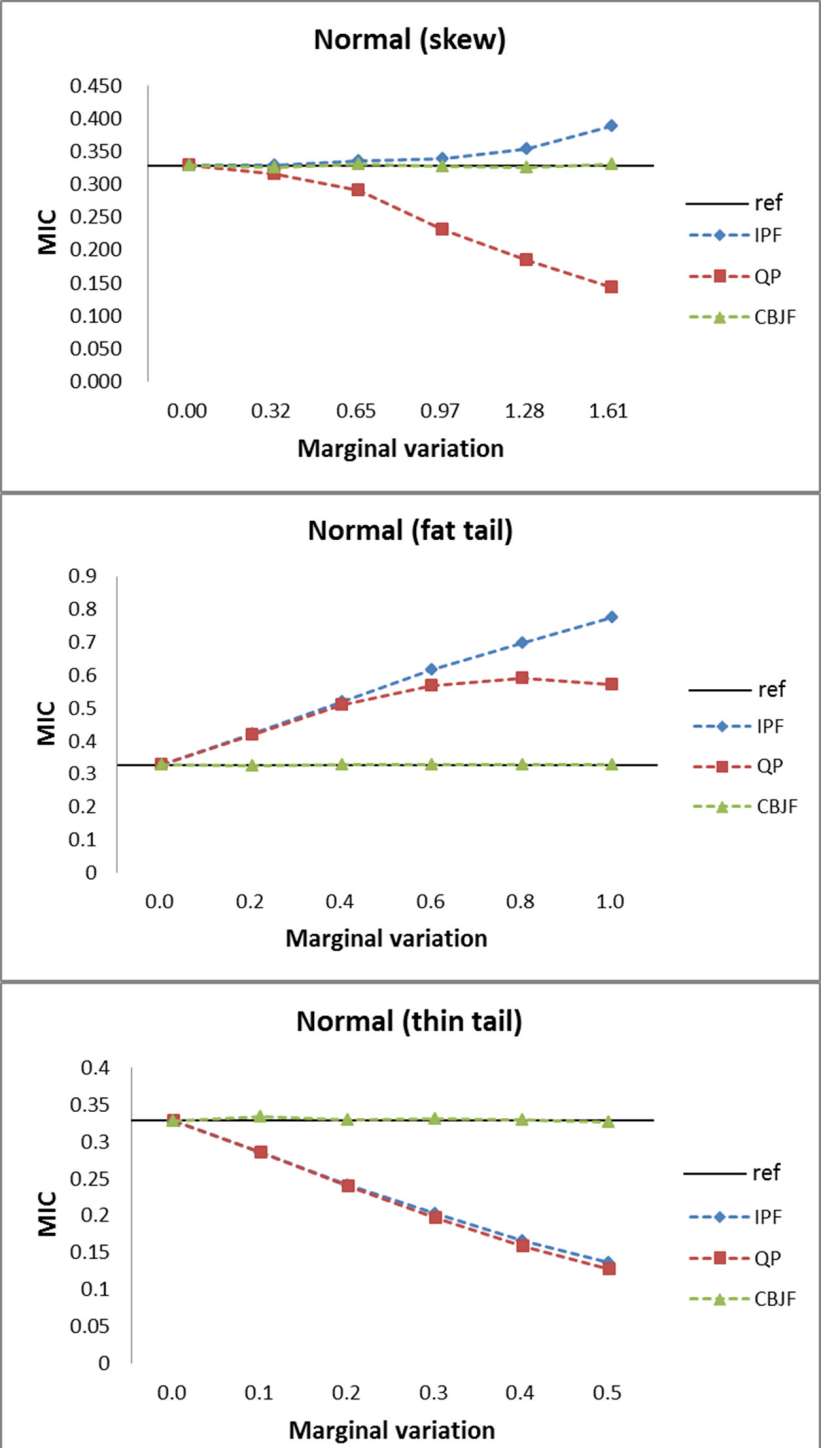
Results from the normal joint distribution with skew, fat tail and thin tail operators.

**Fig 10:**
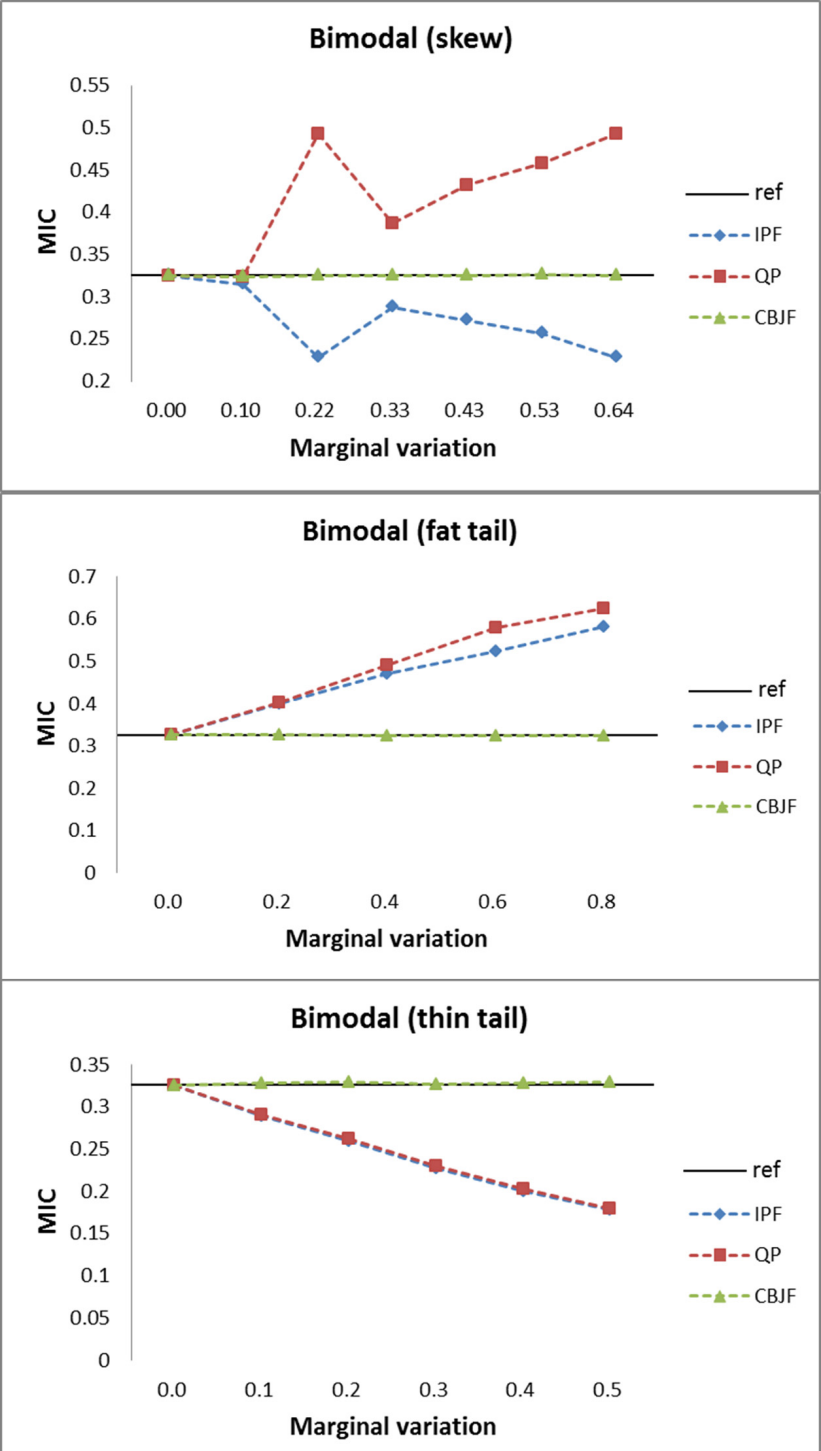
Results from the bimodal joint distribution with skew, fat tail and thin tail operators.

**Fig 11:**
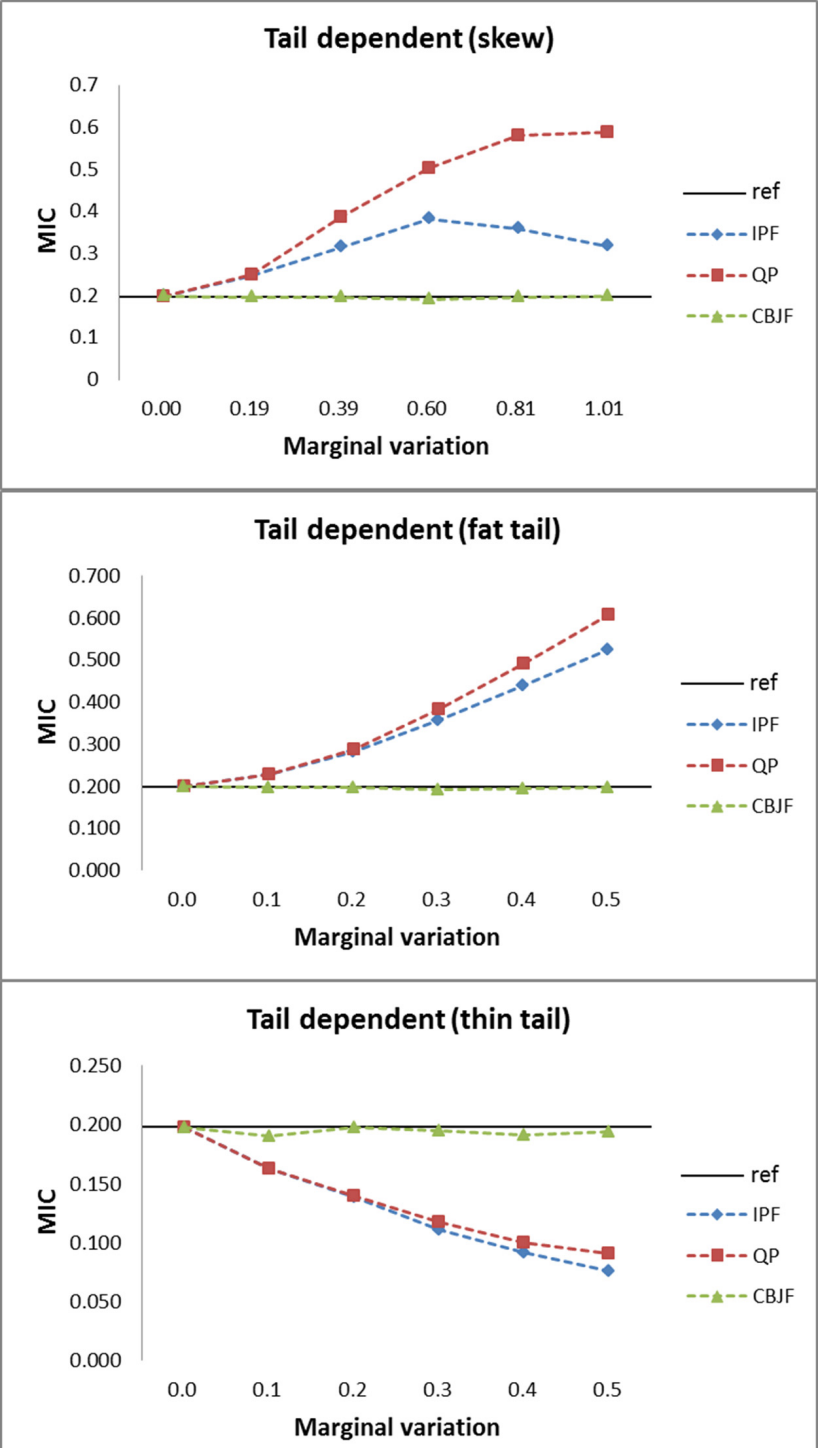
Results from the tail dependent joint distribution with skew, fat tail and thin tail operators.

**Fig 12:**
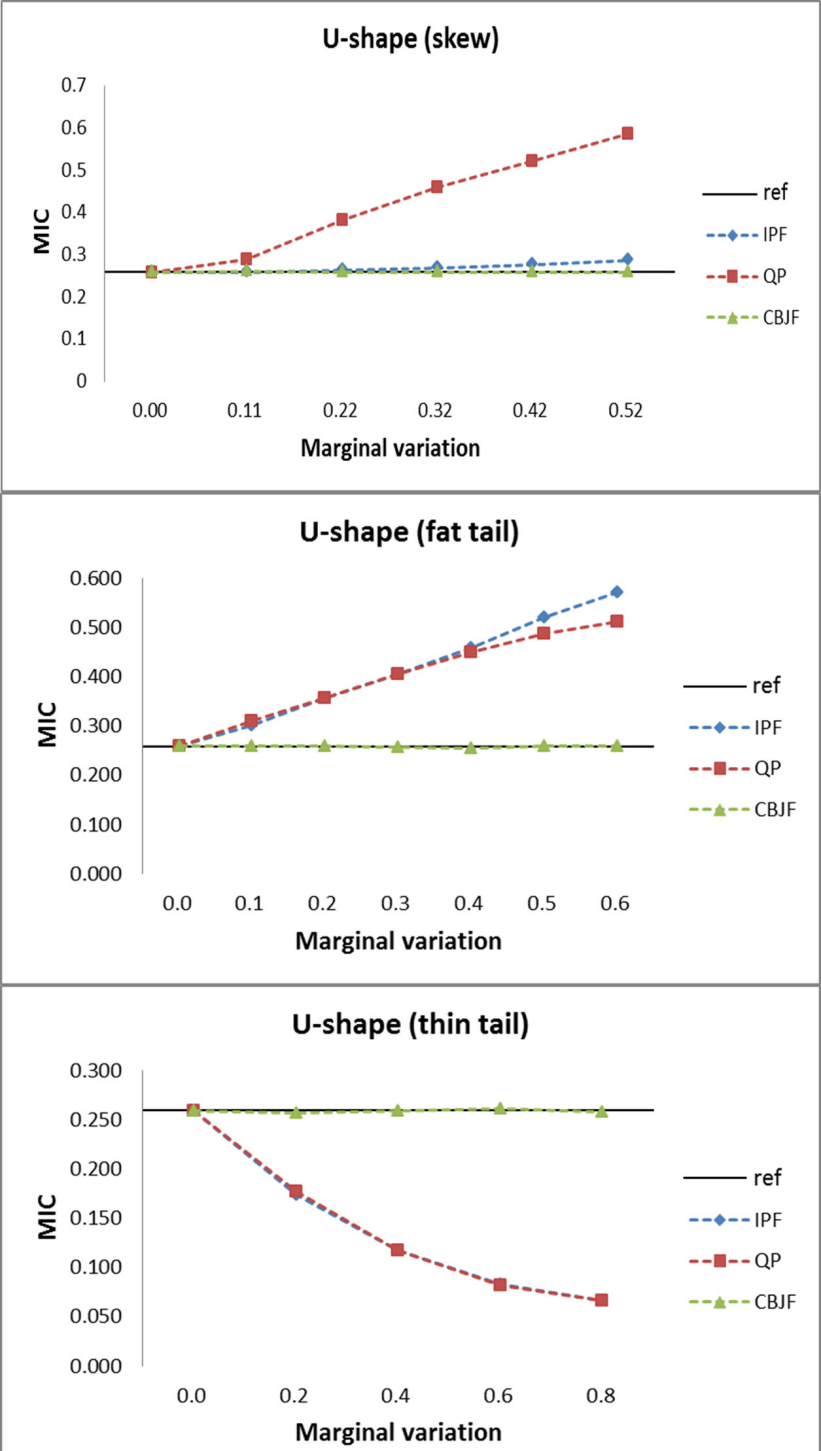
Results from the U-shape joint distribution with skew, fat tail and thin tail operators.

**Fig 13:**
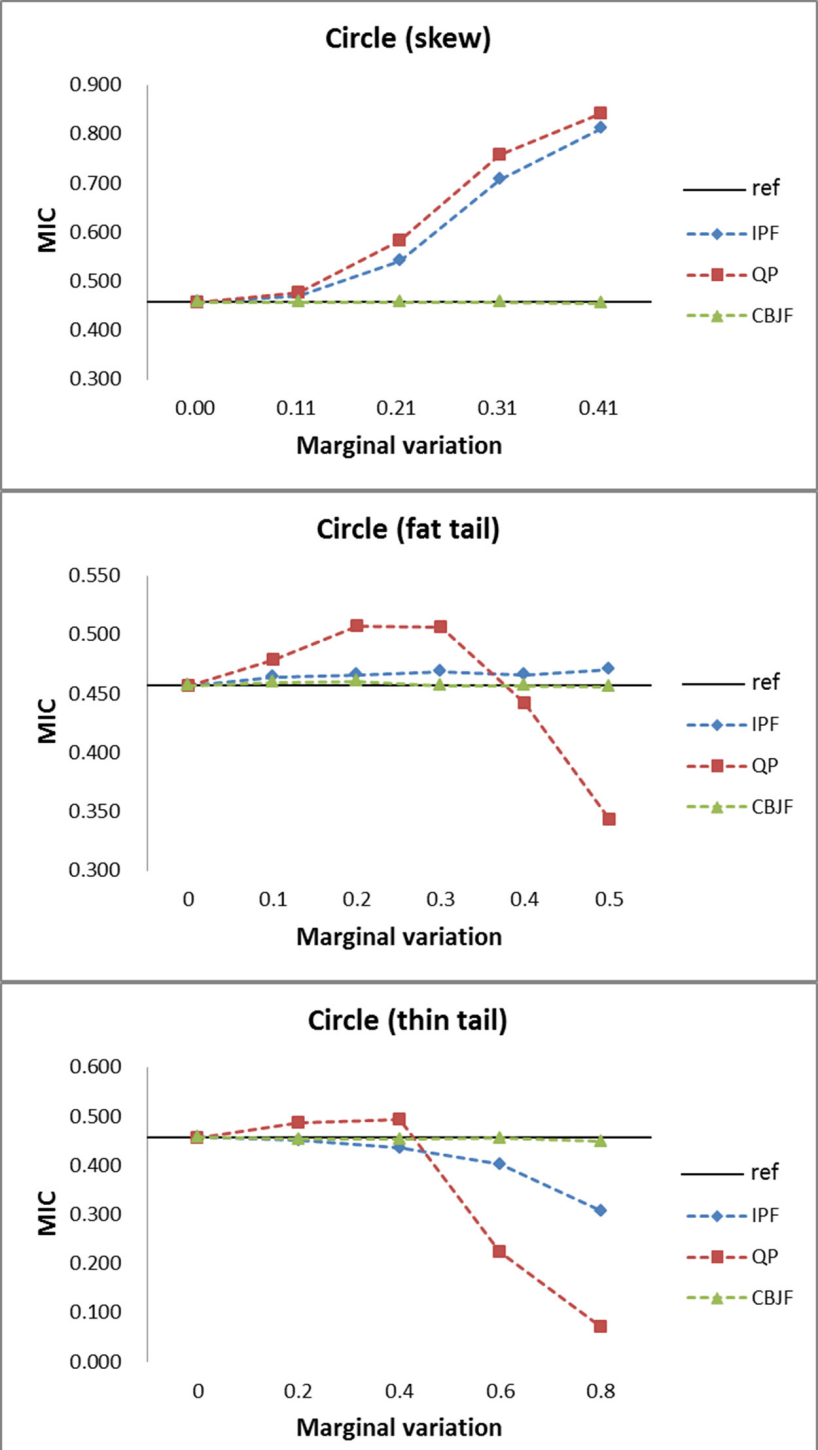
Results from the circle joint distribution with skew, fat tail and thin tail operators.

From the results shown in Figs 9–13, we can see that all the methods perform well in maintaining the reference’s dependence structure when the marginal variation is very small. However, as the variation gets bigger, IPF and QP fail to preserve reference joint distribution’s dependence structure measured by MIC. On the other hand, MIC of the CBJF output remains almost unchanged as the level of marginal variation increases.

**Fig 14:**
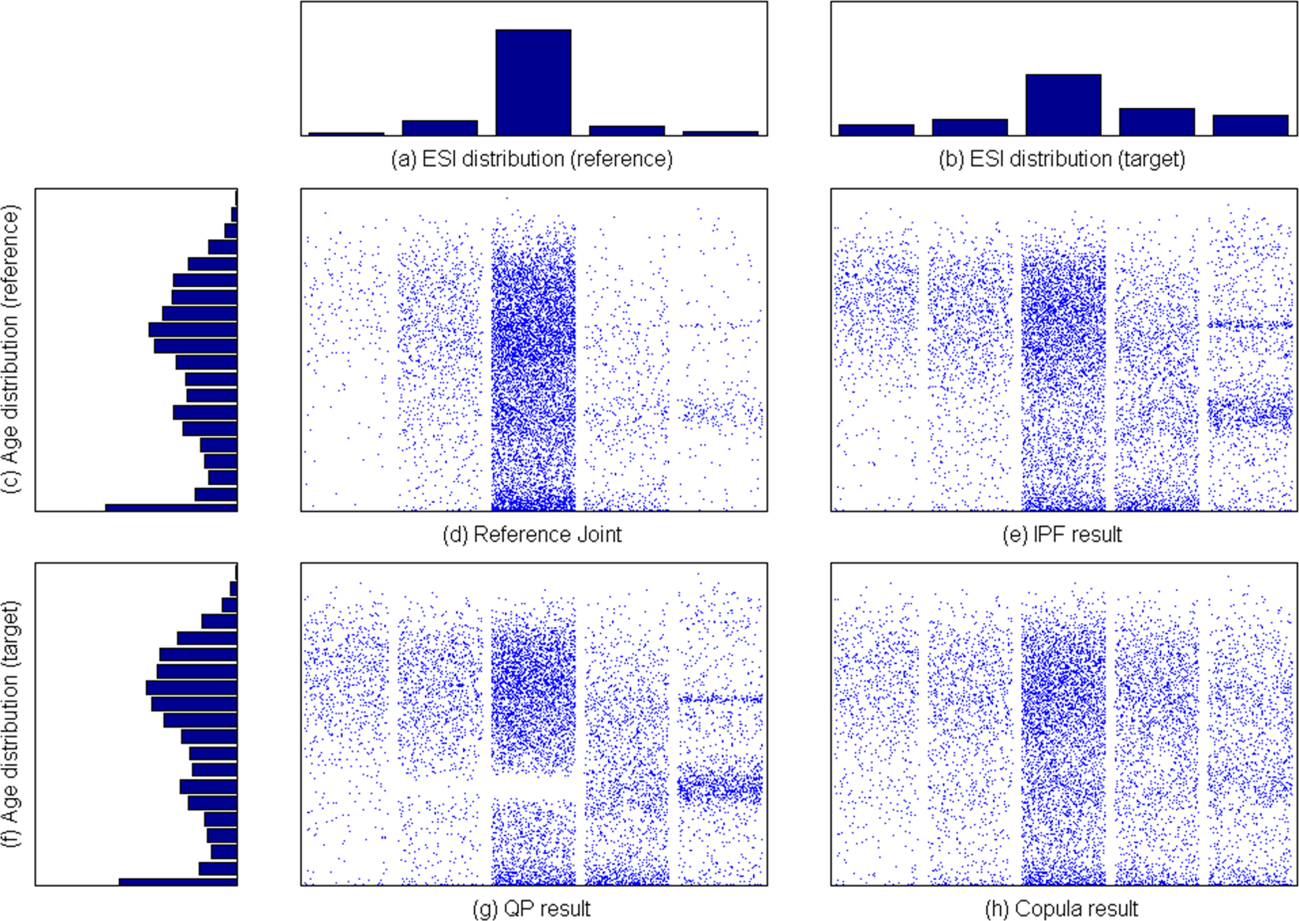
Synthetic population for ED(emergency department) simulation.

Finally, we applied the proposed method to generating synthetic patient population for simulating emergency department of a large hospital in Korea. Reference joint samples are obtained from more than 100K patient visit records during the year of 2013. Since the purpose of simulation analysis is to evaluate layout, patient admission policy, and staffing of the emergency department, we need to generate synthetic patient population which reflects the forecasted change in marginal distribution of patient age and severity of disease. As reported in [23], South Korea is one of the most rapidly aging society. ESI (Emergency Severity Index) is a 5-level triage system which classifies patients from level 1 (most urgent) to level 5 (least urgent) by both acuity and resource needs [24]. In this simulation study, the hospital wanted to analyze the capability of its emergency department in various situations, and hence the distribution of ESI was varied. Fig 14 shows the reference patient population in 2013 and synthetic populations generated by IPF, QP, and CBJF. In this study, target marginal distribution of age reflects the forecast in Statistics Korea [23], where the portion of elderly people (60+) is predicted to reach 42% in 2025 (from 31% in the reference population of 2013). In order to see the effect of reducing the patient concentration at ESI level 3, the distribution of ESI is changed from (2%, 11%, 78%, 6%, 3%) to (8%, 12%, 45%, 20%, 15%). As shown in Table 8, CBJF outperforms IPF and QP in preserving all dependency measures used in this study.

**Table 8:** Dependency measures for synthetic ED patient populations.

Dependency measure		Reference	IPF	QP	Copula
Pearson	Observed	-0.171	-0.260	-0.291	-0.144
	Abs. Dev.		0.089	0.120	0.027
Spearman	Observed	-0.165	-0.294	-0.314	-0.146
	Abs. Dev.		0.129	0.149	0.019
Kendall	Observed	-0.089	-0.179	-0.203	-0.086
	Abs. Dev.		0.090	0.114	0.003
MIC	Observed	0.031	0.098	0.209	0.019
	Abs. Dev.		0.067	0.178	0.012

## Summary and Concluding Remarks

The joint fitting problem turned out to be a natural application area of the copula concept so as to preserve the dependence structure of the reference distribution. In this paper, a novel method based on the copula concept, called CBJF, was proposed. Although IPF has long been used in a wide range of applications and studied with mathematical rigor, it is not a silver bullet for joint fitting problems including synthetic population generation. From the numerical tests, it was found that CBJF is superior to IPF or QP methods in most cases for the dependence structure preservation. Furthermore, CBJF is computationally efficient since it does not require iterative procedure. Also, its robustness is a significant advantage of CBJF as it does not need to consider the convergence problem or zero cells.

A disadvantage of CBJF is that it requires caution when X and/or Y are categorical variables, such as gender or ethnic groups. In such cases, CBJF result is affected by the ordering of the rows (or columns) when there is no definite natural ordering of the attribute values. In its present form, CBJF can be applied when the attribute values have a natural ordering, such as age, annual income, number of vehicles, or location coordinates. Handling categorical variables is a topic requiring further research. When categorical target variables are mixed with ordinal target variables, IPF may be applied first to the categorical variables and then CBJF can work on the remaining ordinal variables.

In many cases of agent-based simulation applications, micro-samples (such as PUMS) from the reference population may not be available mainly because of cost of survey or privacy issues. There are recent researches on generating synthetic population without micro-samples [25–27]. We believe the concept of CBJF can be applied even when micro-samples are not available, however the details depend of the available information and require further research.

## Supporting Information

S1 Fig Experiments on 40 test cases. (PDF)

S2 Fig Experiments on various changes on marginal distributions. (PDF)

S1 Text Proofs for Lemma 1 & Lemma 2. (PDF)
