# Supplementary material for: Copula-Based Approach to Synthetic Population Generation
Source: PLoS One. 2016 Aug 4;11(8):e0159496. doi: 10.1371/journal.pone.0159496 (PMC4973930; doi:10.1371/journal.pone.0159496)

S1 Fig. Experiments on 40 test cases : (5 reference joint distribution types) X (8 marginal modification operators)

1-1-1. Reference joint distribution : Bivariate normal, Target marginal type : Skew LL

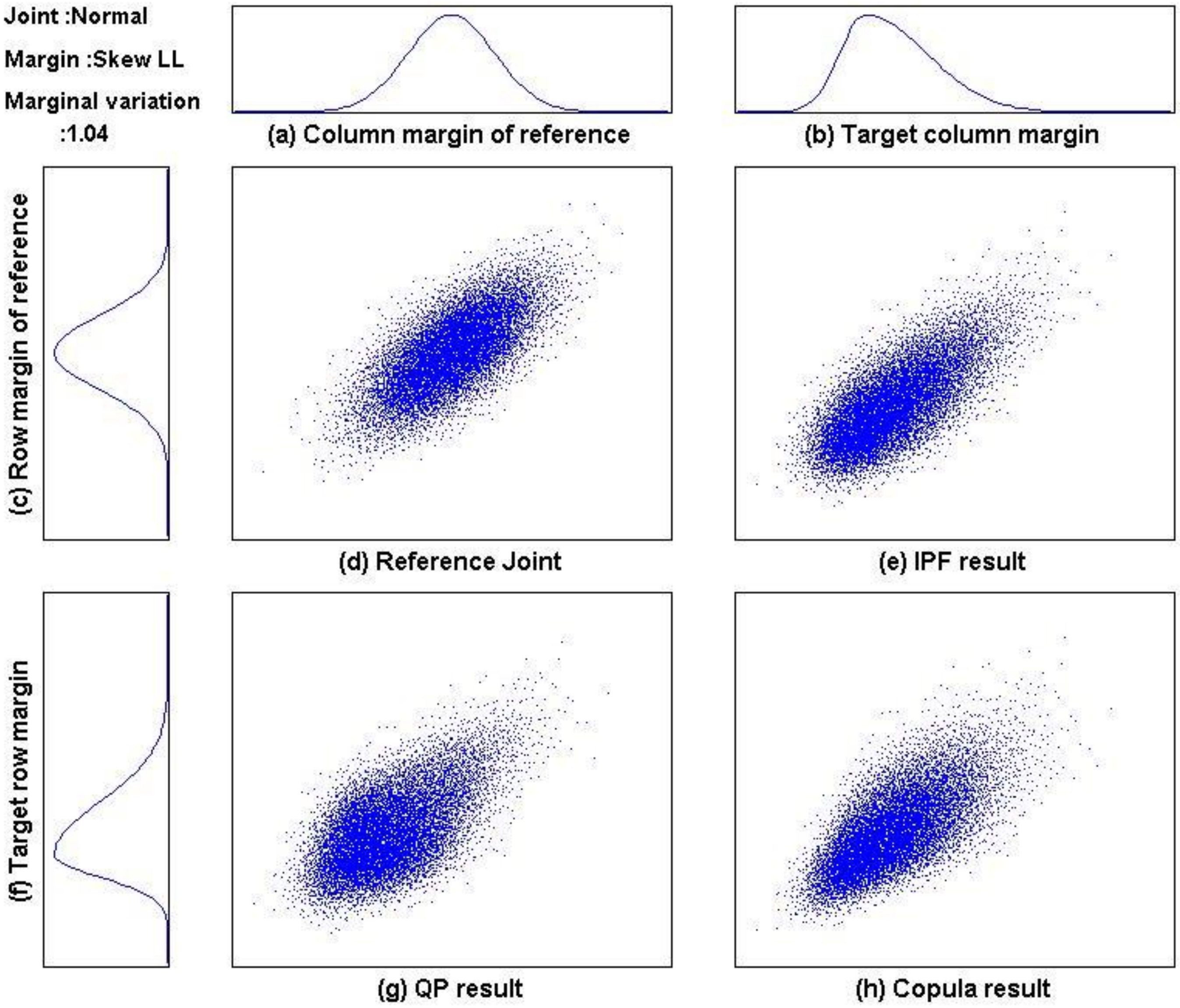

1-1-2. Reference joint distribution : Bivariate normal, Target marginal type : Skew RR

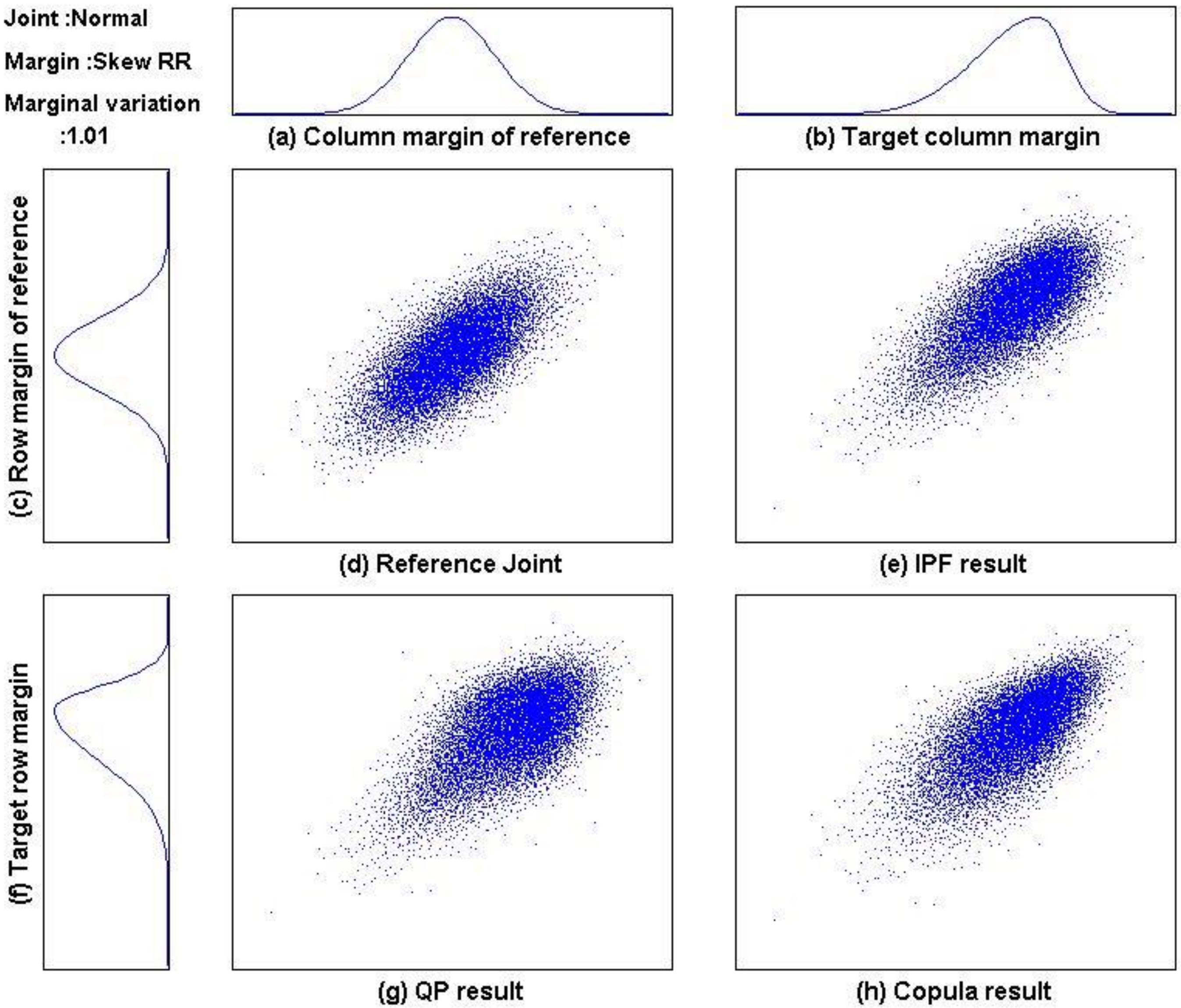

1-1-3. Reference joint distribution : Bivariate normal, Target marginal type : Skew LR

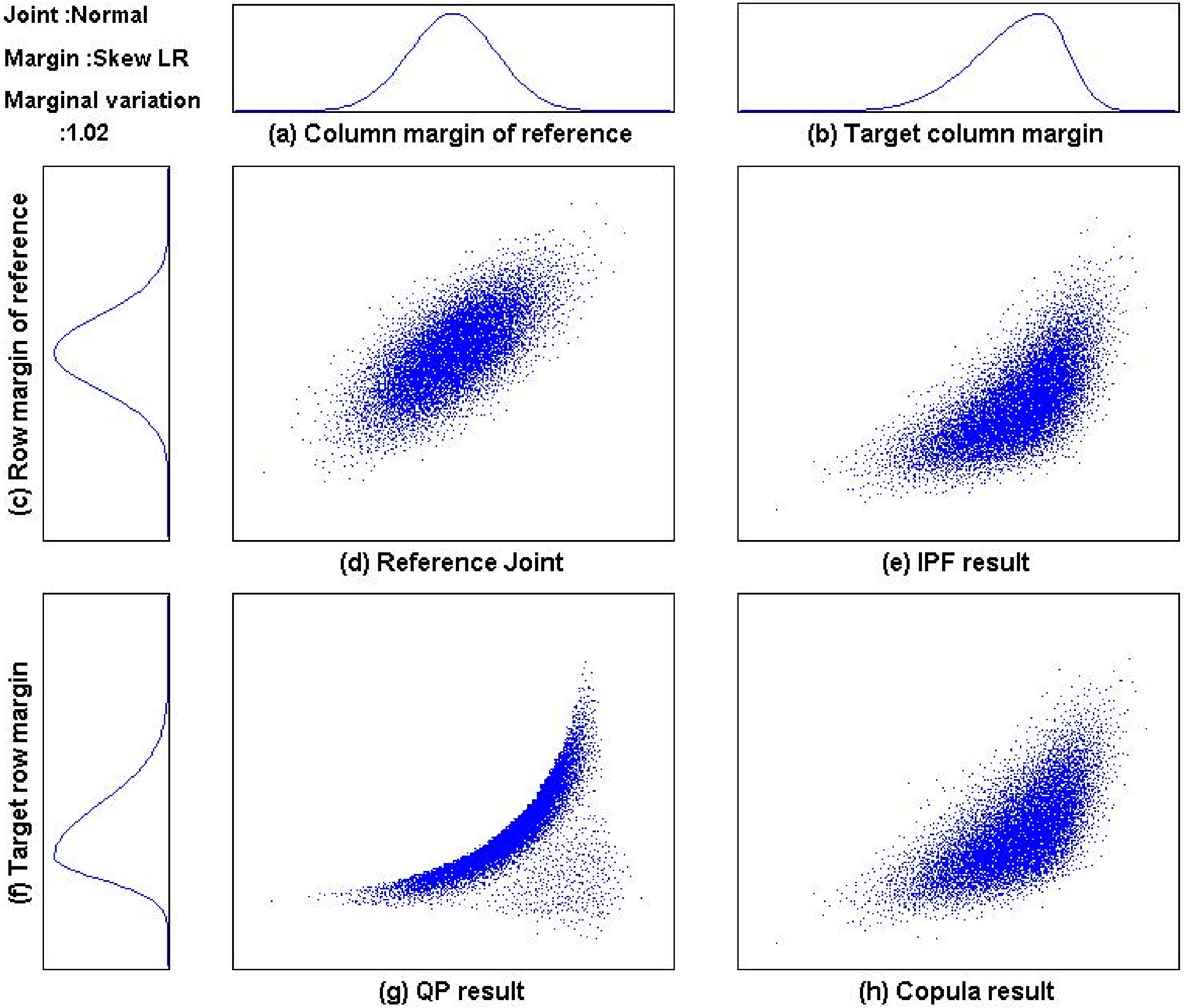

1-1-4. Reference joint distribution : Bivariate normal, Target marginal type : Skew RL

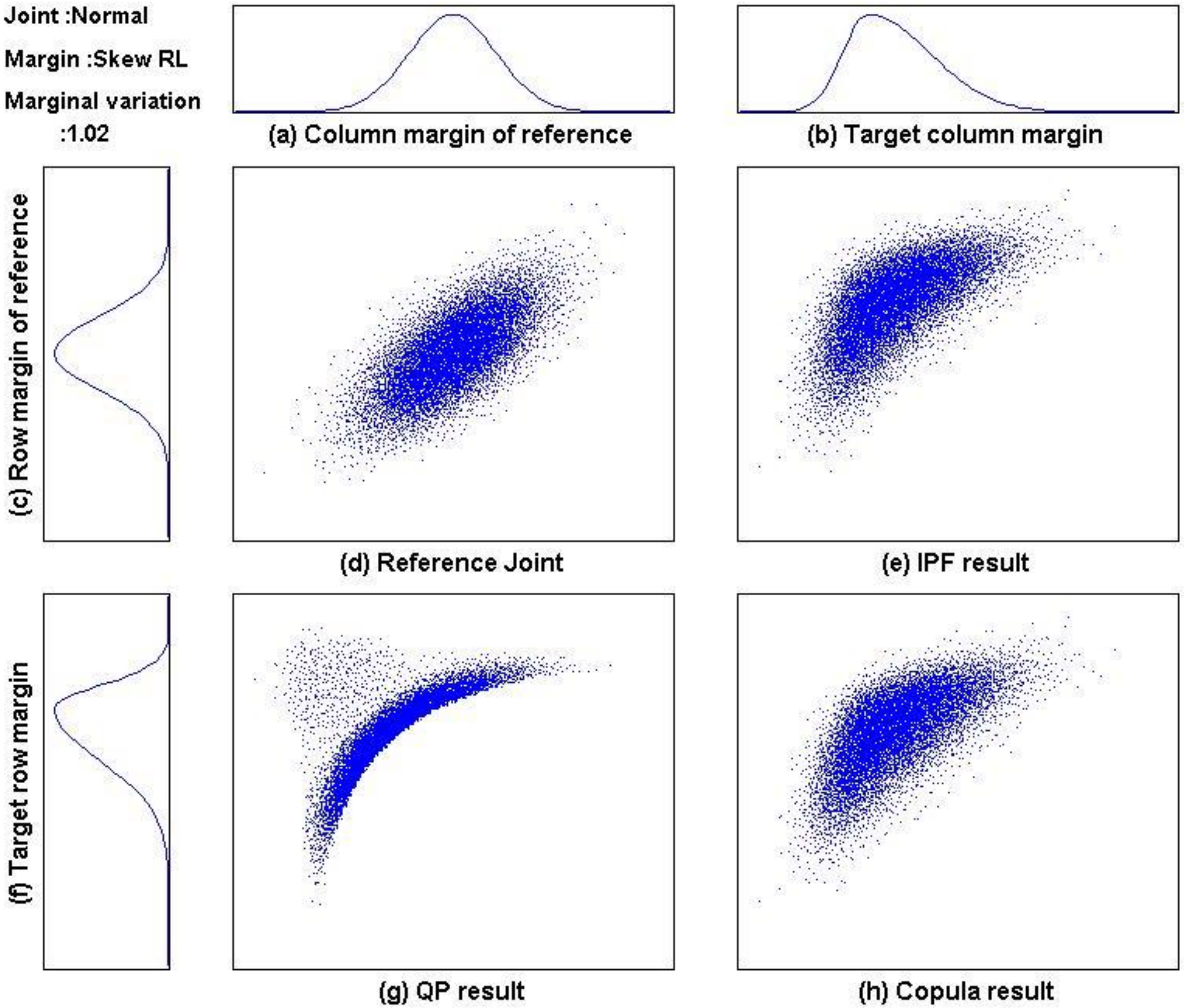

1-1-5. Reference joint distribution : Bivariate normal, Target marginal type : Uniform

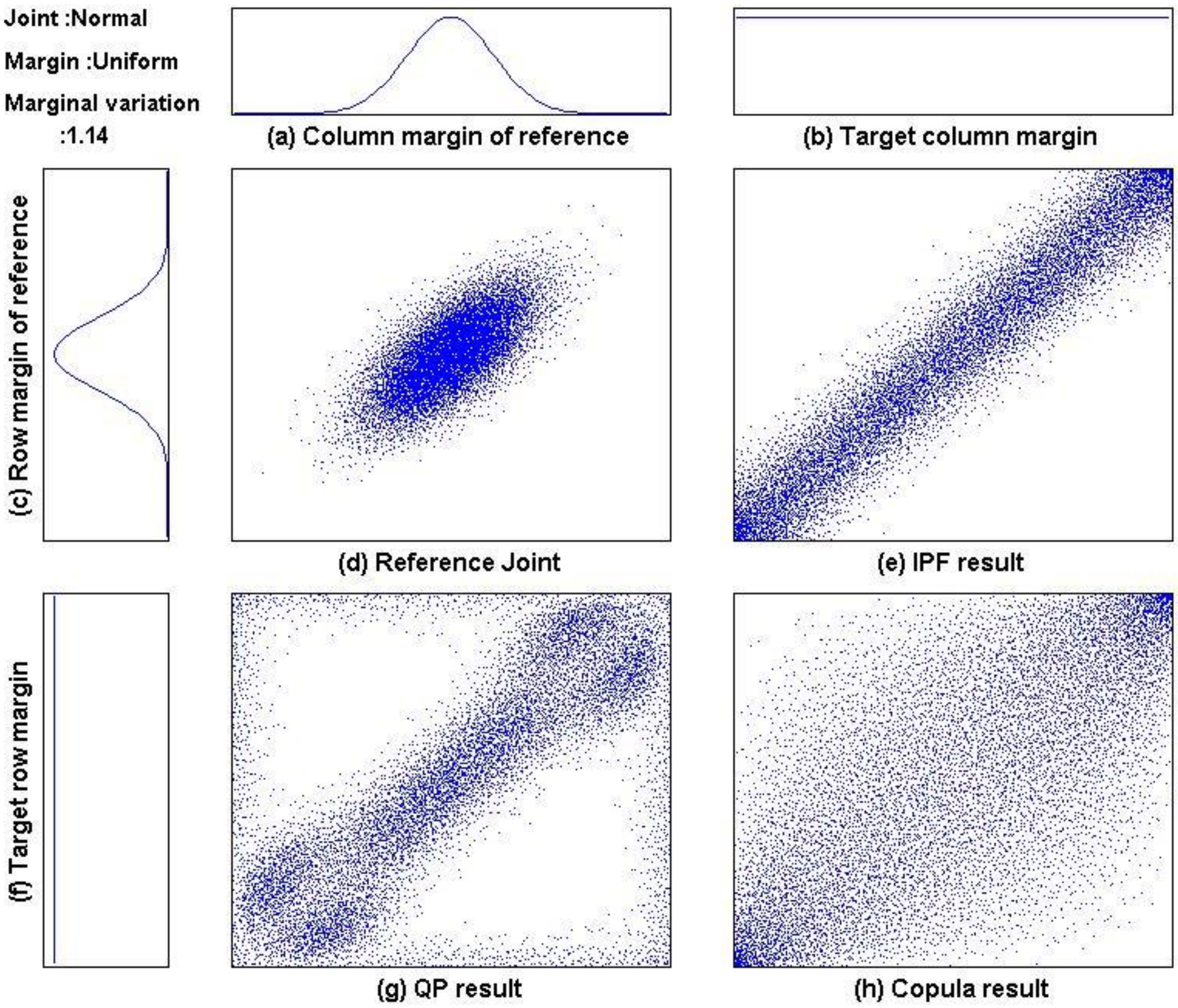

1-1-6. Reference joint distribution : Bivariate normal, Target marginal type : Fat tail

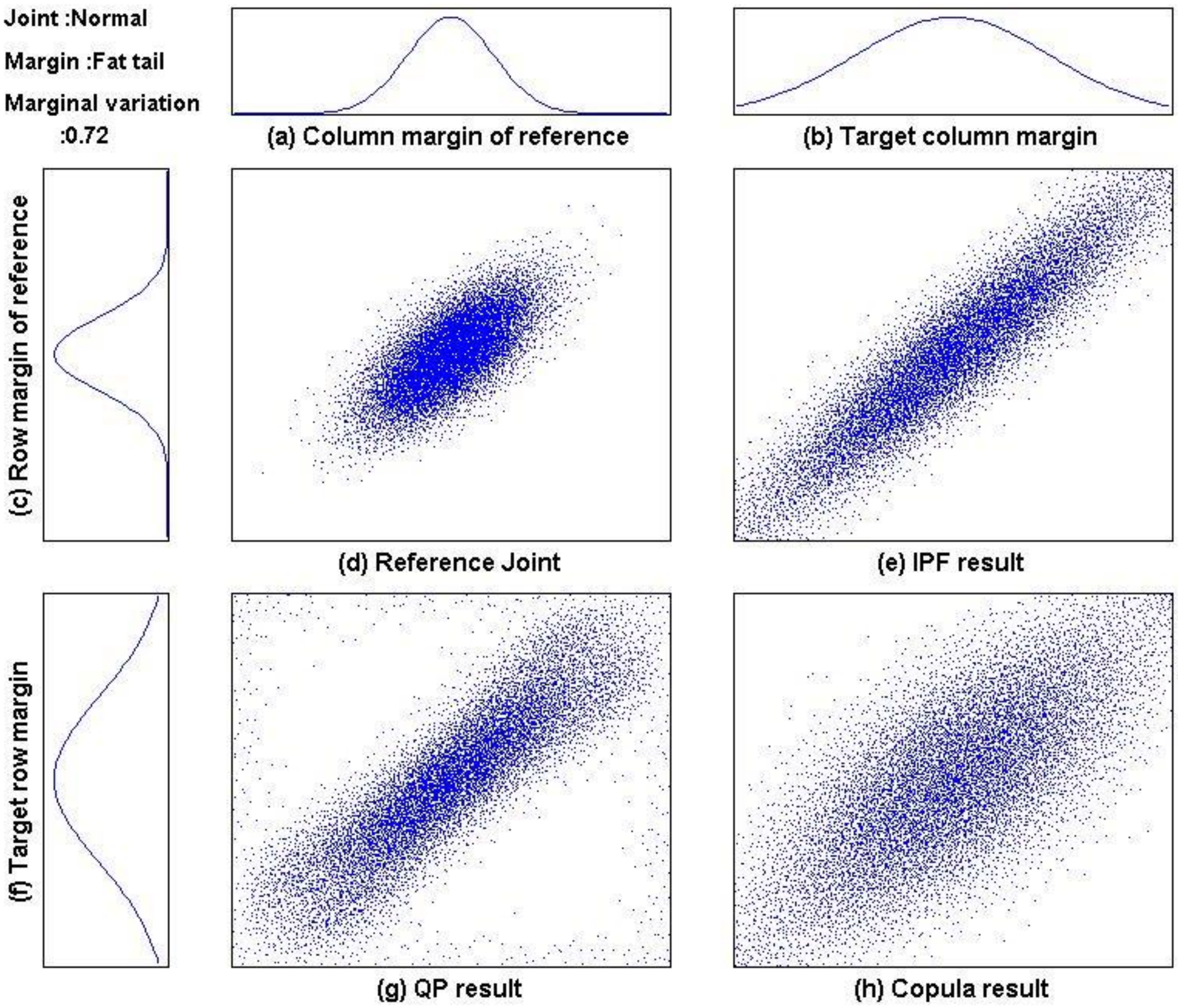

1-1-7. Reference joint distribution : Bivariate normal, Target marginal type : Thin tail

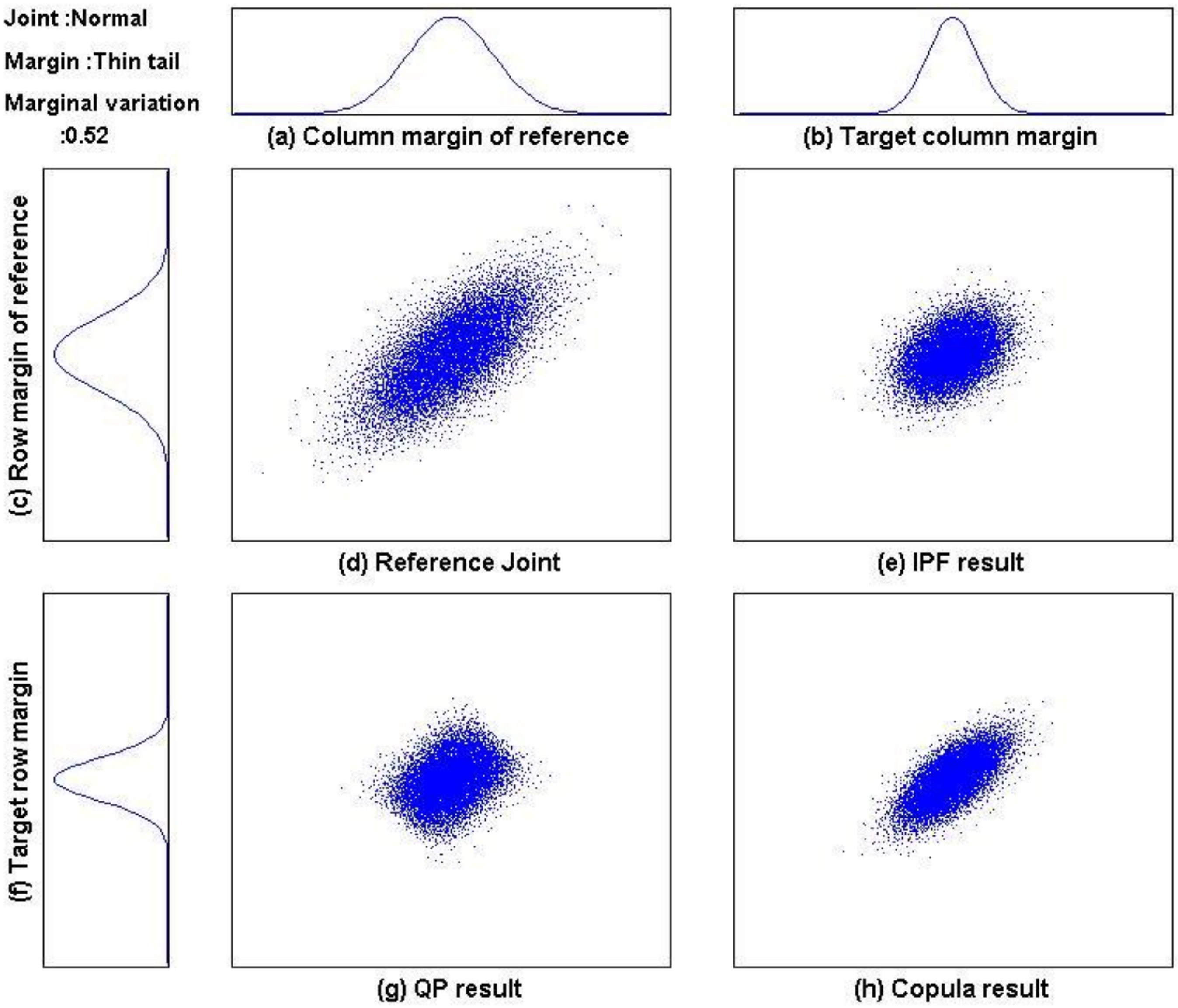

1-1-8. Reference joint distribution : Bivariate normal, Target marginal type : Perturbation

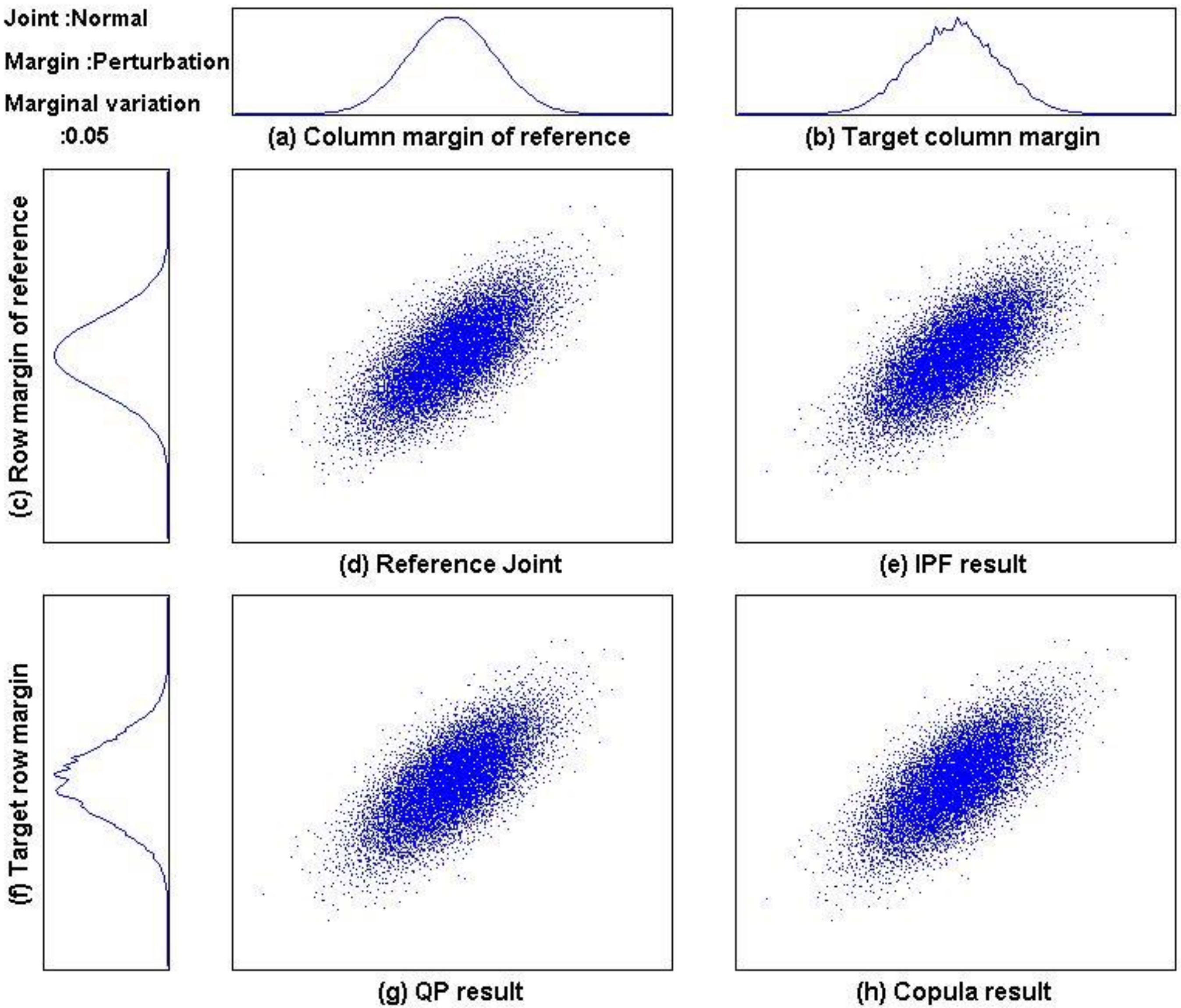

1-2-1. Reference joint distribution : Bimodal, Target marginal type : Skew LL

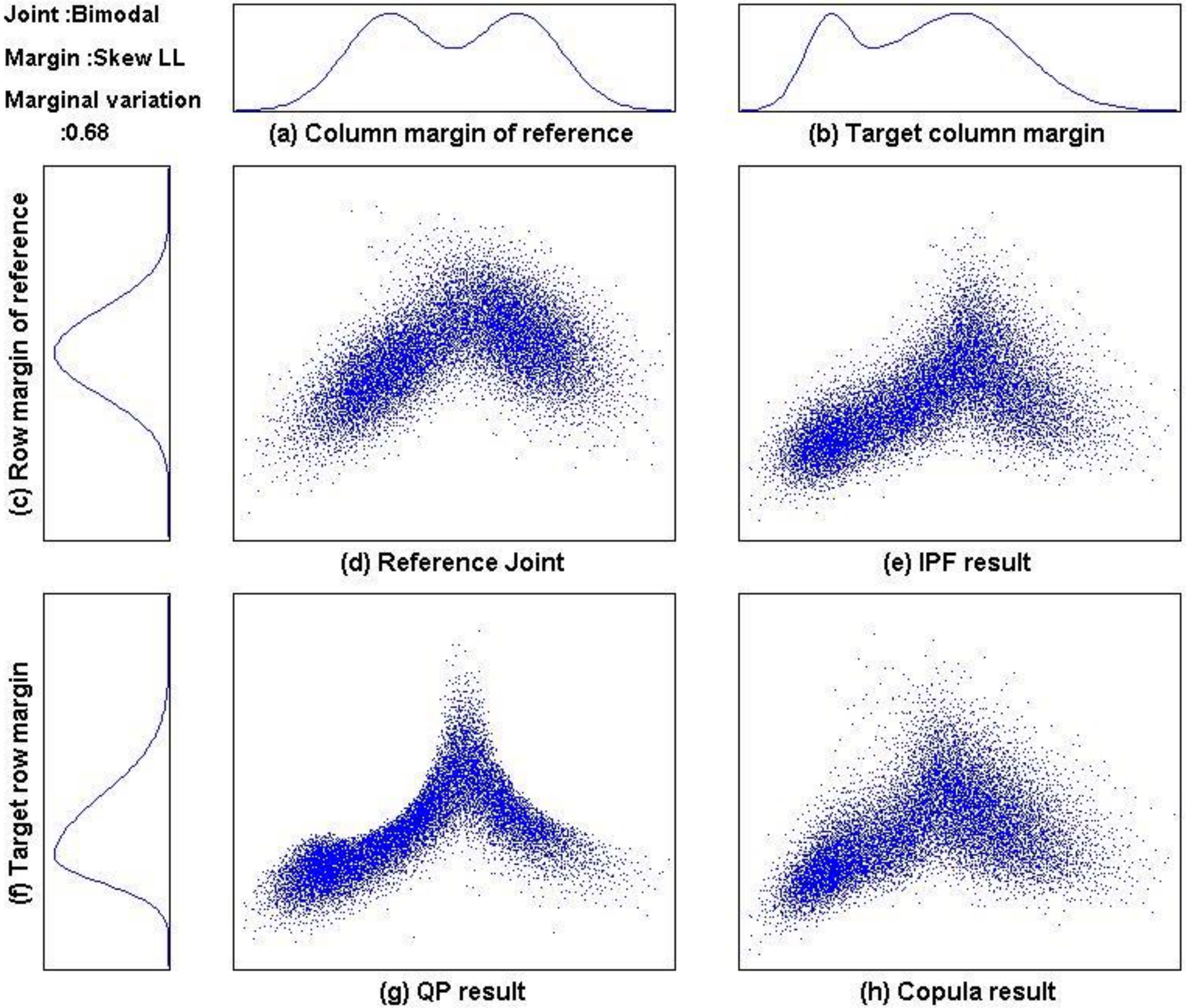

1-2-2. Reference joint distribution : Bimodal, Target marginal type : Skew RR

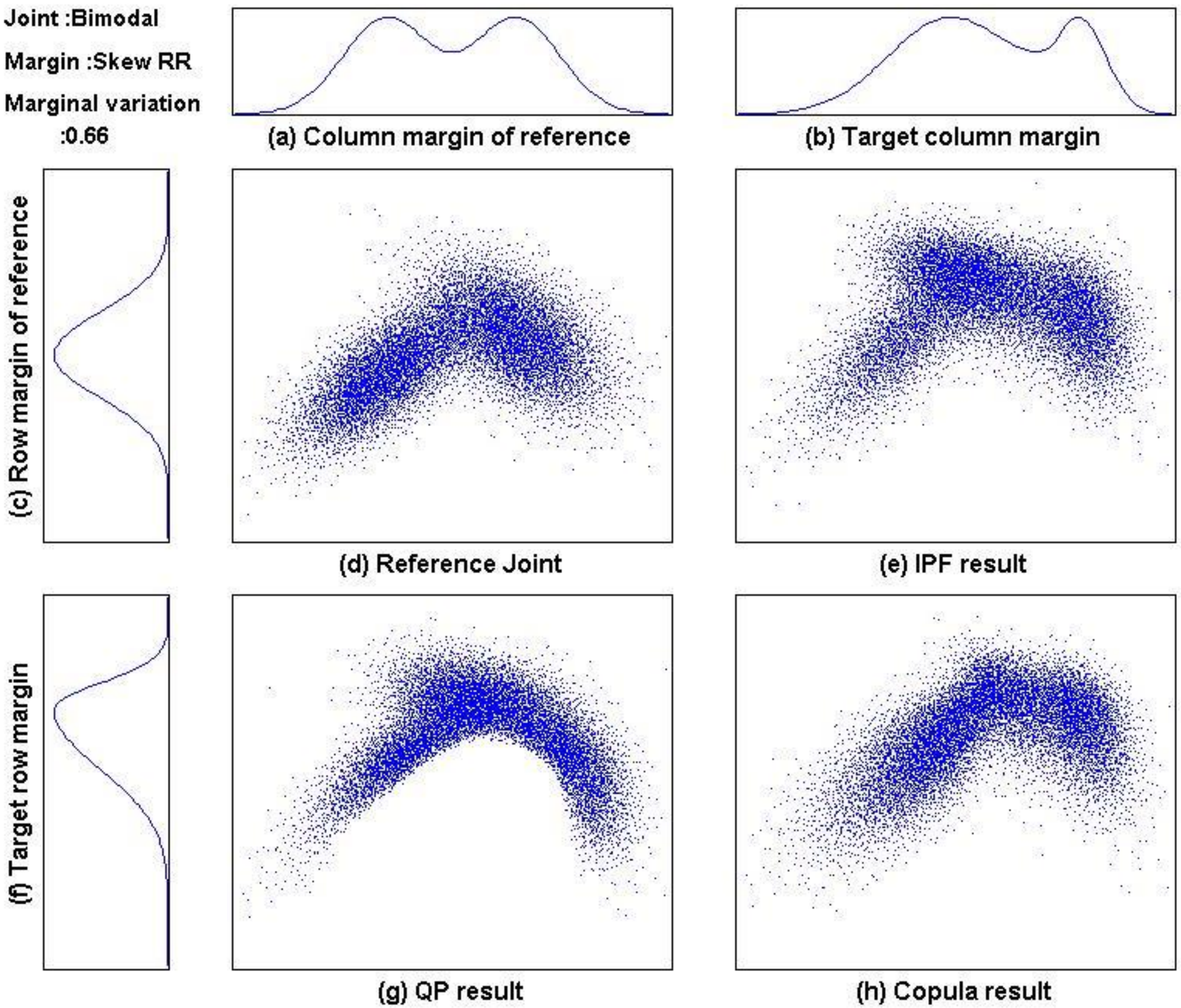

1-2-3. Reference joint distribution : Bimodal, Target marginal type : Skew LR

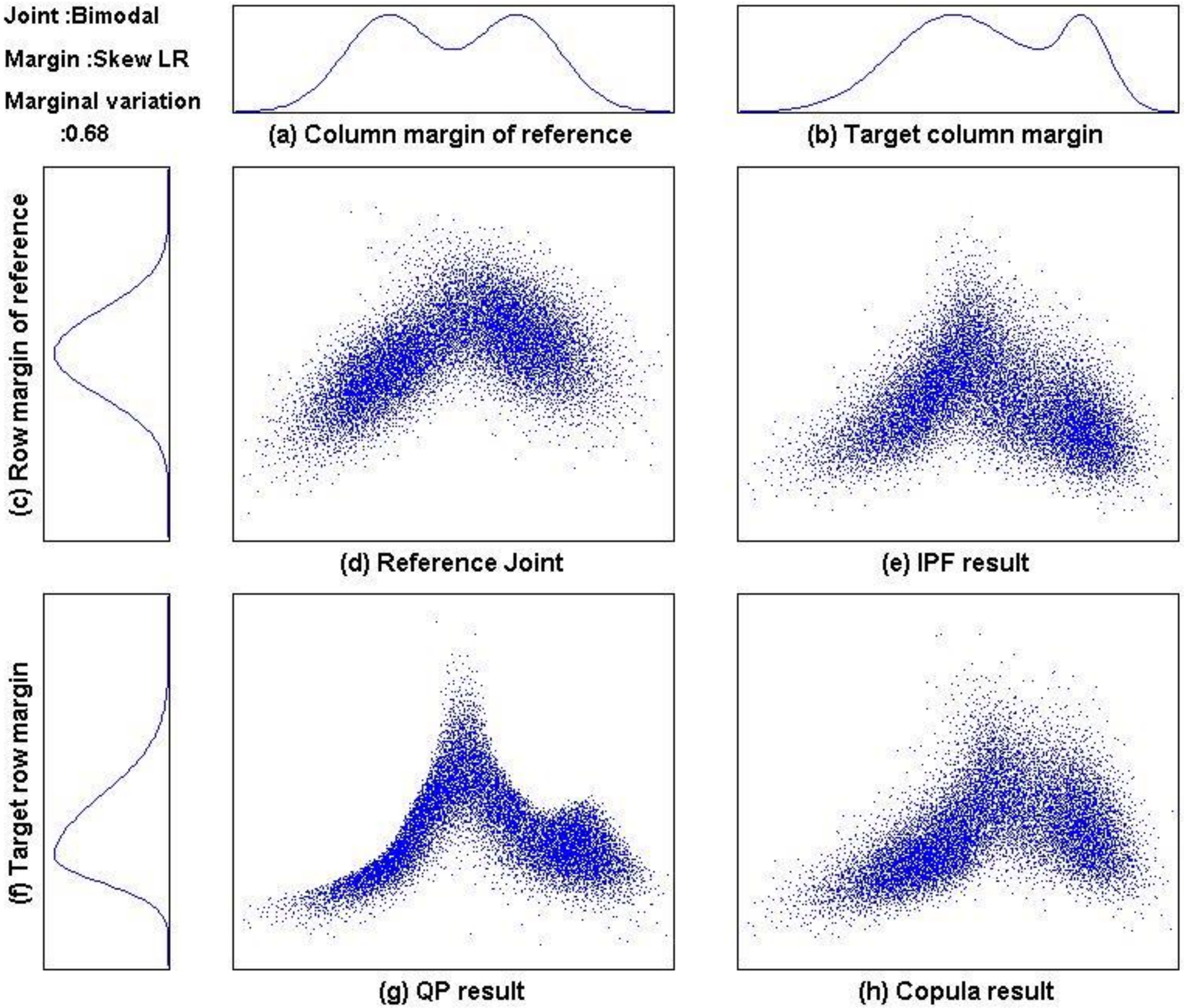

1-2-4. Reference joint distribution : Bimodal, Target marginal type : Skew RL

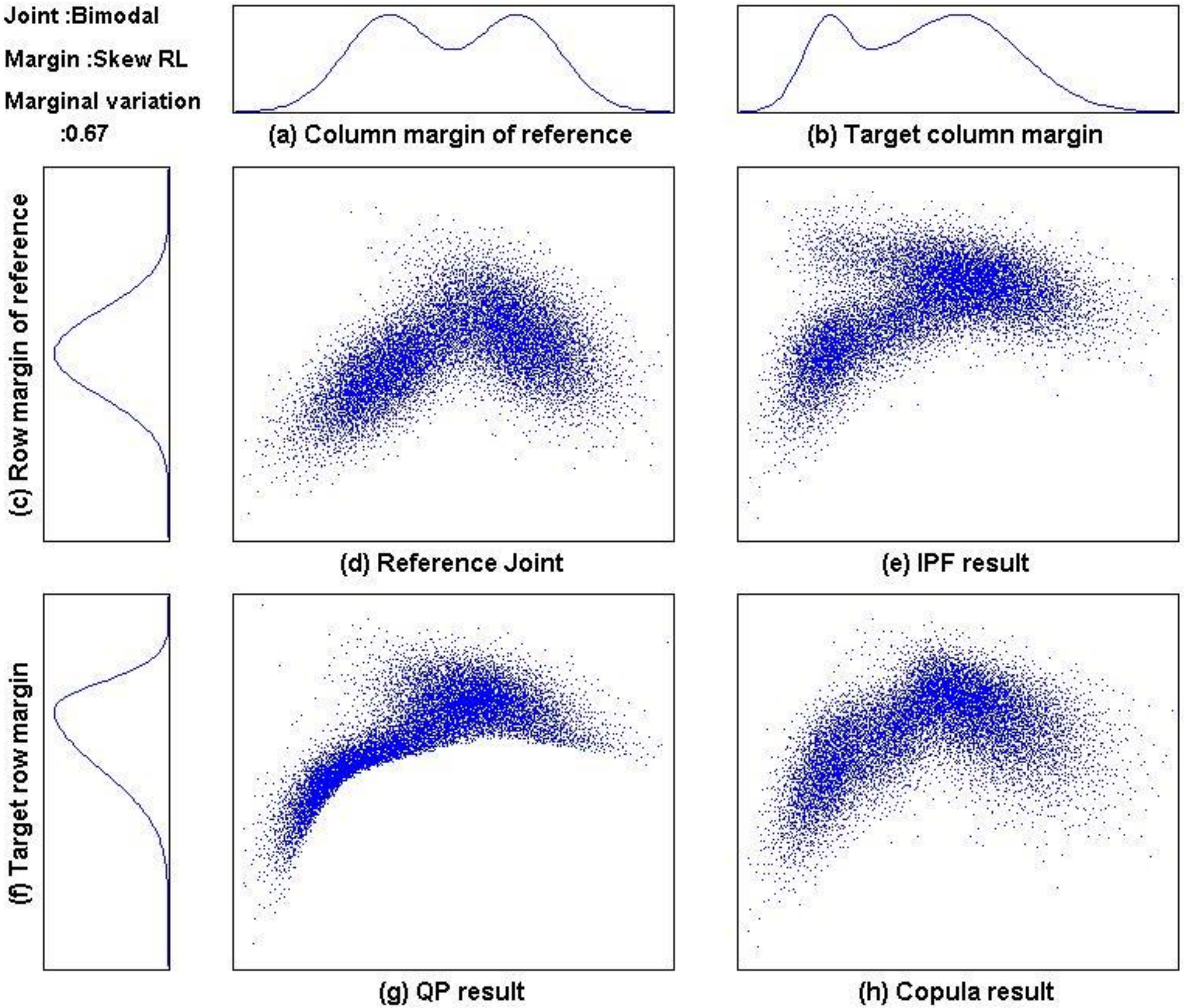

1-2-5. Reference joint distribution : Bimodal, Target marginal type : Uniform

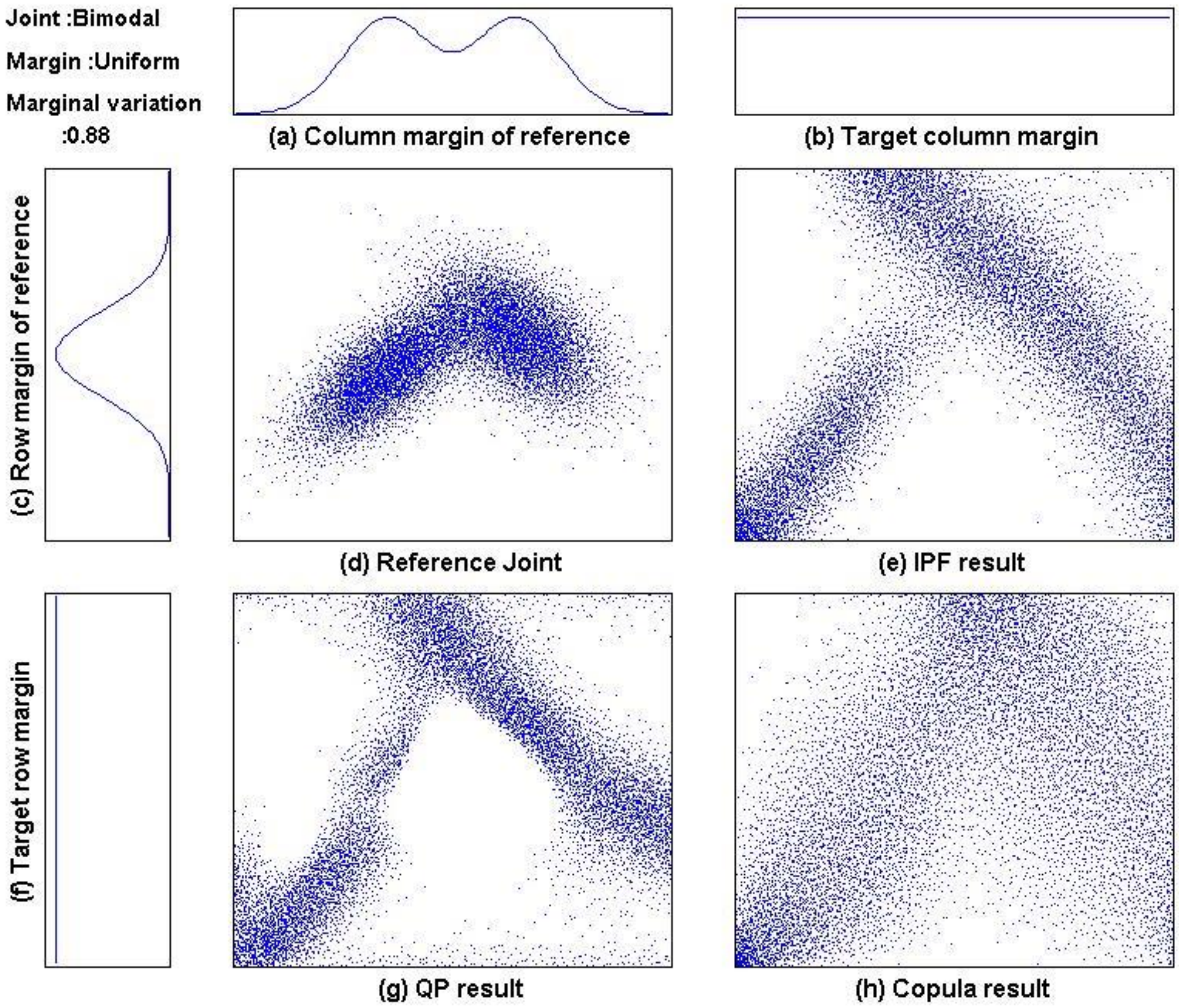

1-2-6. Reference joint distribution : Bimodal, Target marginal type : Fat tail

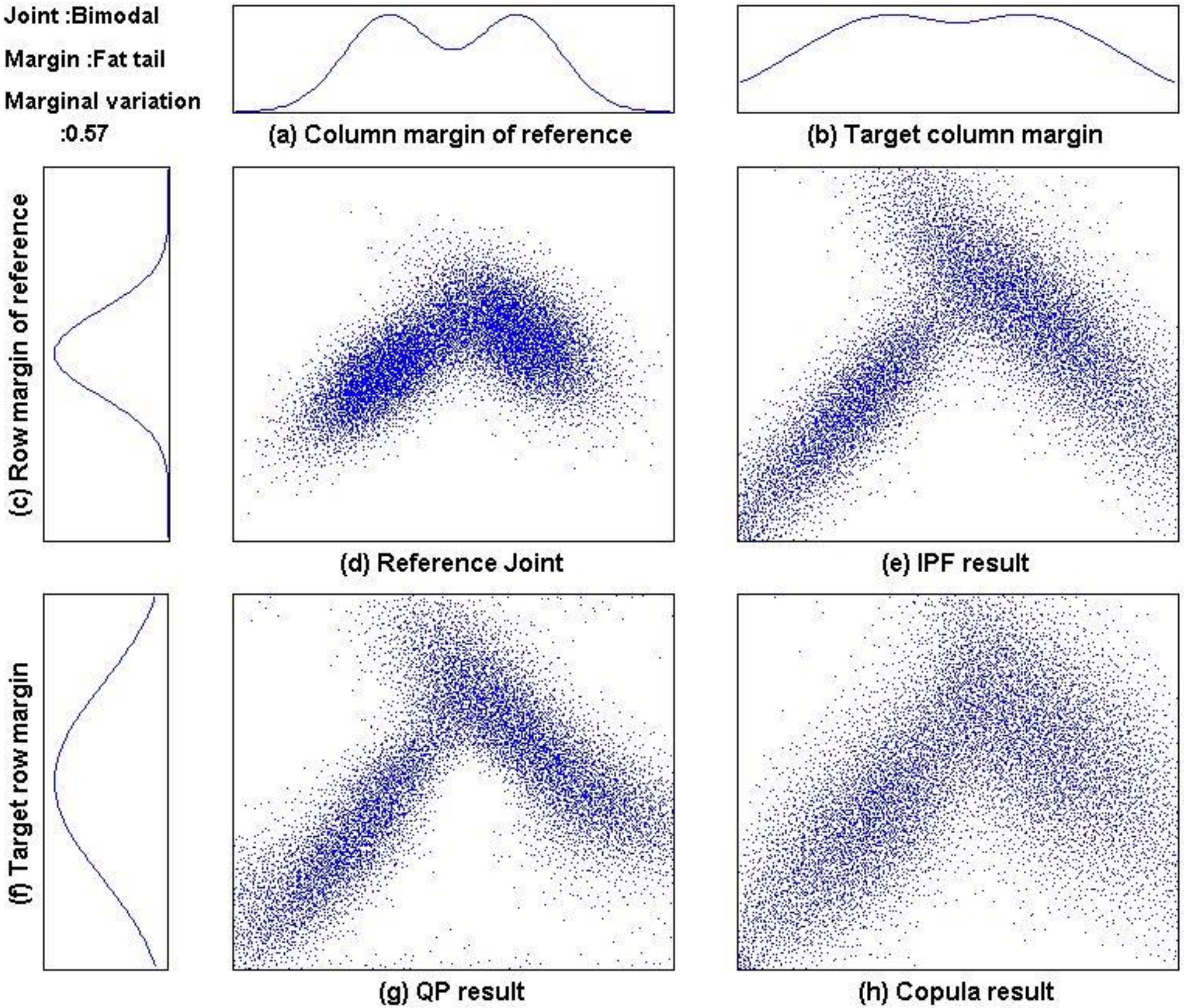

1-2-7. Reference joint distribution : Bimodal, Target marginal type : Thin tail

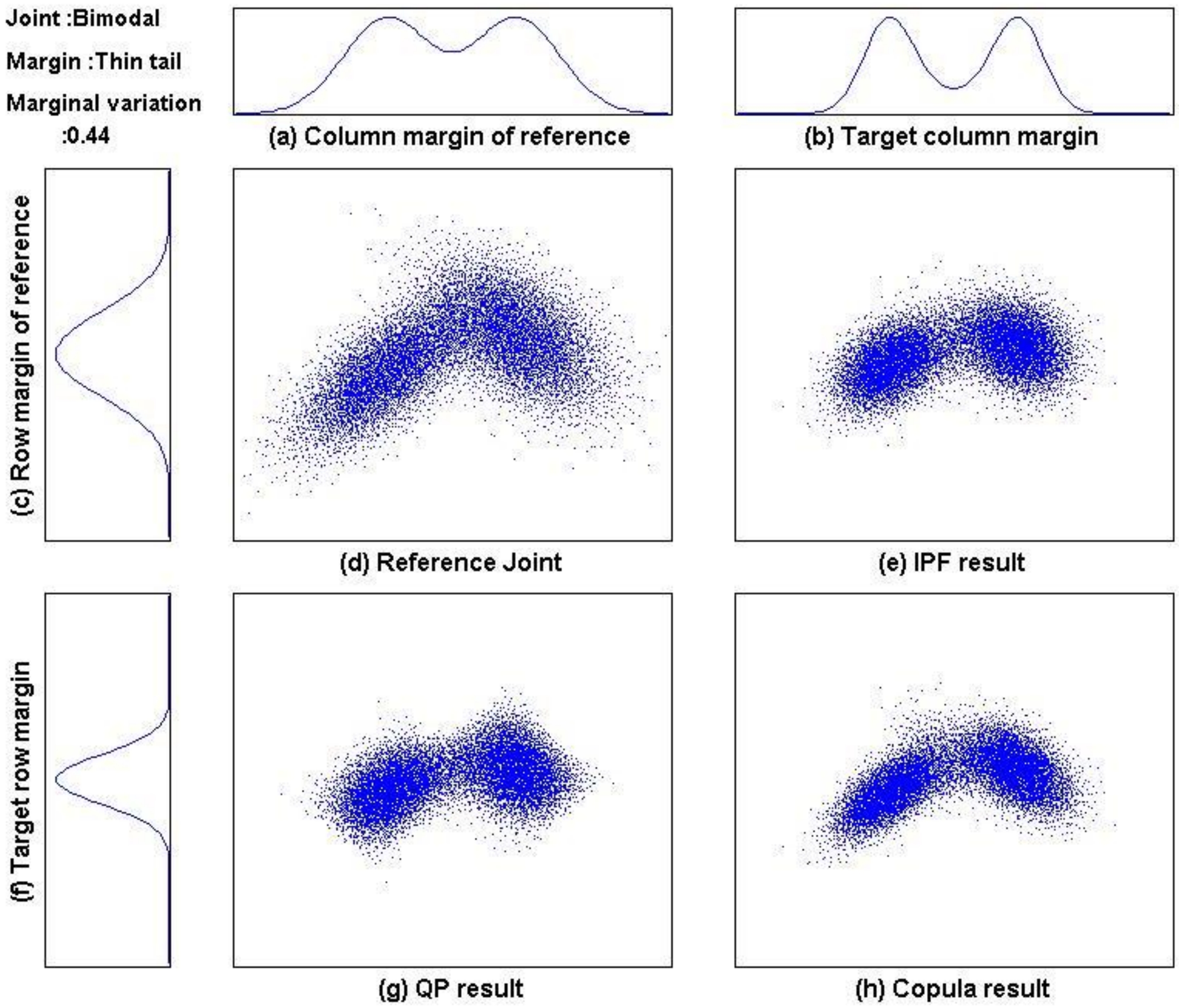

1-2-8. Reference joint distribution : Bimodal, Target marginal type : Perturbation

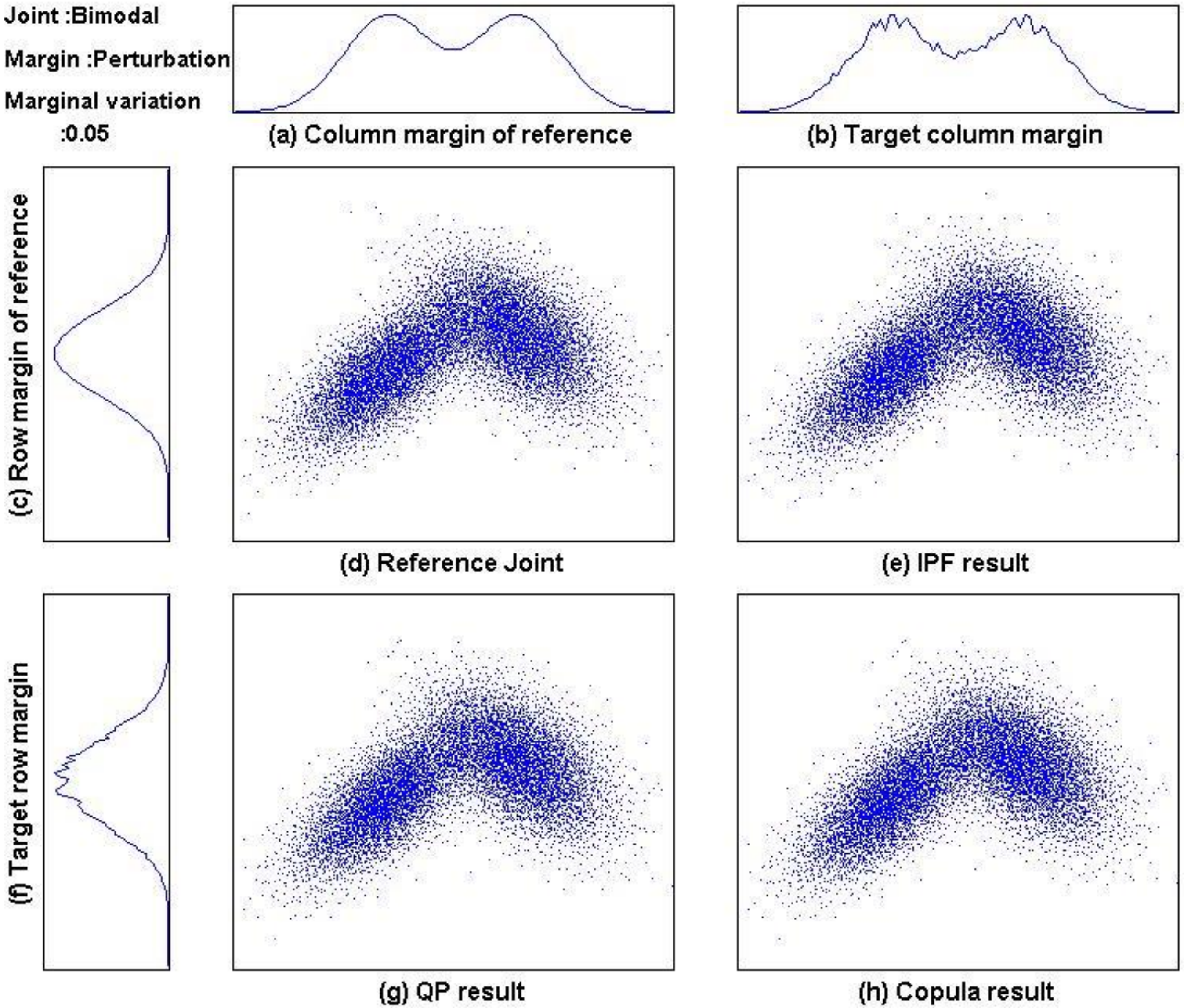

1-3-1. Reference joint distribution : Tail dependent, Target marginal type : Skew LL

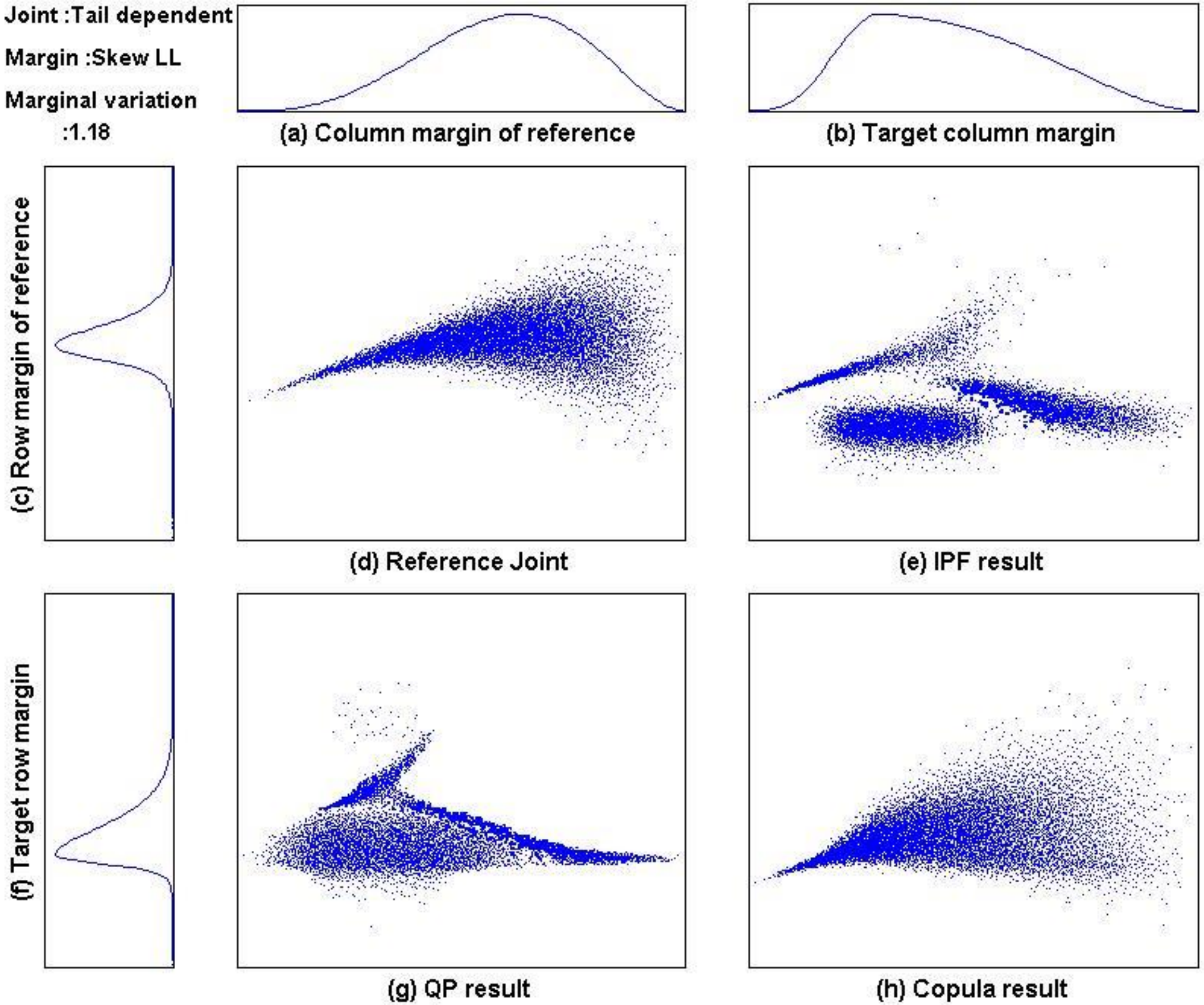

1-3-2. Reference joint distribution : Tail dependent, Target marginal type : Skew RR

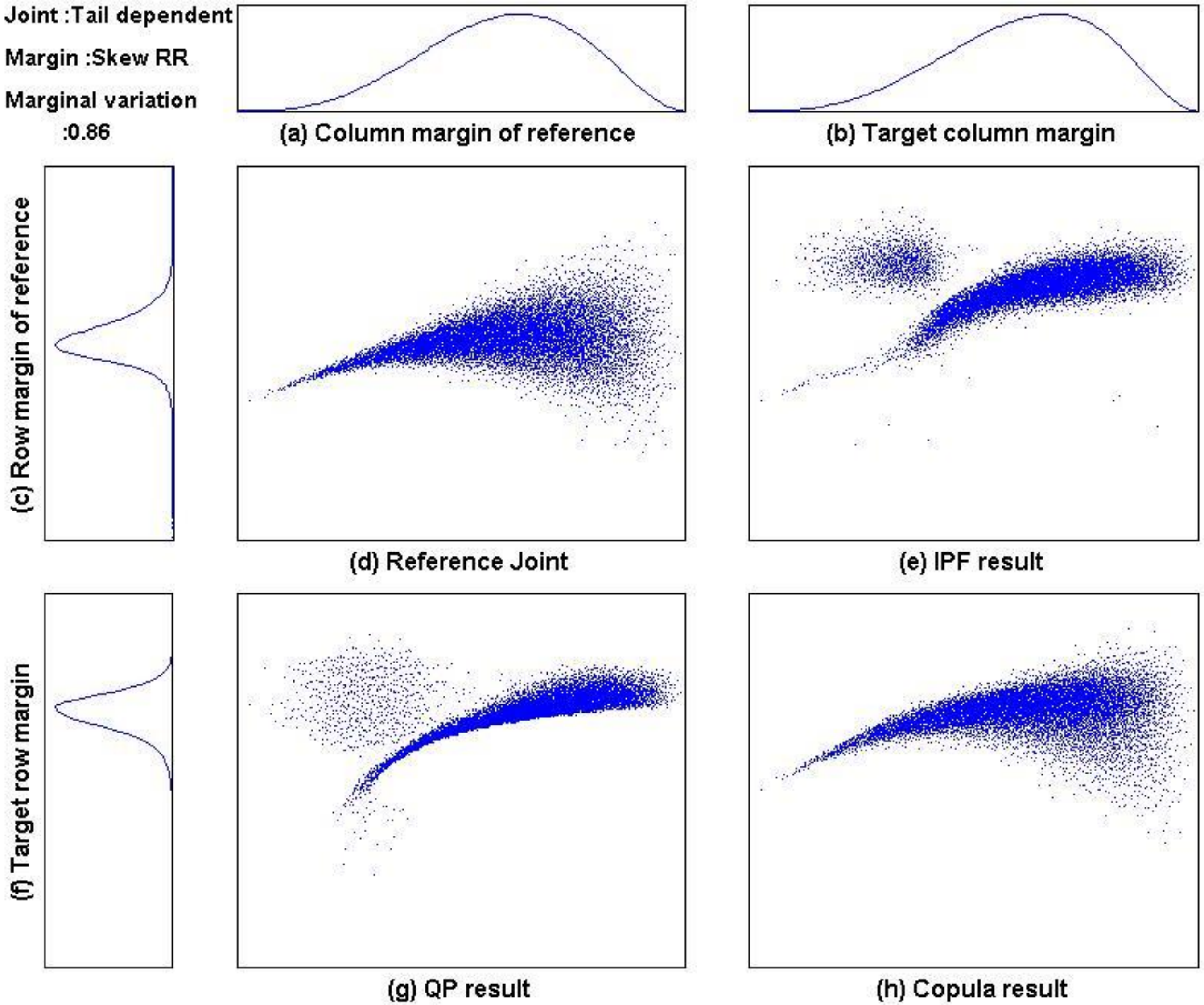

1-3-3. Reference joint distribution : Tail dependent, Target marginal type : Skew LR

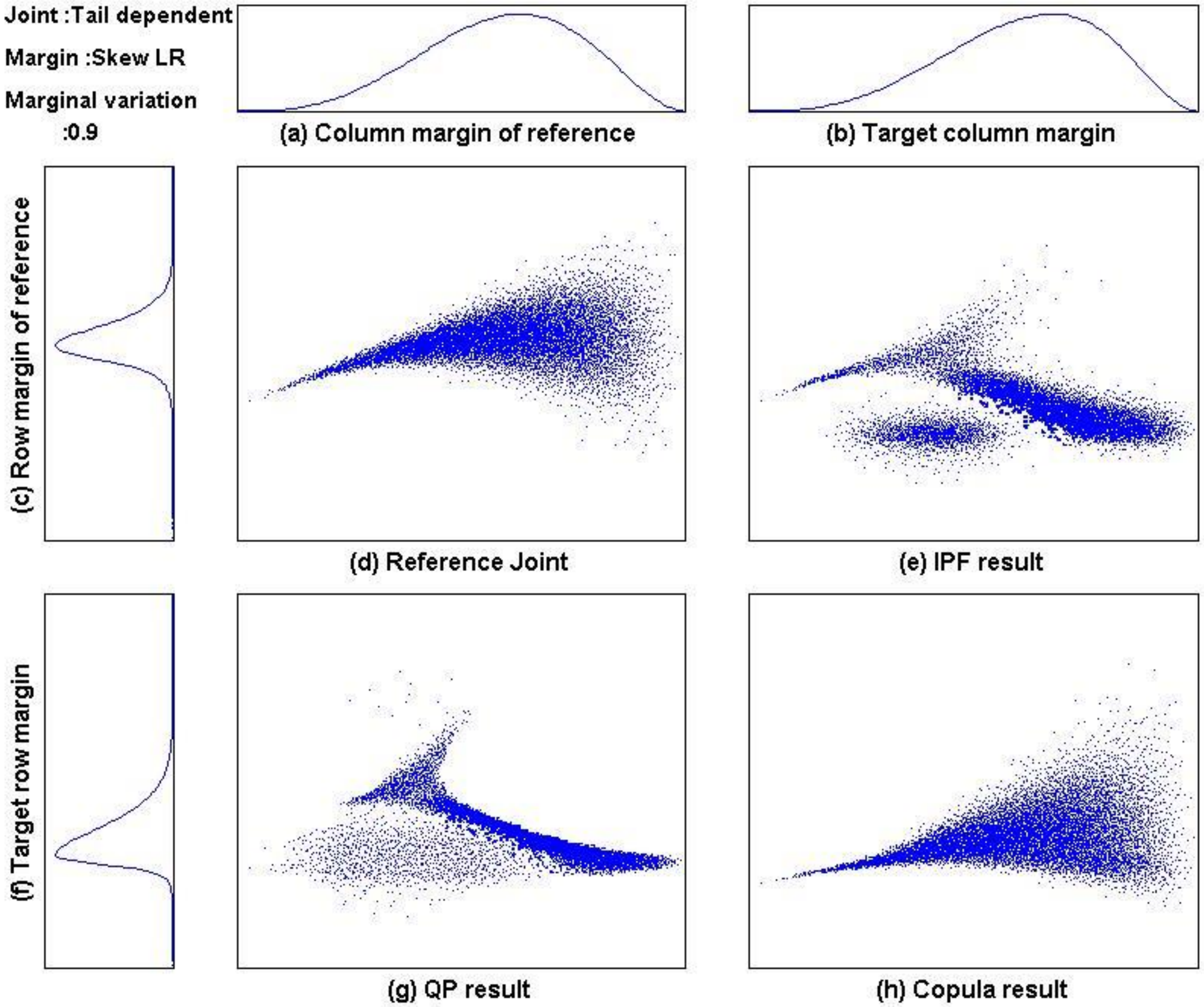

1-3-4. Reference joint distribution : Tail dependent, Target marginal type : Skew RL

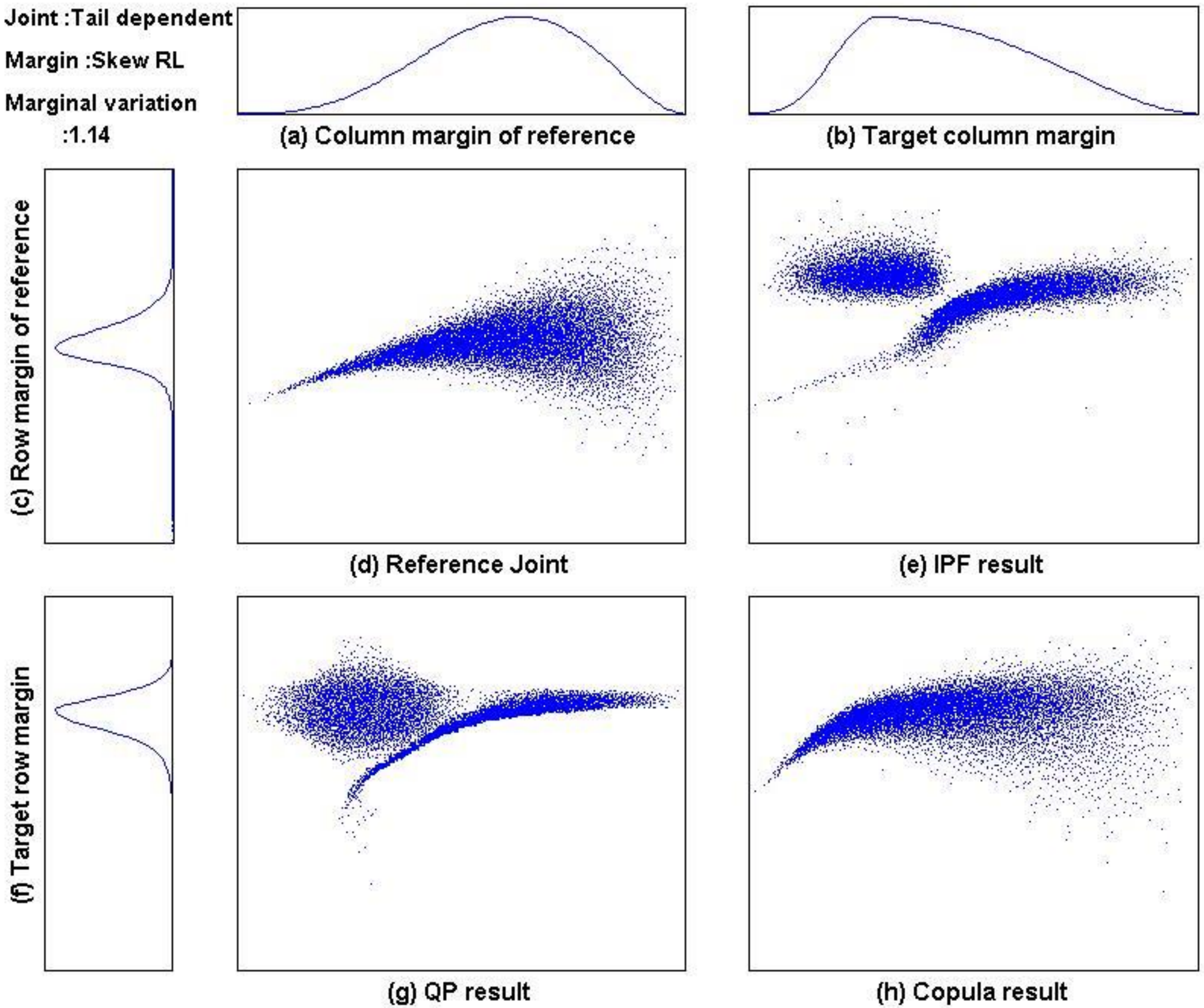

1-3-5. Reference joint distribution : Tail dependent, Target marginal type : Uniform

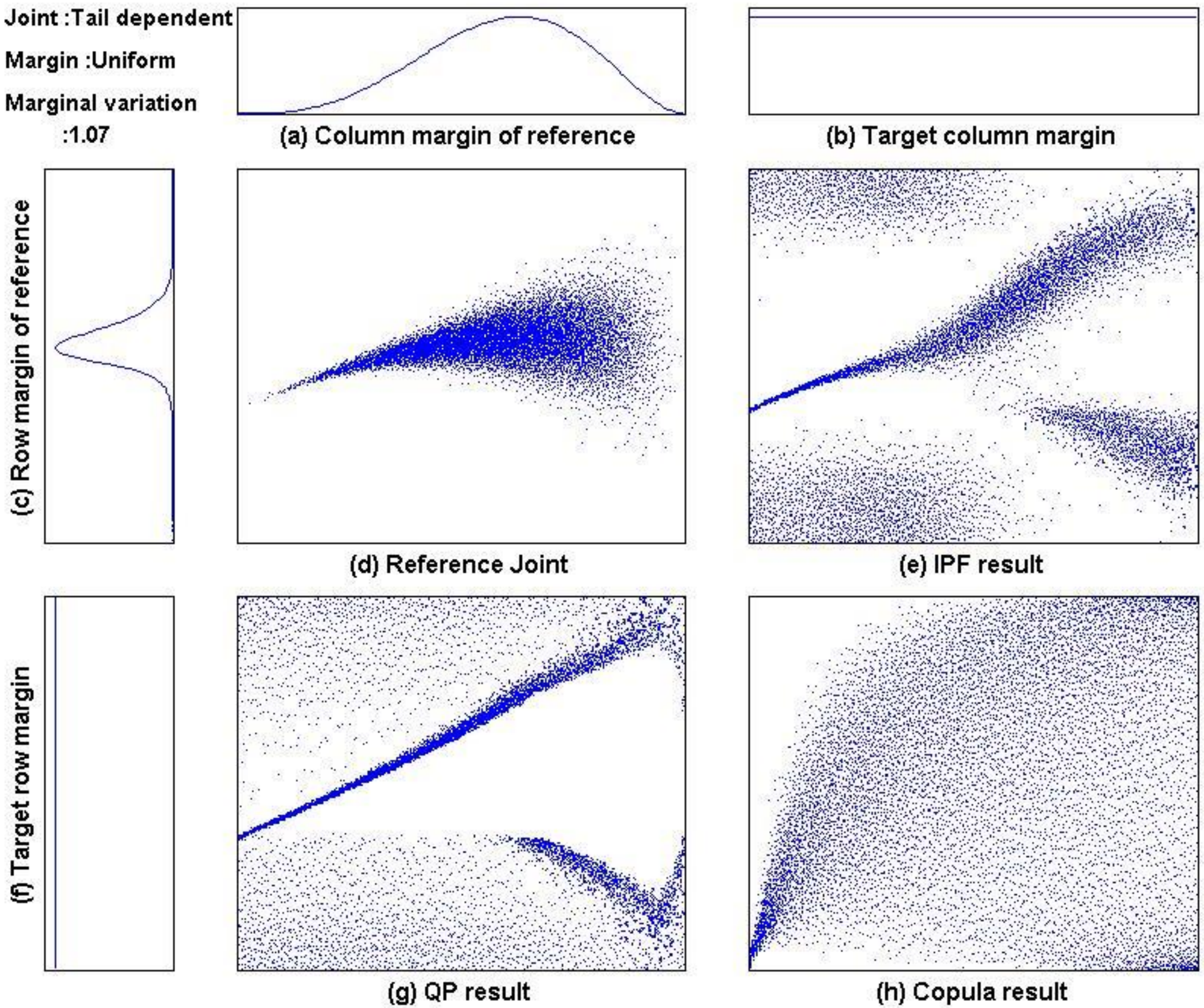

1-3-6. Reference joint distribution : Tail dependent, Target marginal type : Fat tail

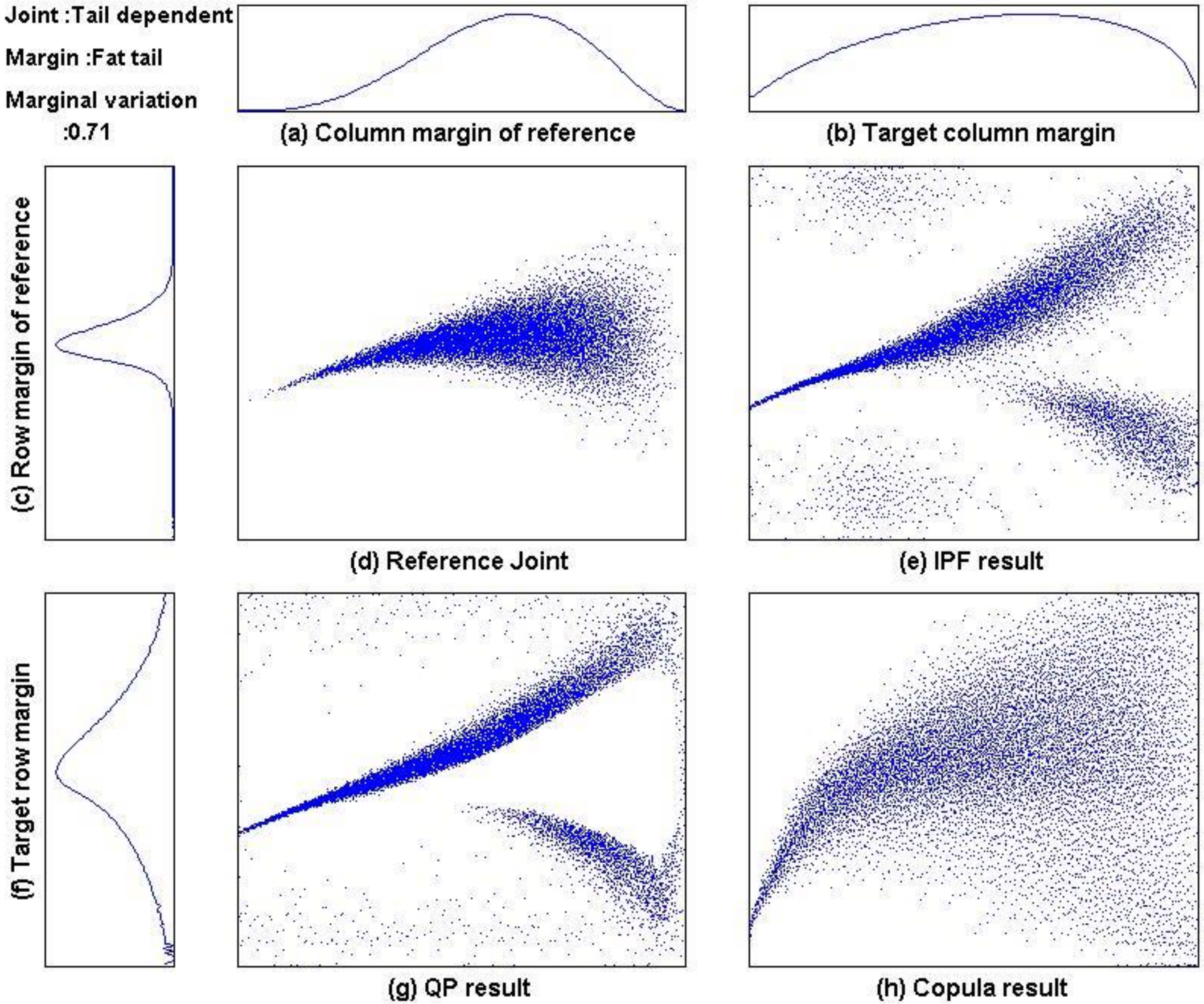

1-3-7. Reference joint distribution : Tail dependent, Target marginal type : Thin tail

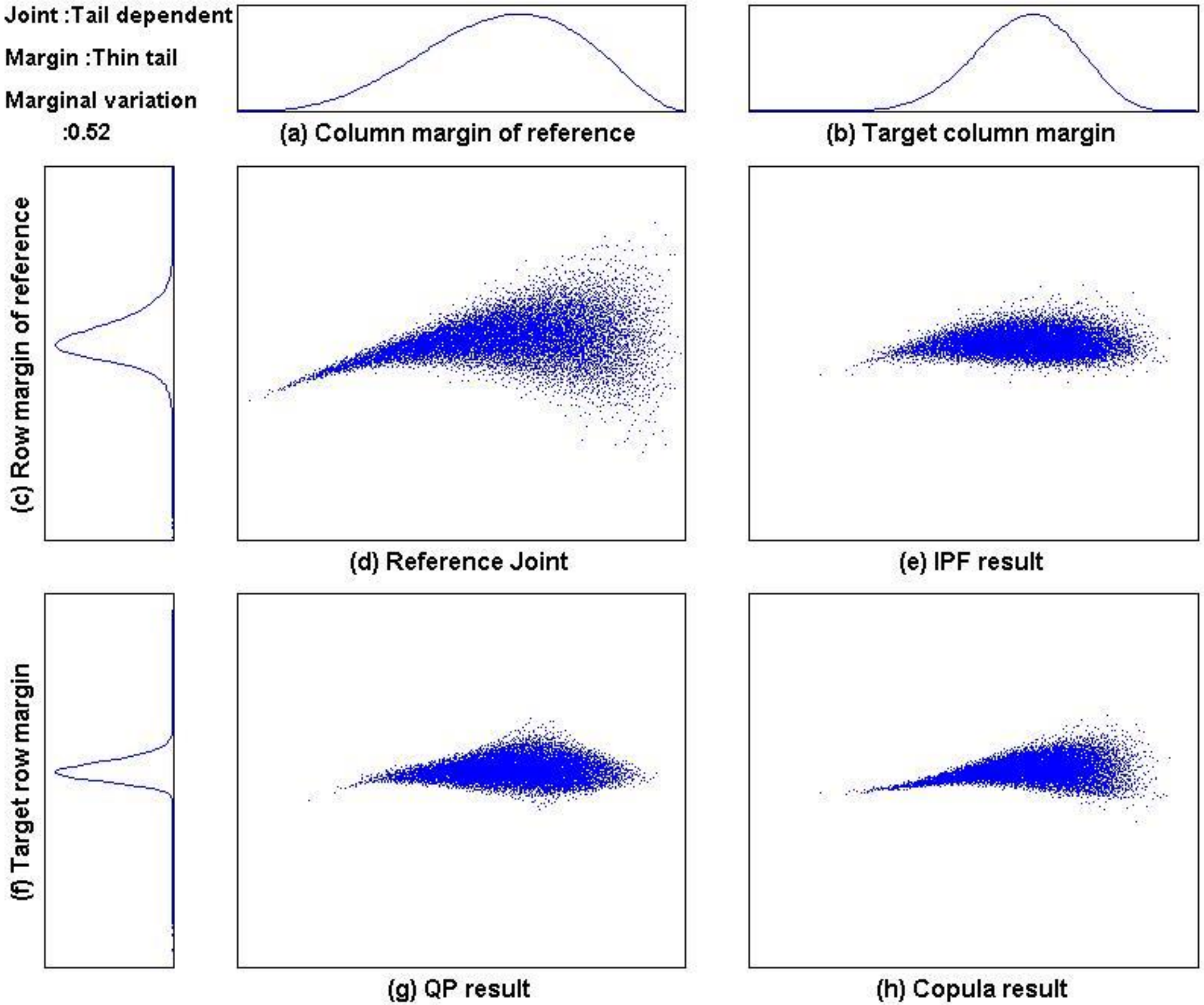

1-3-8. Reference joint distribution : Tail dependent, Target marginal type : Perturbation

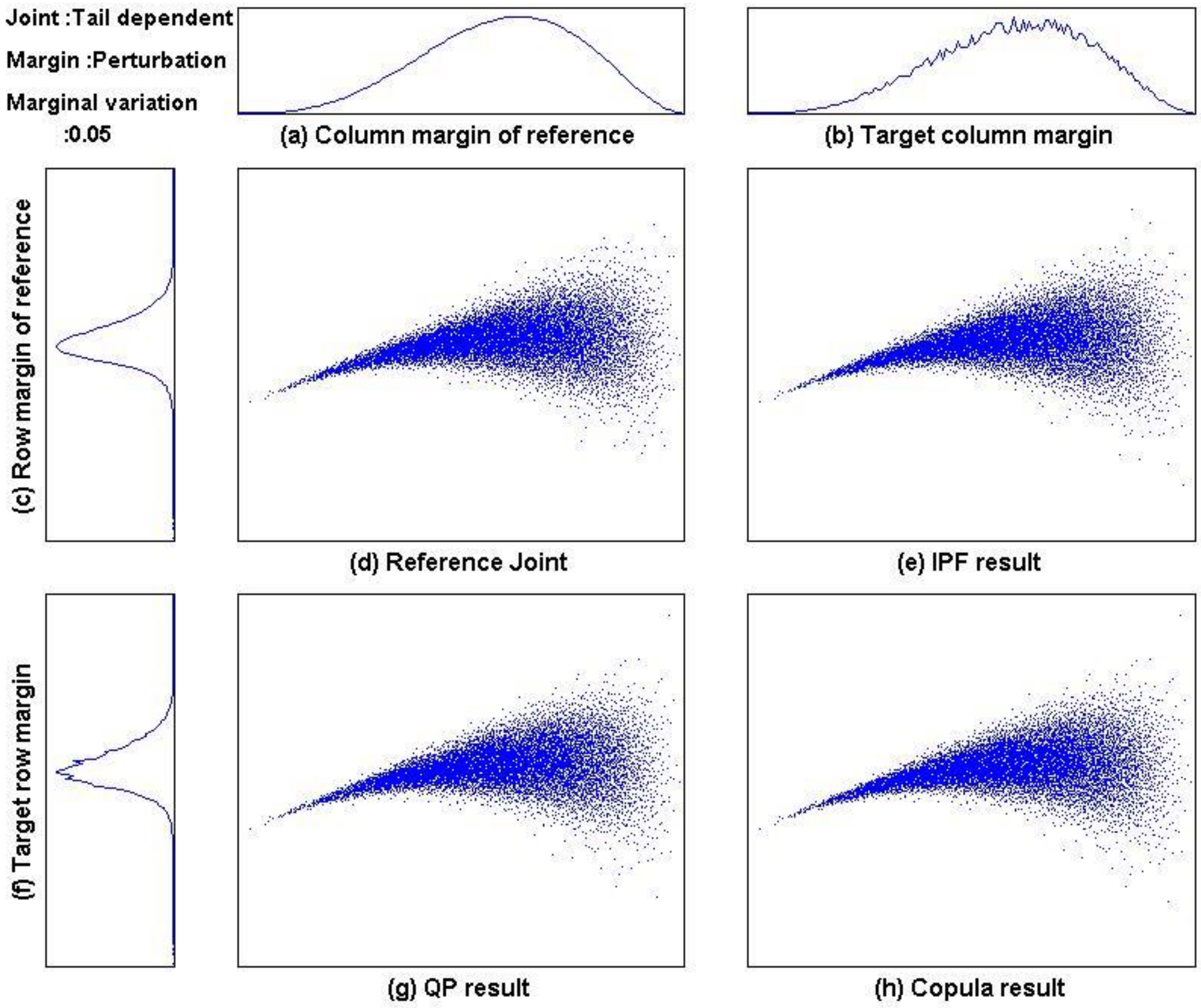

1-4-1. Reference joint distribution : U-shape, Target marginal type : Skew LL

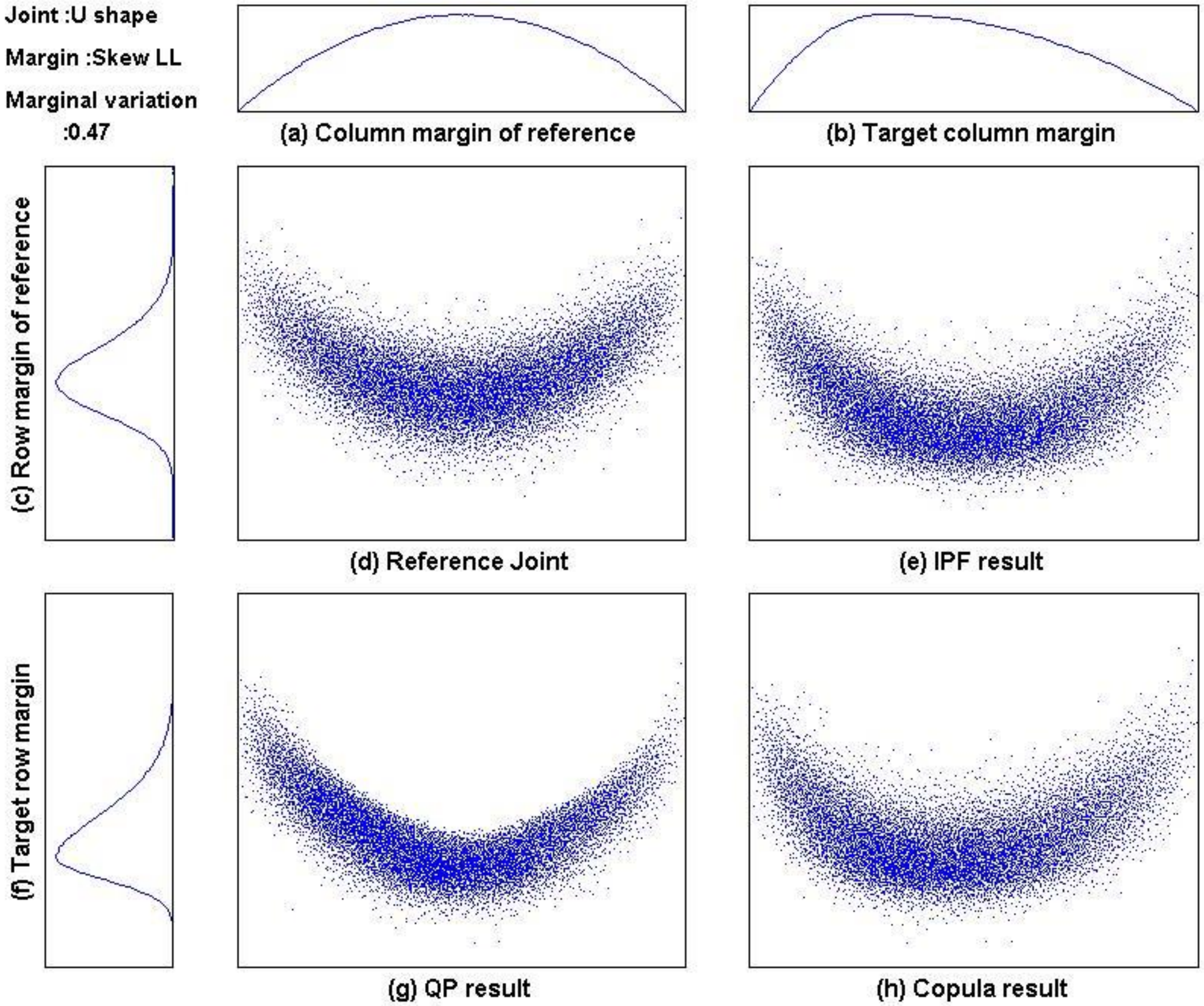

1-4-2. Reference joint distribution : U-shape, Target marginal type : Skew RR

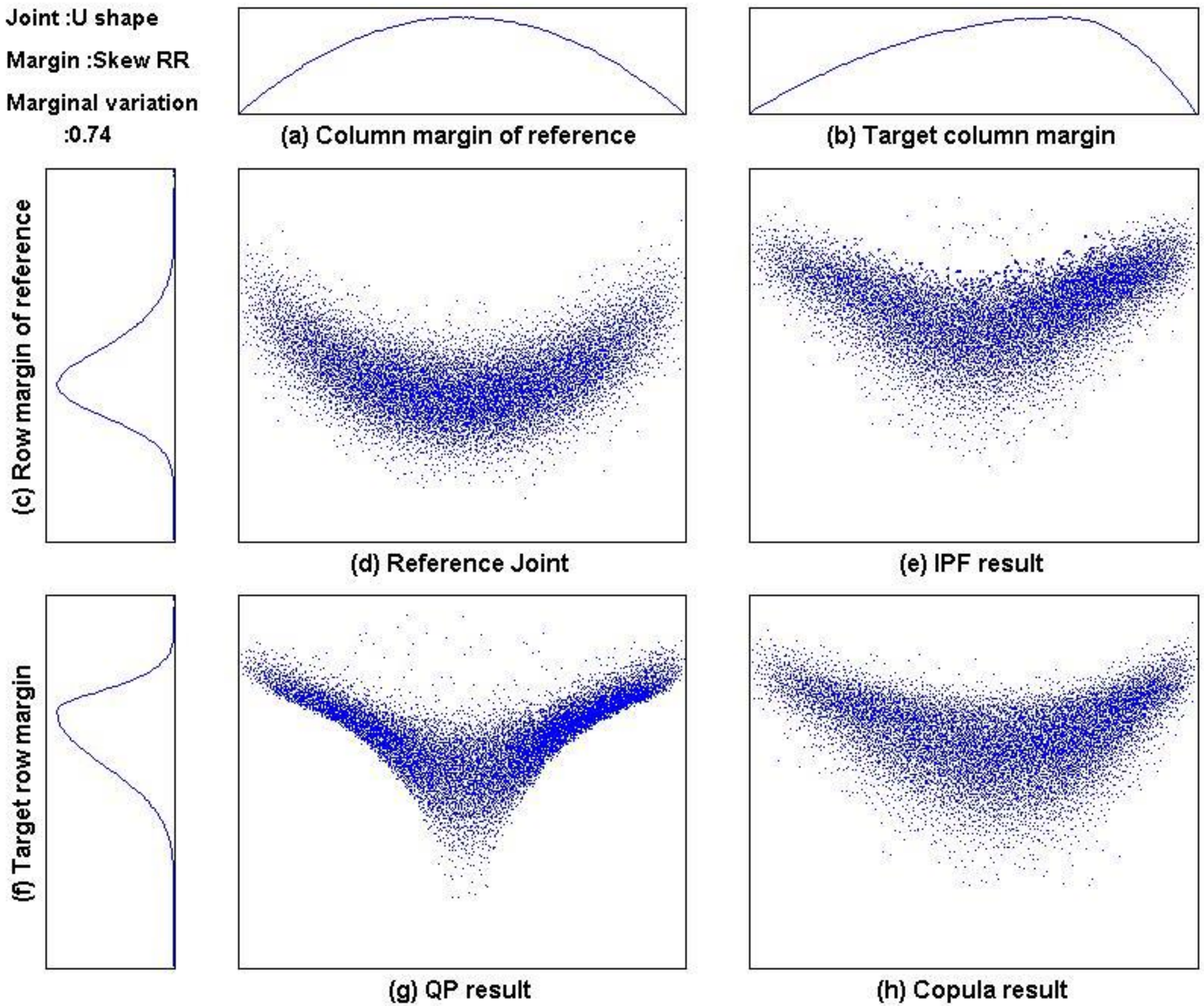

1-4-3. Reference joint distribution : U-shape, Target marginal type : Skew LR

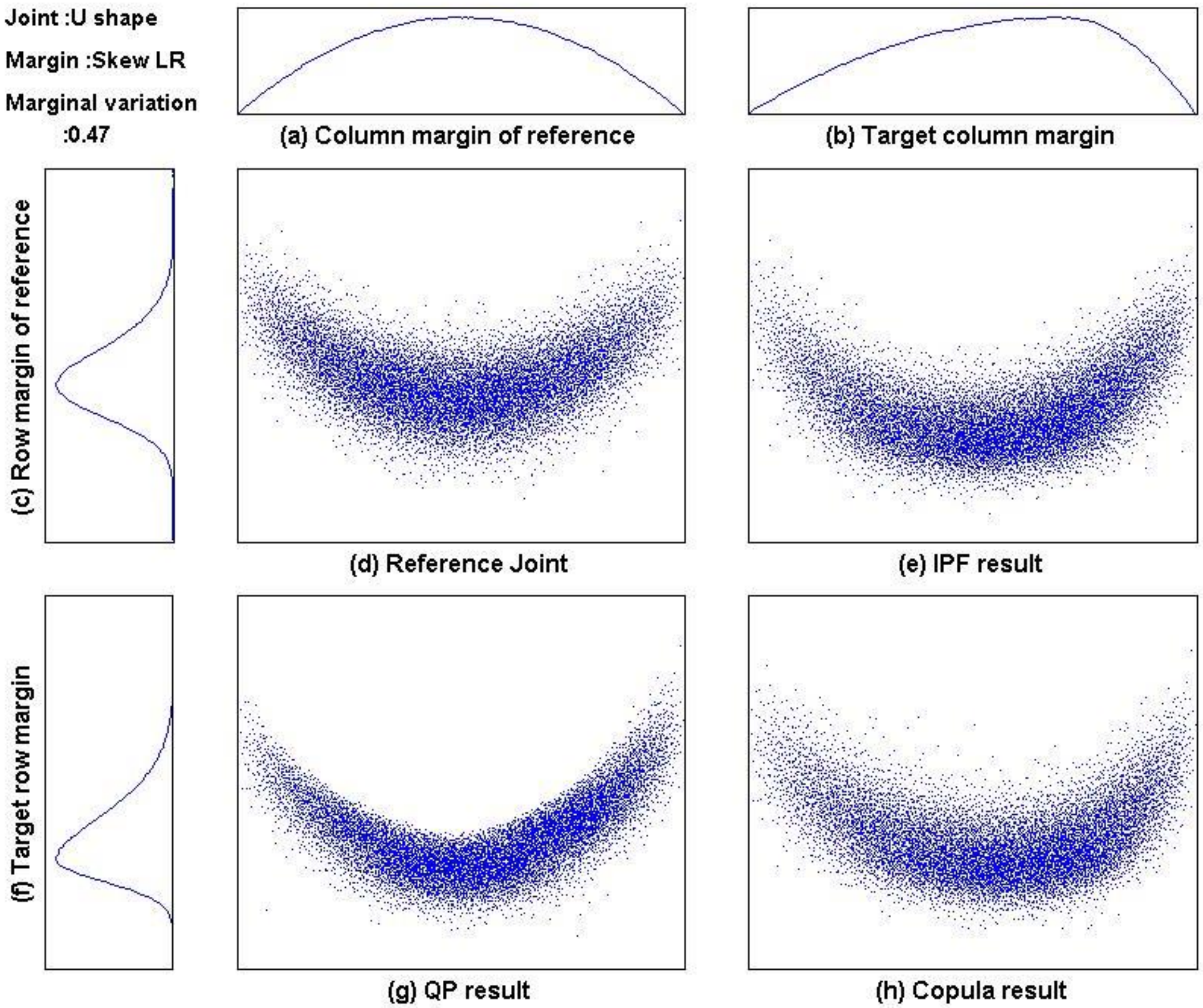

1-4-4. Reference joint distribution : U-shape, Target marginal type : Skew RL

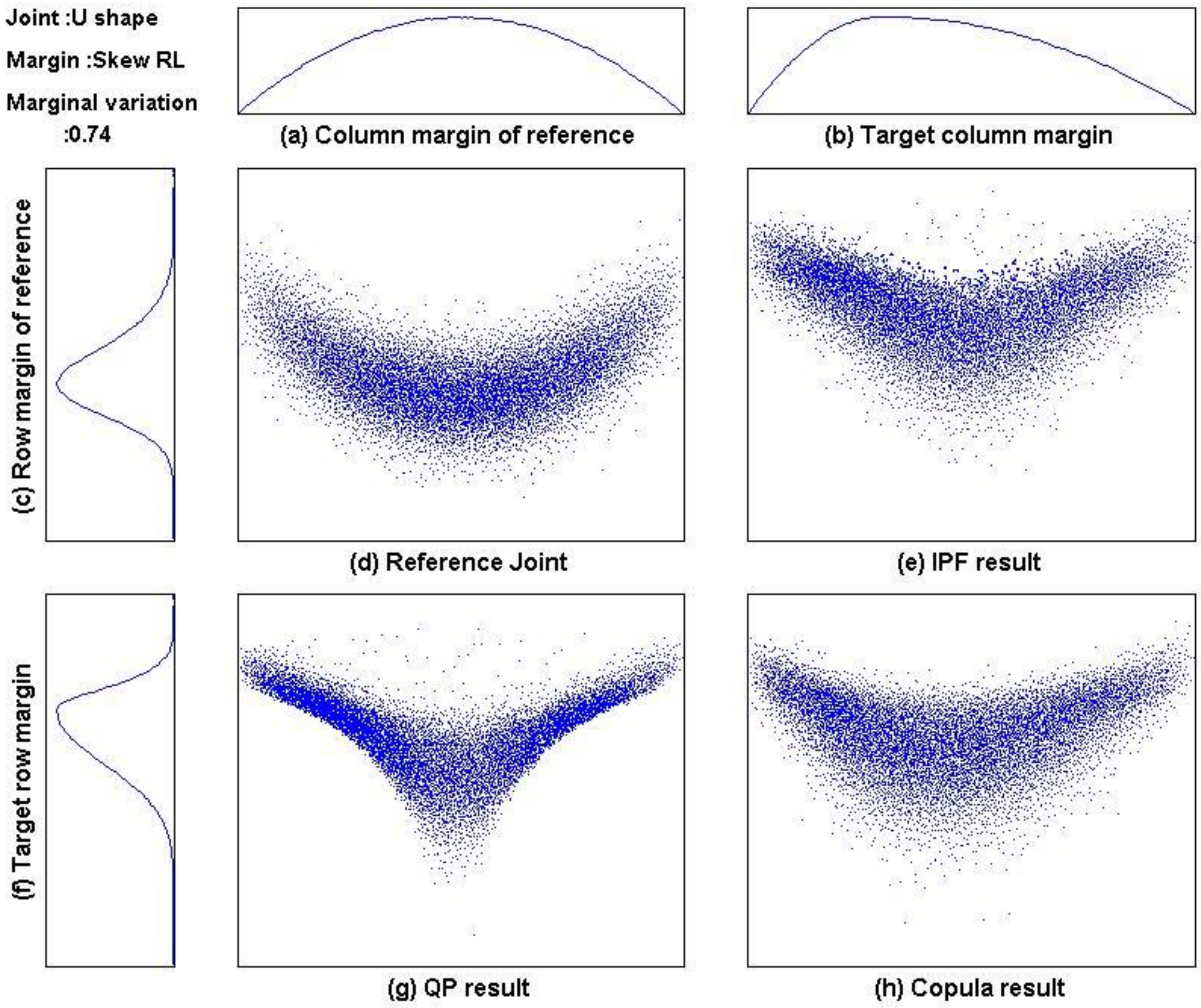

1-4-5. Reference joint distribution : U-shape, Target marginal type : Uniform

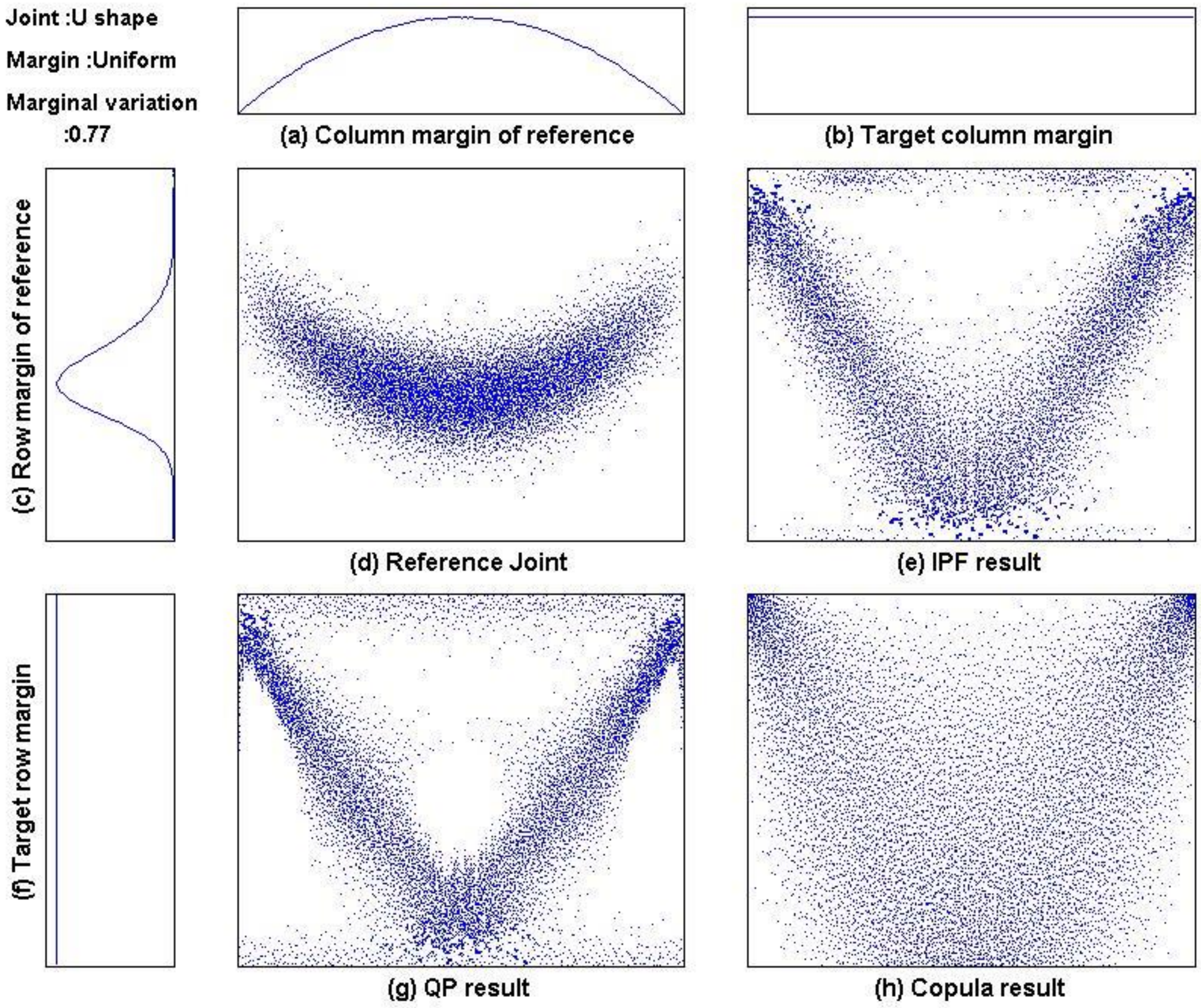

1-4-6. Reference joint distribution : U-shape, Target marginal type : Fat tail

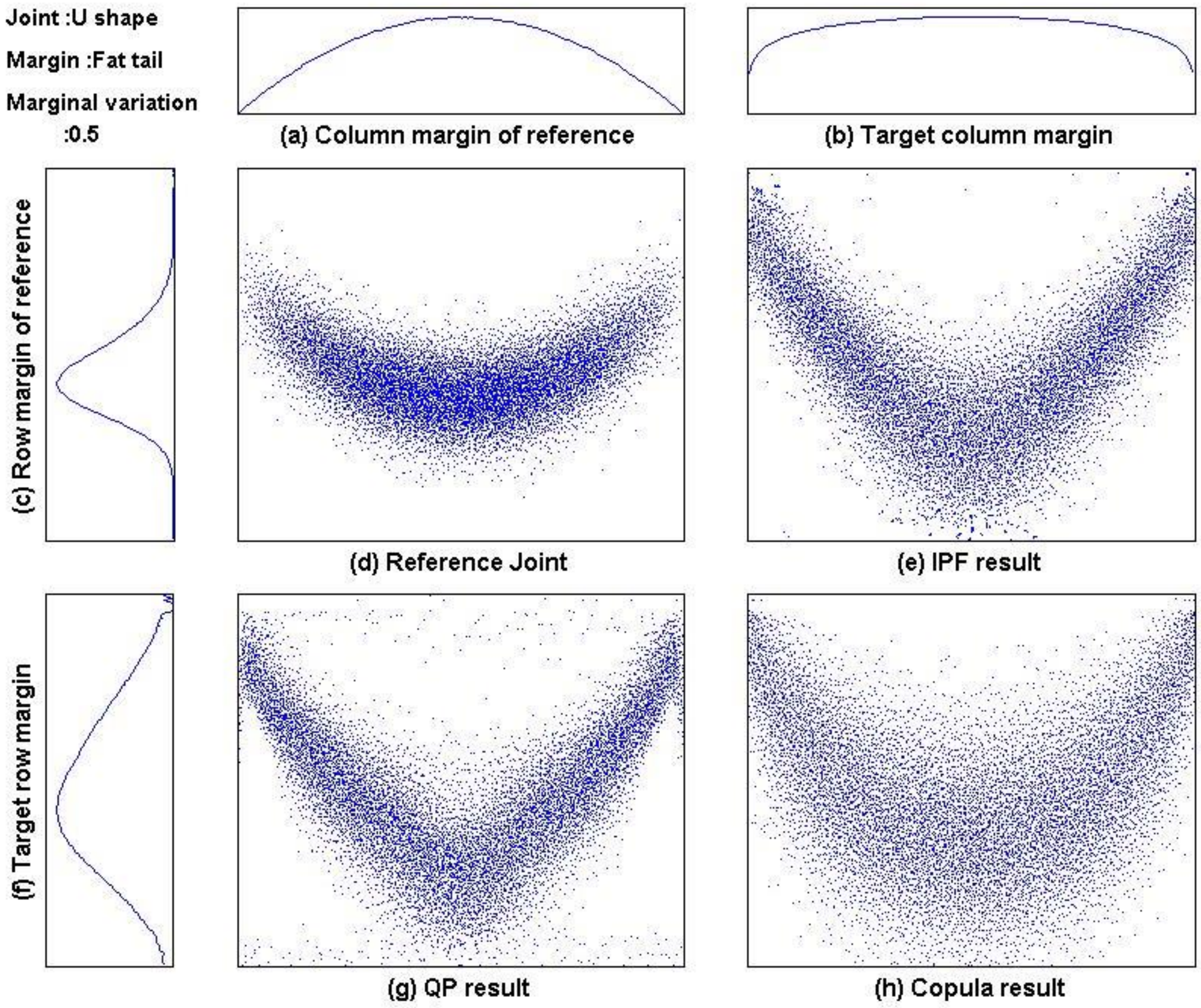

1-4-7. Reference joint distribution : U-shape, Target marginal type : Thin tail

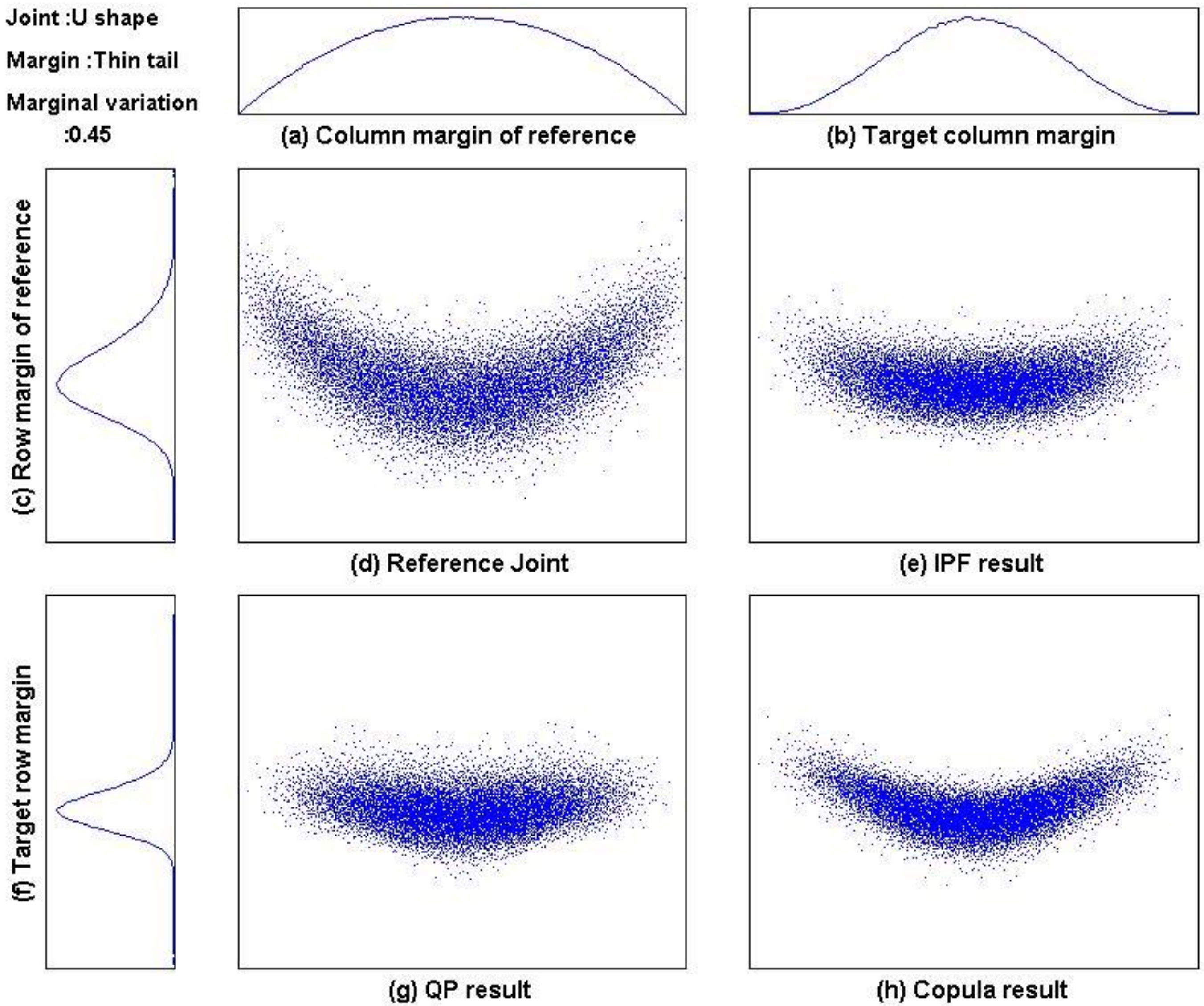

1-4-8. Reference joint distribution : U-shape, Target marginal type : Perturbation

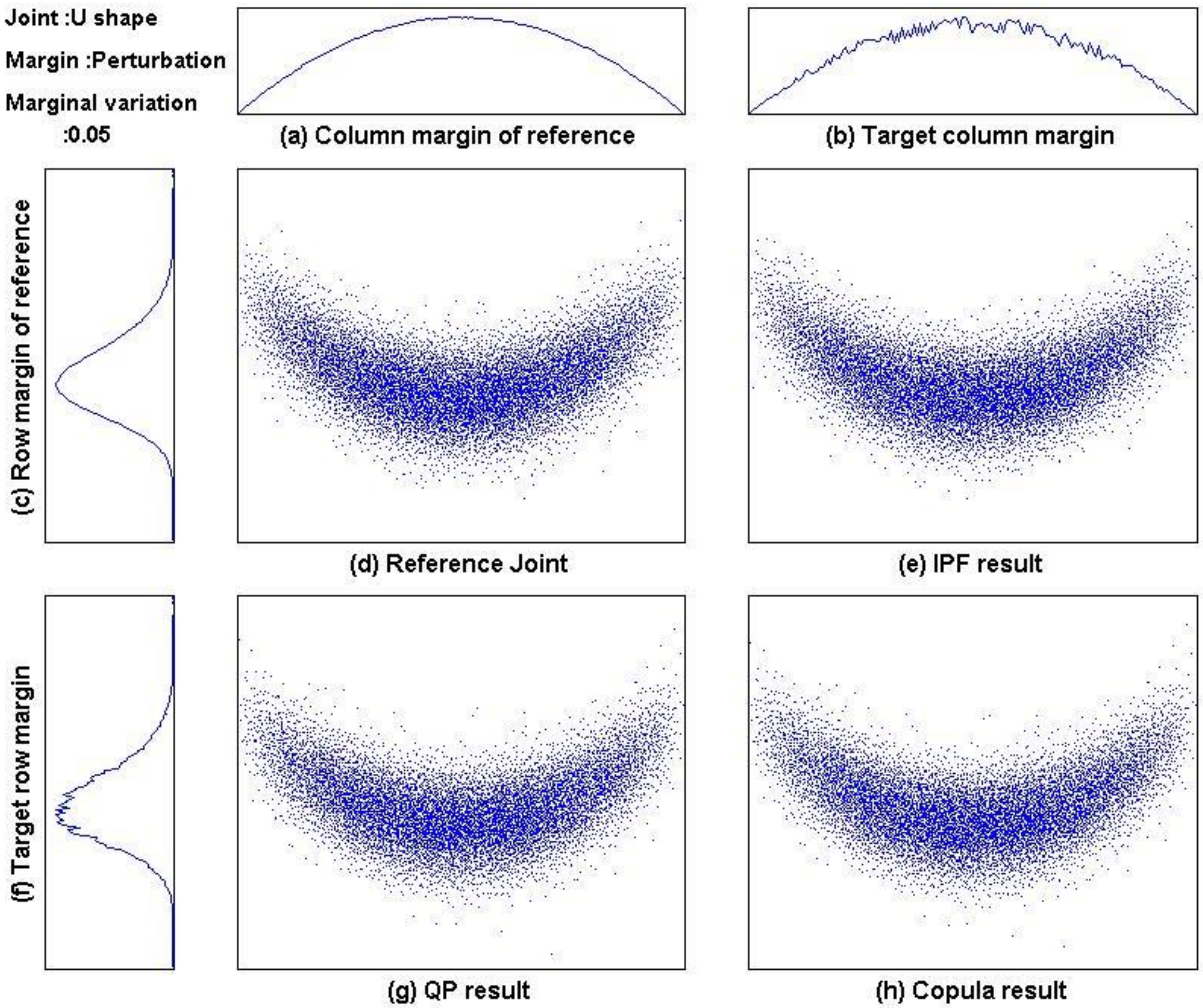

1-5-1. Reference joint distribution : Circle, Target marginal type : Skew LL

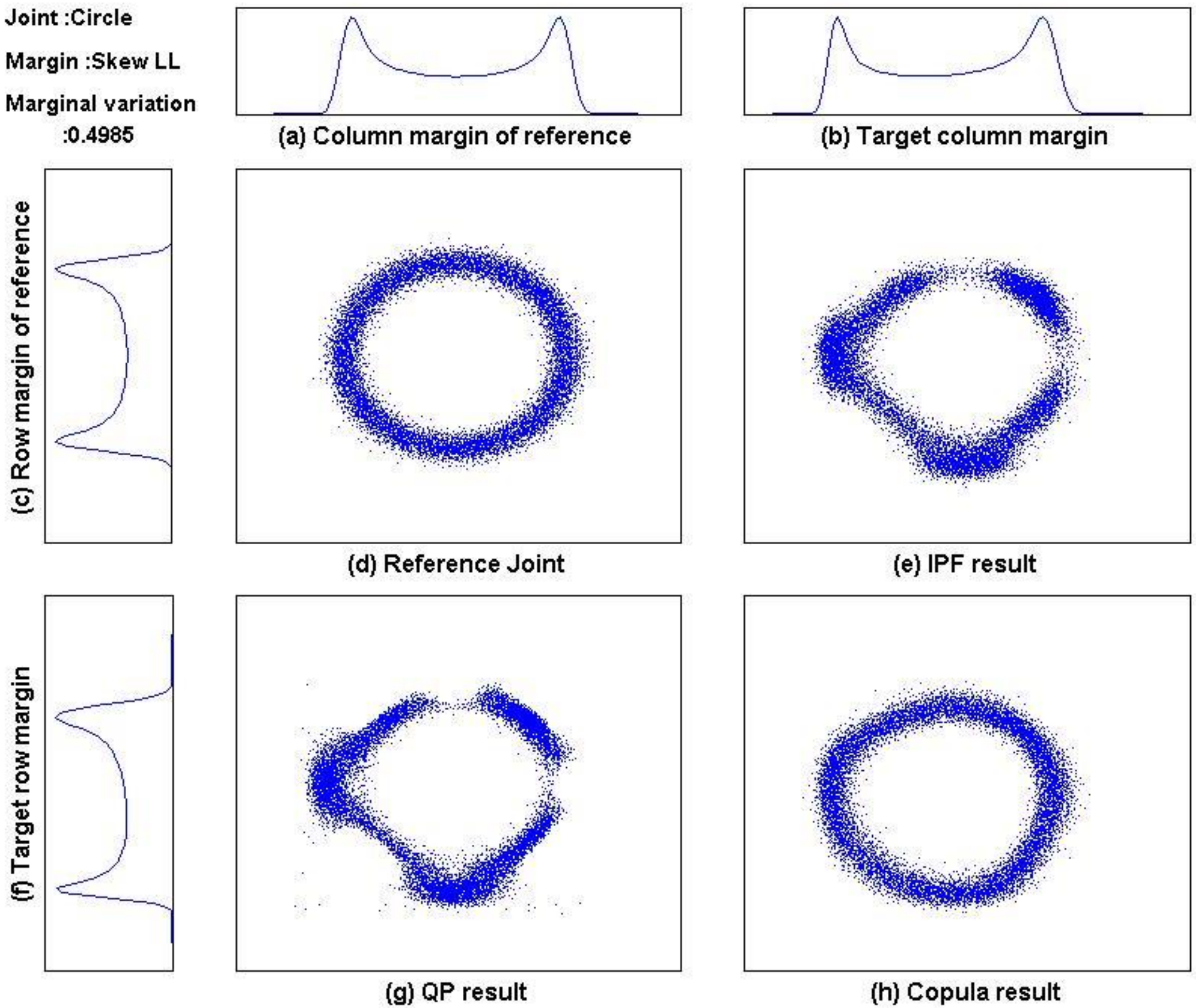

1-5-2. Reference joint distribution : Circle, Target marginal type : Skew RR

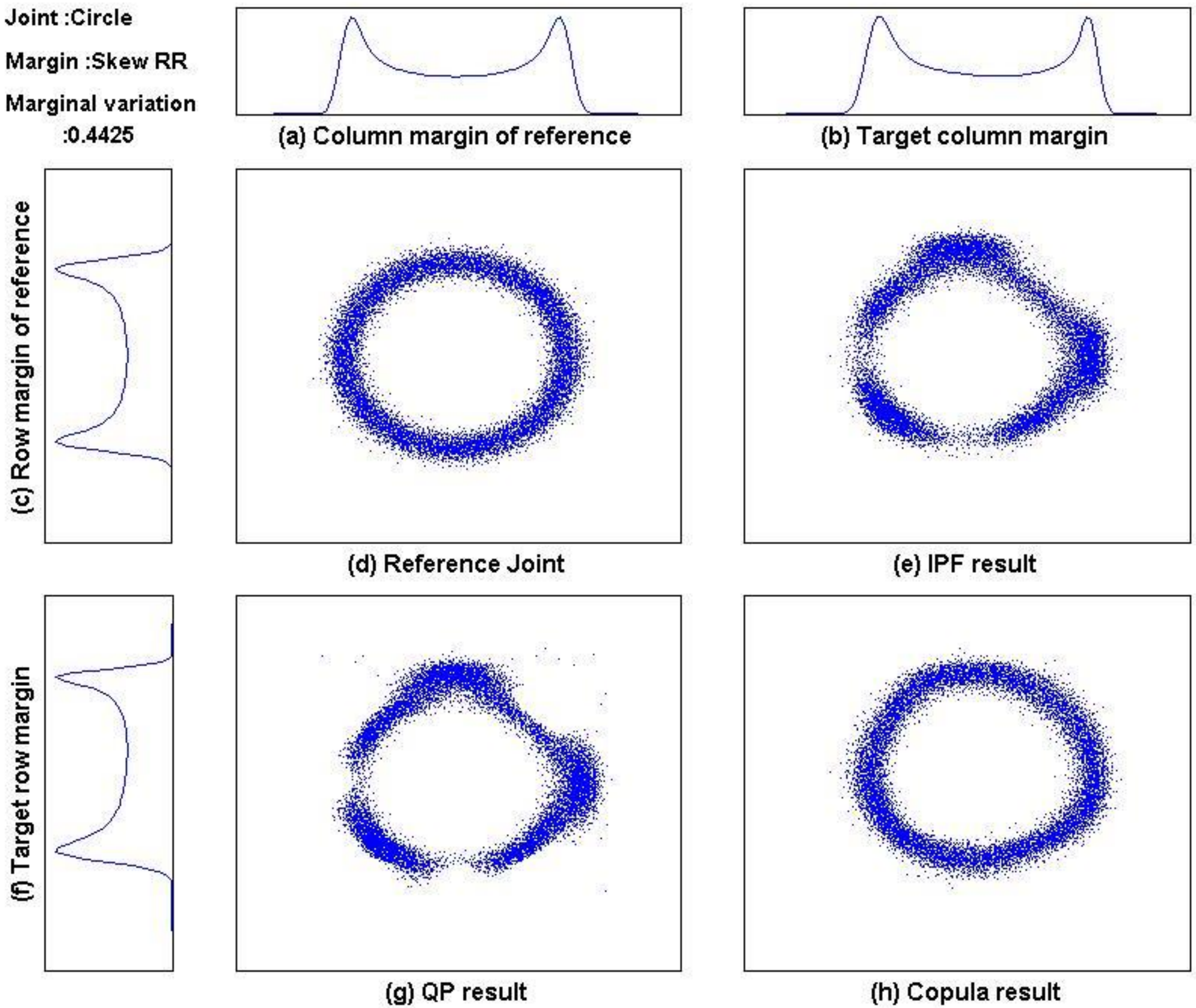

1-5-3. Reference joint distribution : Circle, Target marginal type : Skew LR

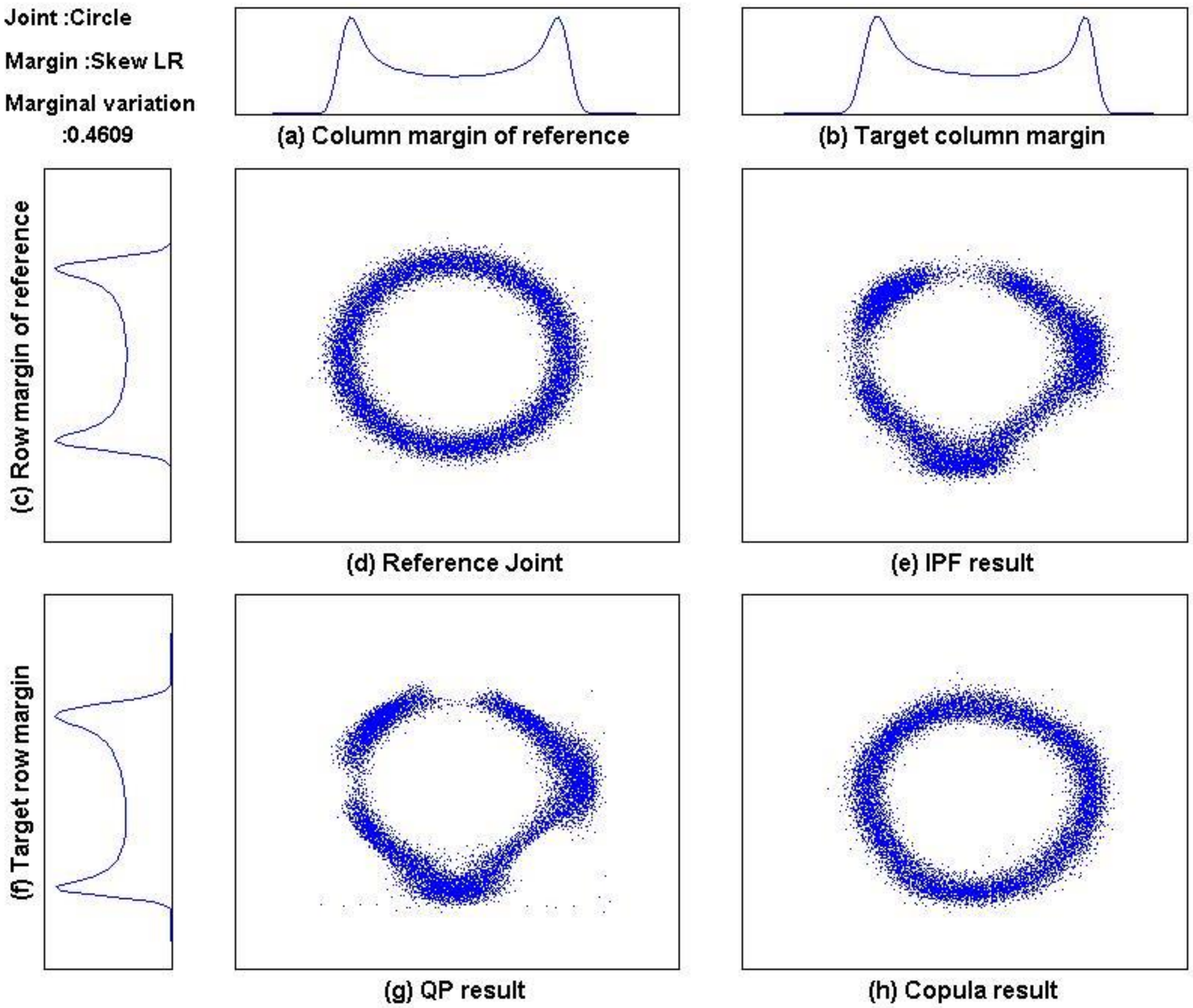

1-5-4. Reference joint distribution : Circle, Target marginal type : Skew RL

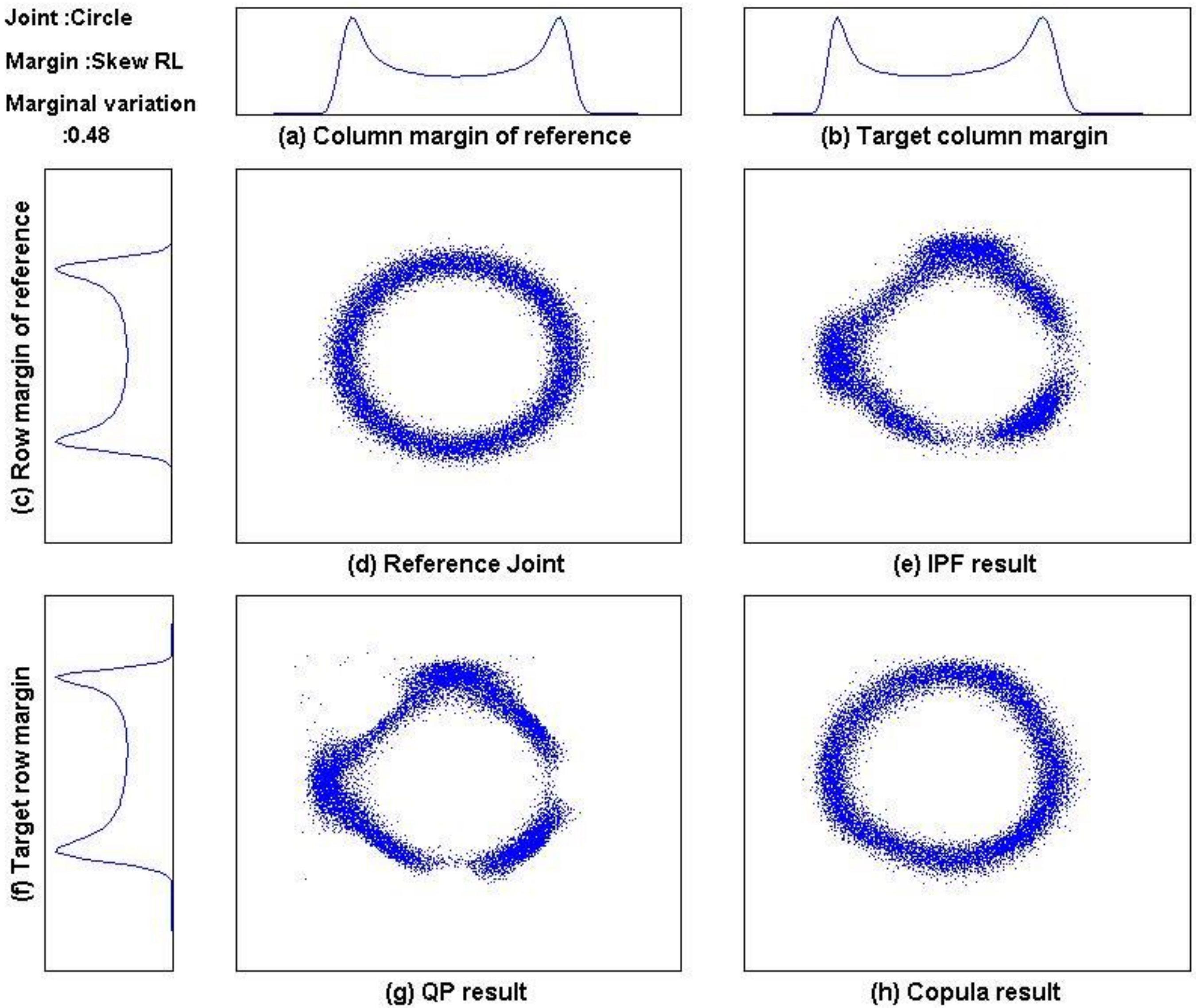

1-5-5. Reference joint distribution : Circle, Target marginal type : Uniform

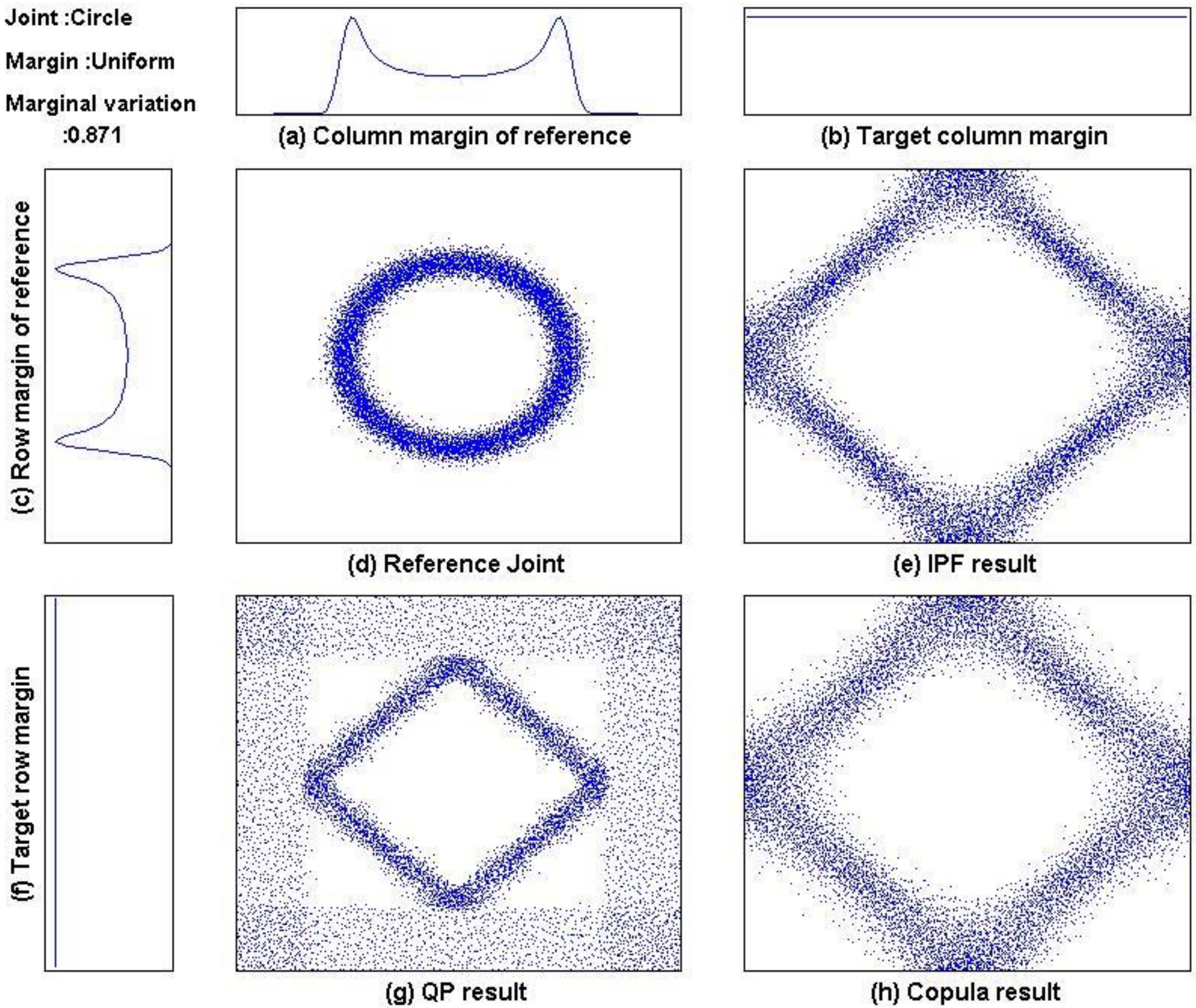

1-5-6. Reference joint distribution : Circle, Target marginal type : Fat tail

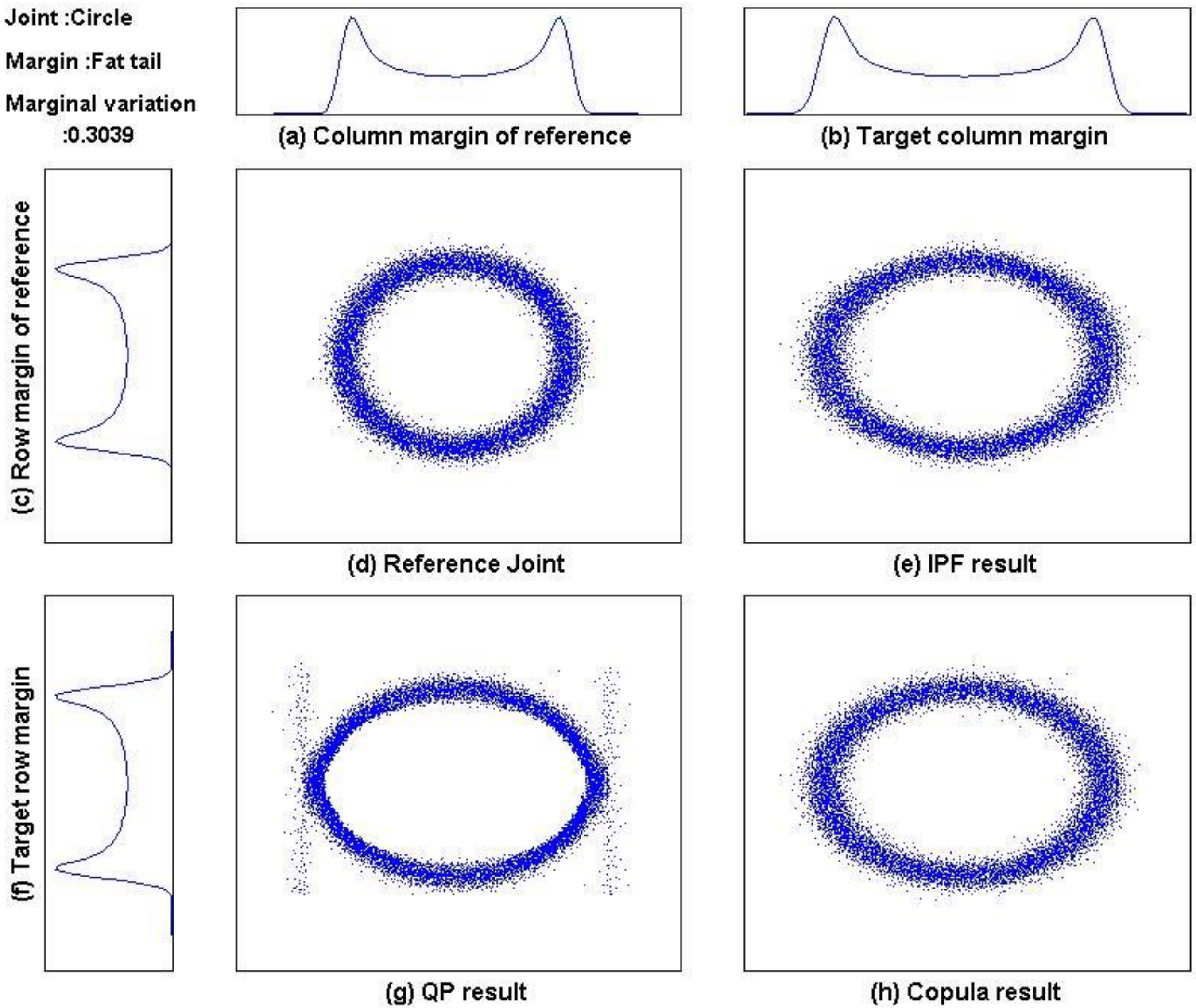

1-5-7. Reference joint distribution : Circle, Target marginal type : Thin tail

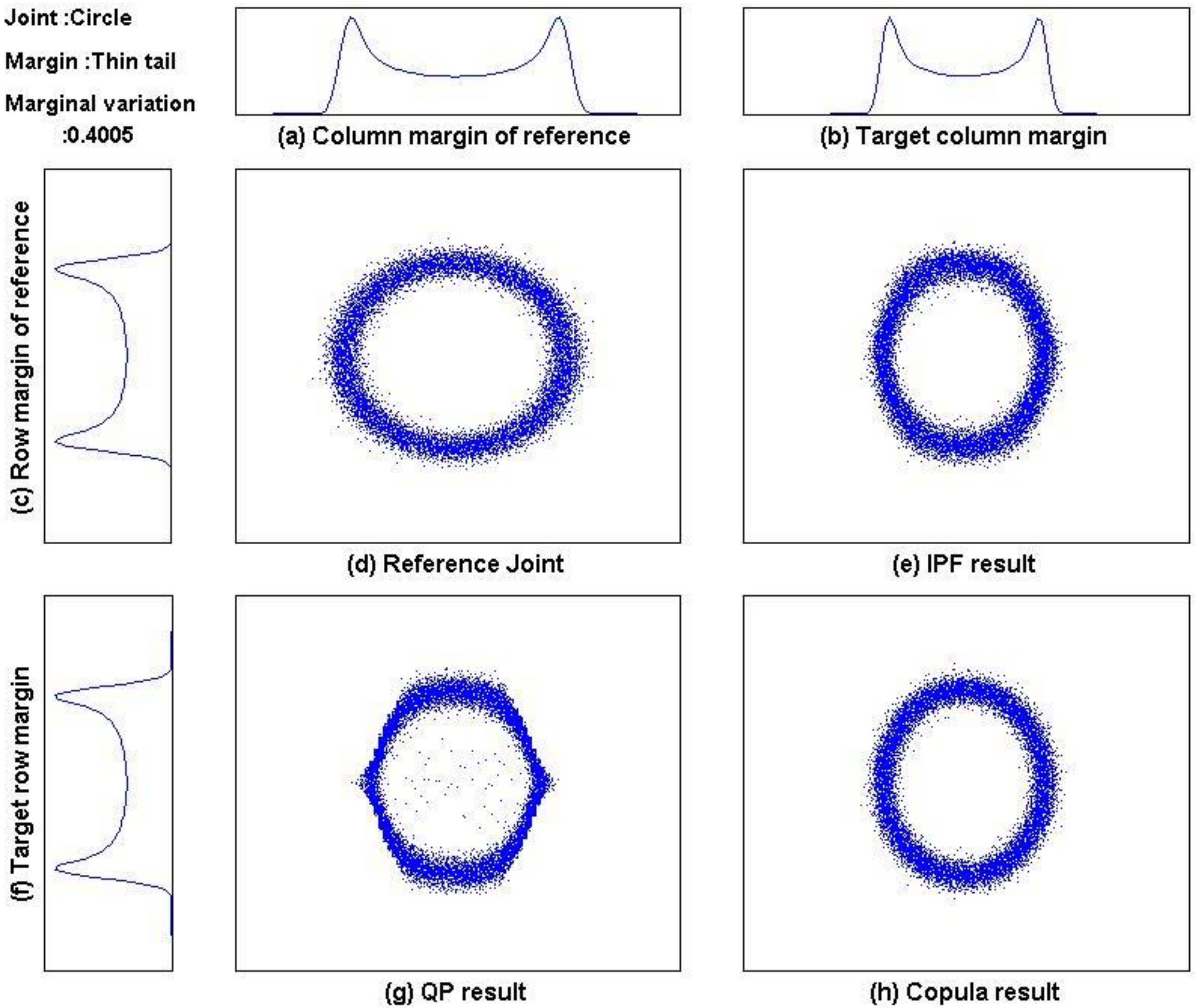

1-5-8. Reference joint distribution : Circle, Target marginal type : Perturbation

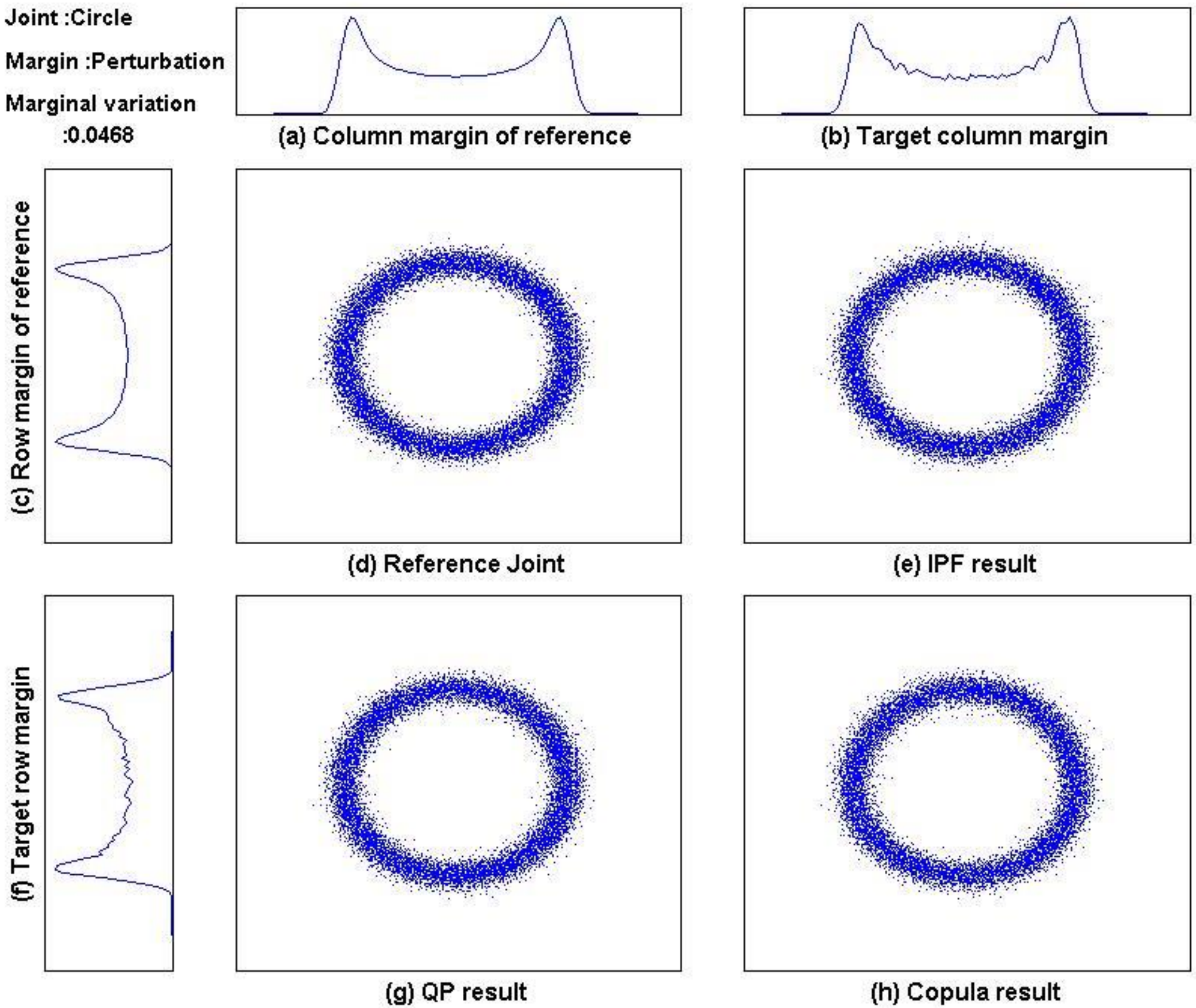

Supplement: S1 Fig — (PDF) [file pone.0159496.s001.pdf]
