# Supplementary material for: Copula-Based Approach to Synthetic Population Generation
Source: PLoS One. 2016 Aug 4;11(8):e0159496. doi: 10.1371/journal.pone.0159496 (PMC4973930; doi:10.1371/journal.pone.0159496)

S2 Fig. Experiments on various changes on marginal distributions

2-1-1. Reference joint distribution : Bivariate normal, Target marginal type : Skew, Marginal variation : 0.32

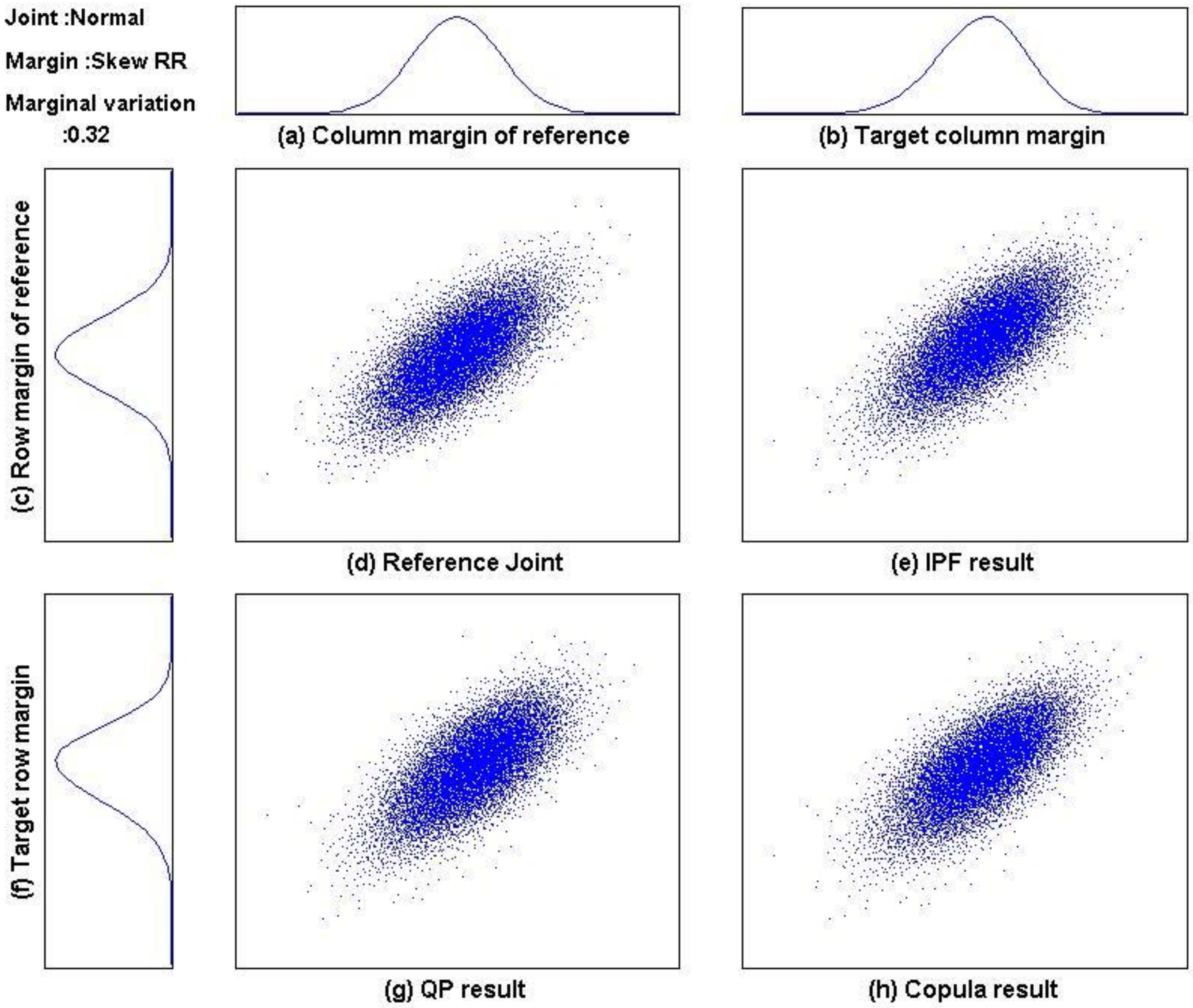

2-1-2. Reference joint distribution : Bivariate normal, Target marginal type : Skew, Marginal variation : 0.65

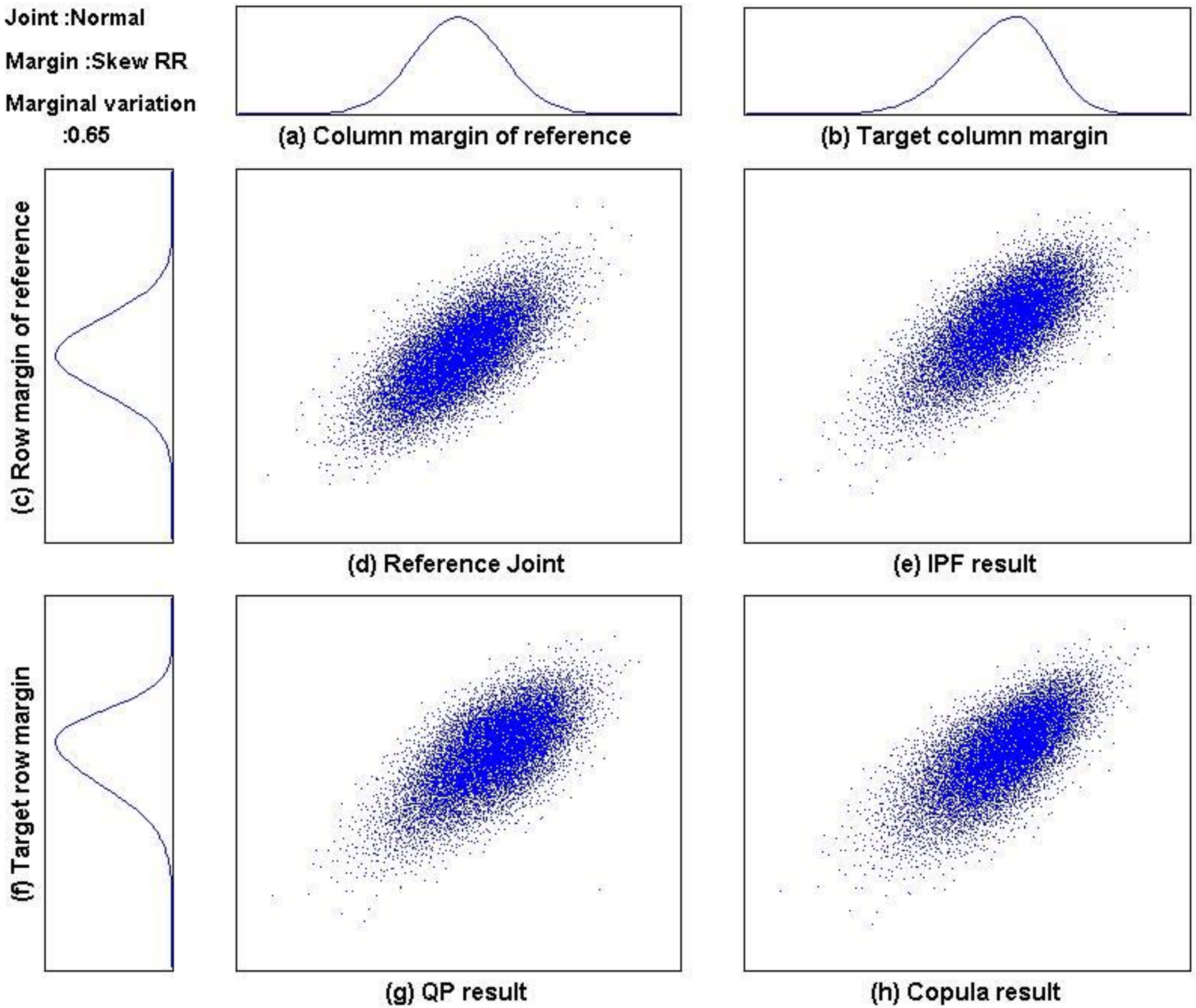

2-1-3. Reference joint distribution : Bivariate normal, Target marginal type : Skew, Marginal variation : 0.97

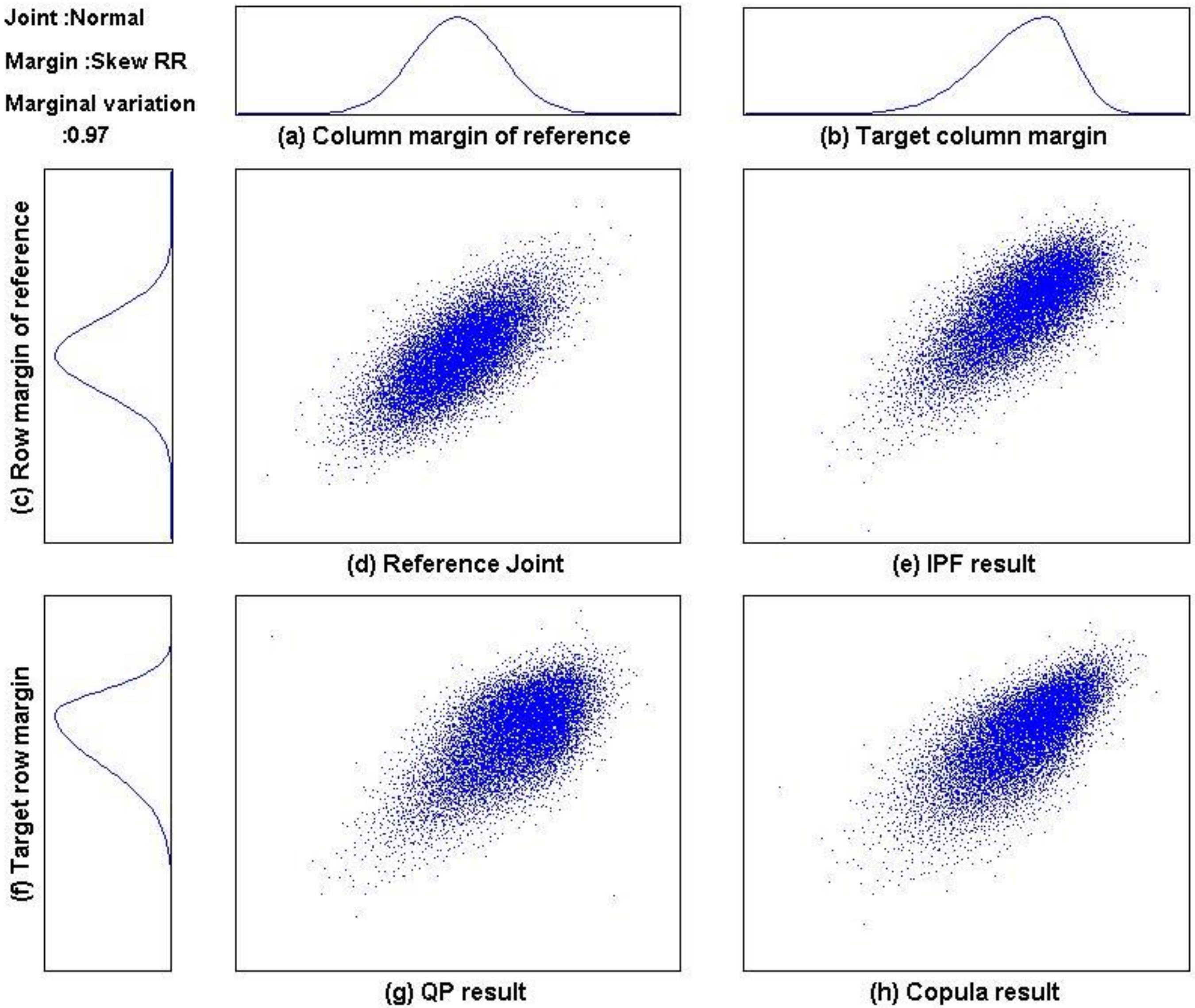

2-1-4. Reference joint distribution : Bivariate normal, Target marginal type : Skew, Marginal variation : 1.28

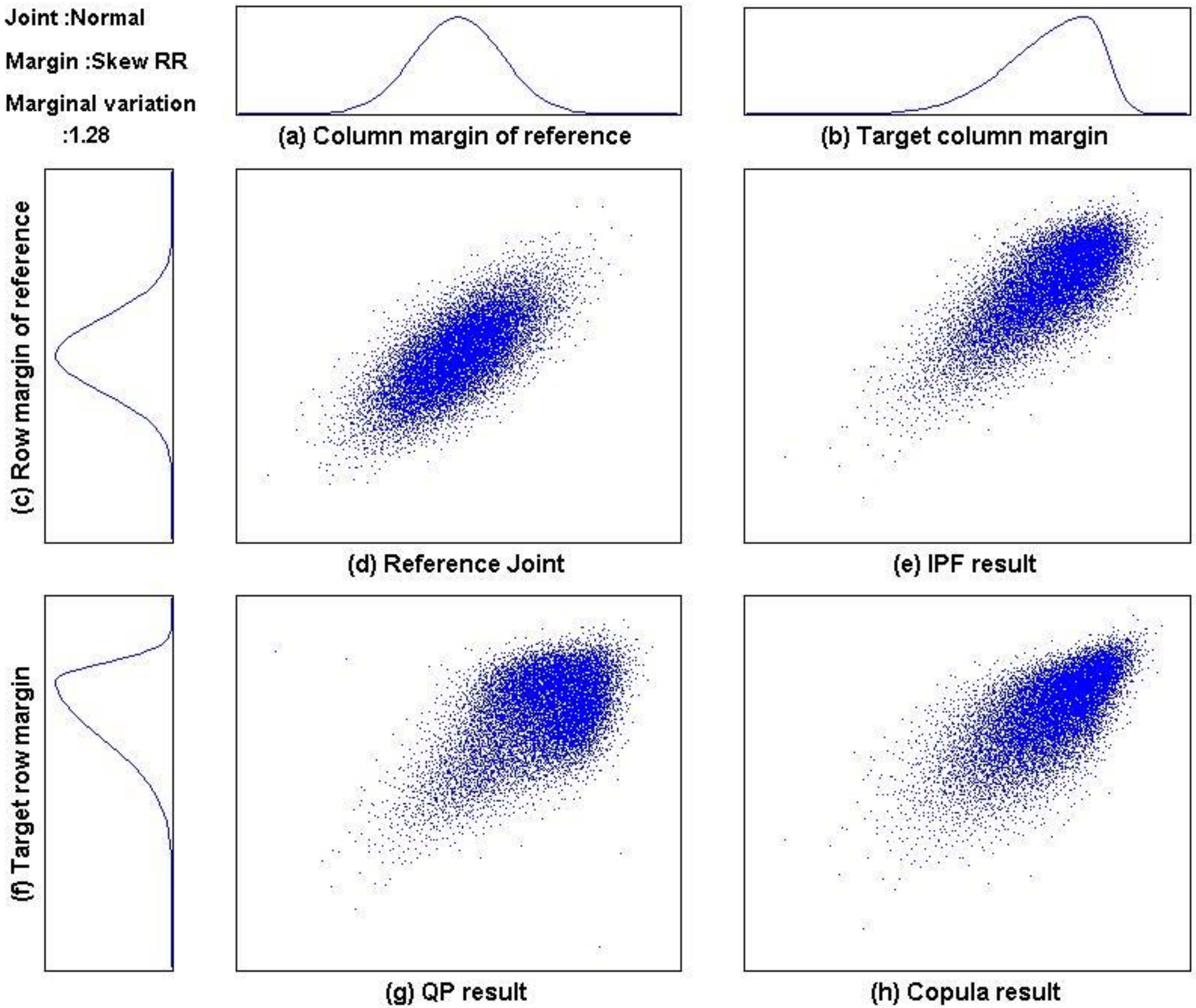

2-1-5. Reference joint distribution : Bivariate normal, Target marginal type : Skew, Marginal variation : 1.61

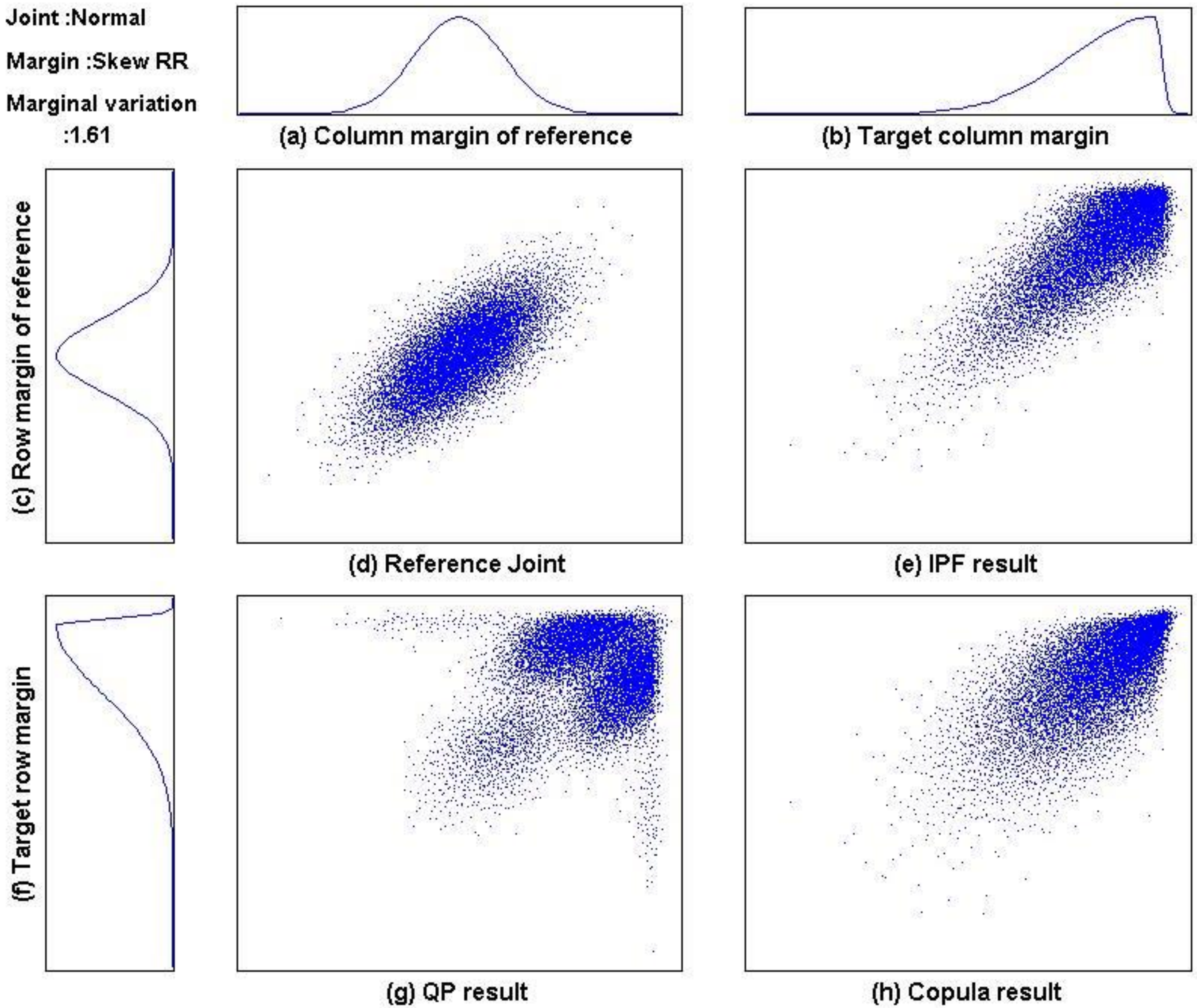

2-2-1. Reference joint distribution : Bivariate normal, Target marginal type : Fat tail, Marginal variation : 0.2

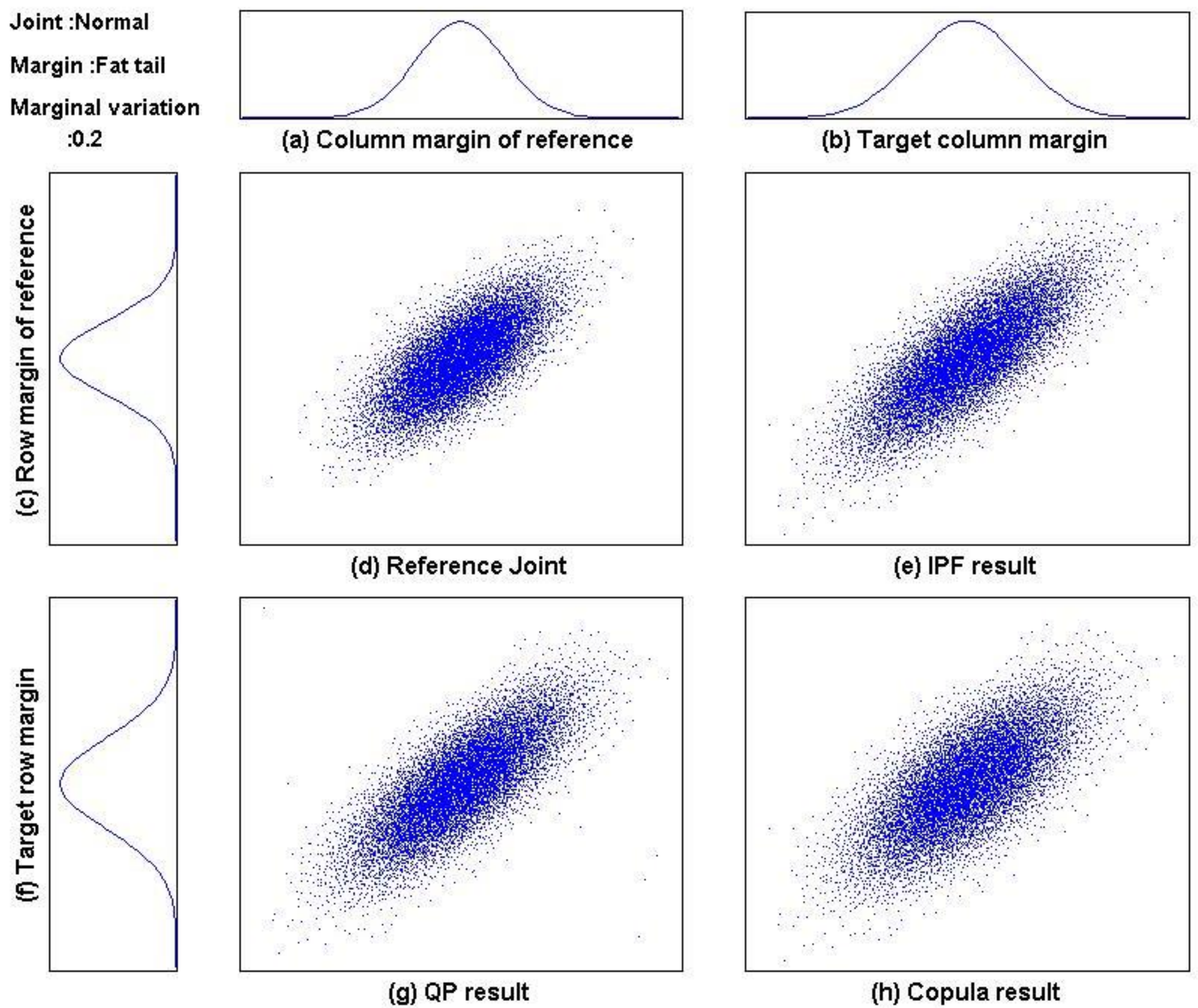

2-2-2. Reference joint distribution : Bivariate normal, Target marginal type : Fat tail, Marginal variation : 0.4

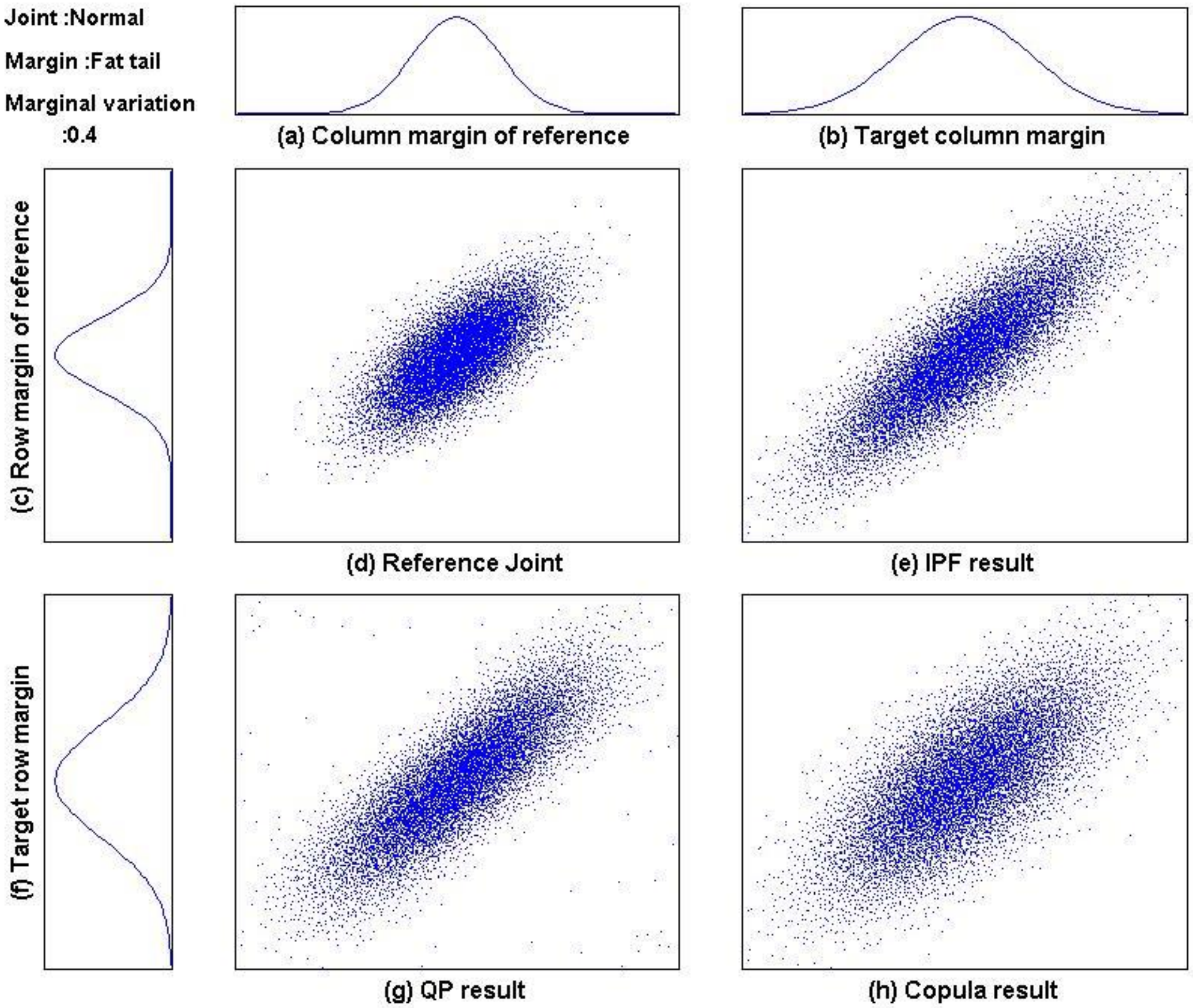

2-2-3. Reference joint distribution : Bivariate normal, Target marginal type : Fat tail, Marginal variation : 0.6

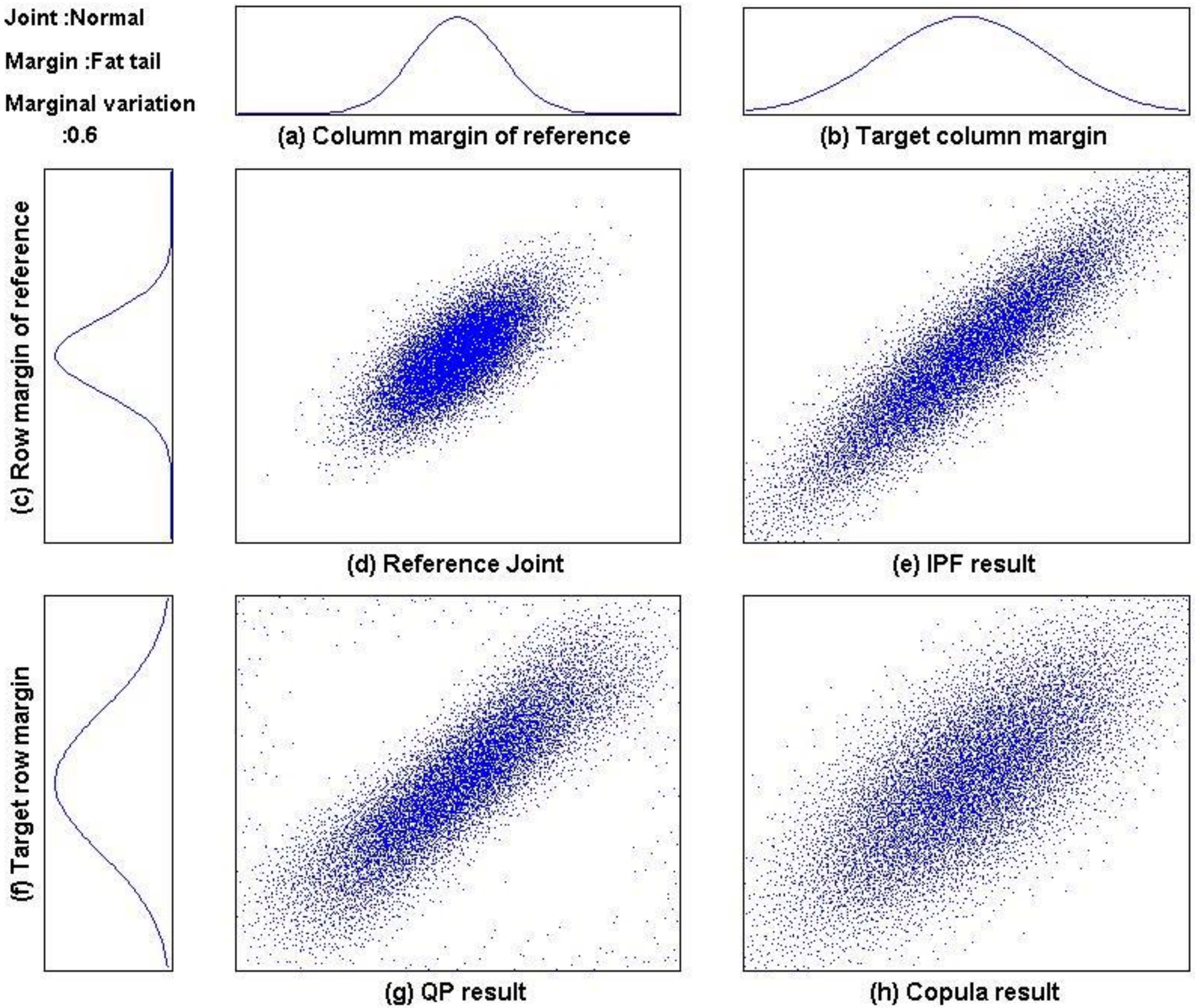

2-2-4. Reference joint distribution : Bivariate normal, Target marginal type : Fat tail, Marginal variation : 0.8

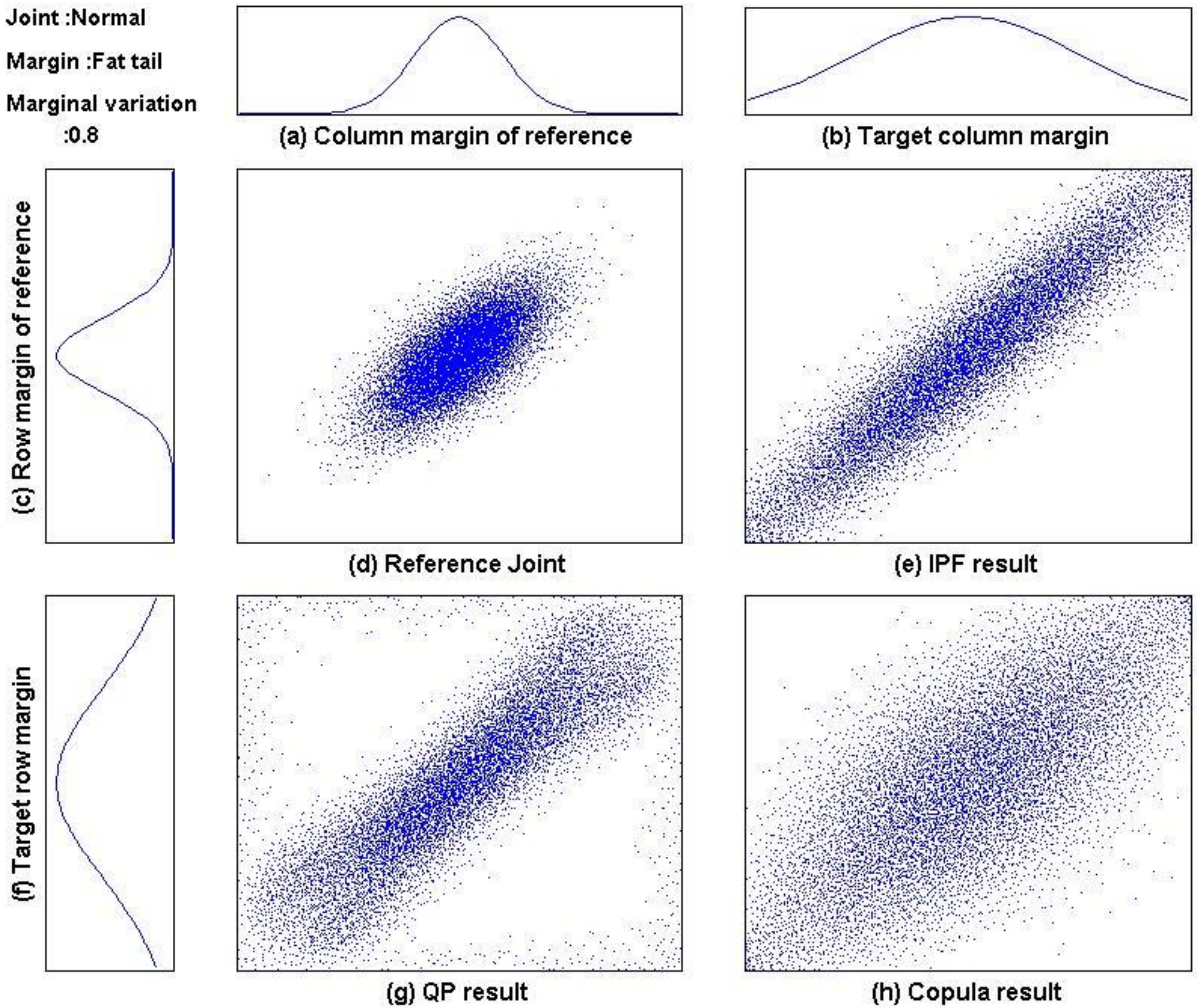

2-2-5. Reference joint distribution : Bivariate normal, Target marginal type : Fat tail, Marginal variation : 1.0

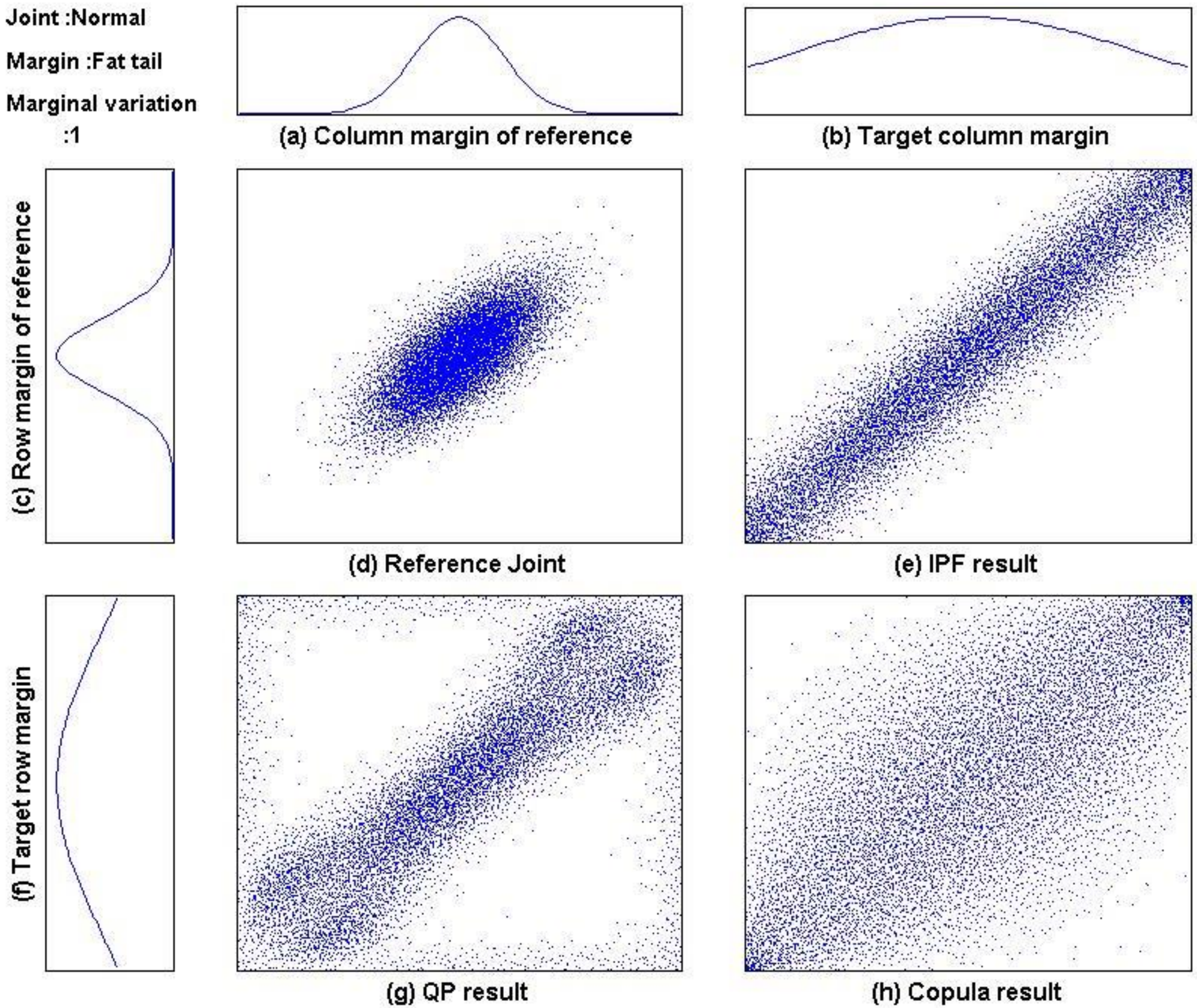

2-3-1. Reference joint distribution : Bivariate normal, Target marginal type : Thin tail, Marginal variation : 0.1

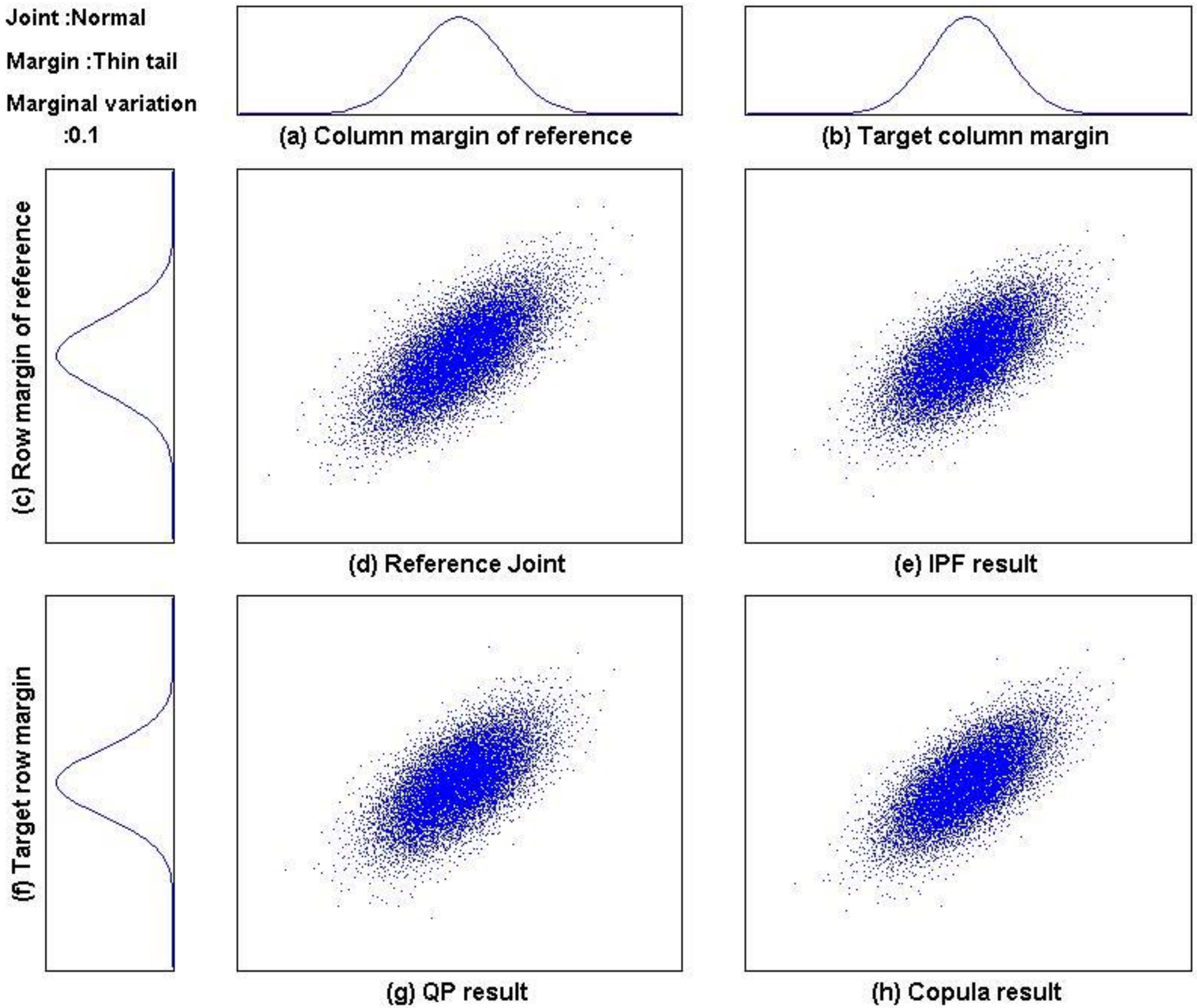

2-3-2. Reference joint distribution : Bivariate normal, Target marginal type : Thin tail, Marginal variation : 0.2

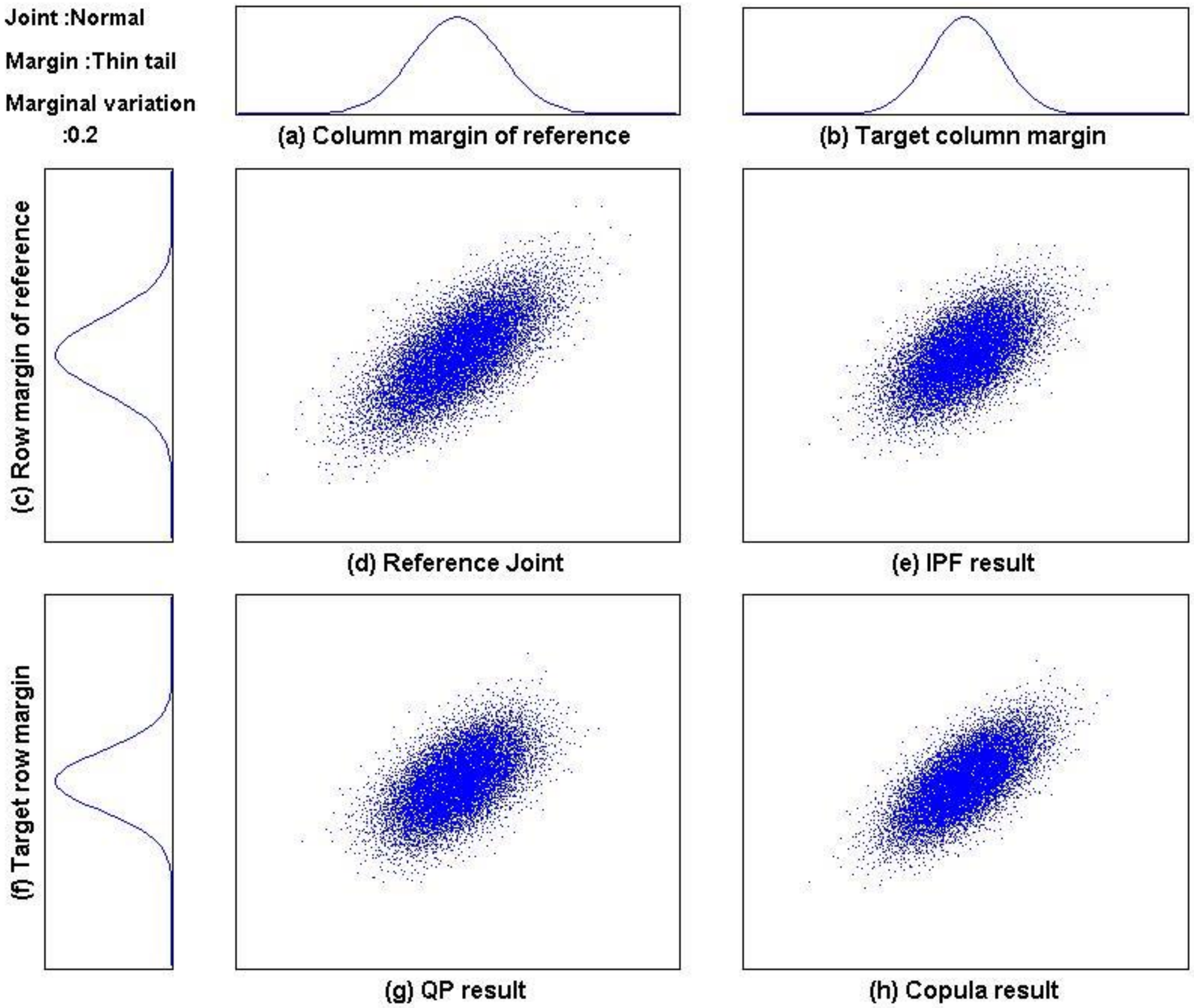

2-3-3. Reference joint distribution : Bivariate normal, Target marginal type : Thin tail, Marginal variation : 0.3

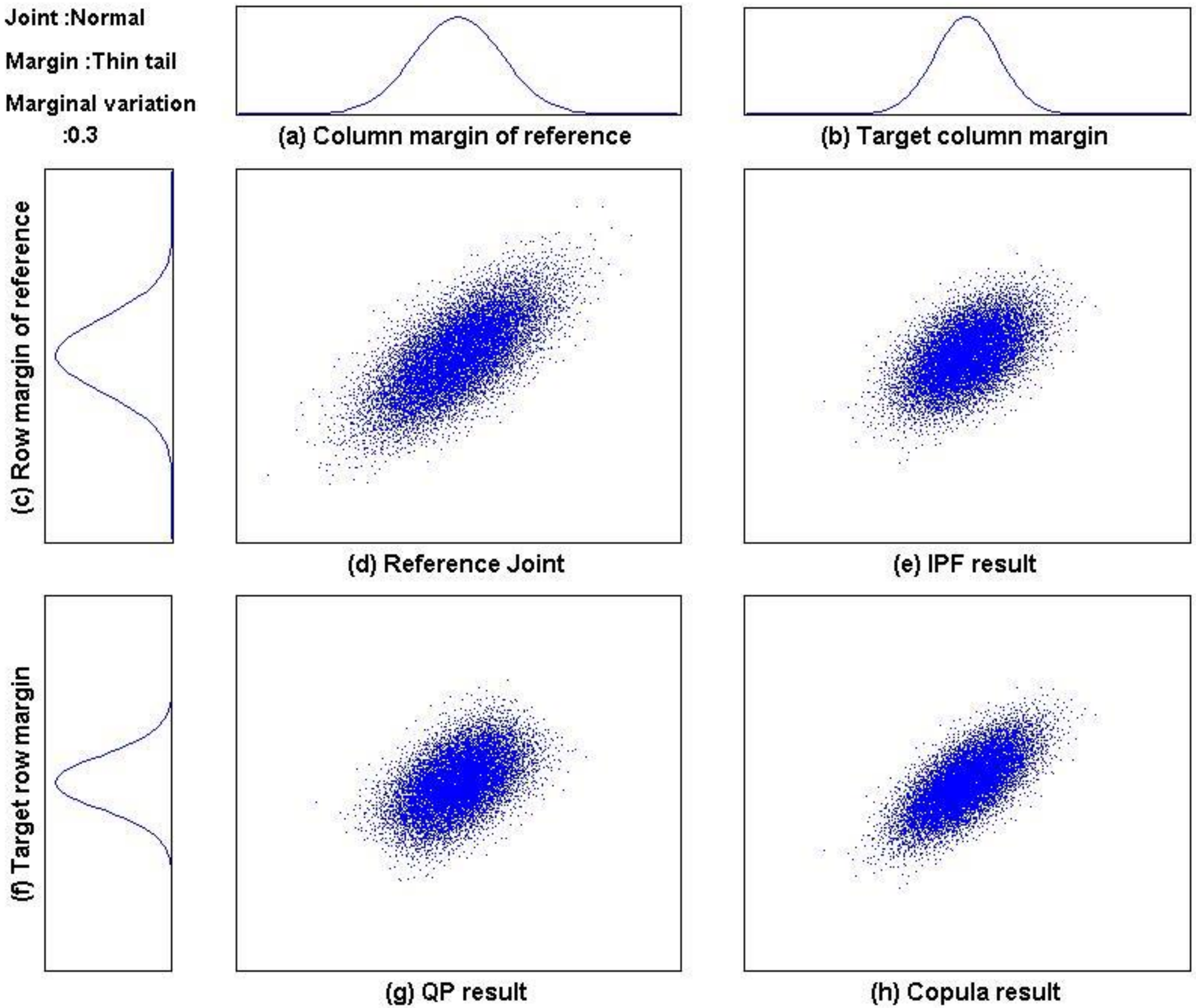

2-3-4. Reference joint distribution : Bivariate normal, Target marginal type : Thin tail, Marginal variation : 0.4

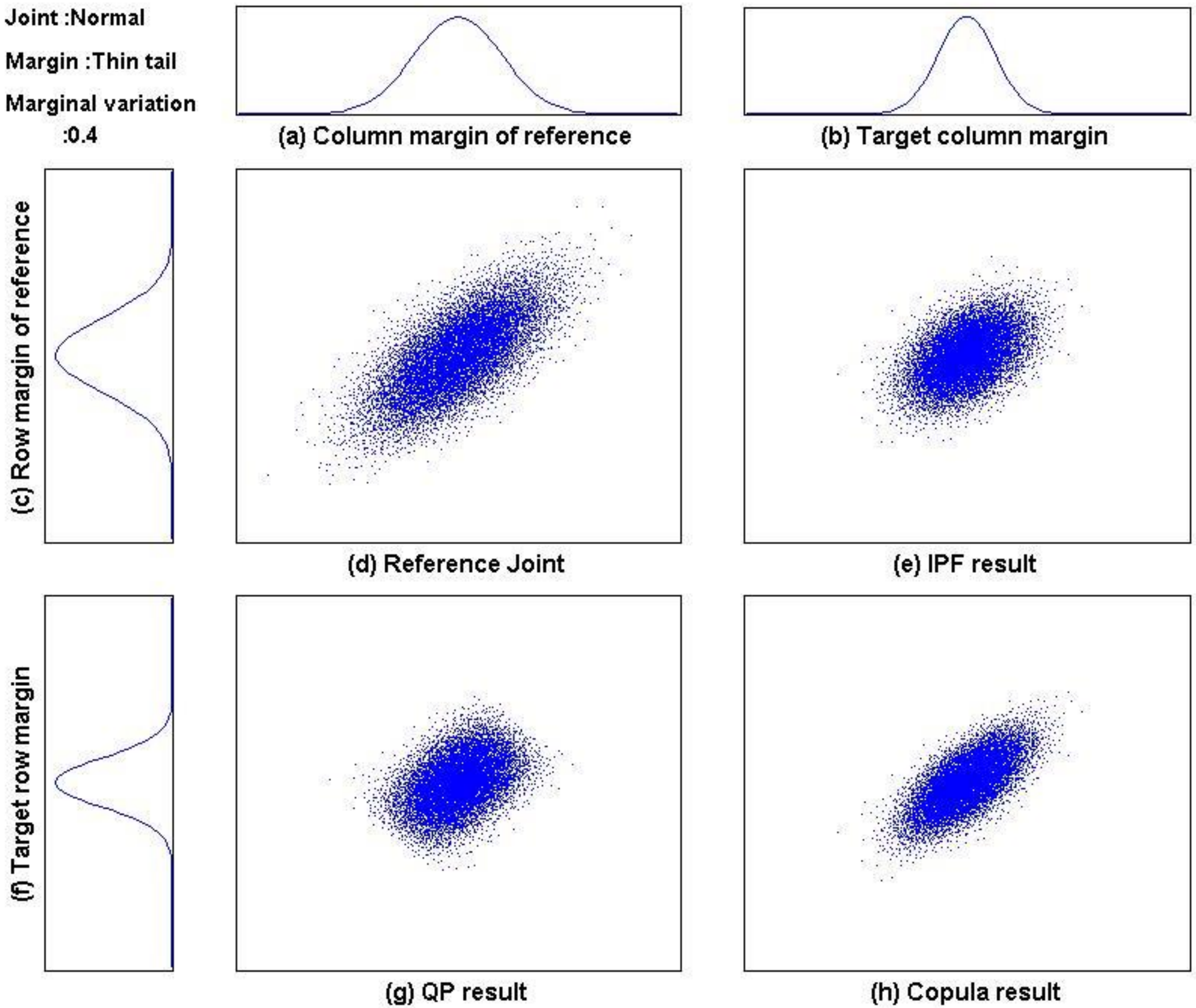

2-3-5. Reference joint distribution : Bivariate normal, Target marginal type : Thin tail, Marginal variation : 0.5

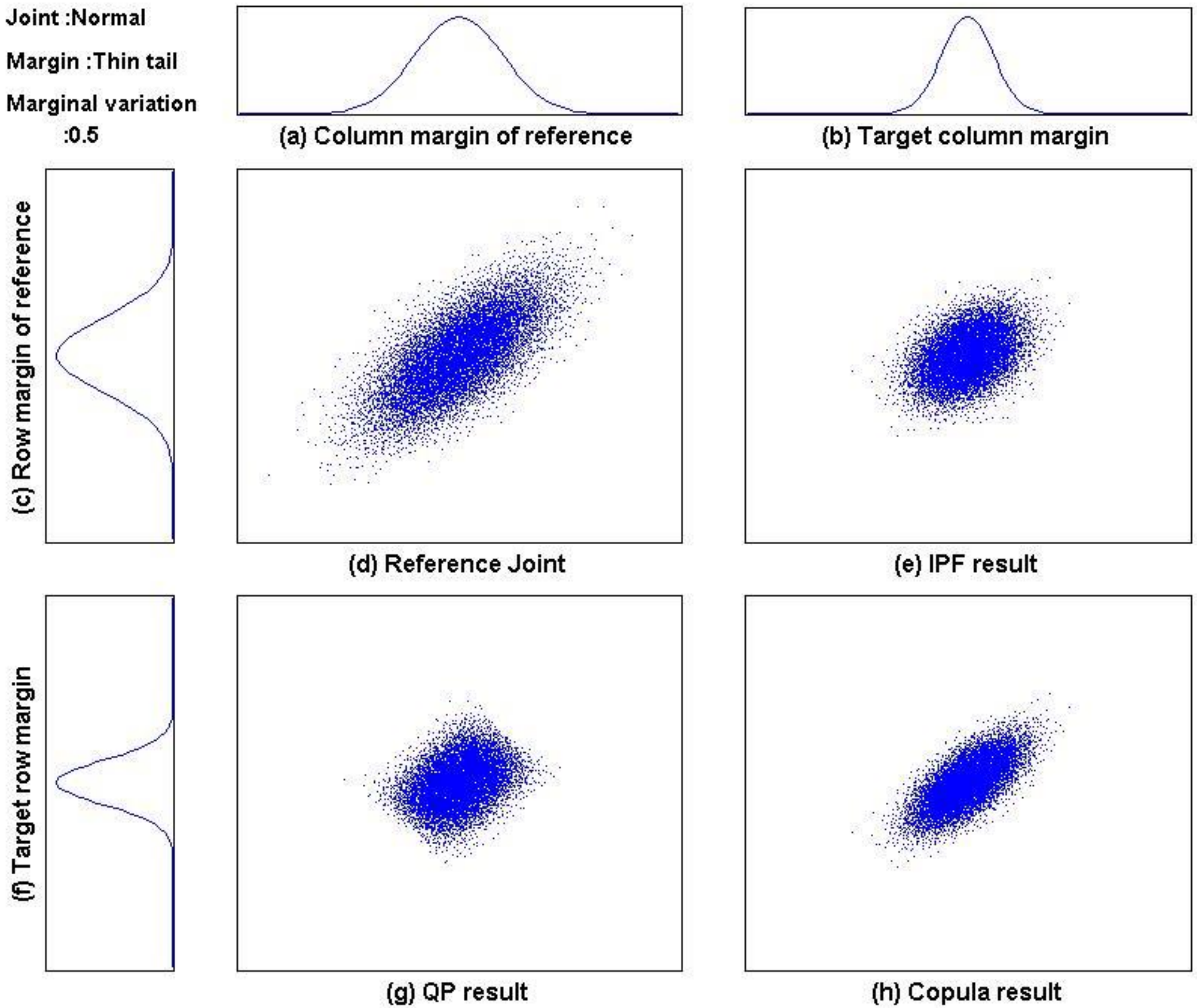

2-4-1. Reference joint distribution : Bimodal, Target marginal type : Skew, Marginal variation : 0.10

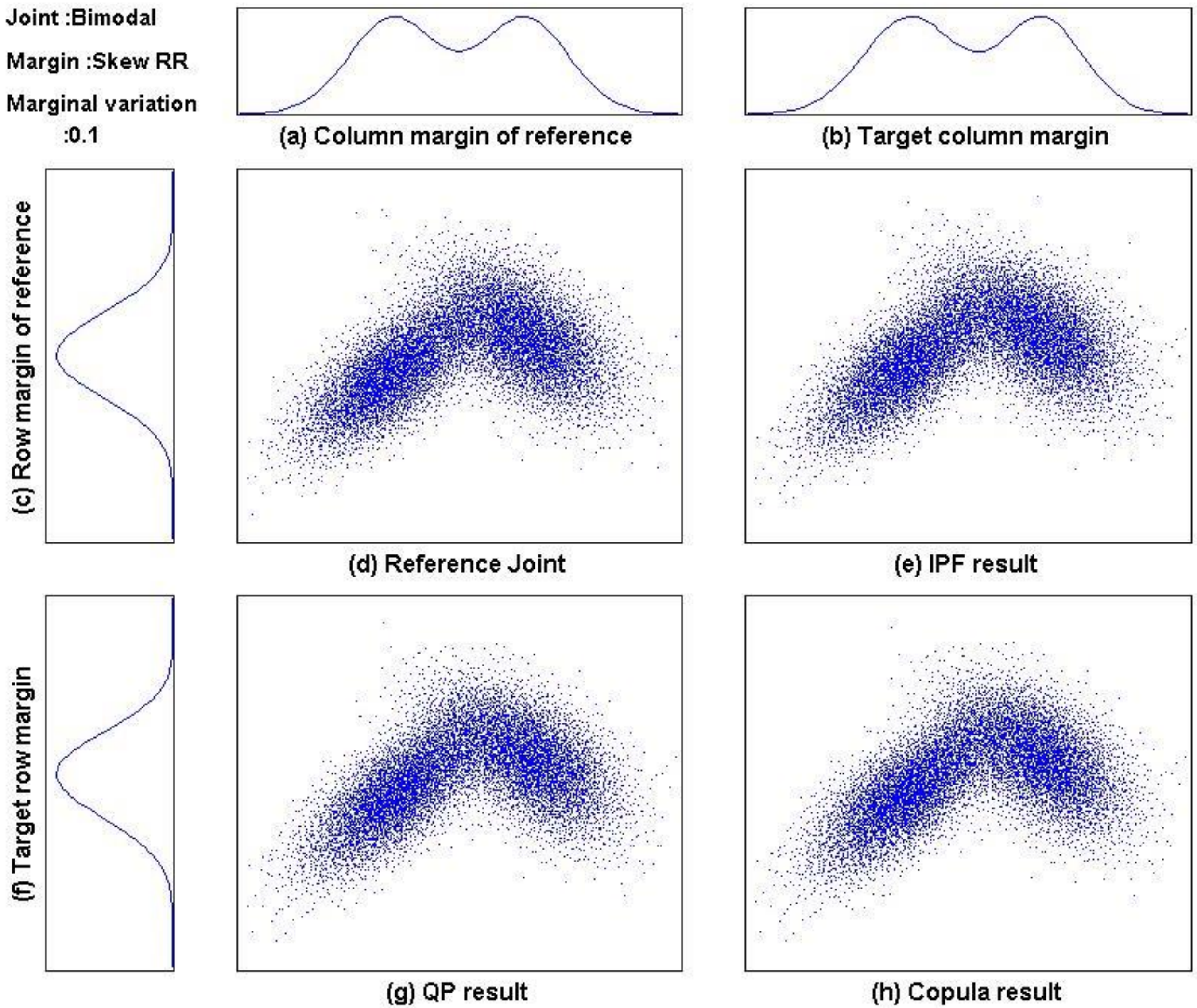

2-4-2. Reference joint distribution : Bimodal, Target marginal type : Skew, Marginal variation : 0.22

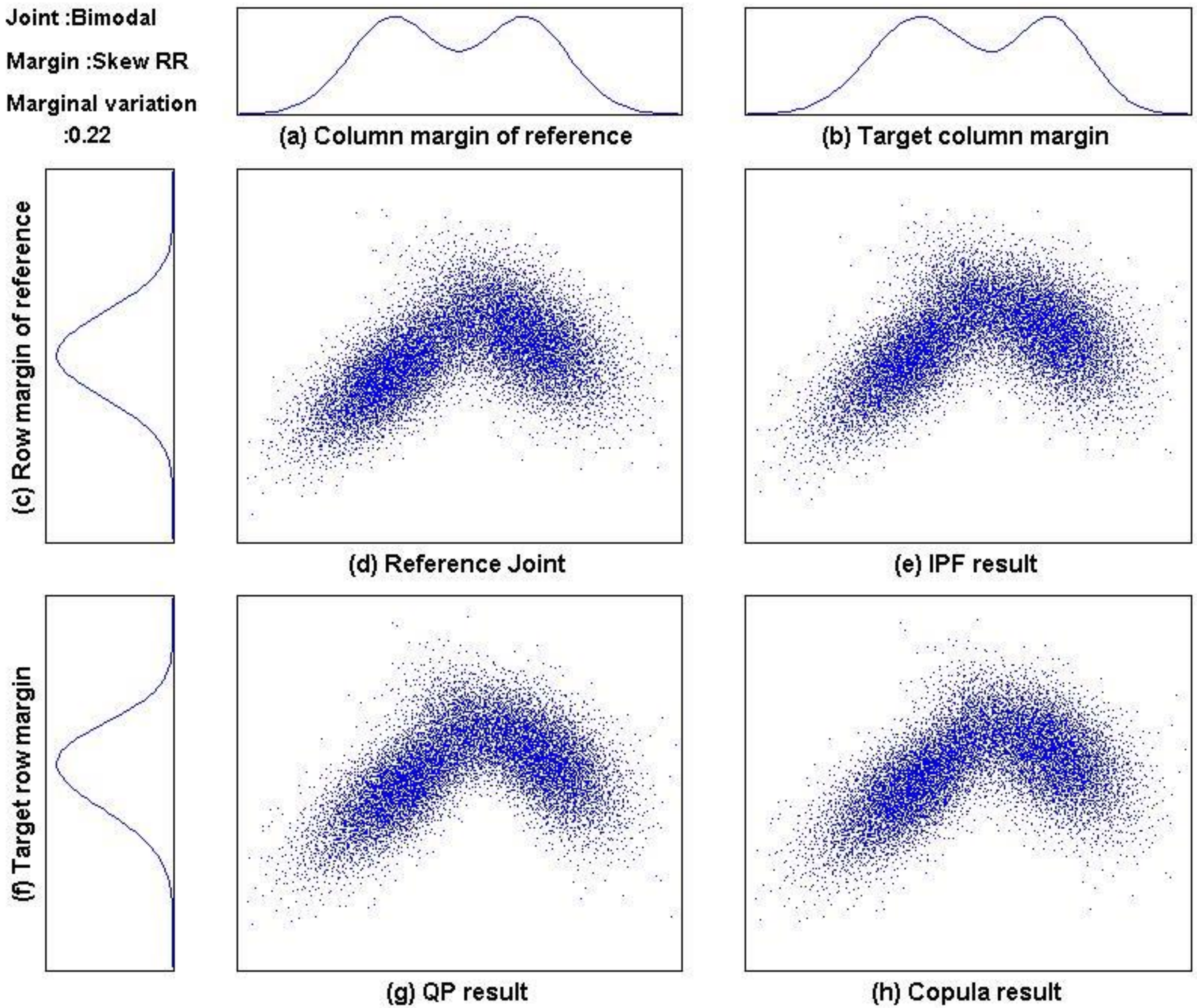

2-4-3. Reference joint distribution : Bimodal, Target marginal type : Skew, Marginal variation : 0.33

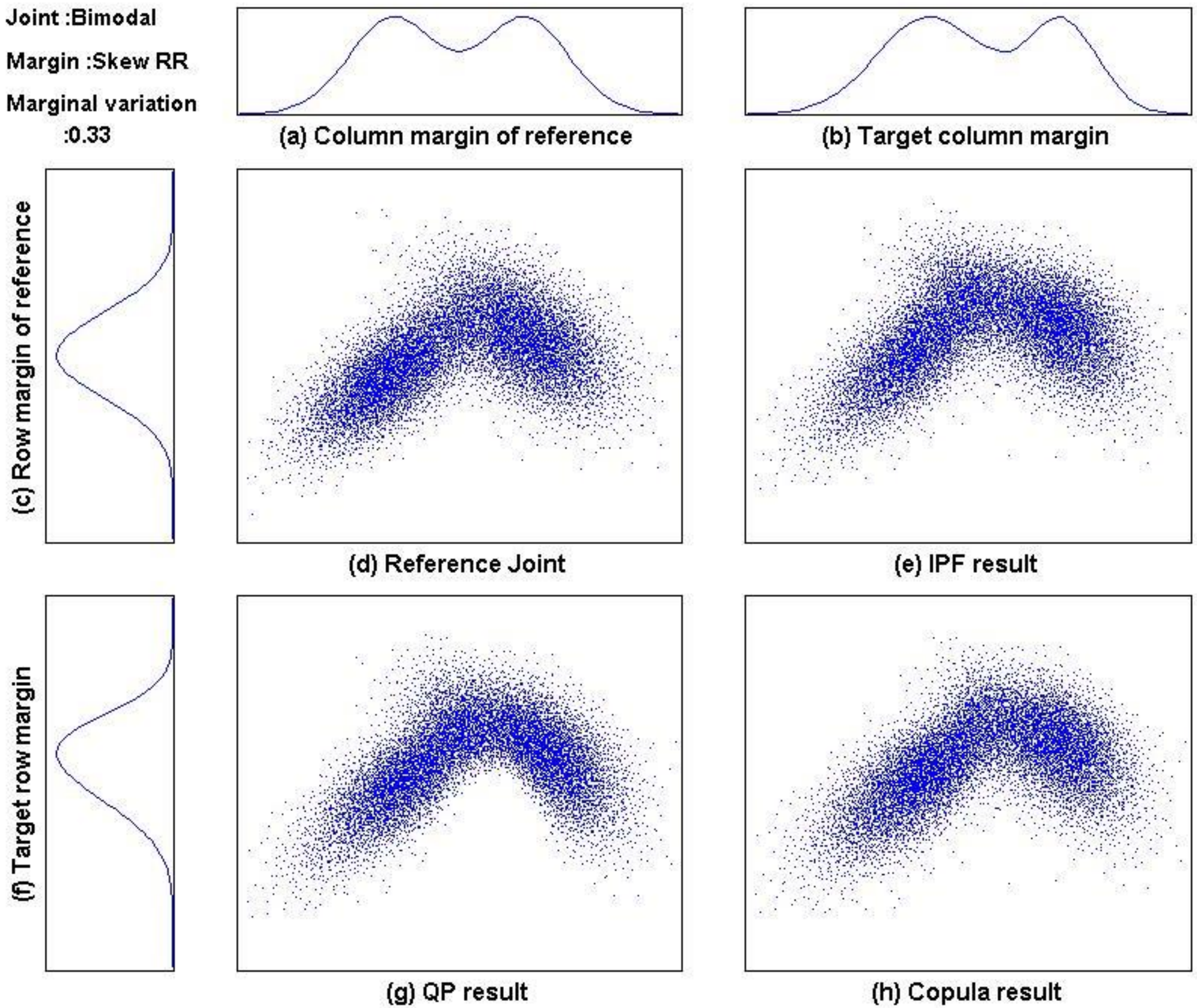

2-4-4. Reference joint distribution : Bimodal, Target marginal type : Skew, Marginal variation : 0.43

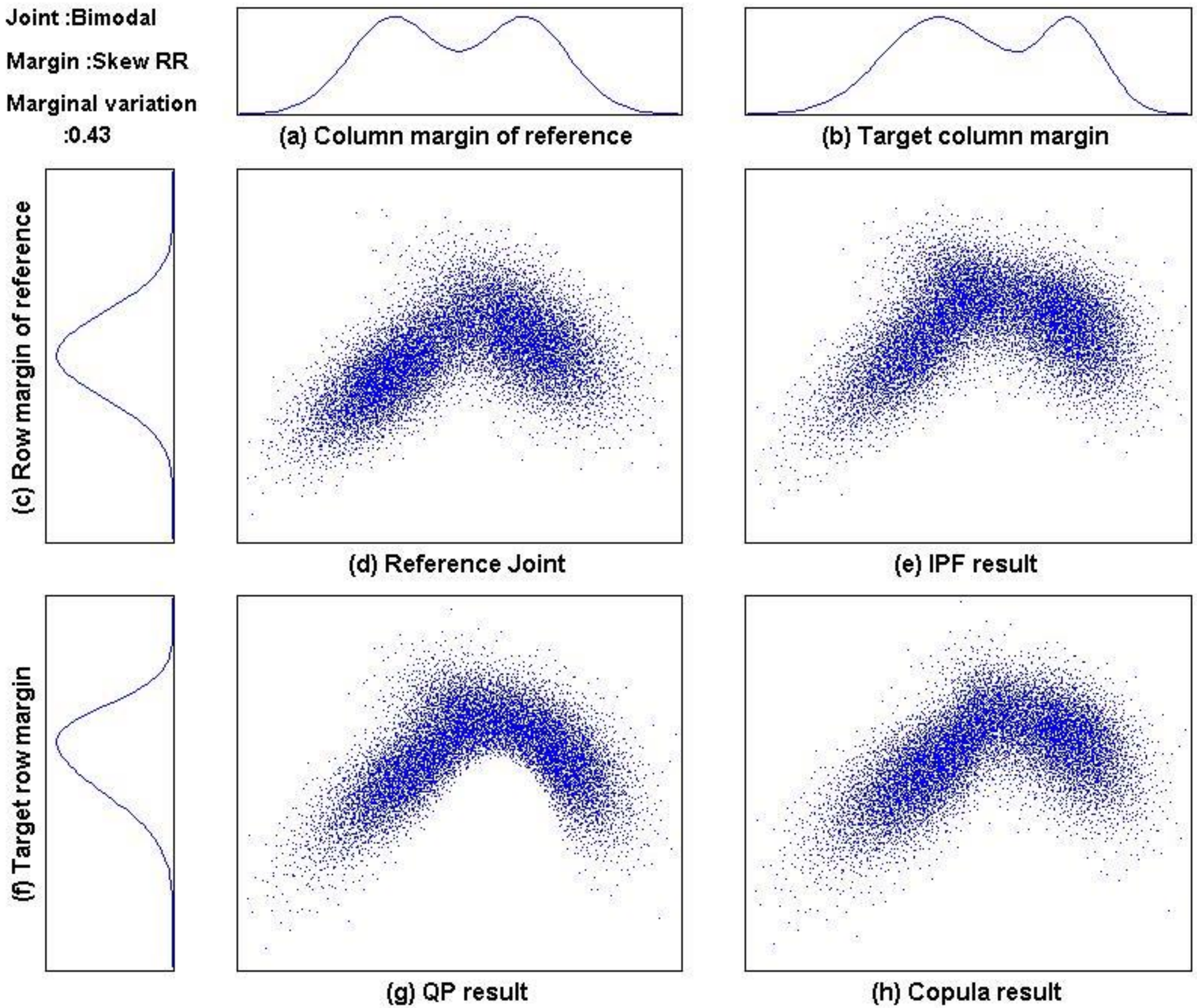

2-4-5. Reference joint distribution : Bimodal, Target marginal type : Skew, Marginal variation : 0.53

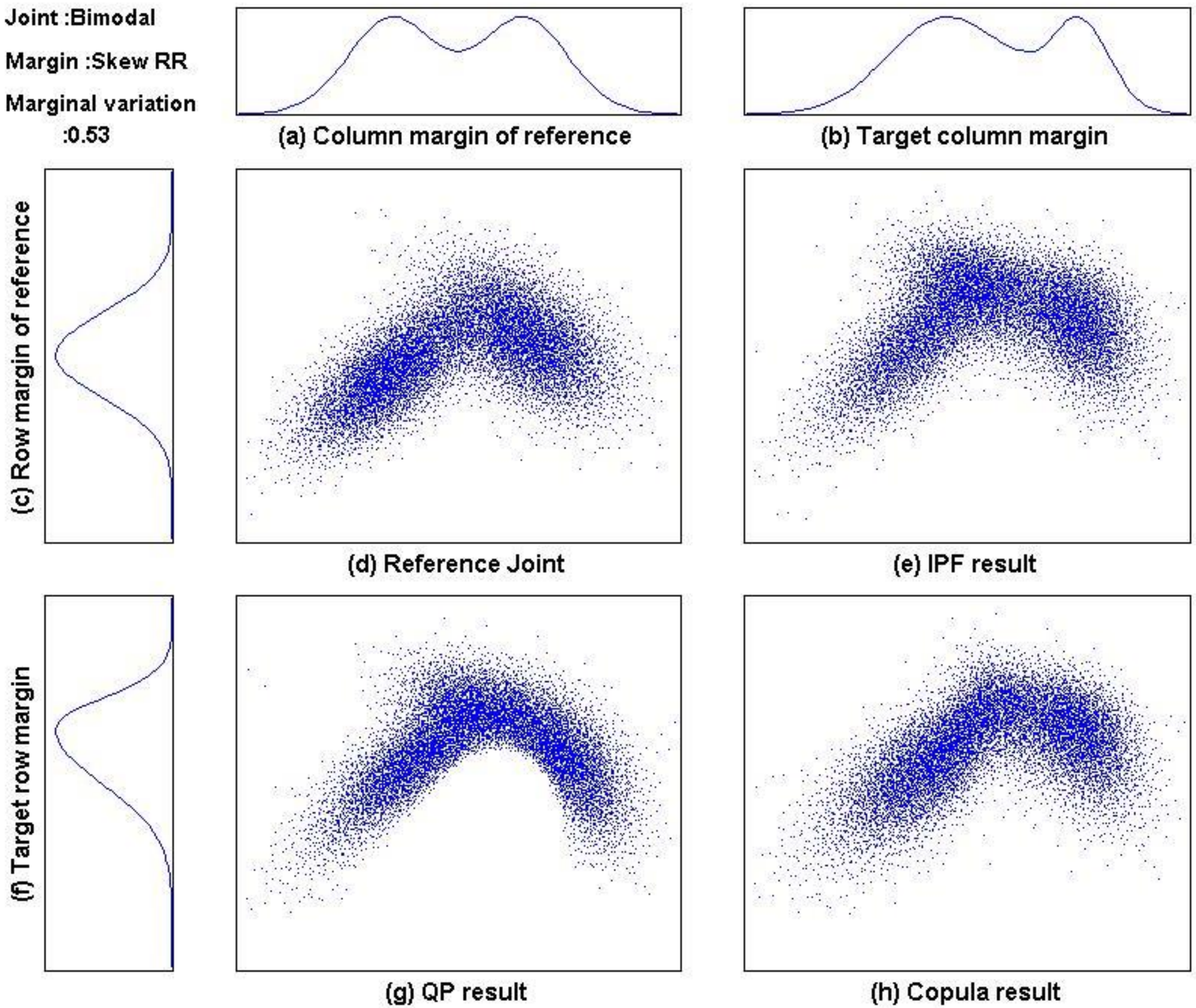

2-4-6. Reference joint distribution : Bimodal, Target marginal type : Skew, Marginal variation : 0.64

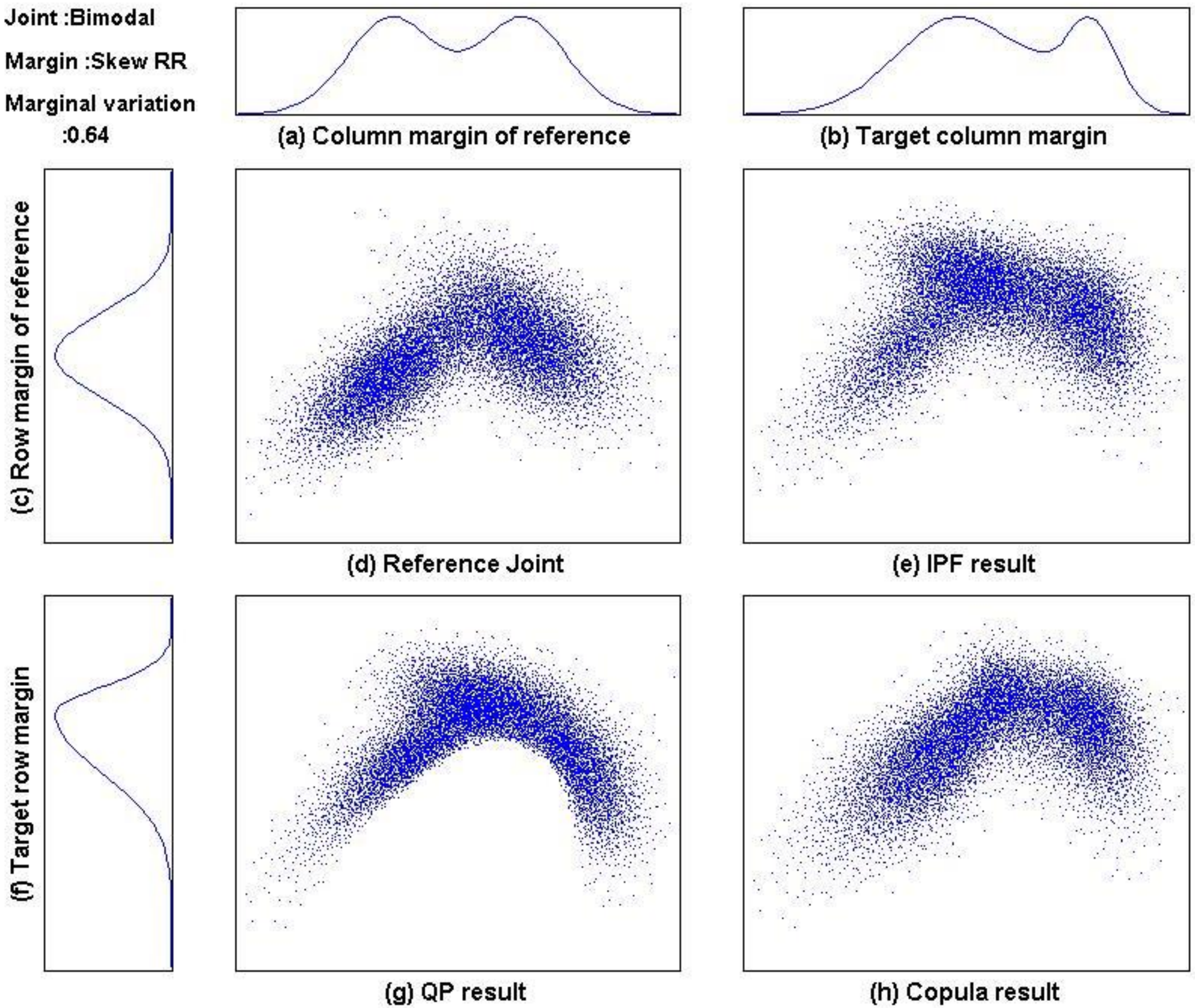

2-5-1. Reference joint distribution : Bimodal, Target marginal type : Fat tail, Marginal variation : 0.2

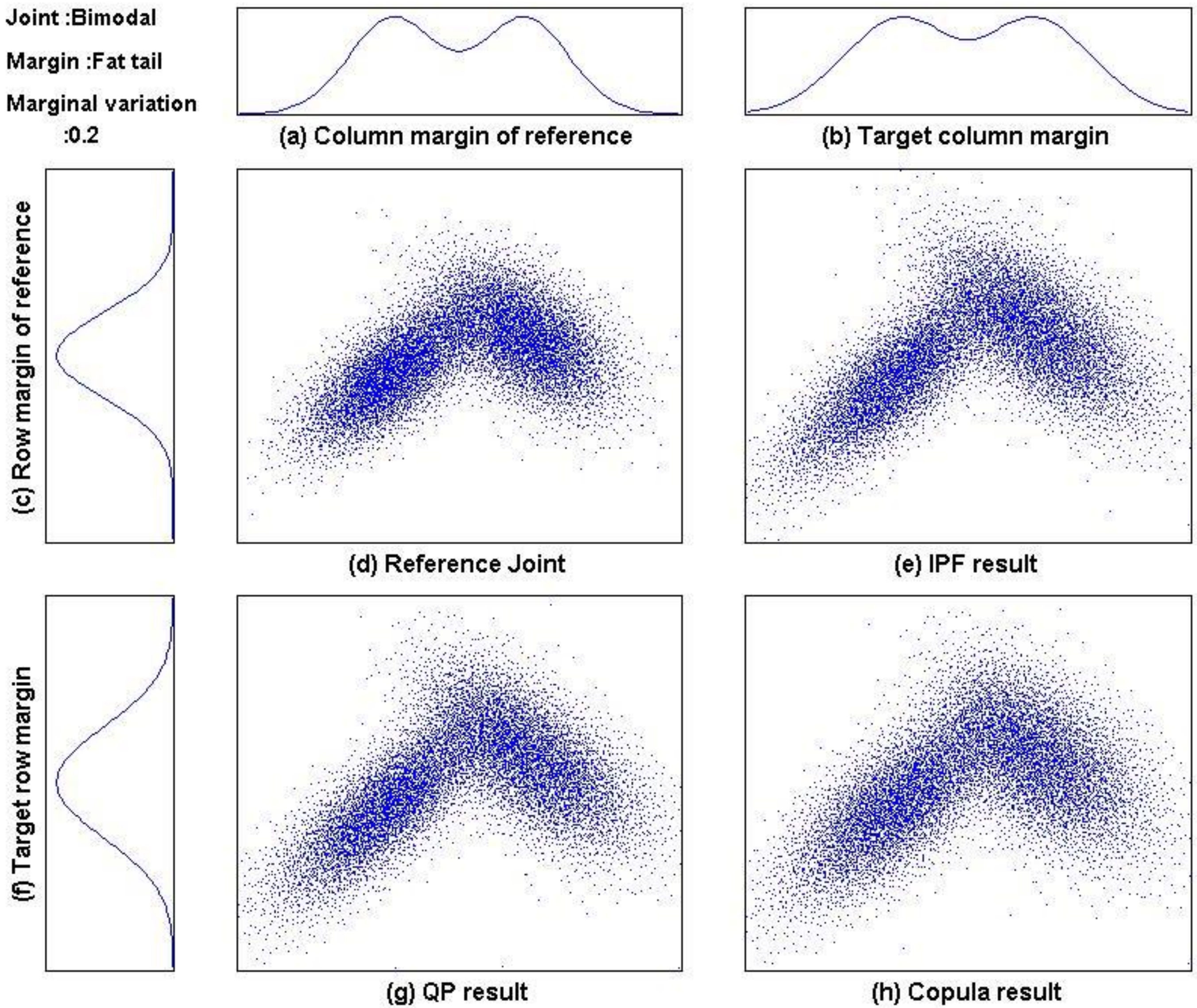

2-5-2. Reference joint distribution : Bimodal, Target marginal type : Fat tail, Marginal variation : 0.4

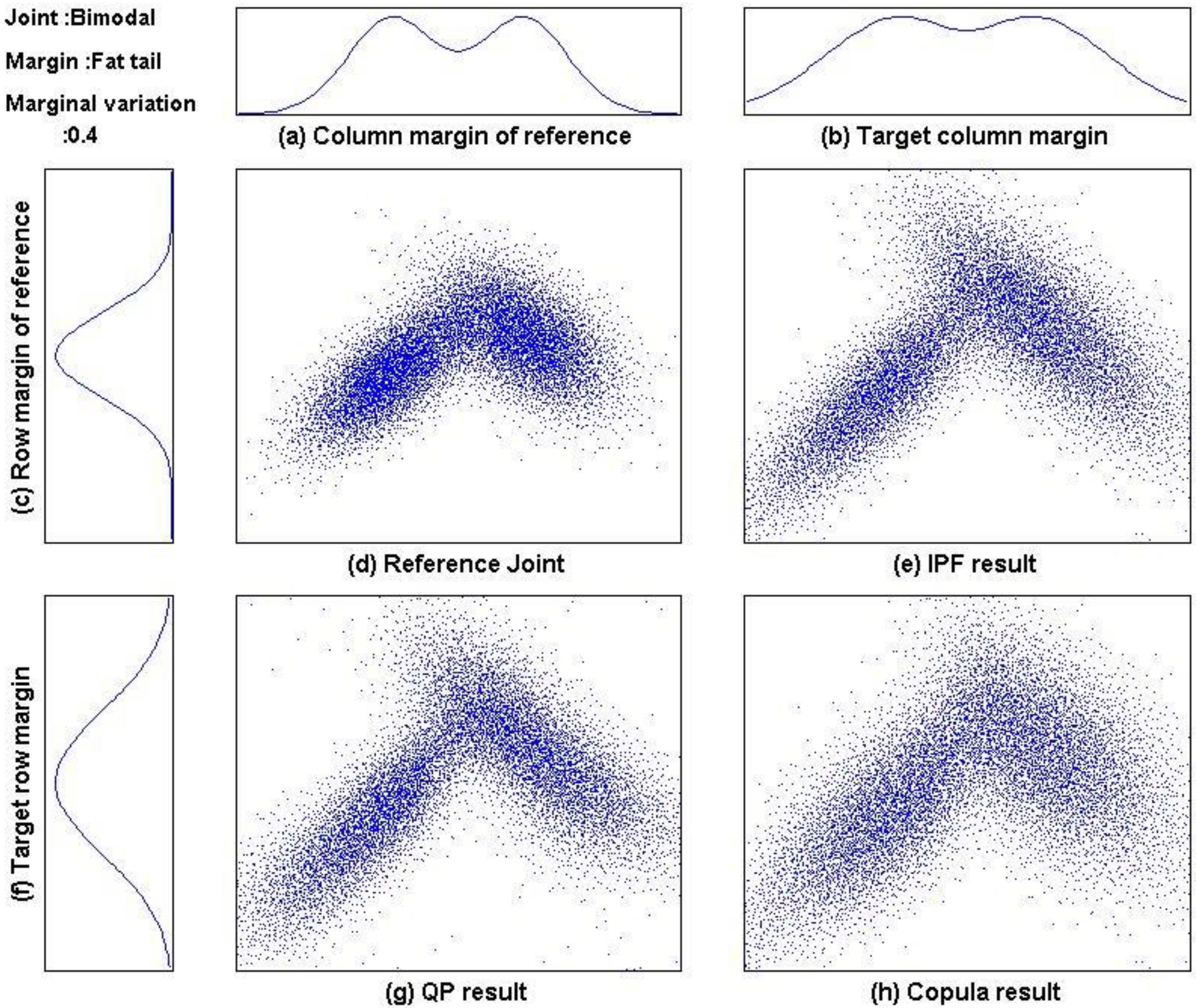

2-5-3. Reference joint distribution : Bimodal, Target marginal type : Fat tail, Marginal variation : 0.6

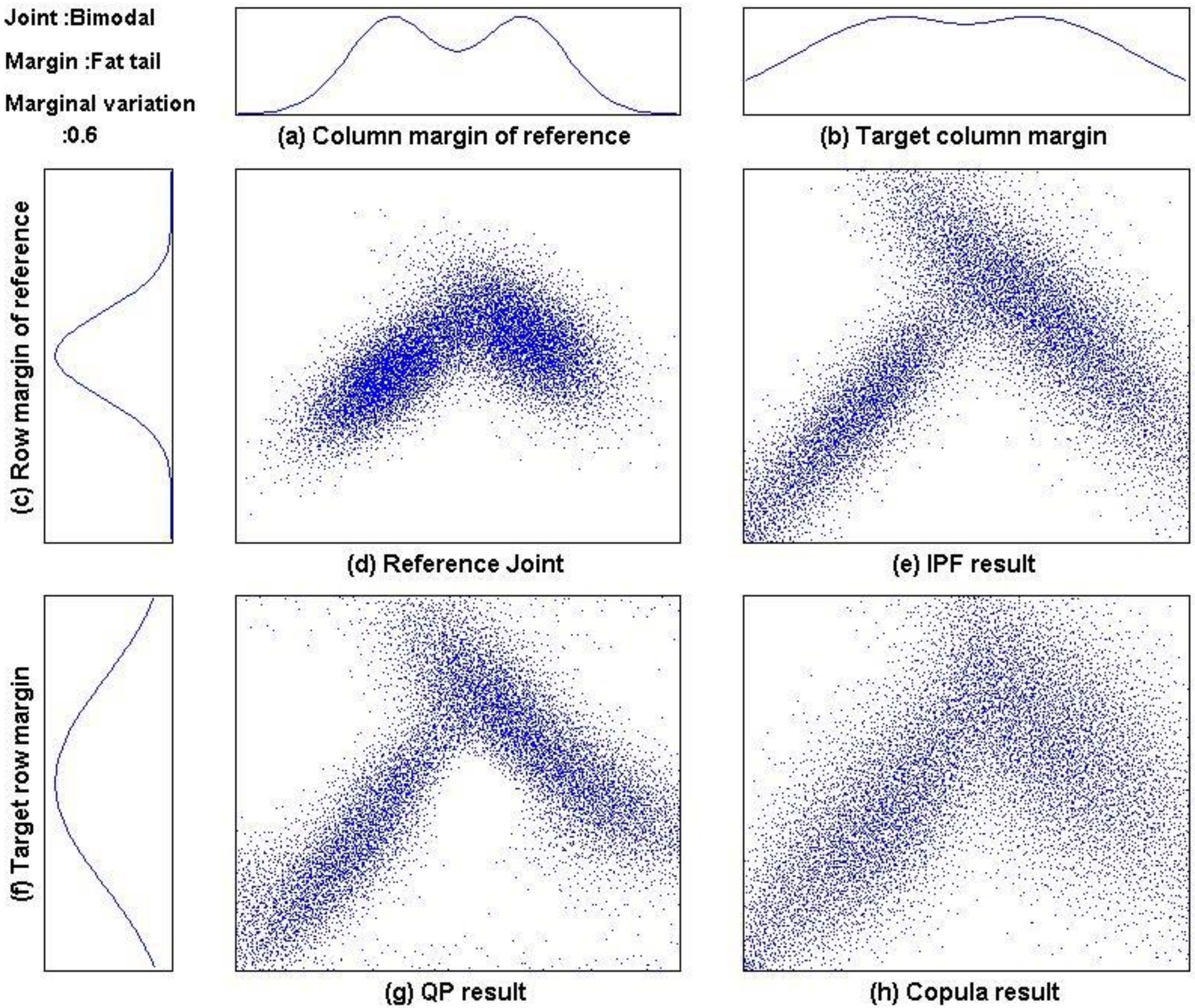

2-5-4. Reference joint distribution : Bimodal, Target marginal type : Fat tail, Marginal variation : 0.8

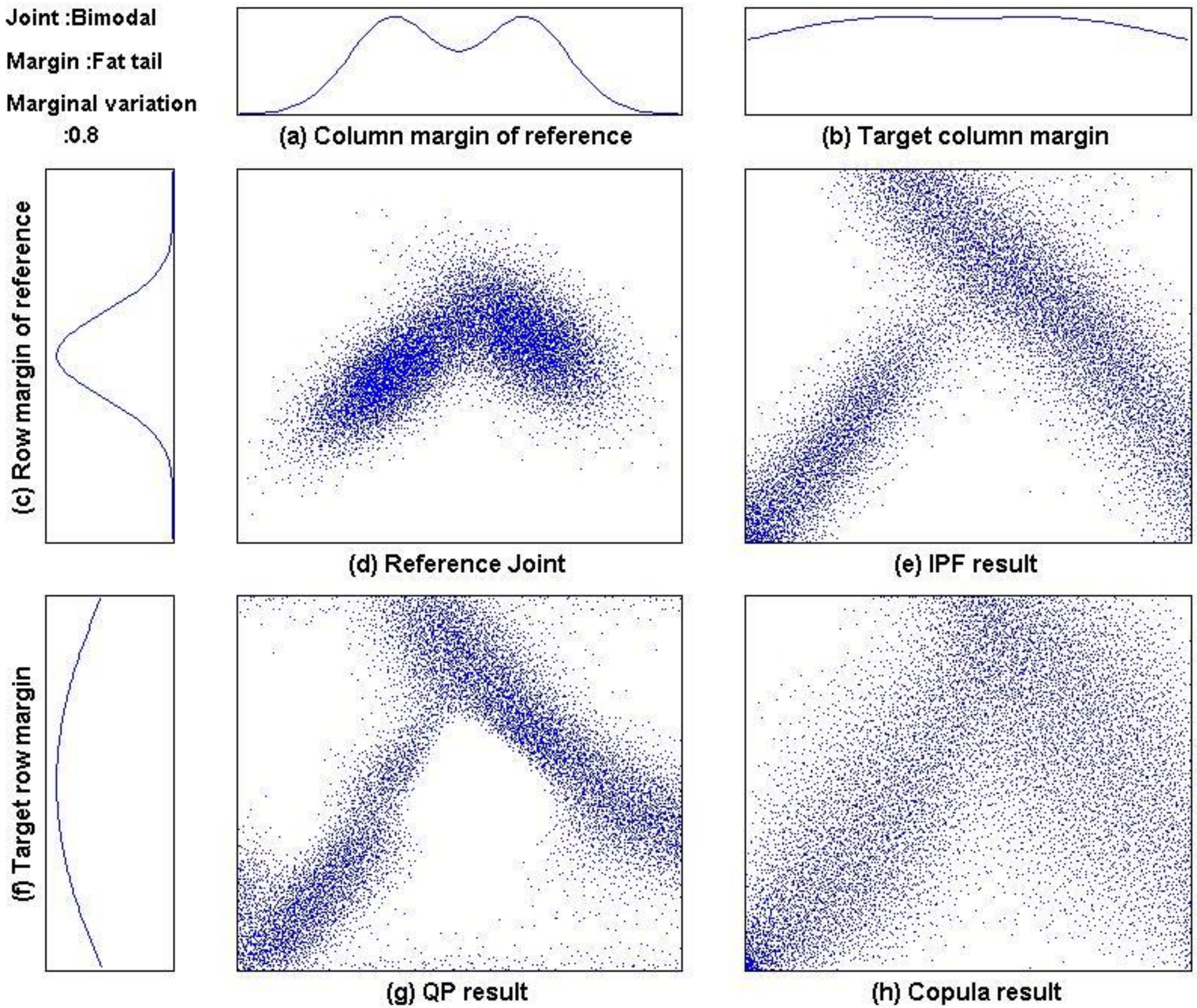

2-6-1. Reference joint distribution : Bimodal, Target marginal type : Thin tail, Marginal variation : 0.1

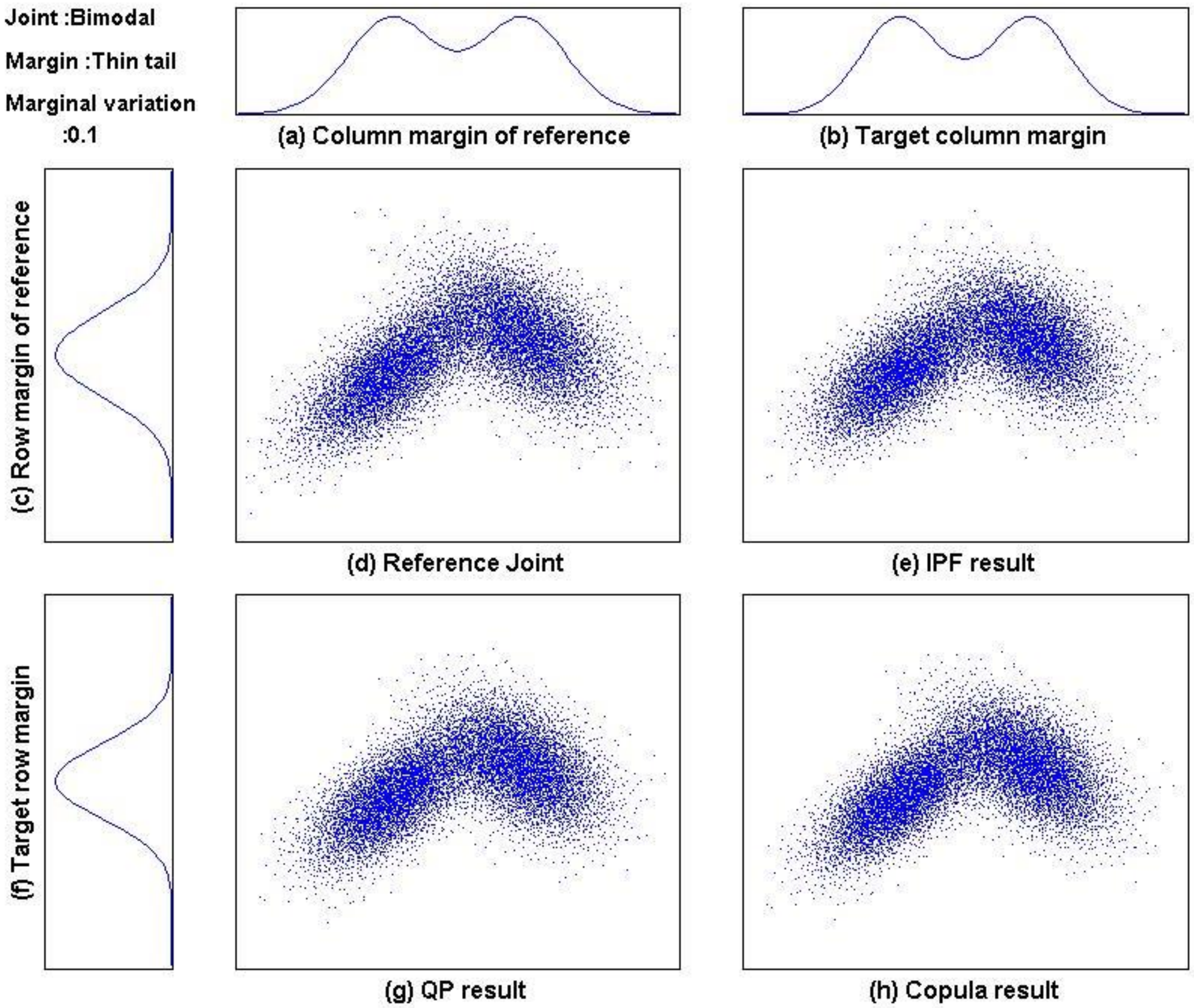

2-6-2. Reference joint distribution : Bimodal, Target marginal type : Thin tail, Marginal variation : 0.2

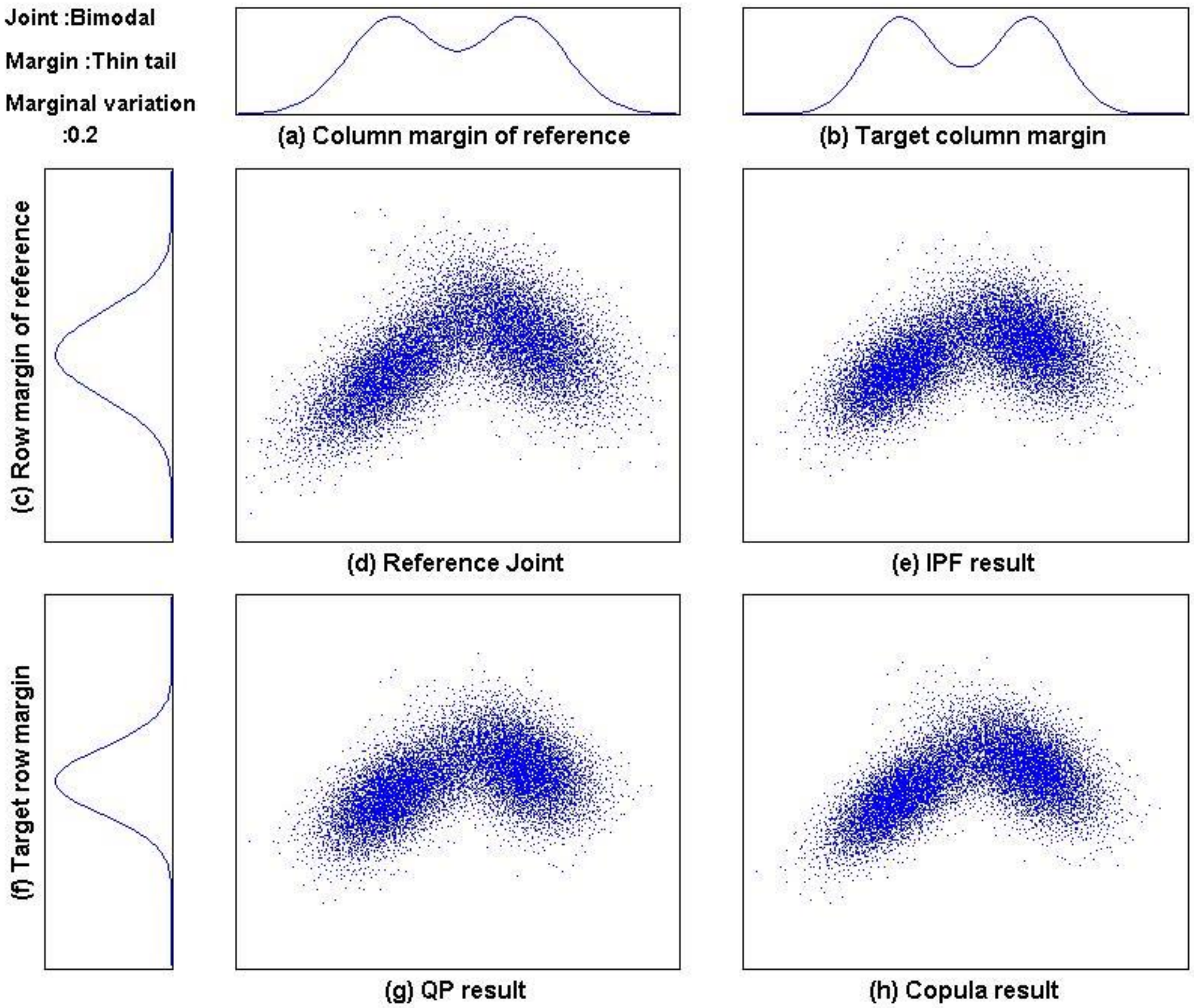

2-6-3. Reference joint distribution : Bimodal, Target marginal type : Thin tail, Marginal variation : 0.3

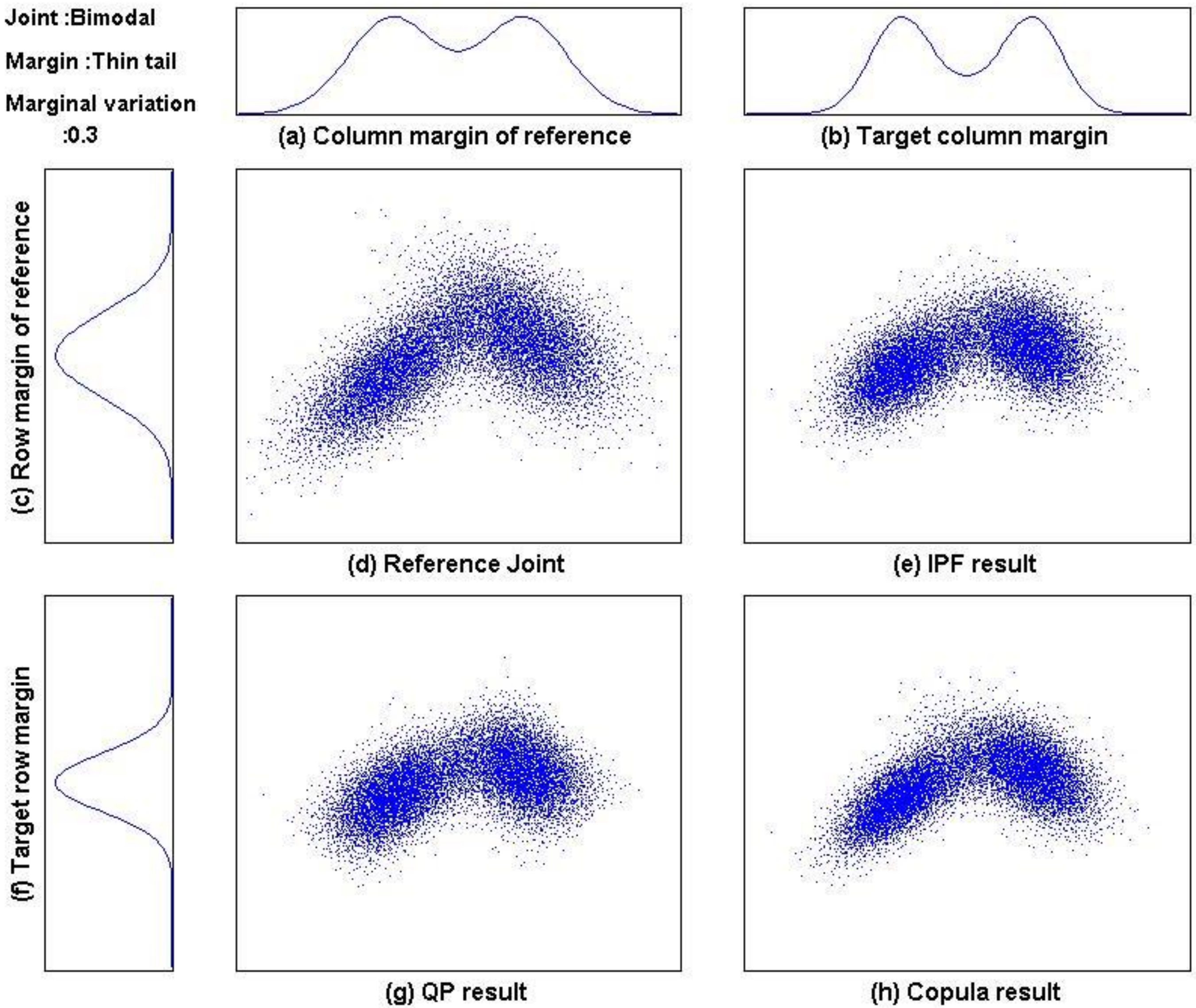

2-6-4. Reference joint distribution : Bimodal, Target marginal type : Thin tail, Marginal variation : 0.4

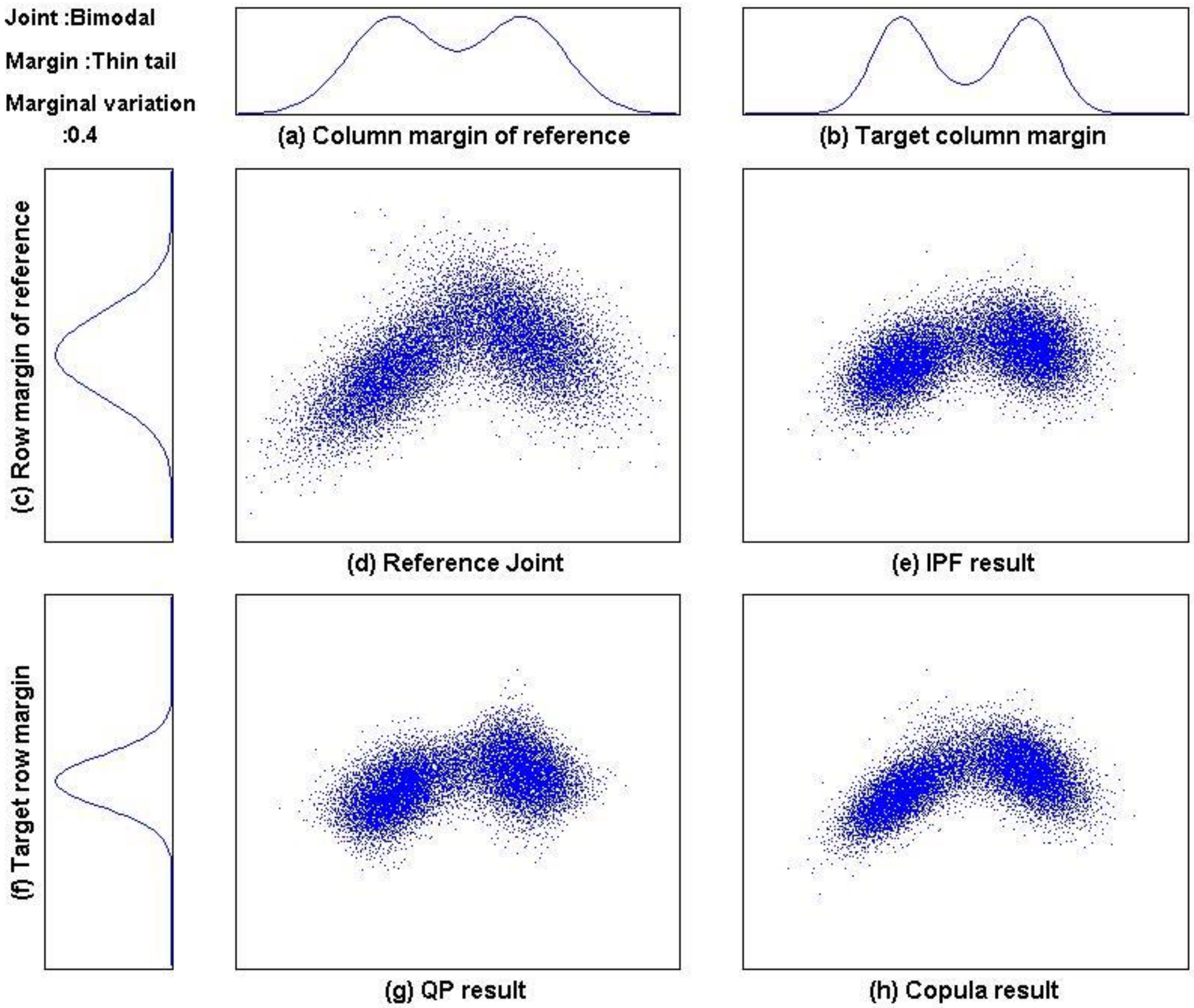

2-6-5. Reference joint distribution : Bimodal, Target marginal type : Thin tail, Marginal variation : 0.5

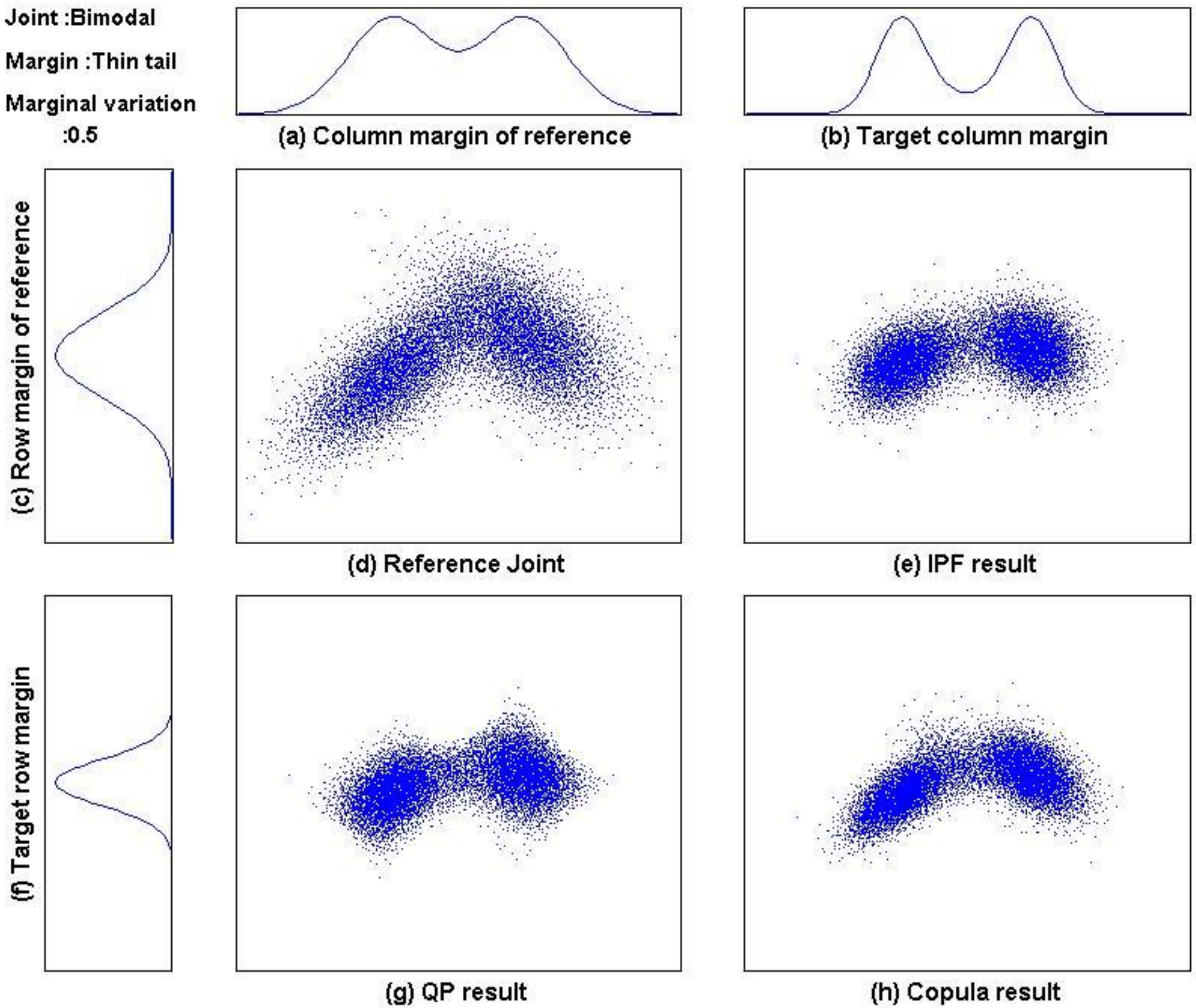

2-7-1. Reference joint distribution : Tail dependent, Target marginal type : Skew, Marginal variation : 0.19

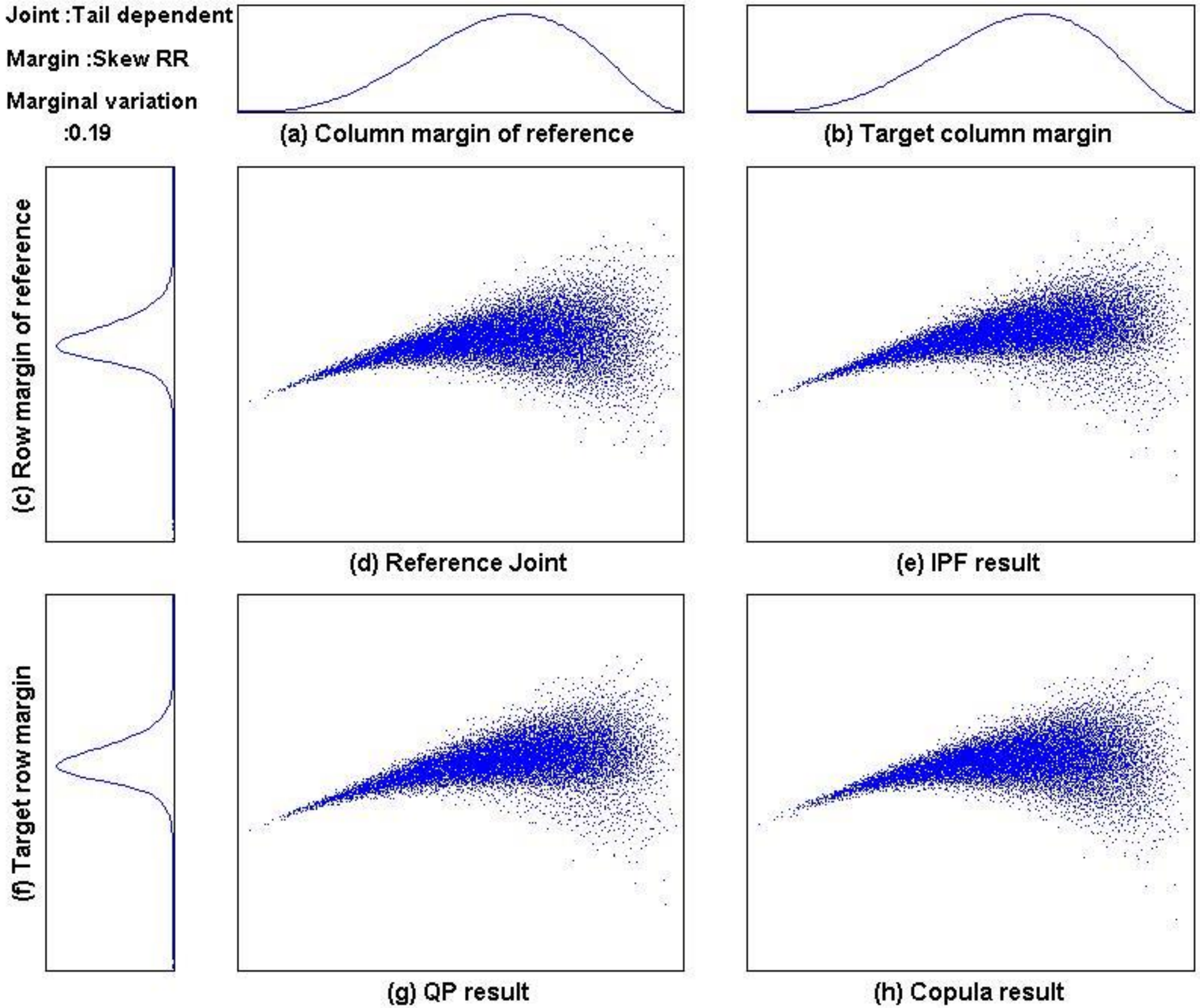

2-7-2. Reference joint distribution : Tail dependent, Target marginal type : Skew, Marginal variation : 0.39

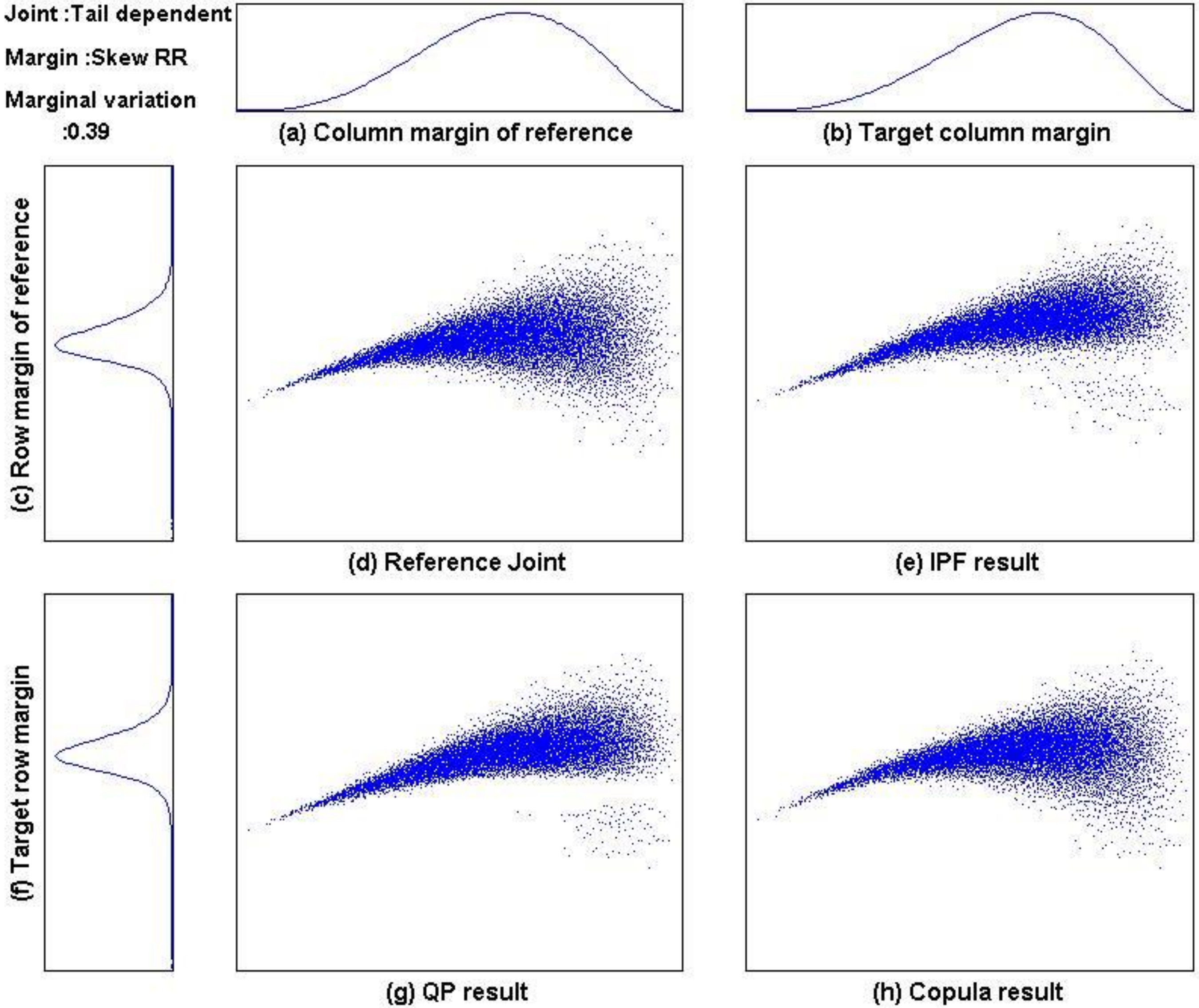

2-7-3. Reference joint distribution : Tail dependent, Target marginal type : Skew, Marginal variation : 0.60

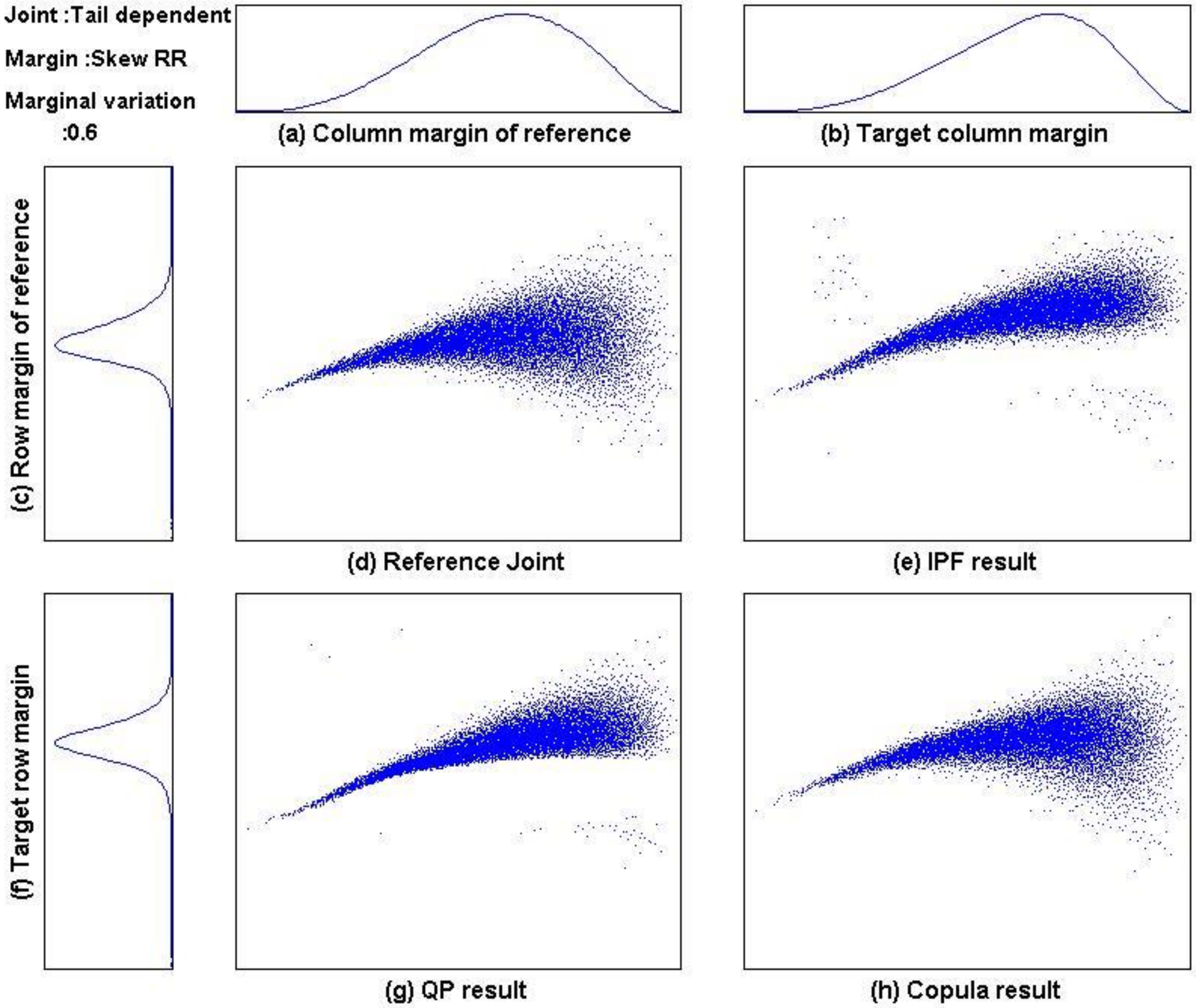

2-7-4. Reference joint distribution : Tail dependent, Target marginal type : Skew, Marginal variation : 0.81

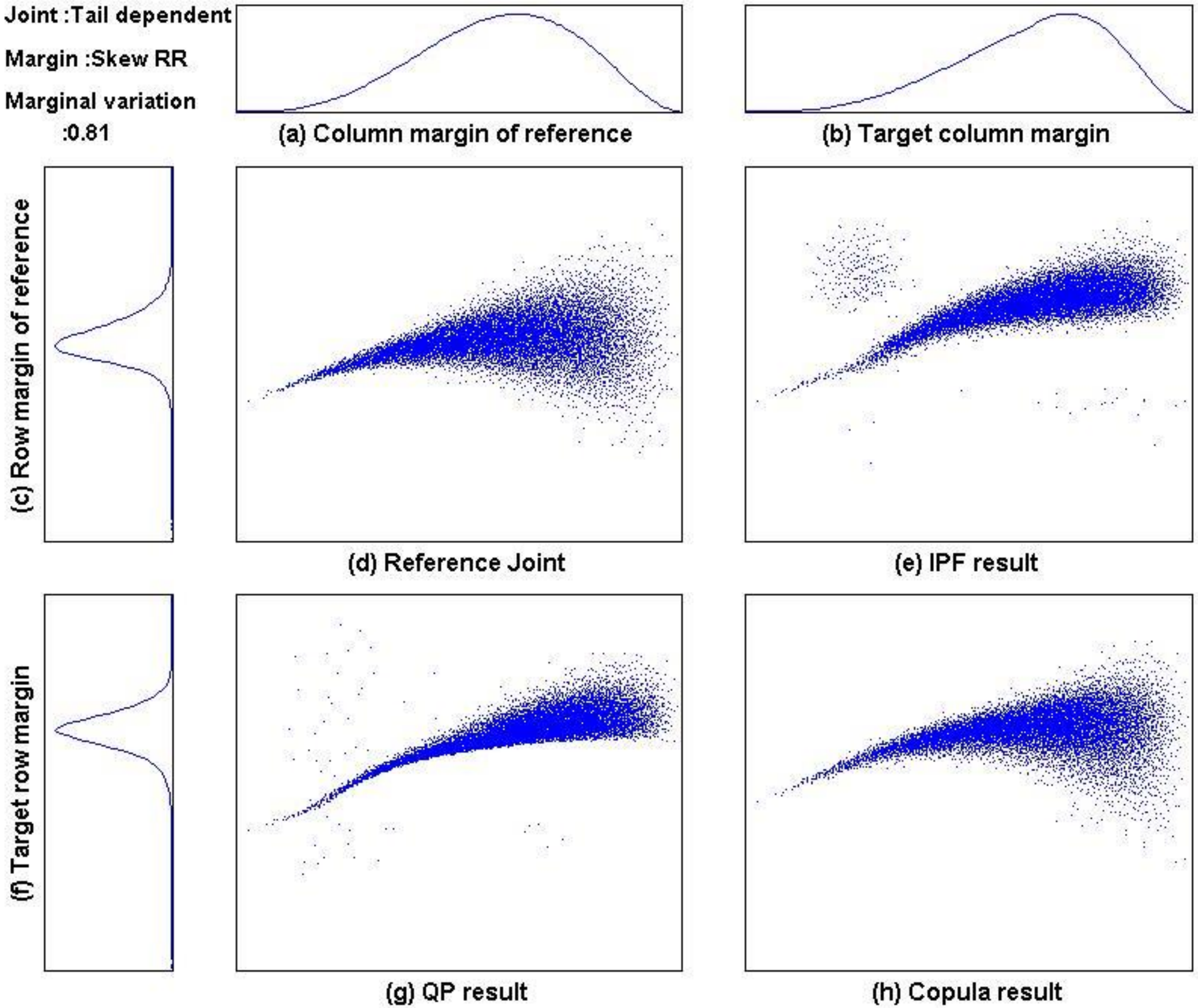

2-7-5. Reference joint distribution : Tail dependent, Target marginal type : Skew, Marginal variation : 1.01

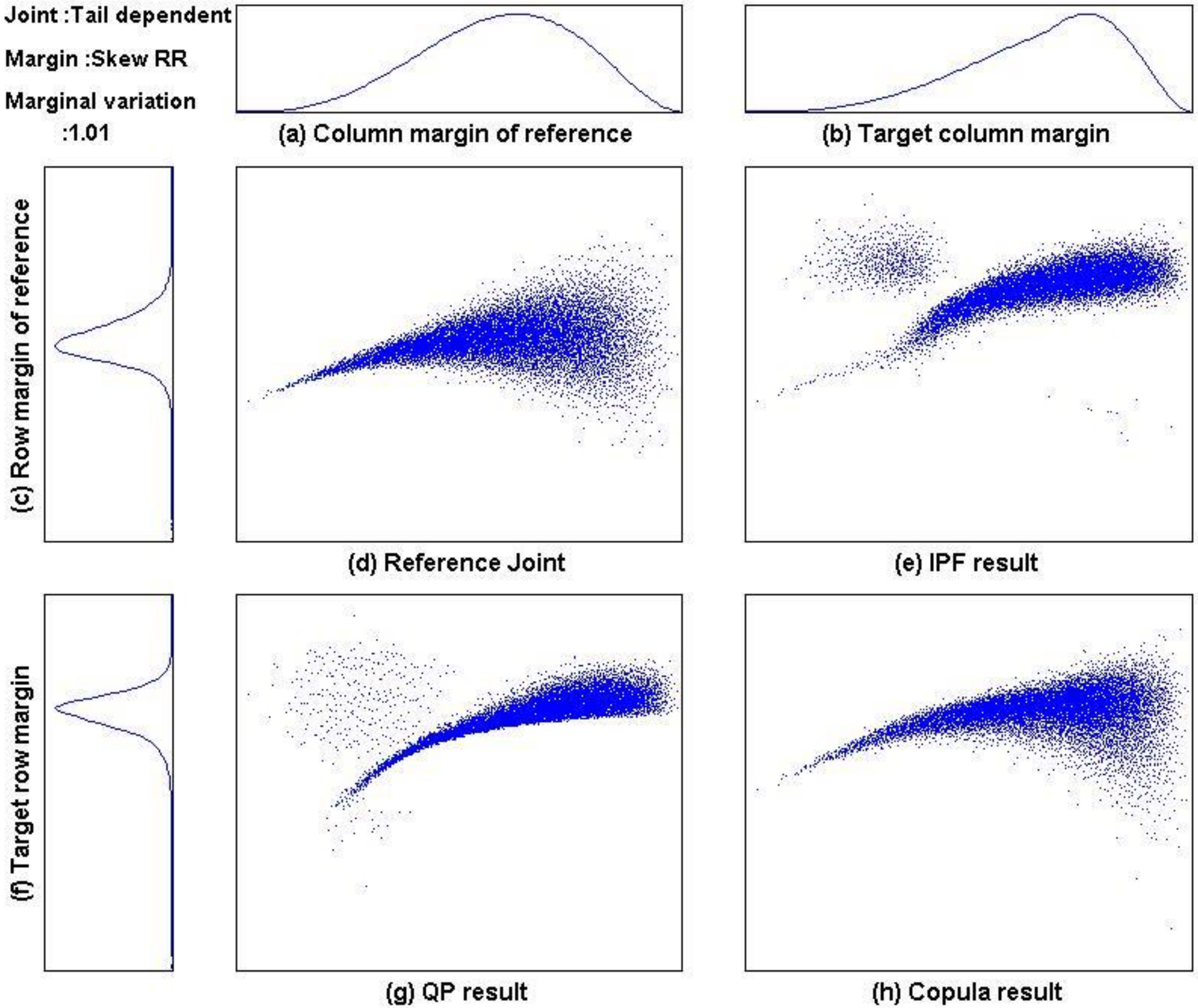

2-8-1. Reference joint distribution : Tail dependent, Target marginal type : Fat tail, Marginal variation : 0.1

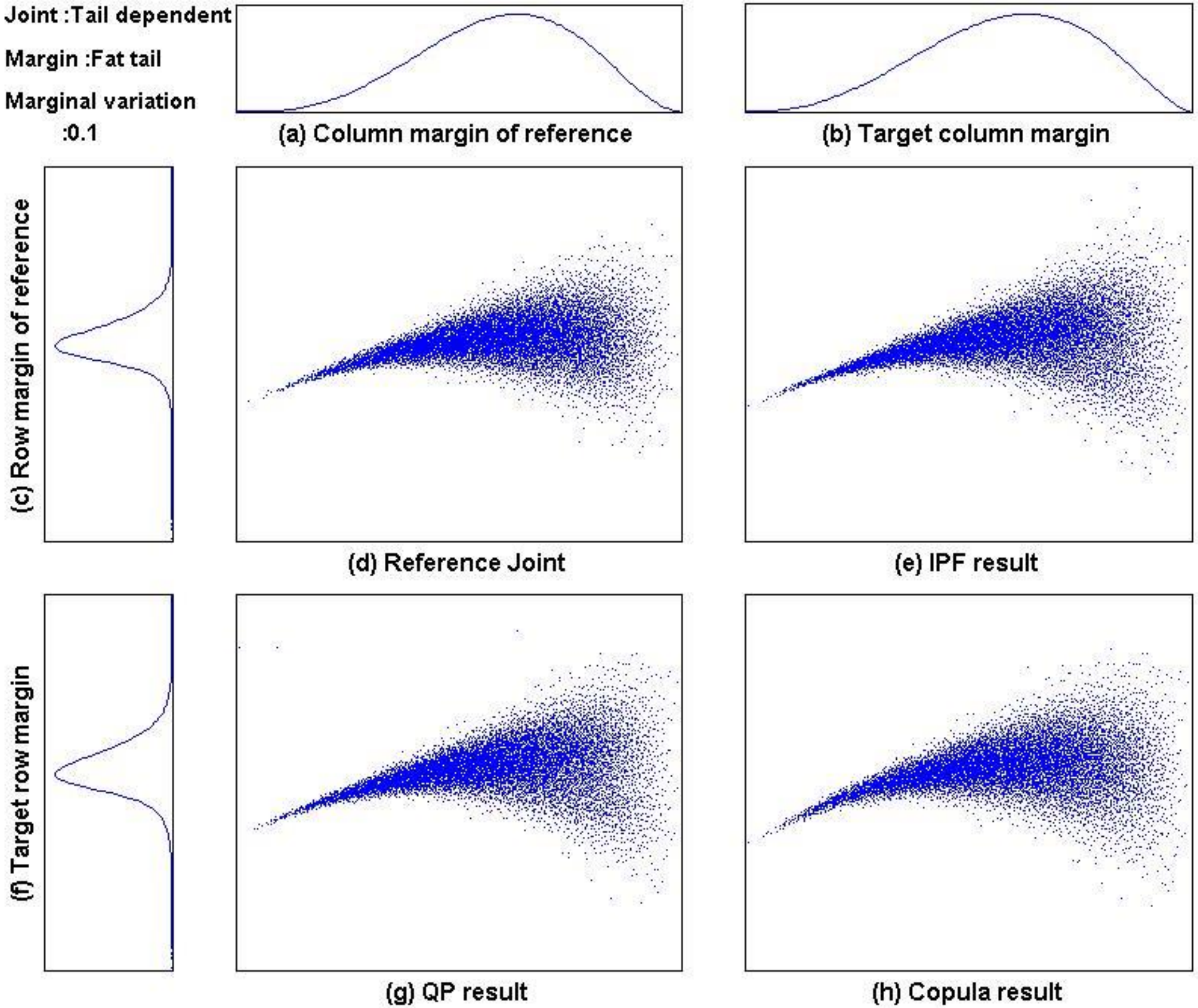

2-8-2. Reference joint distribution : Tail dependent, Target marginal type : Fat tail, Marginal variation : 0.2

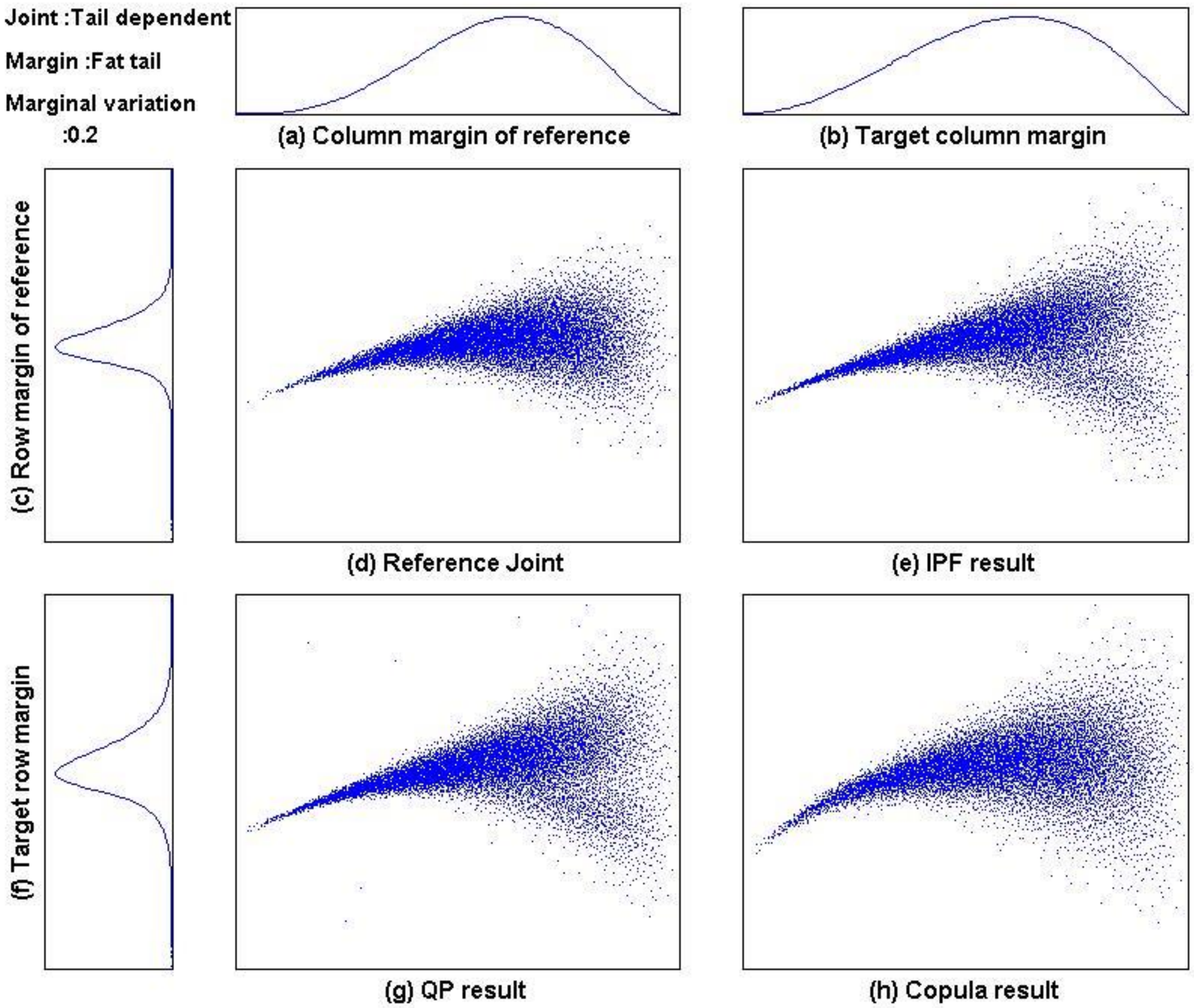

2-8-3. Reference joint distribution : Tail dependent, Target marginal type : Fat tail, Marginal variation : 0.3

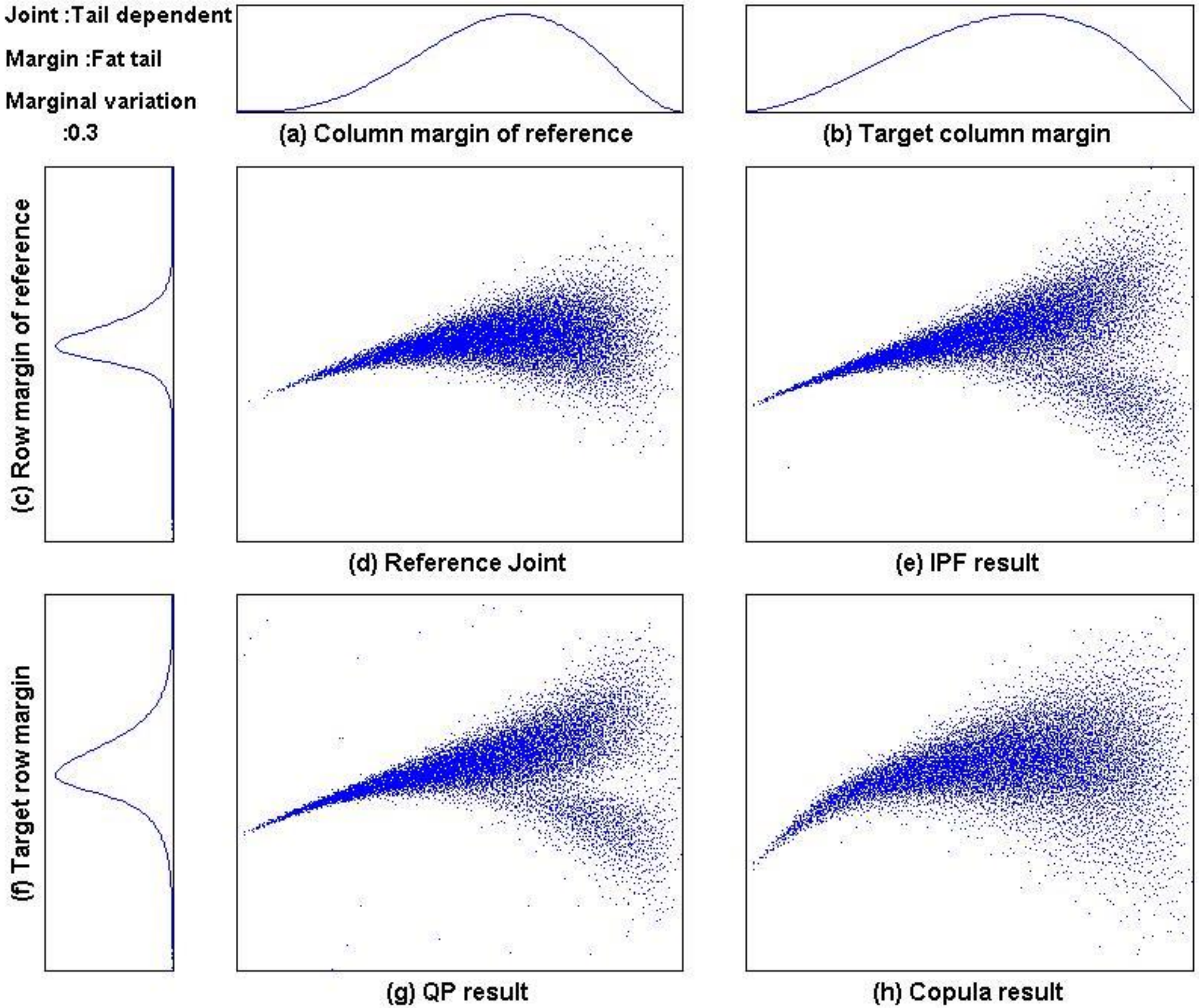

2-8-4. Reference joint distribution : Tail dependent, Target marginal type : Fat tail, Marginal variation : 0.4

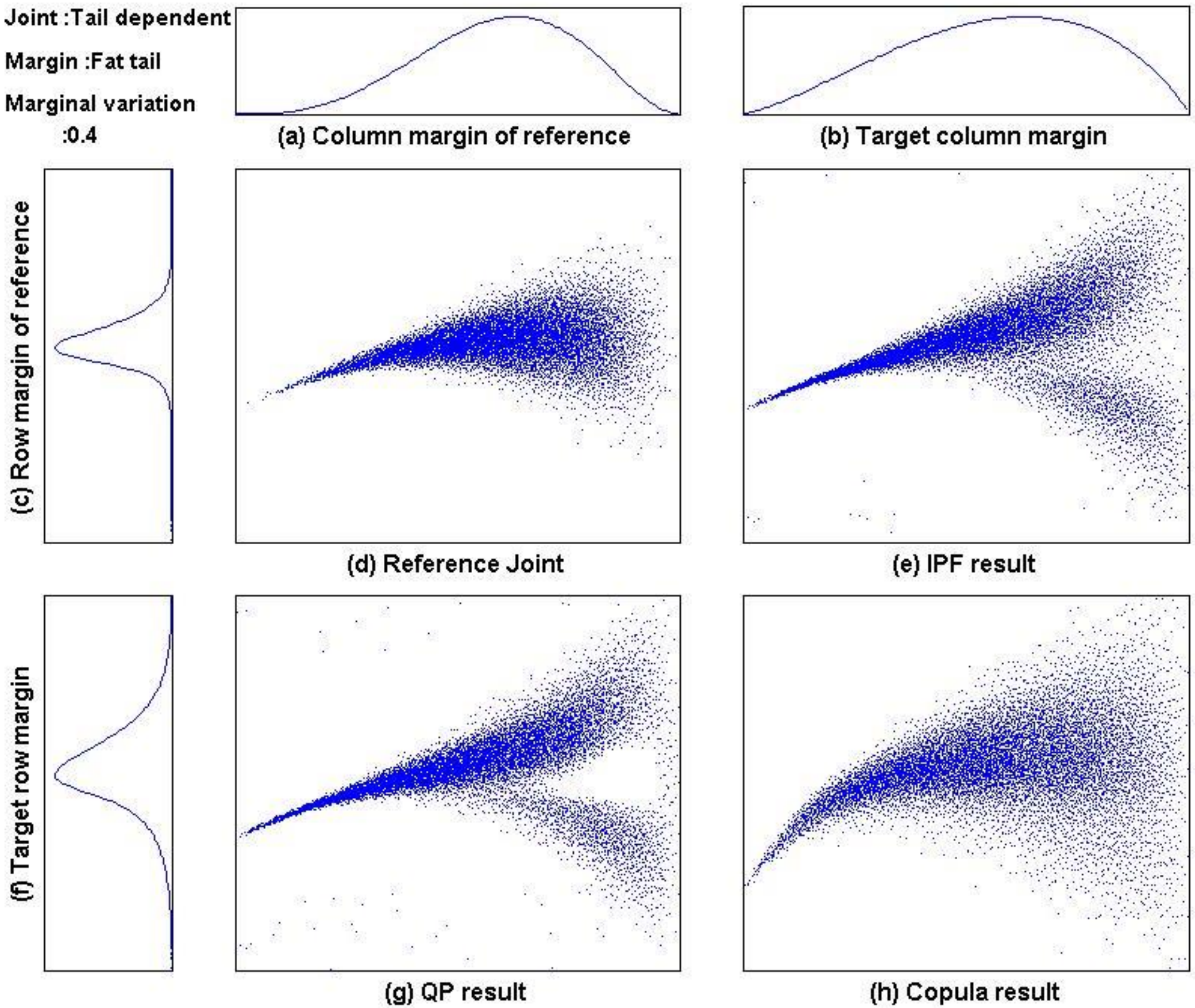

2-8-5. Reference joint distribution : Tail dependent, Target marginal type : Fat tail, Marginal variation : 0.5

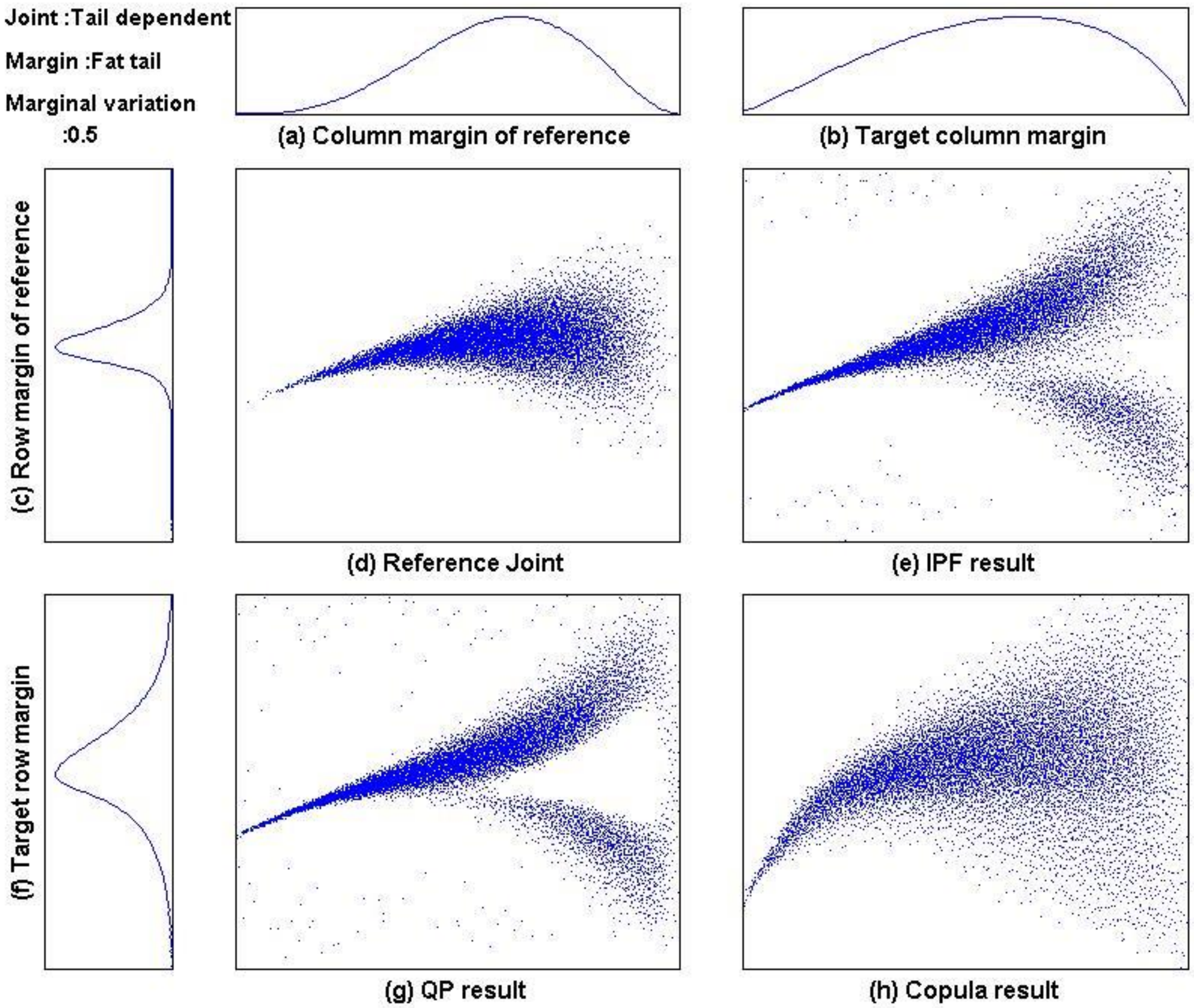

2-9-1. Reference joint distribution : Tail dependent, Target marginal type : Thin tail, Marginal variation : 0.1

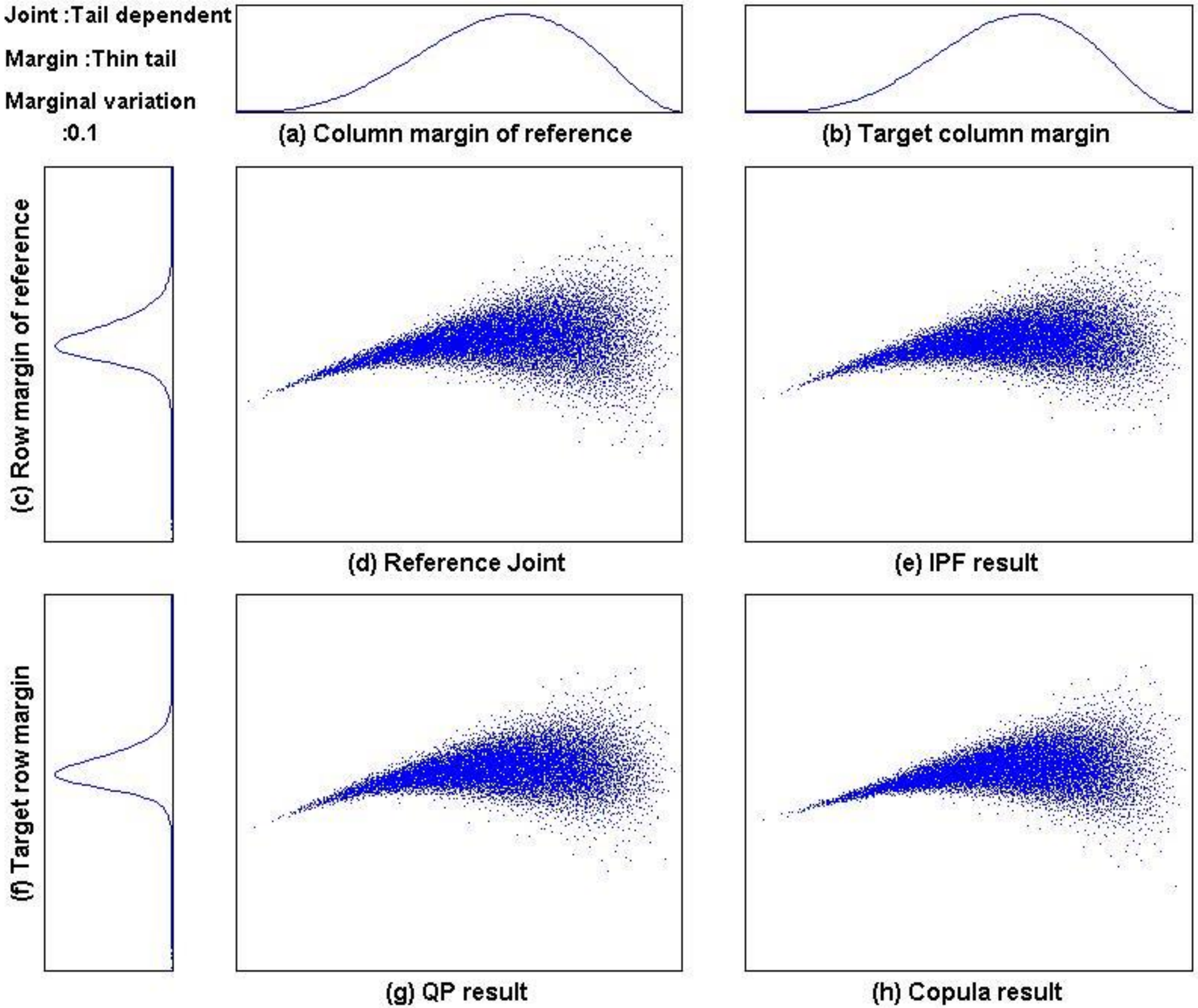

2-9-2. Reference joint distribution : Tail dependent, Target marginal type : Thin tail, Marginal variation : 0.2

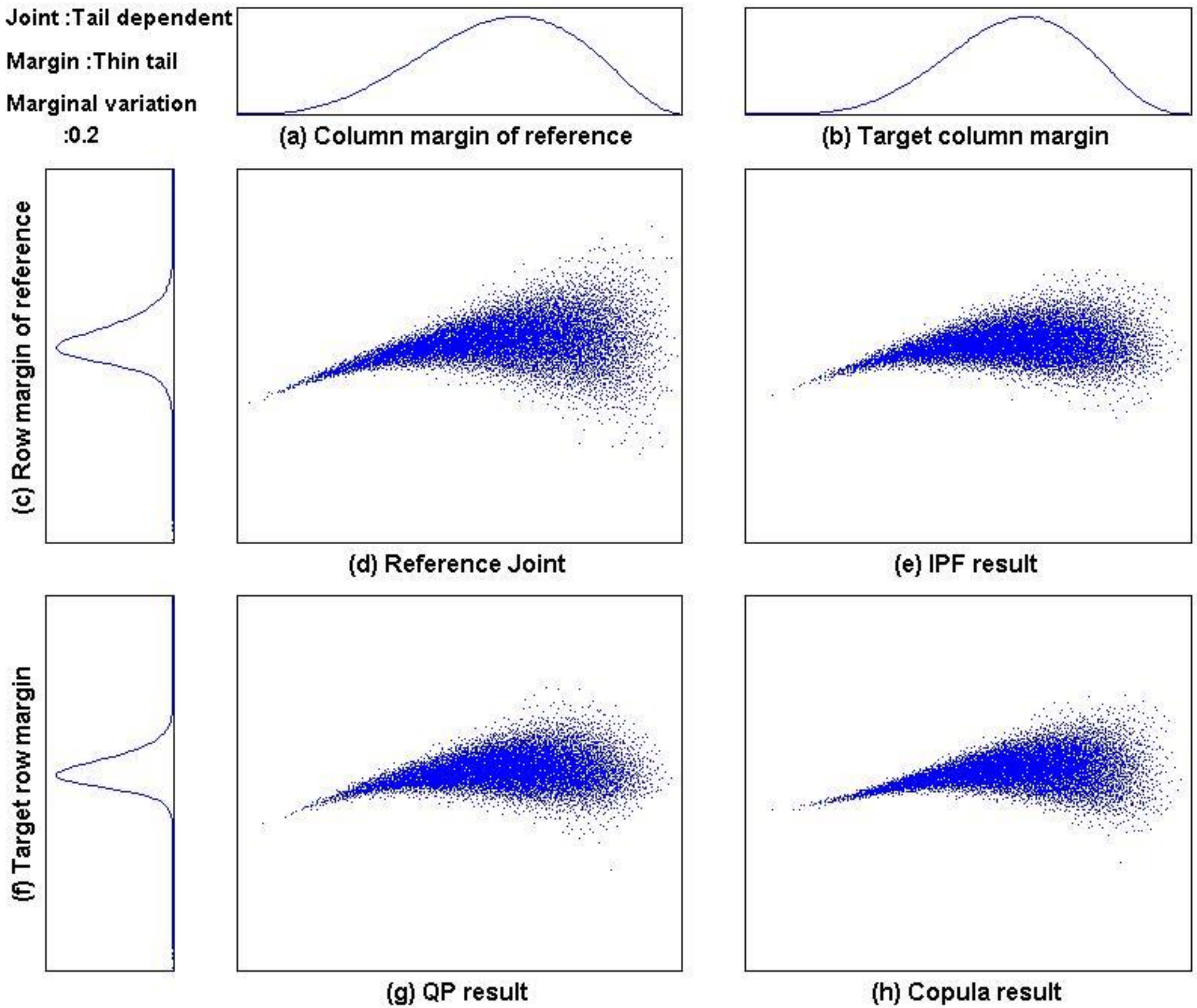

2-9-3. Reference joint distribution : Tail dependent, Target marginal type : Thin tail, Marginal variation : 0.3

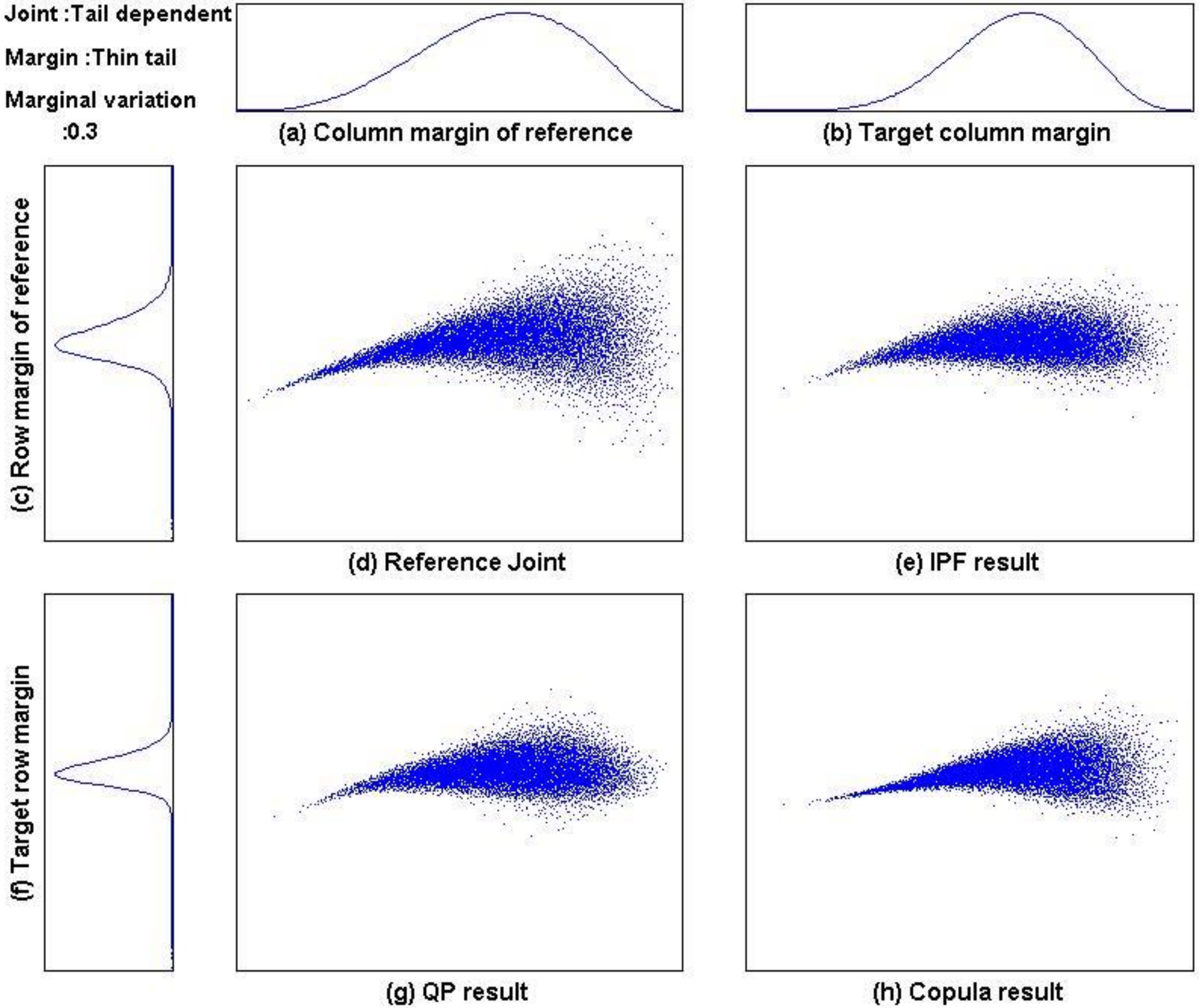

2-9-4. Reference joint distribution : Tail dependent, Target marginal type : Thin tail, Marginal variation : 0.4

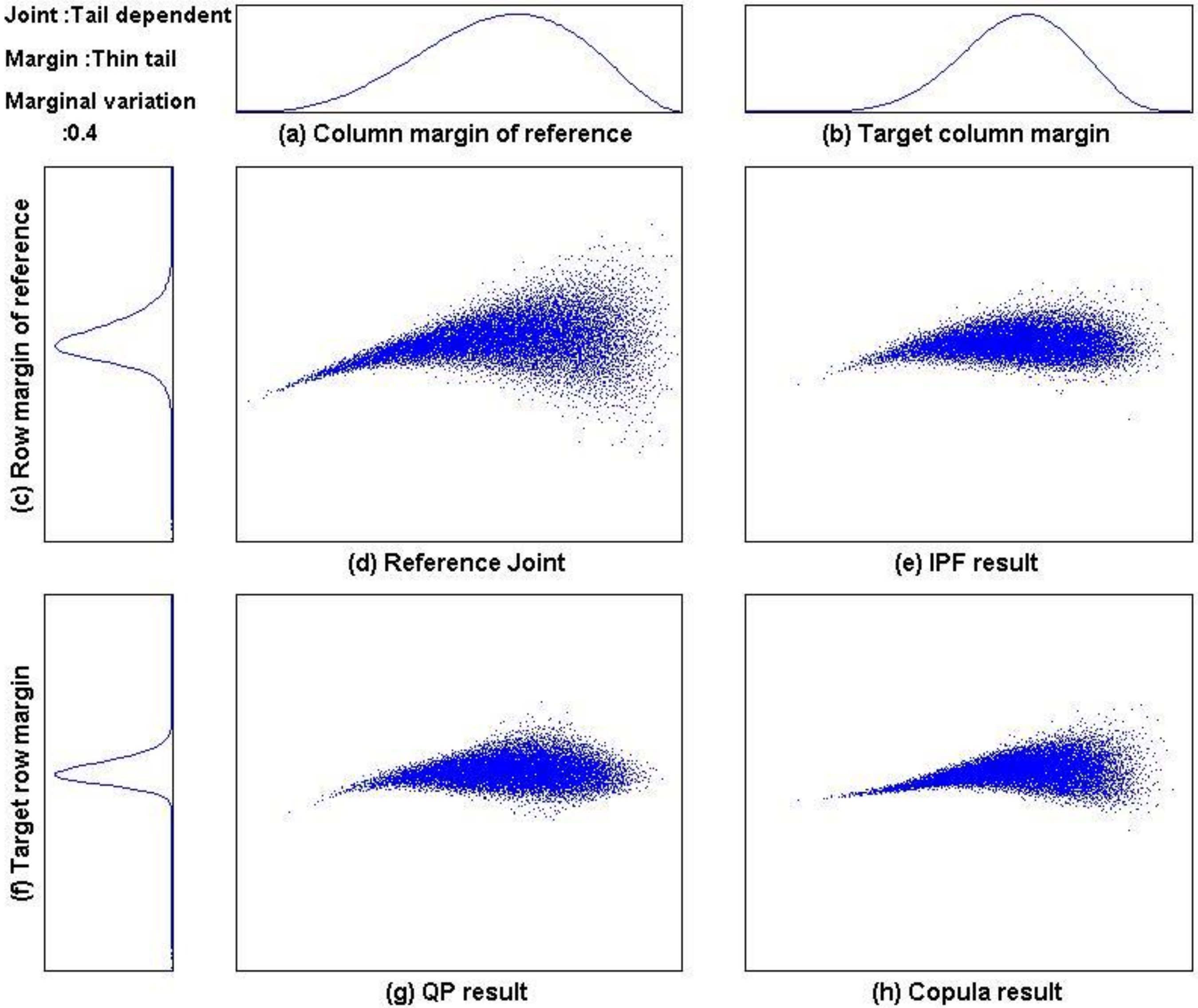

2-9-5. Reference joint distribution : Tail dependent, Target marginal type : Thin tail, Marginal variation : 0.5

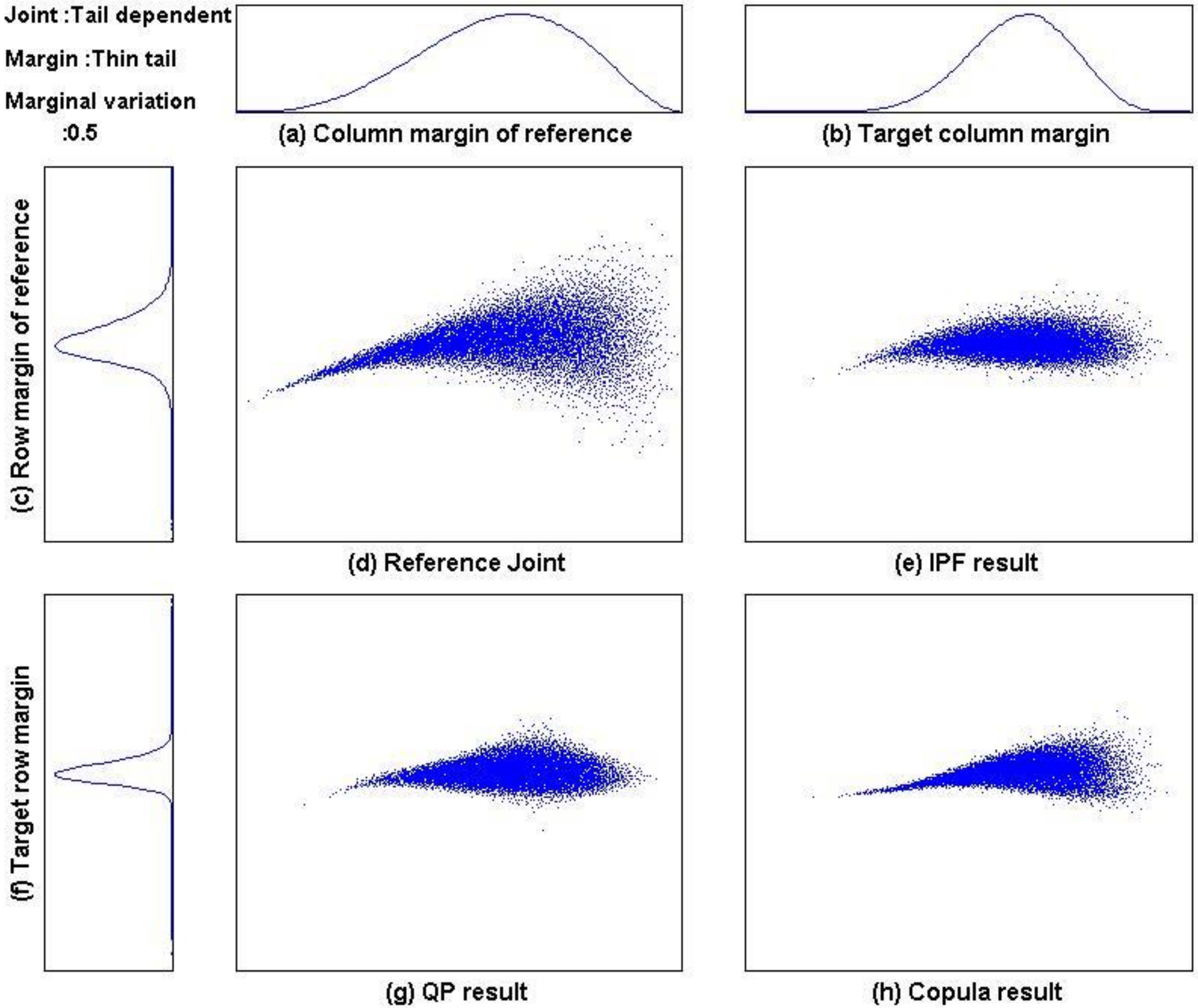

2-10-1. Reference joint distribution : U-shape, Target marginal type : Skew, Marginal variation : 0.11

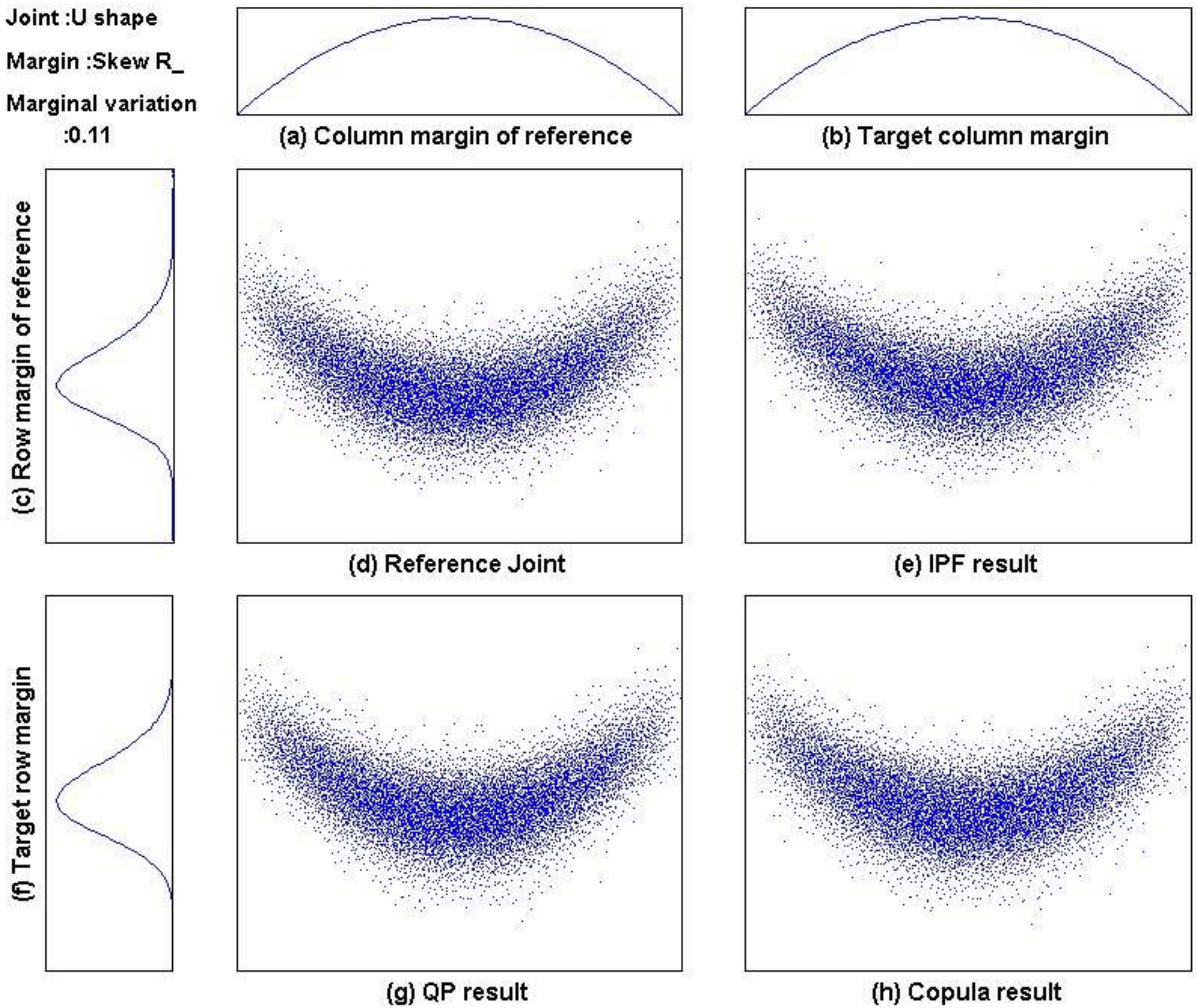

2-10-2. Reference joint distribution : U-shape, Target marginal type : Skew, Marginal variation : 0.22

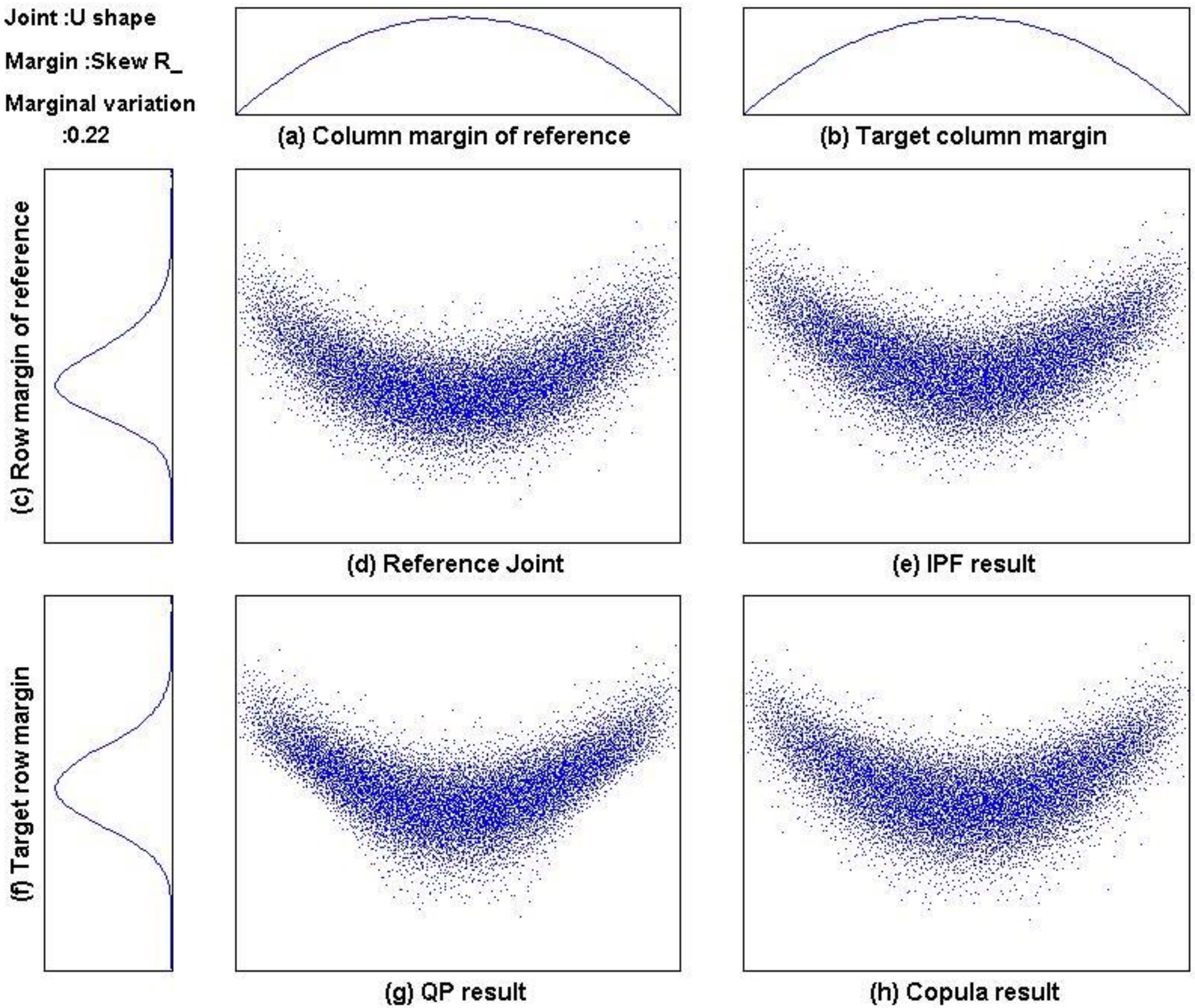

2-10-3. Reference joint distribution : U-shape, Target marginal type : Skew, Marginal variation : 0.32

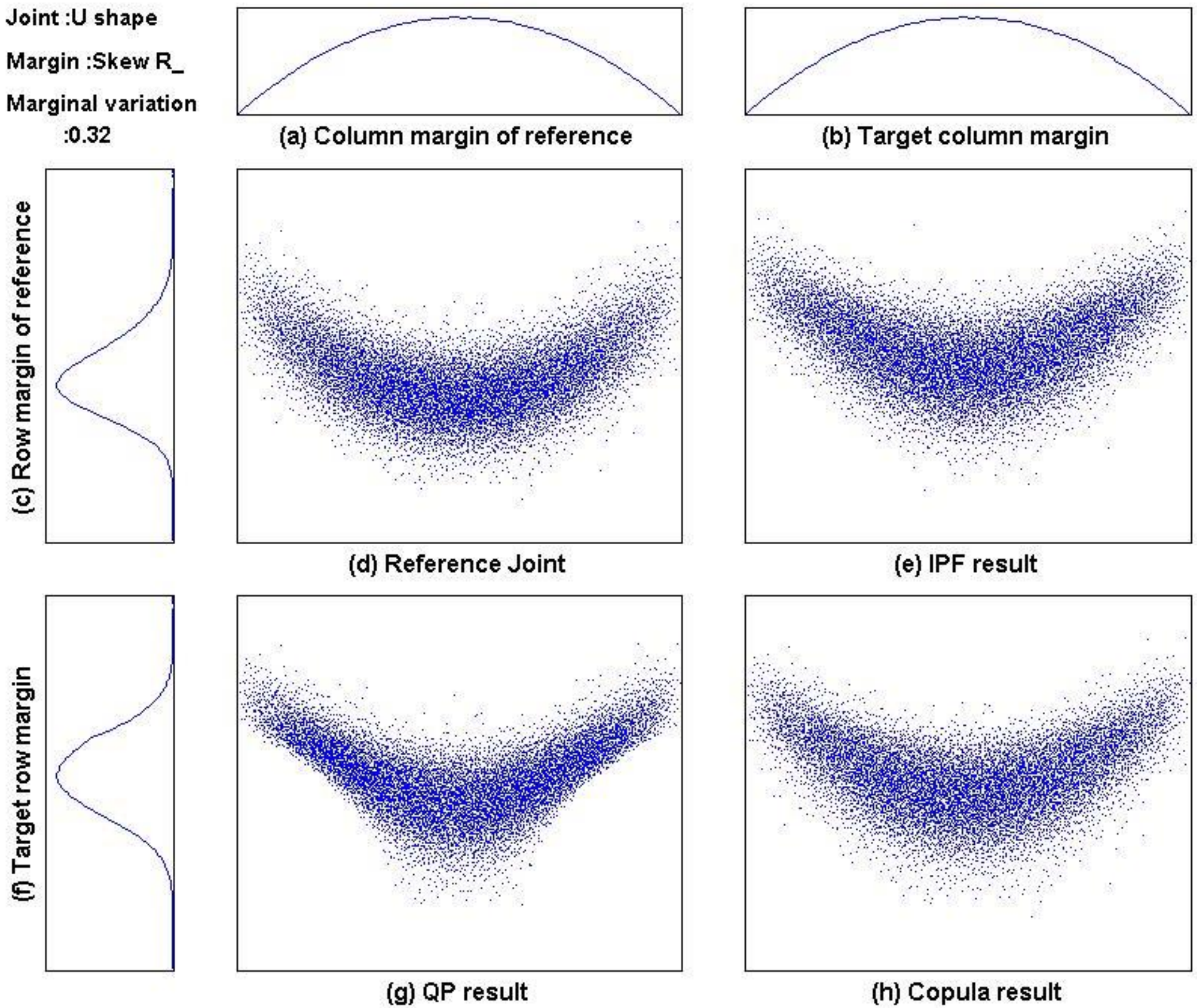

2-10-4. Reference joint distribution : U-shape, Target marginal type : Skew, Marginal variation : 0.42

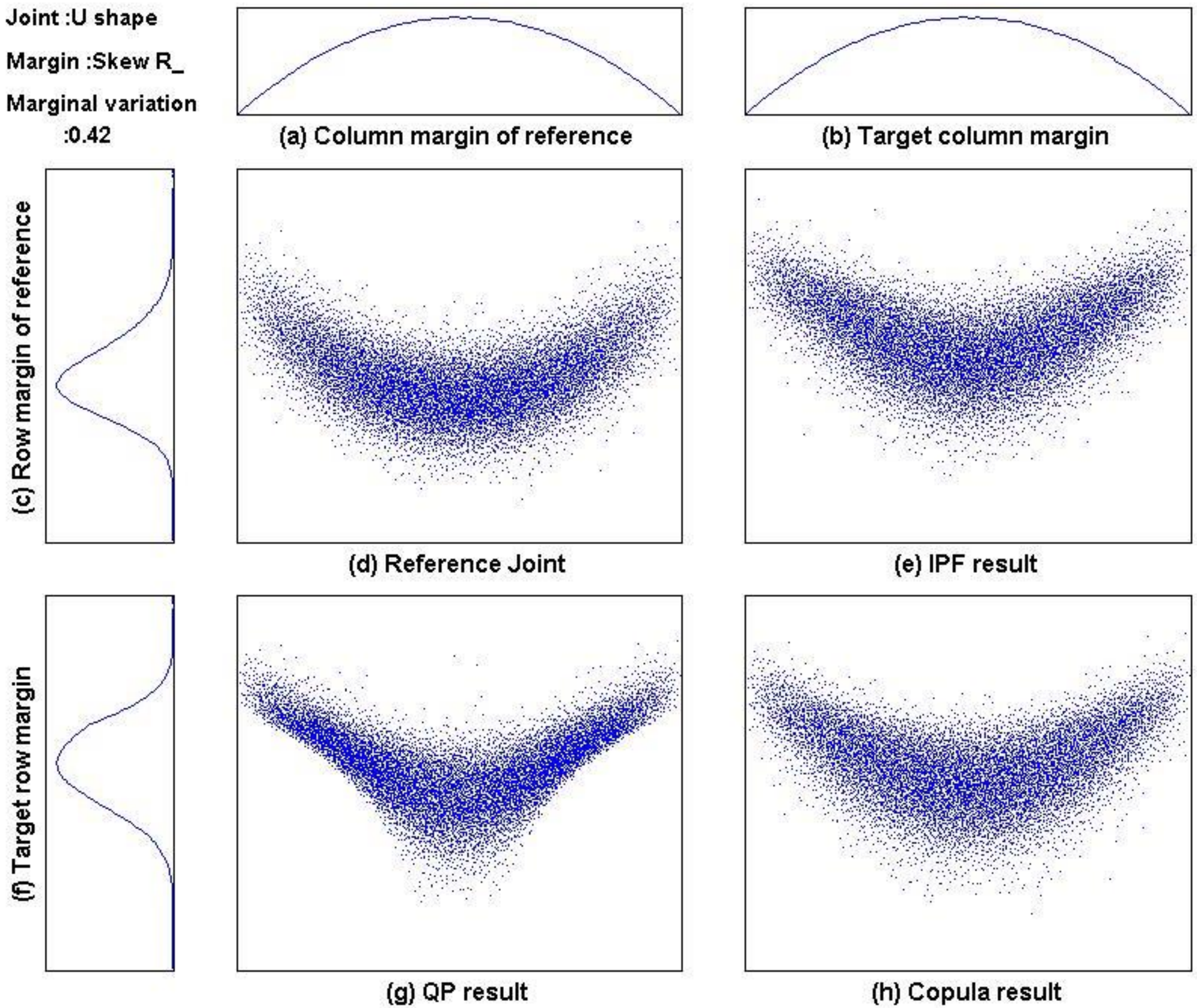

2-10-5. Reference joint distribution : U-shape, Target marginal type : Skew, Marginal variation : 0.52

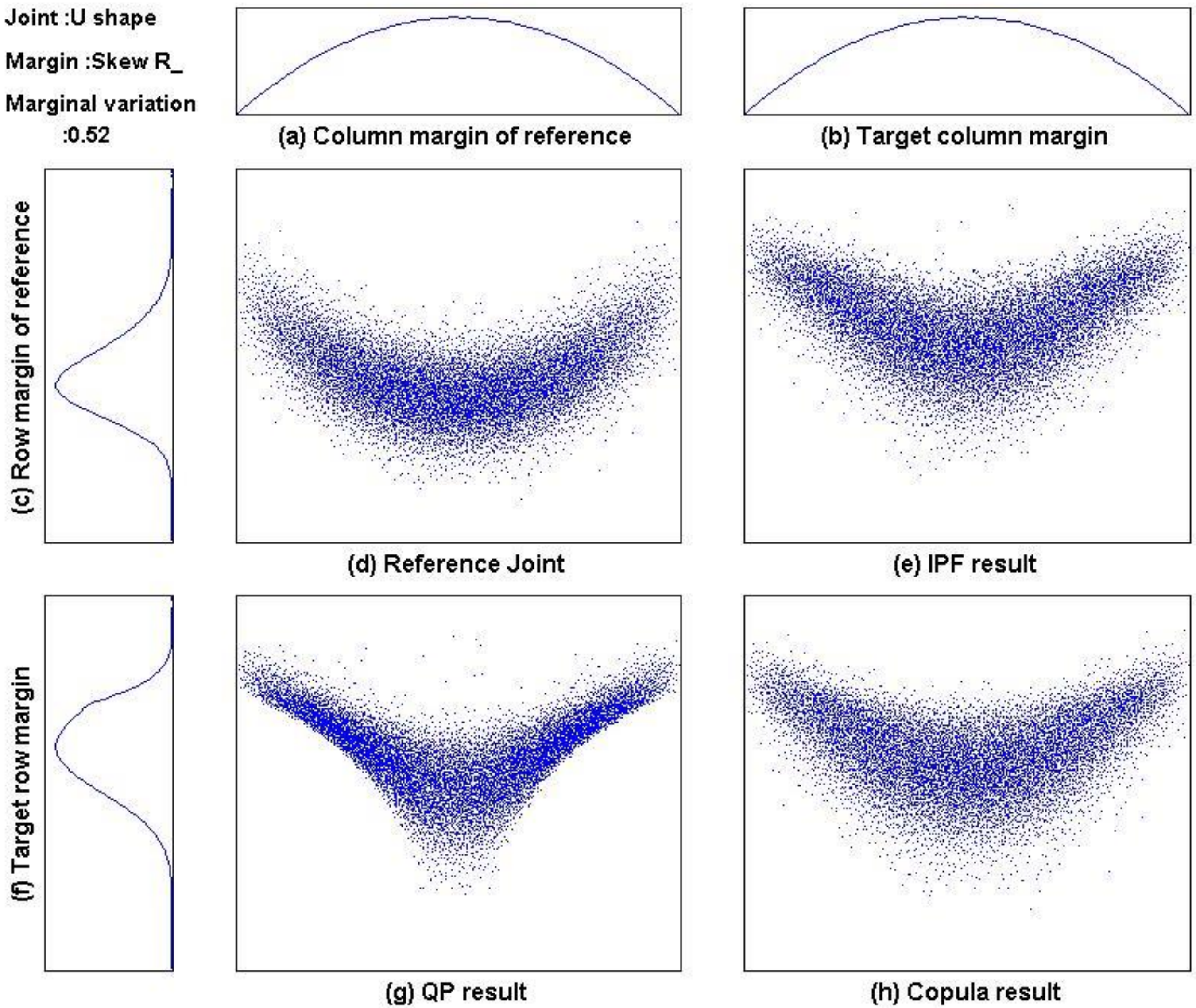

2-11-1. Reference joint distribution : U-shape, Target marginal type : Fat tail, Marginal variation : 0.1

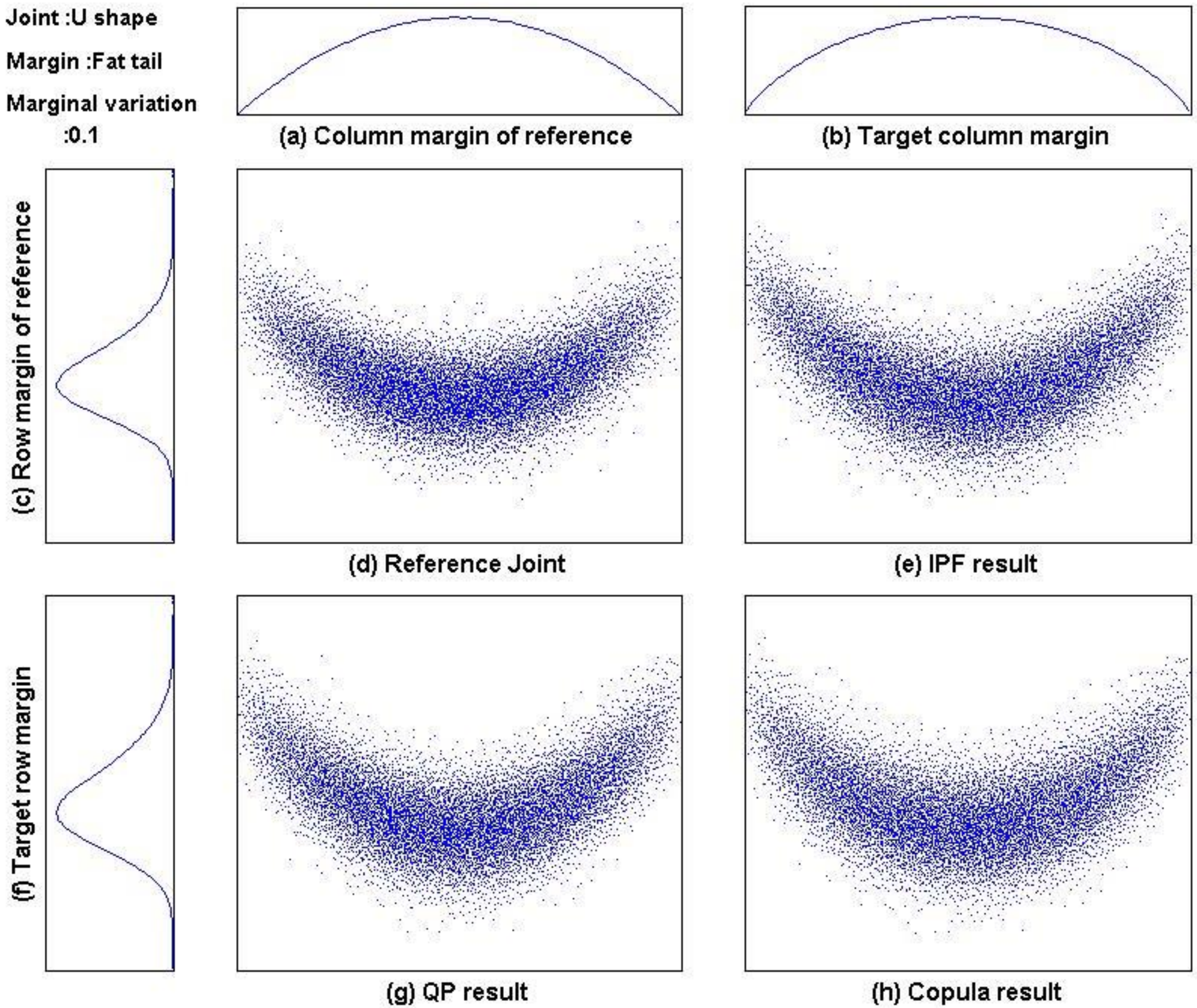

2-11-2. Reference joint distribution : U-shape, Target marginal type : Fat tail, Marginal variation : 0.2

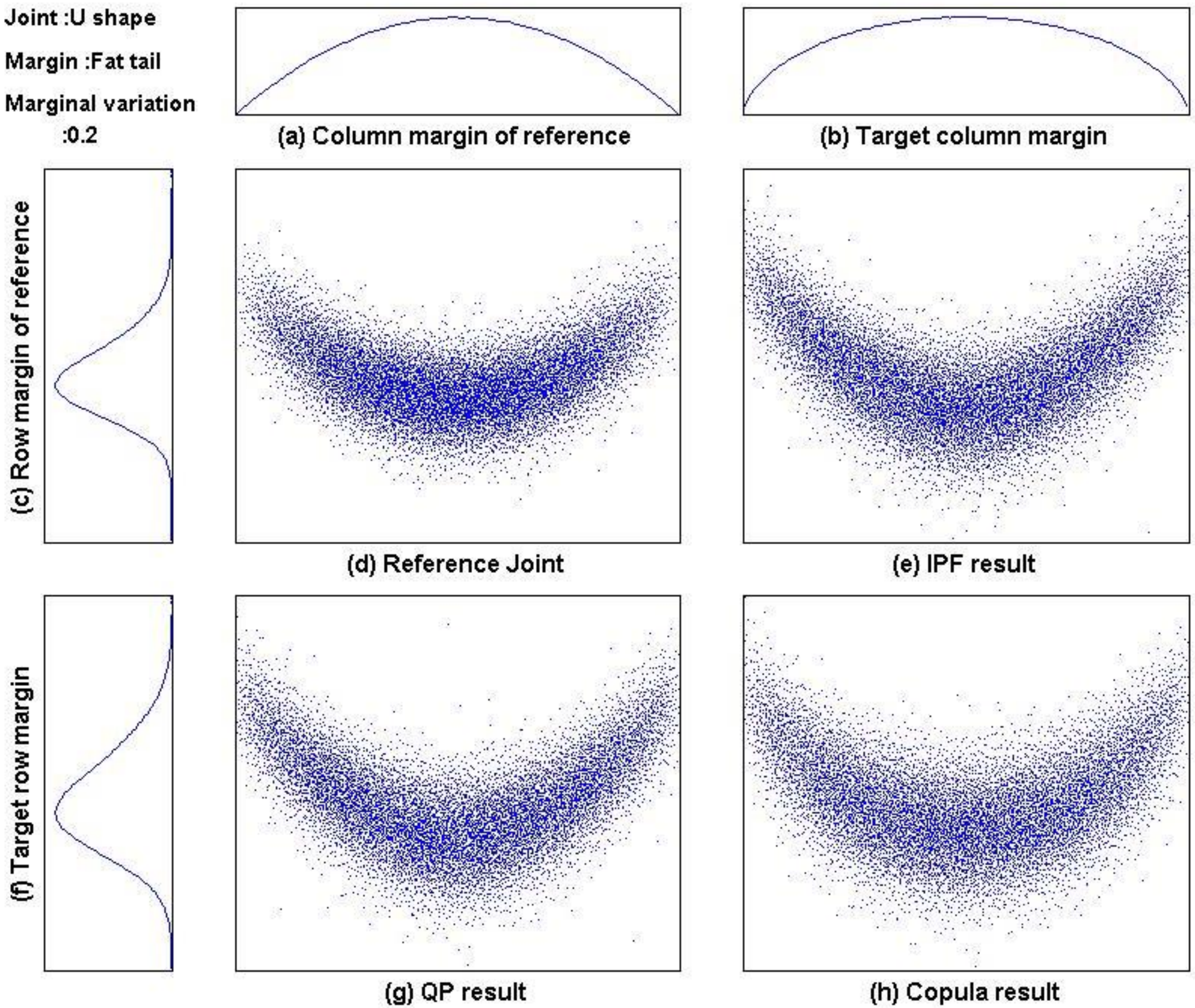

2-11-3. Reference joint distribution : U-shape, Target marginal type : Fat tail, Marginal variation : 0.3

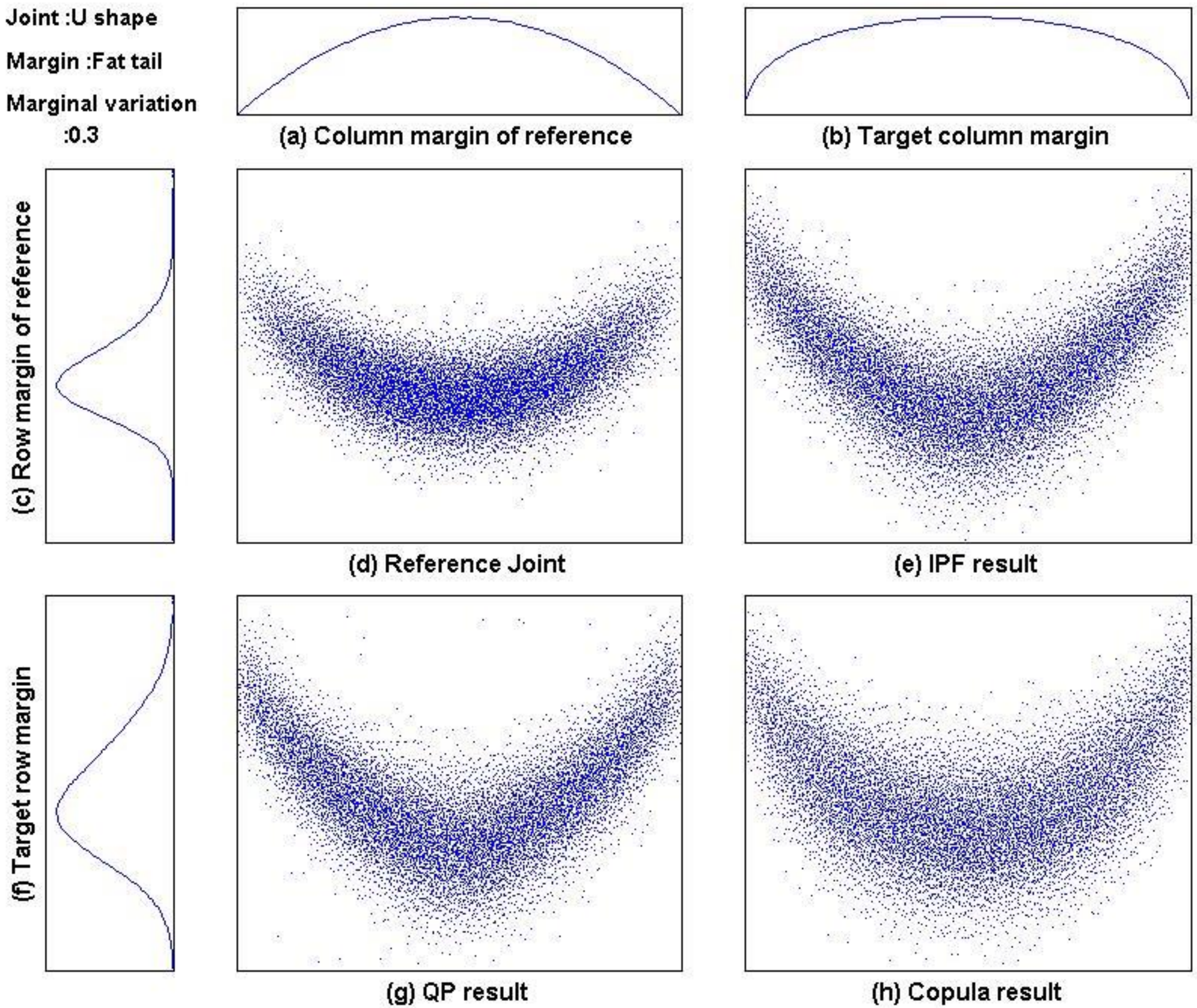

2-11-4. Reference joint distribution : U-shape, Target marginal type : Fat tail, Marginal variation : 0.4

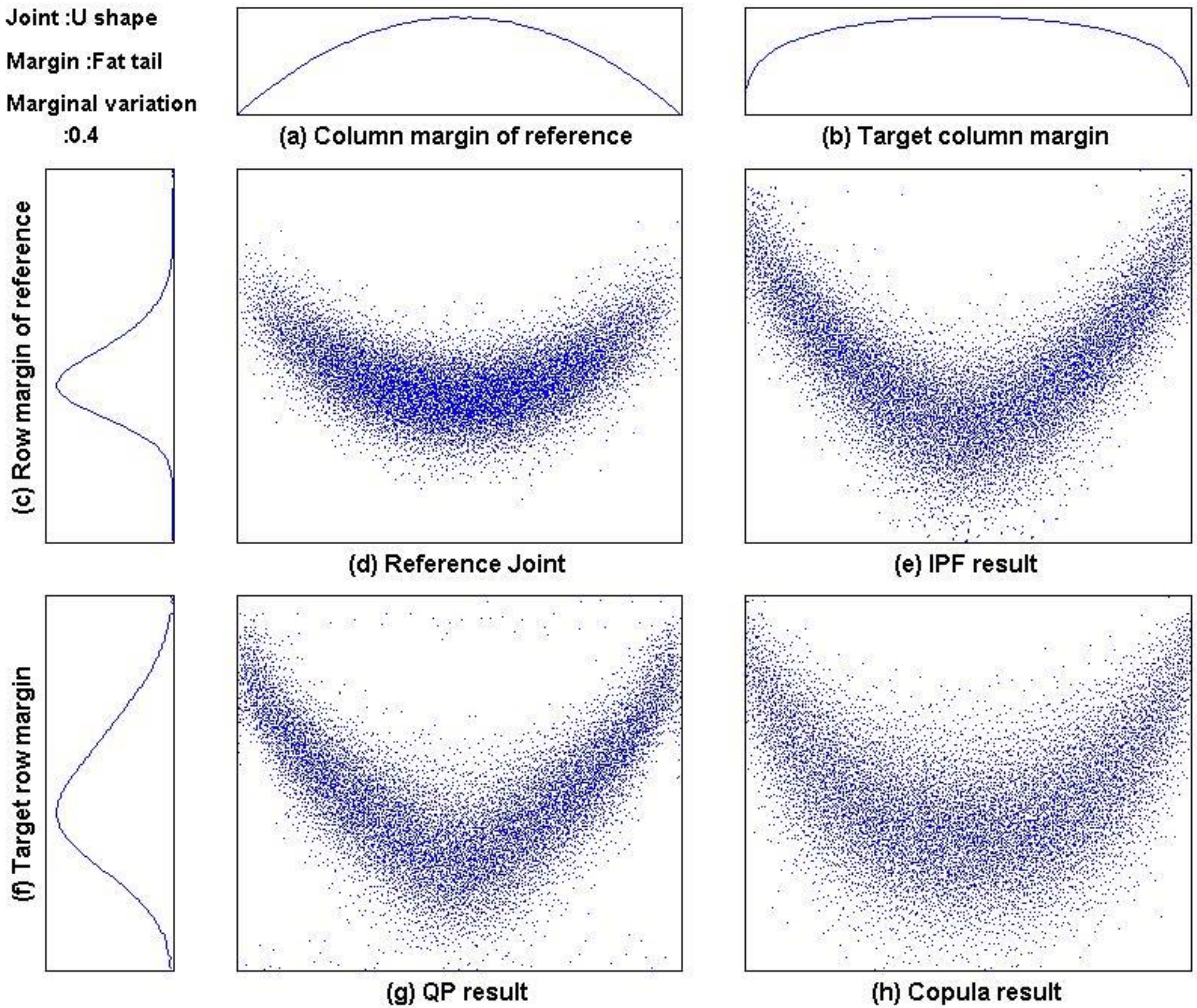

2-11-5. Reference joint distribution : U-shape, Target marginal type : Fat tail, Marginal variation : 0.5

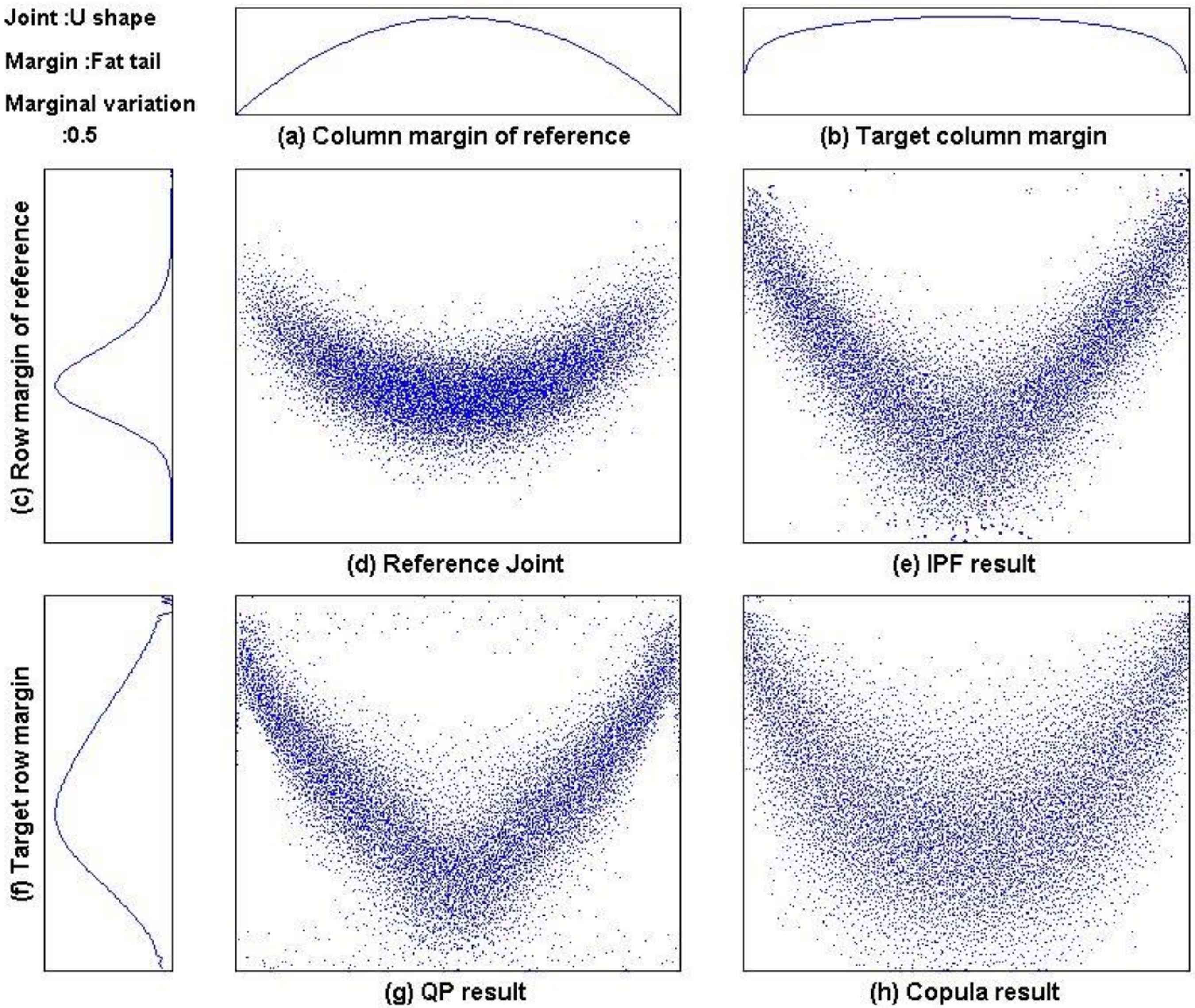

2-11-6. Reference joint distribution : U-shape, Target marginal type : Fat tail, Marginal variation : 0.6

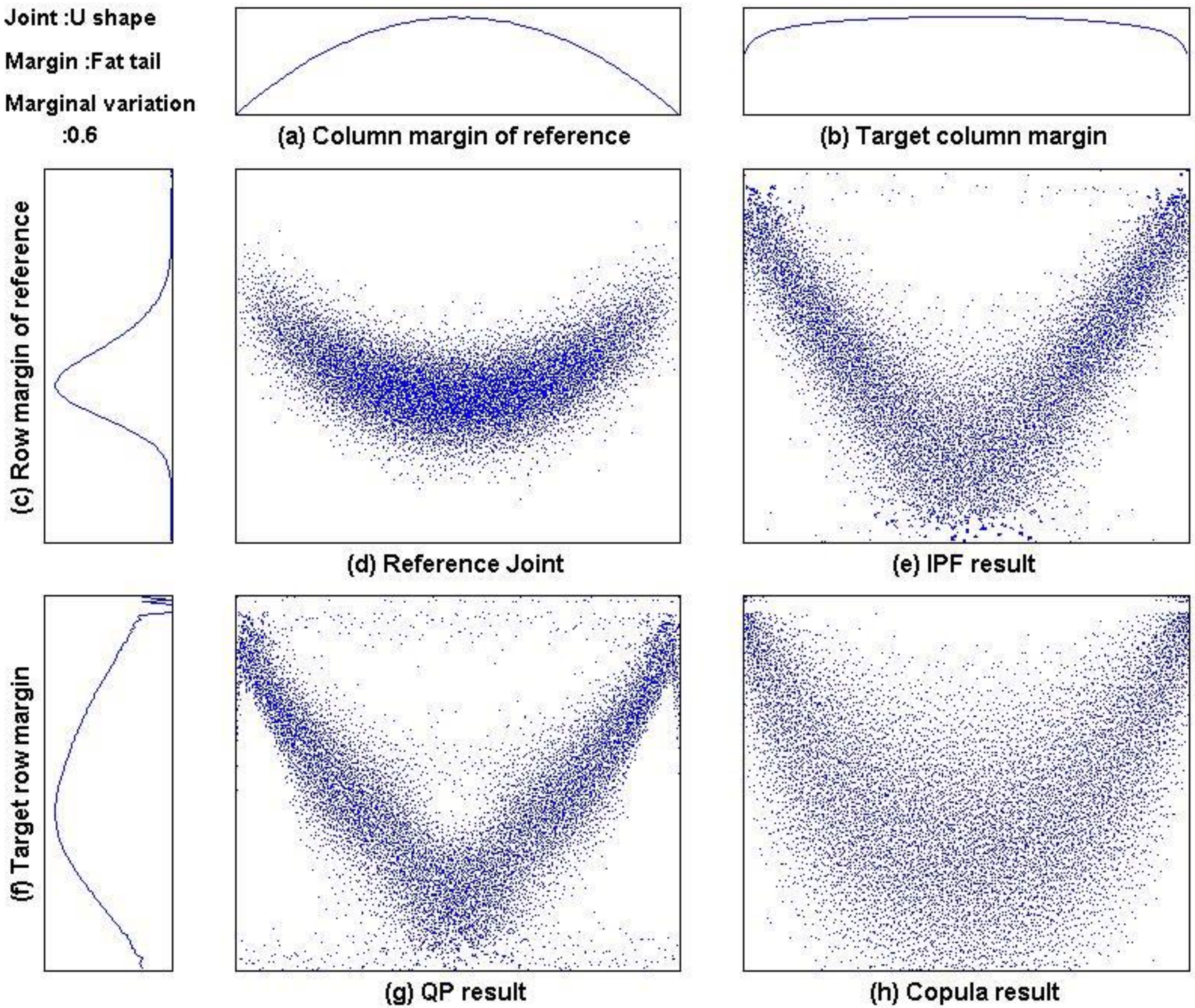

2-12-1. Reference joint distribution : U-shape, Target marginal type : Thin tail, Marginal variation : 0.2

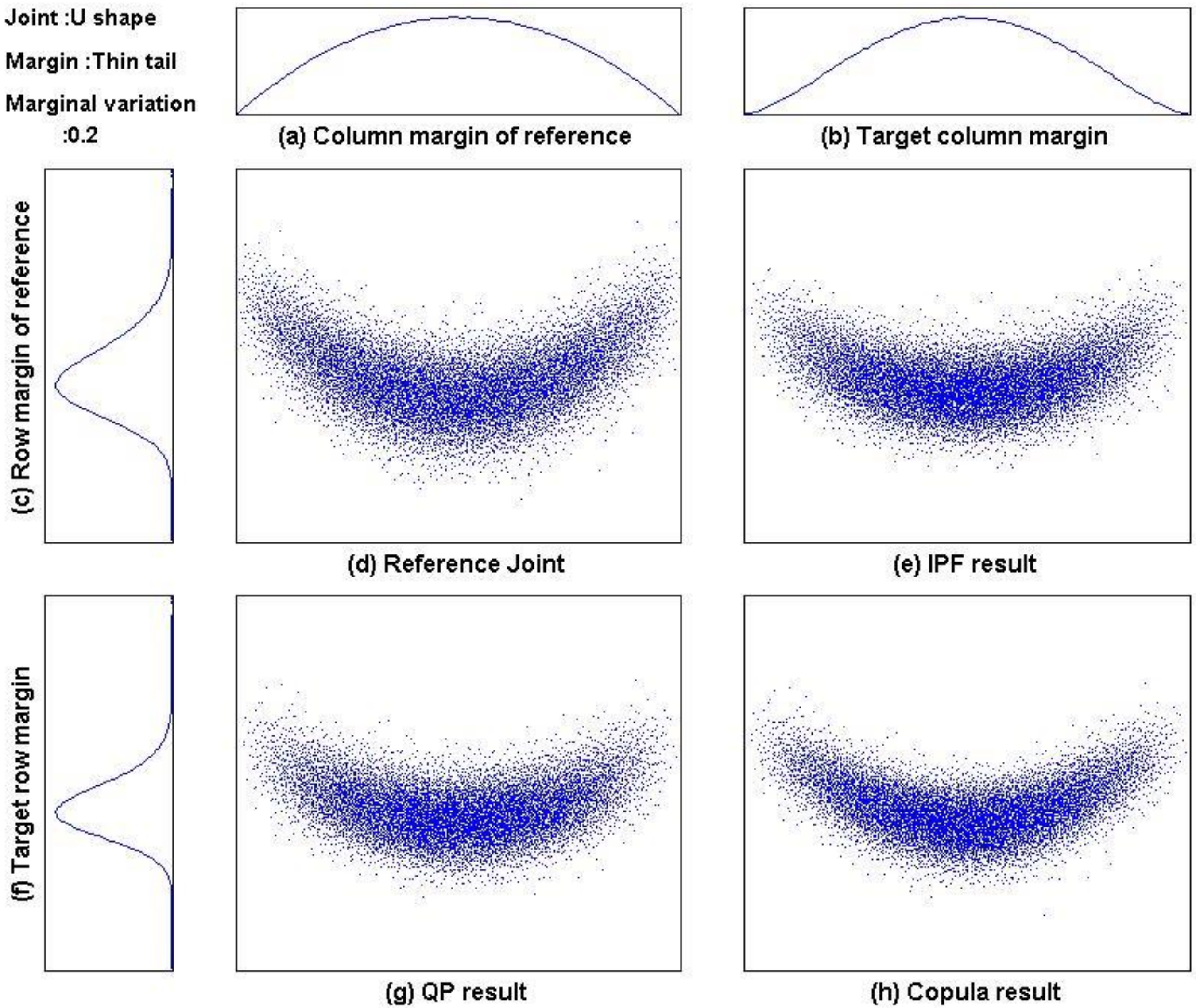

2-12-2. Reference joint distribution : U-shape, Target marginal type : Thin tail, Marginal variation : 0.4

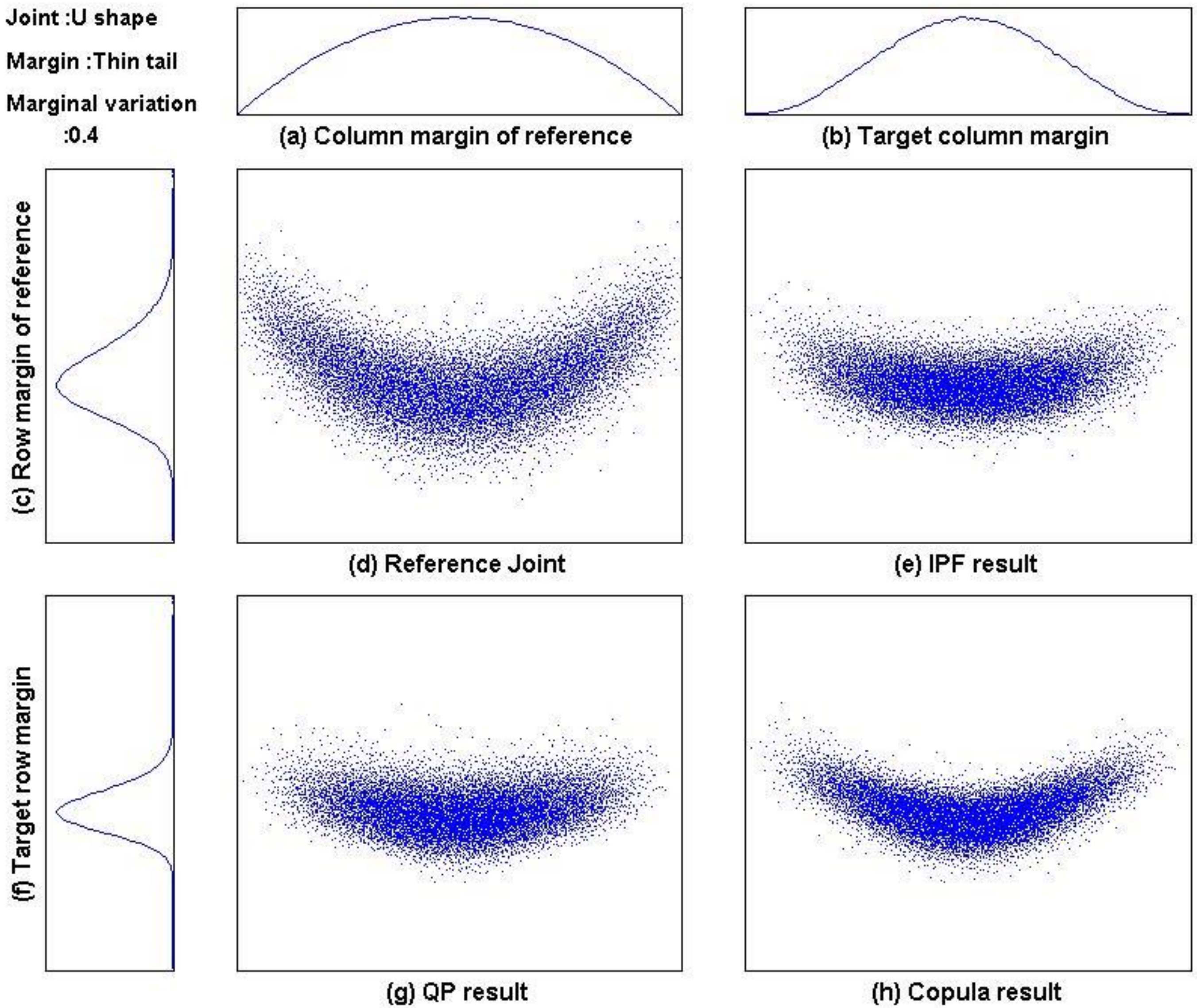

2-12-3. Reference joint distribution : U-shape, Target marginal type : Thin tail, Marginal variation : 0.6

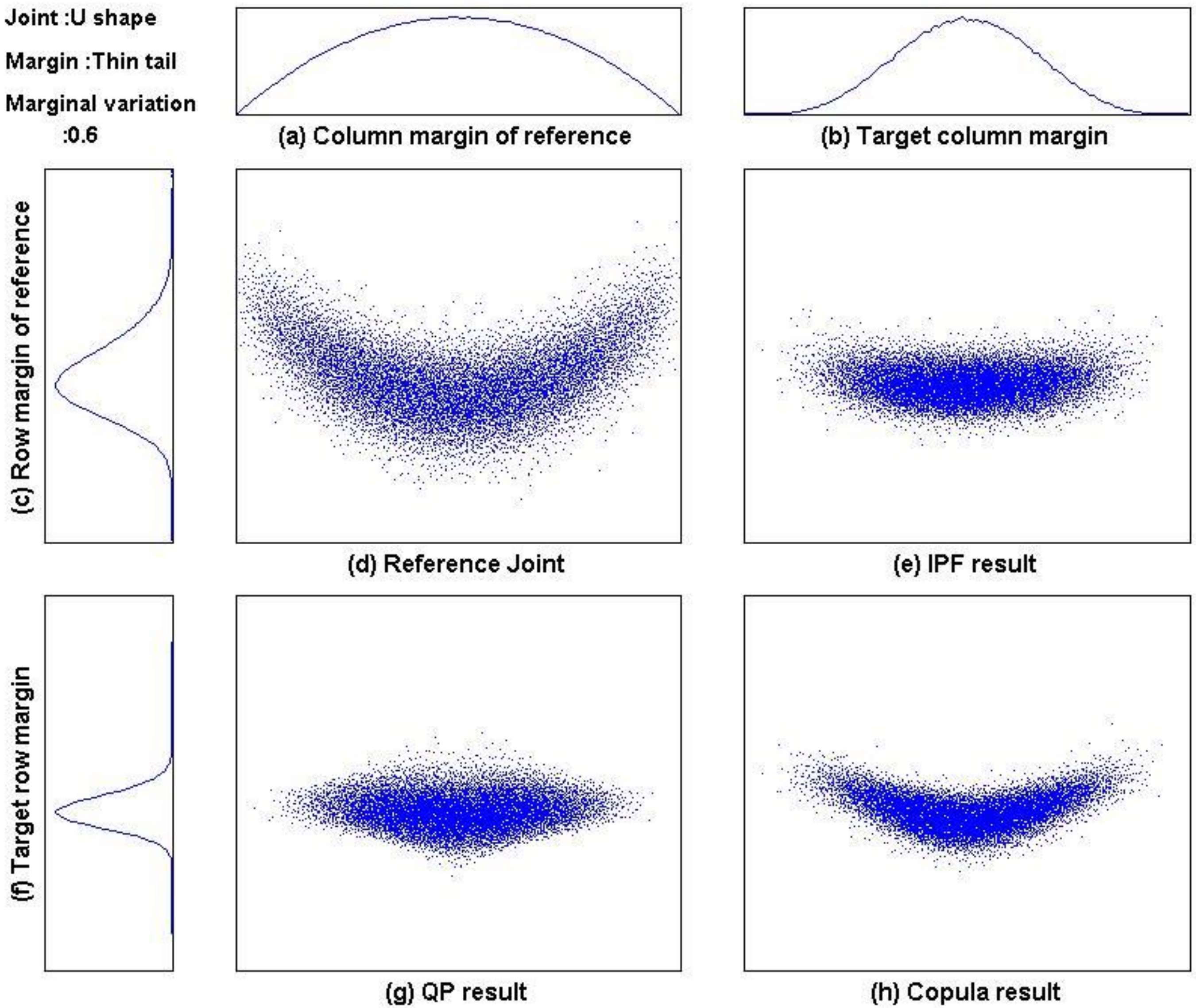

2-12-4. Reference joint distribution : U-shape, Target marginal type : Thin tail, Marginal variation : 0.8

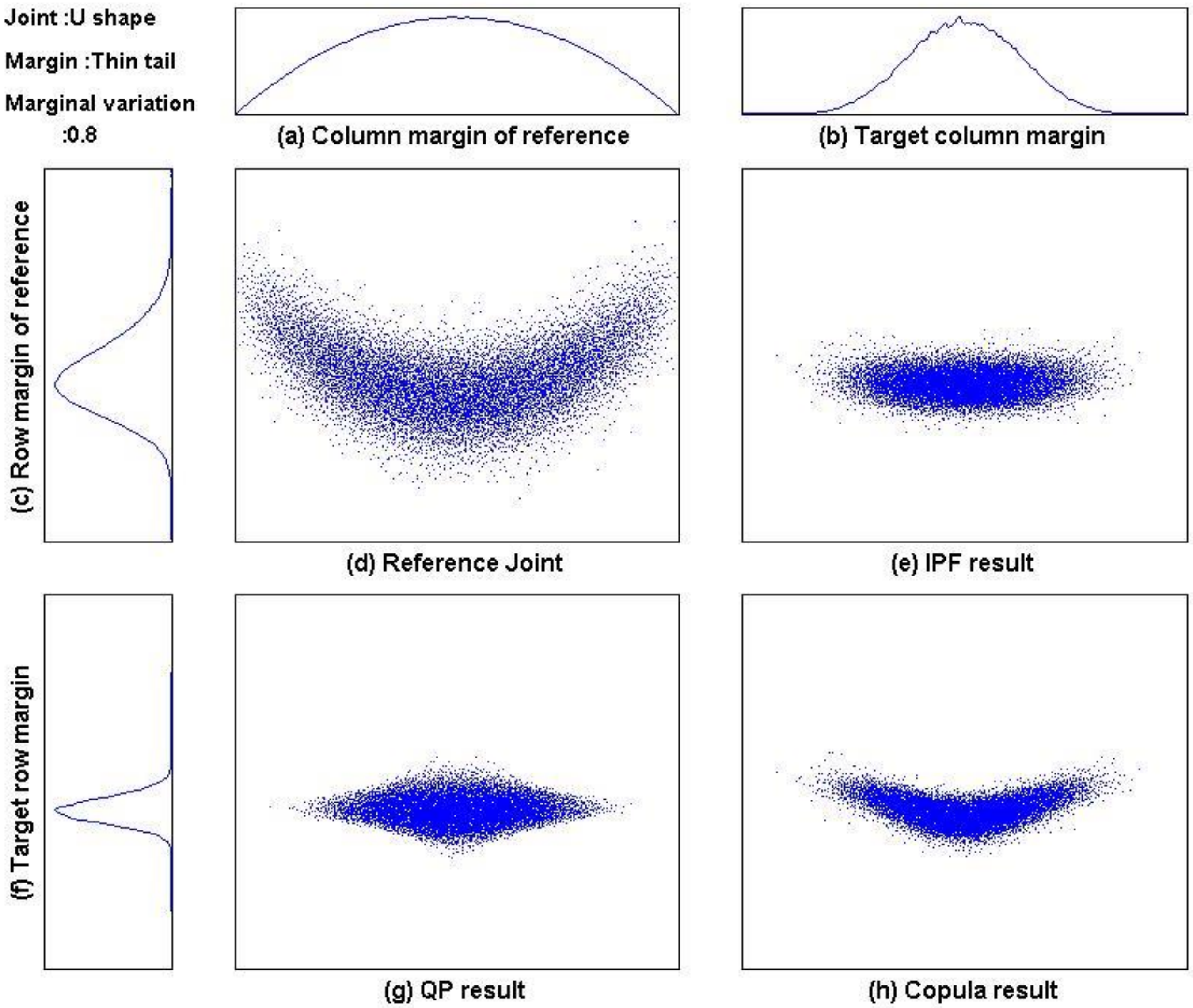

2-13-1. Reference joint distribution : Circle, Target marginal type : Skew, Marginal variation : 0.11

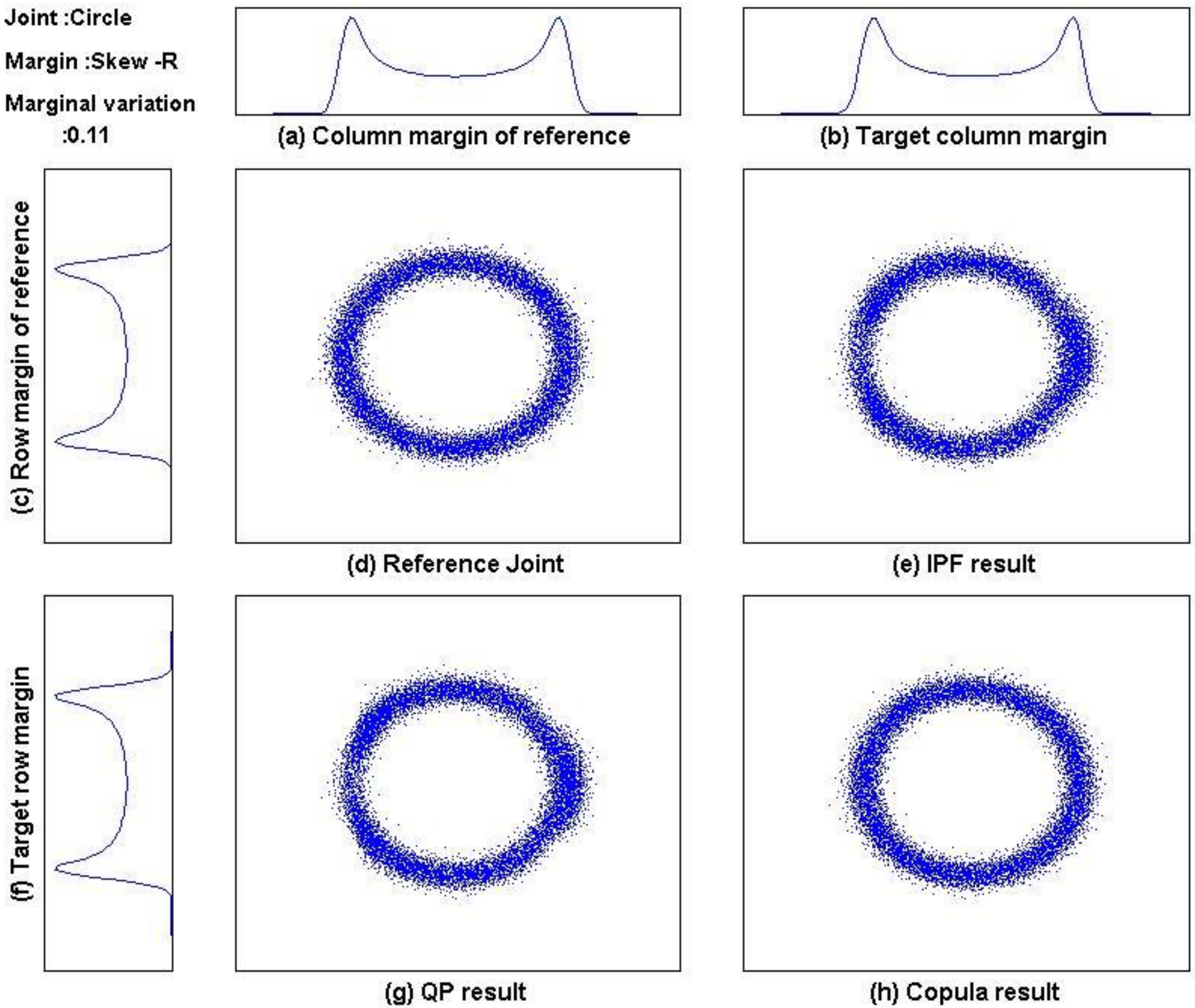

2-13-2. Reference joint distribution : Circle, Target marginal type : Skew, Marginal variation : 0.21

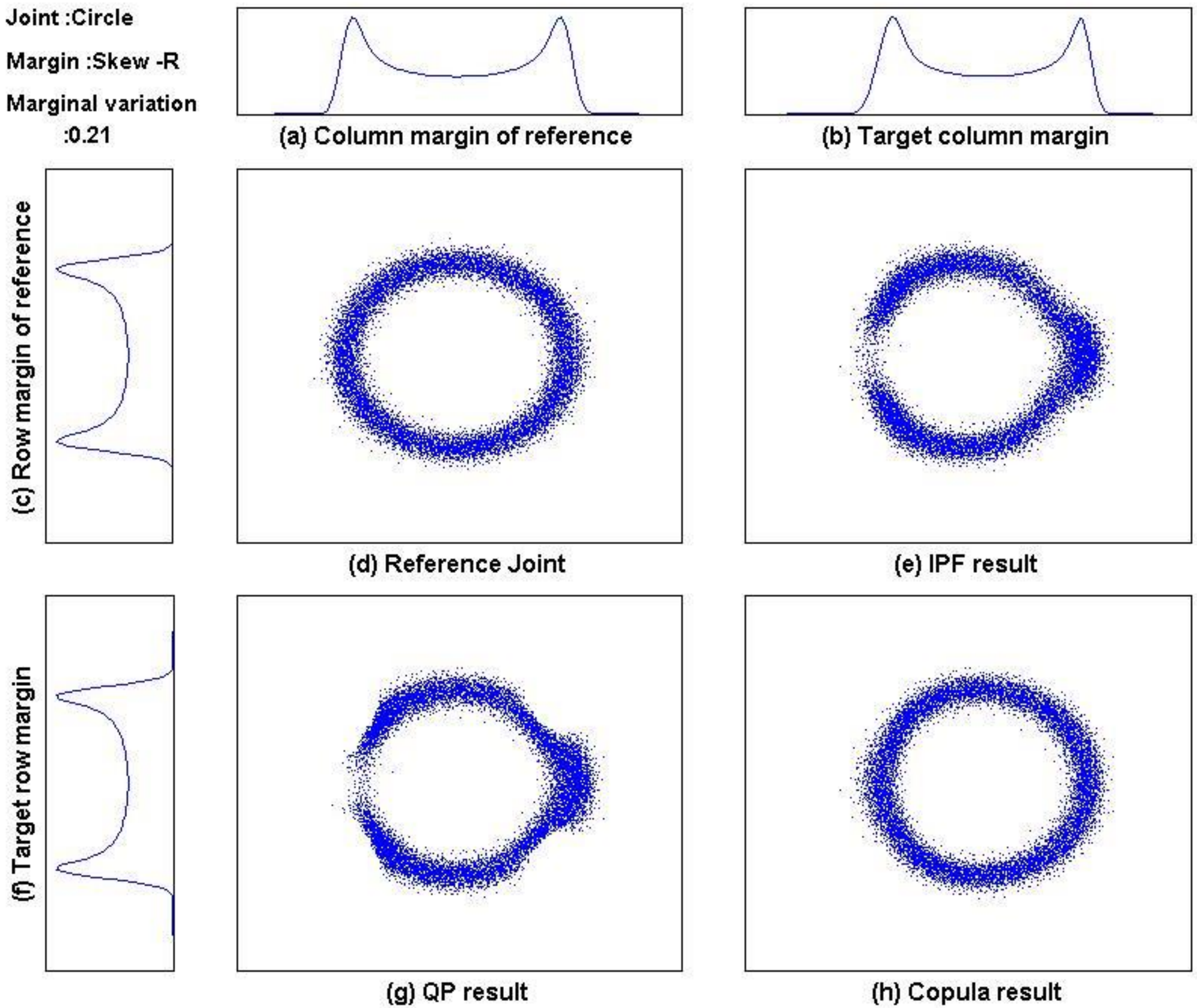

2-13-3. Reference joint distribution : Circle, Target marginal type : Skew, Marginal variation : 0.31

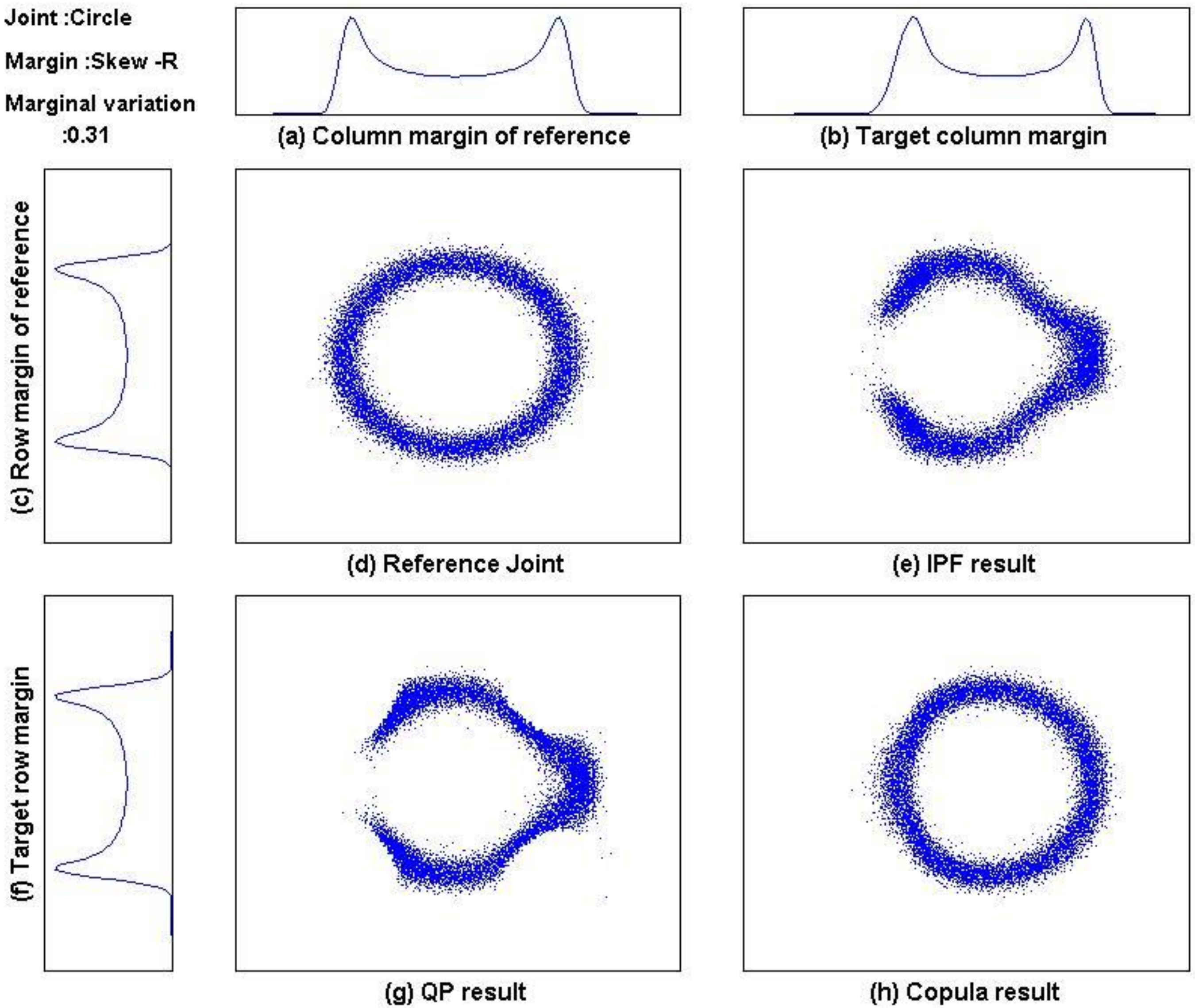

2-13-4. Reference joint distribution : Circle, Target marginal type : Skew, Marginal variation : 0.41

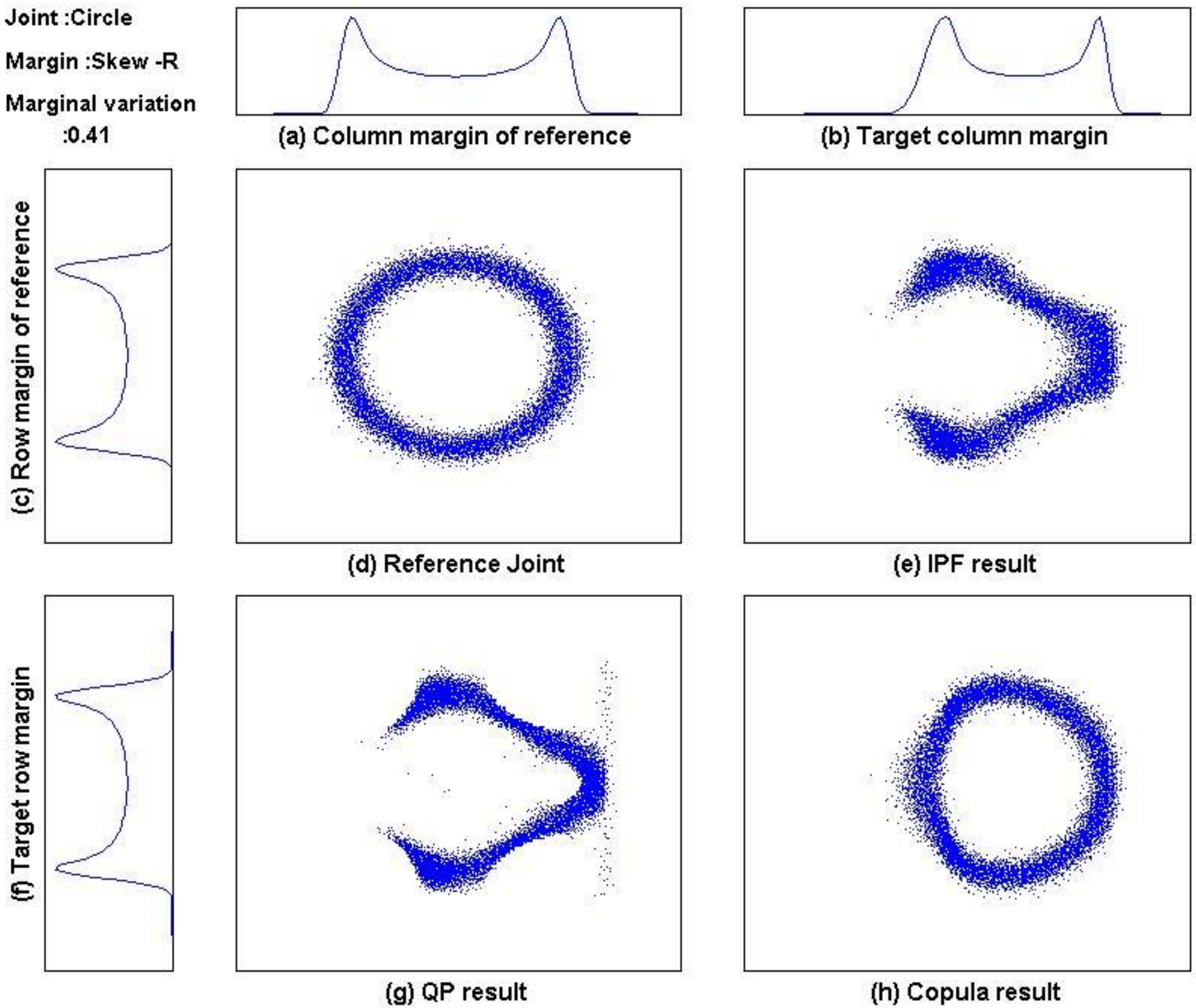

2-13-5. Reference joint distribution : Circle, Target marginal type : Skew, Marginal variation : 0.51

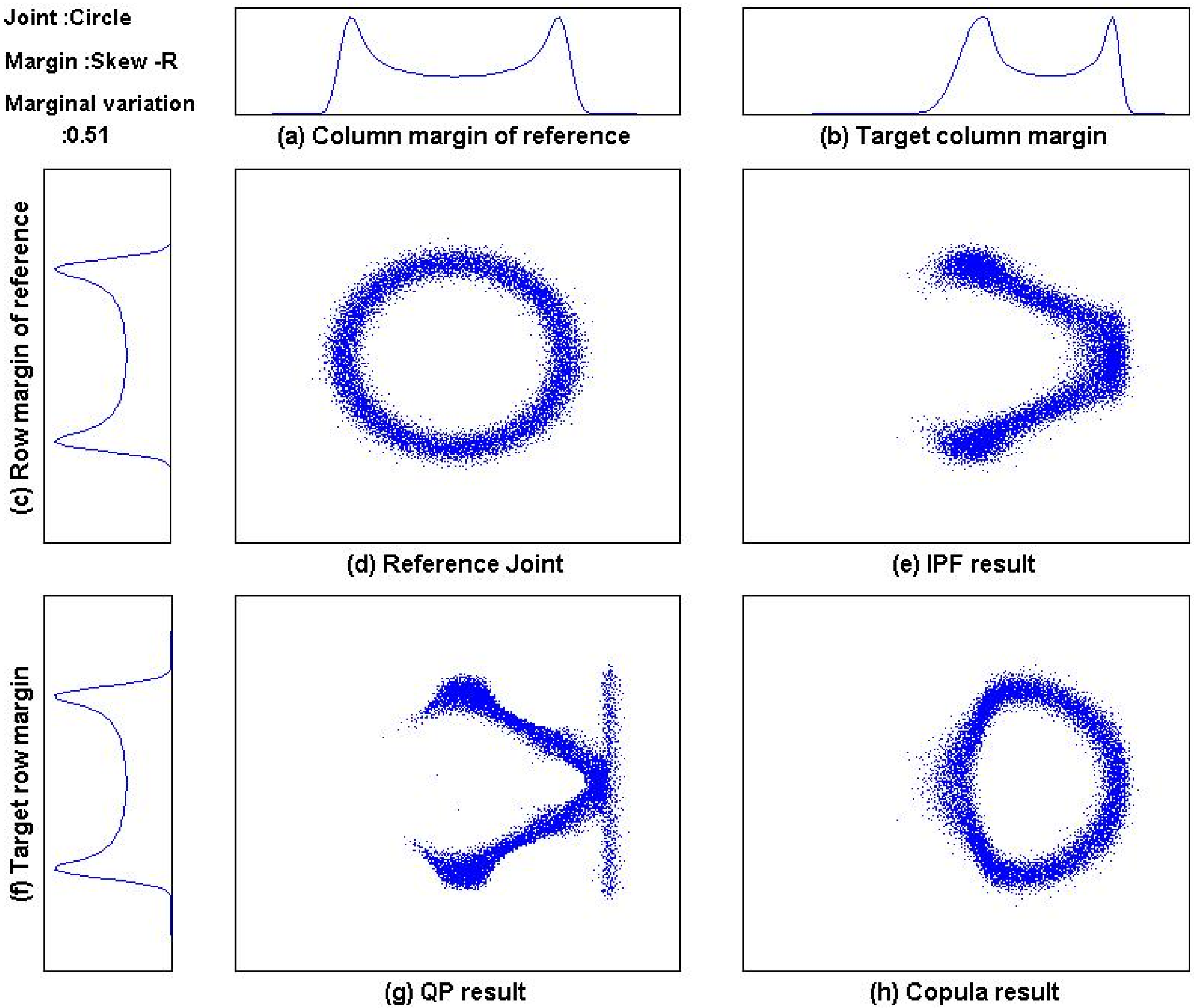

2-14-1. Reference joint distribution : Circle, Target marginal type : Fat tail, Marginal variation : 0.1

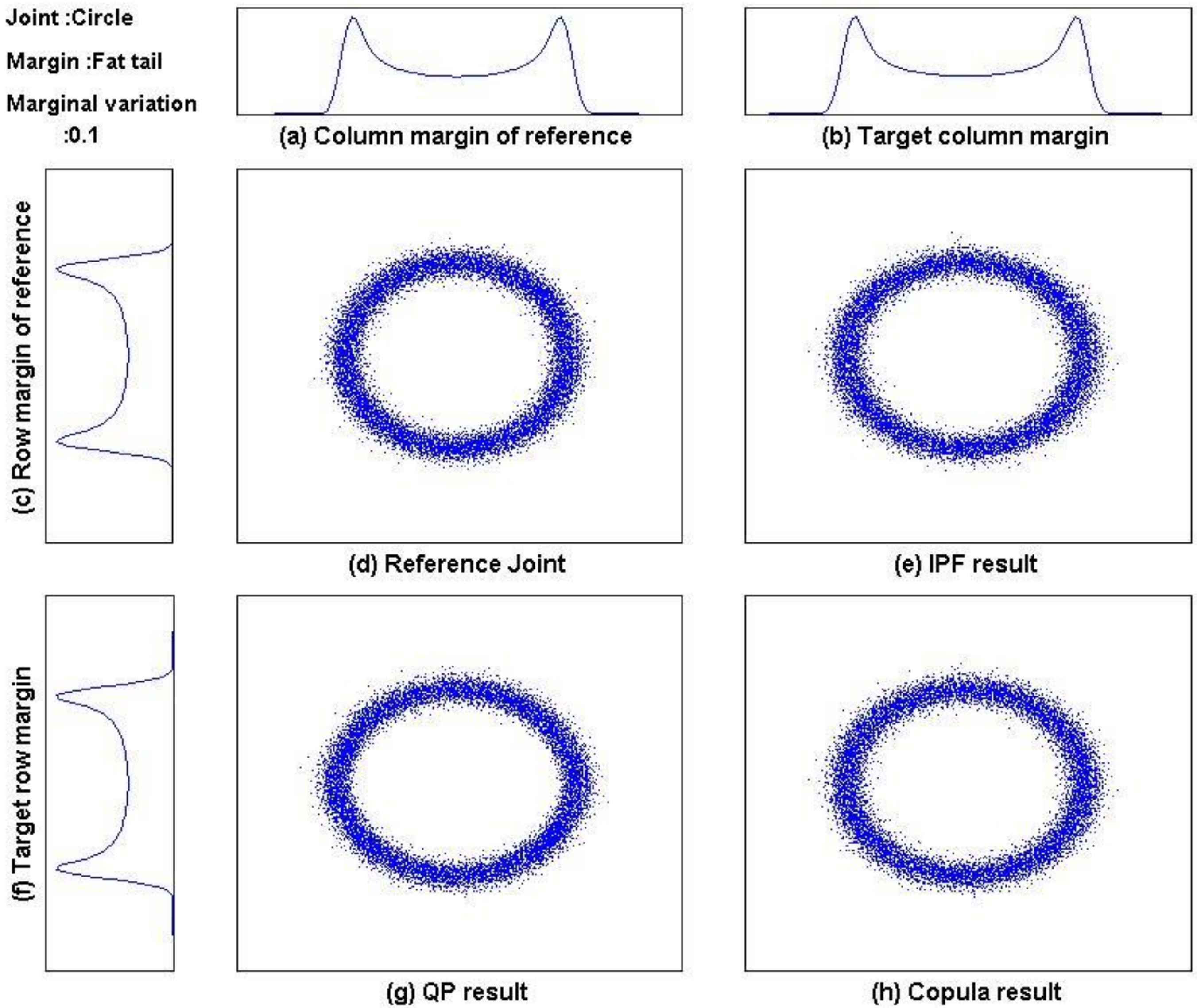

2-14-2. Reference joint distribution : Circle, Target marginal type : Fat tail, Marginal variation : 0.2

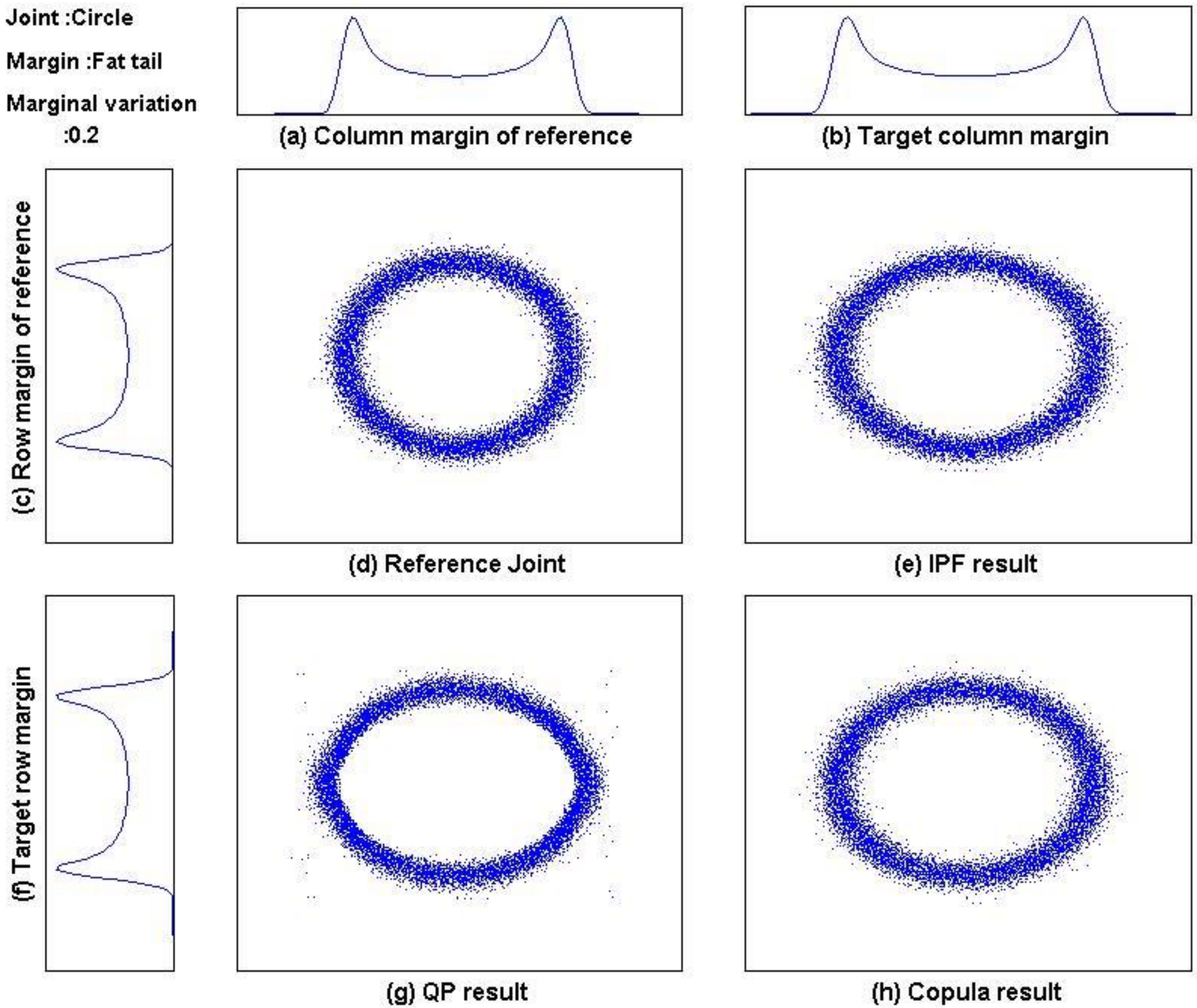

2-14-3. Reference joint distribution : Circle, Target marginal type : Fat tail, Marginal variation : 0.3

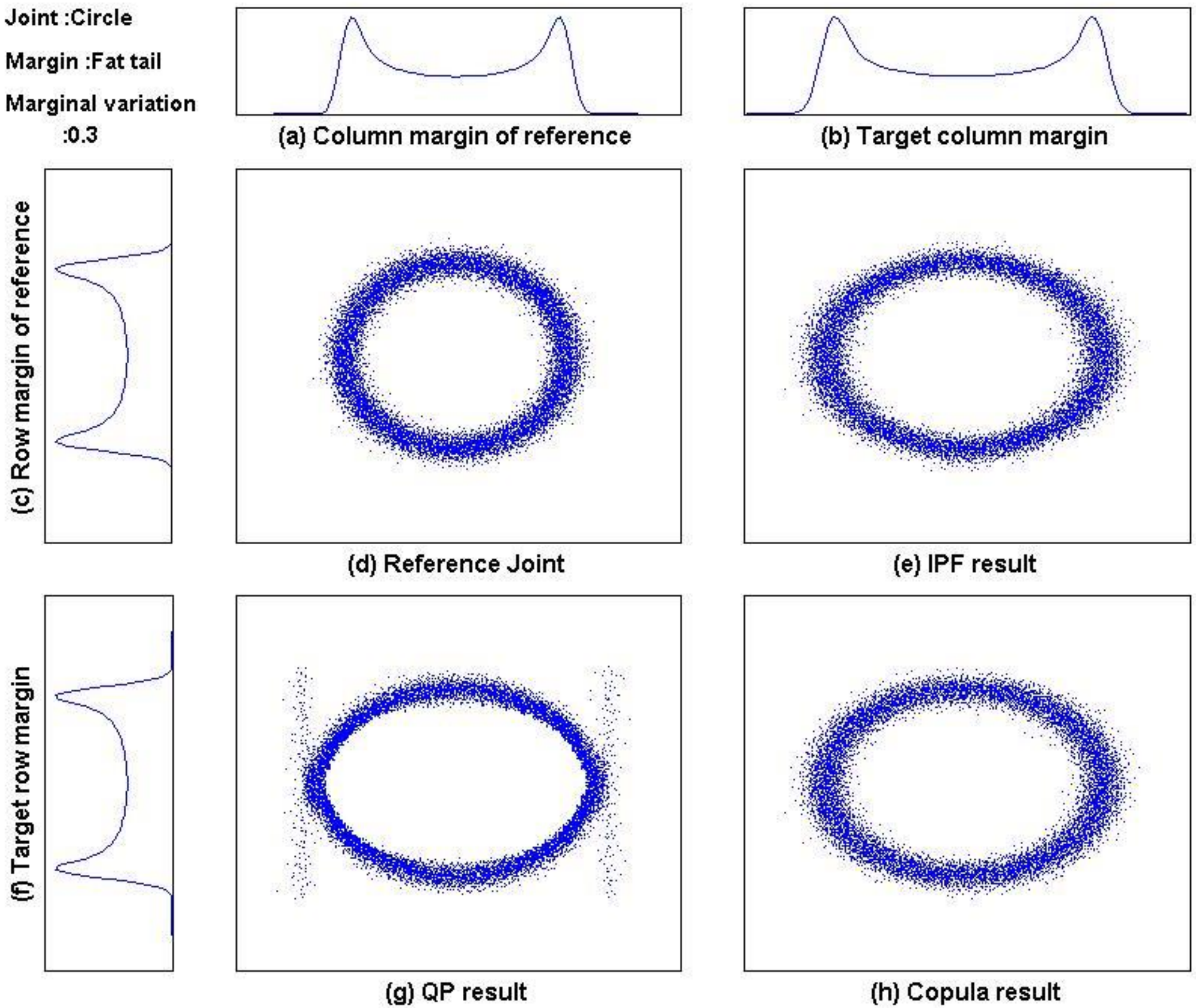

2-14-4. Reference joint distribution : Circle, Target marginal type : Fat tail, Marginal variation : 0.4

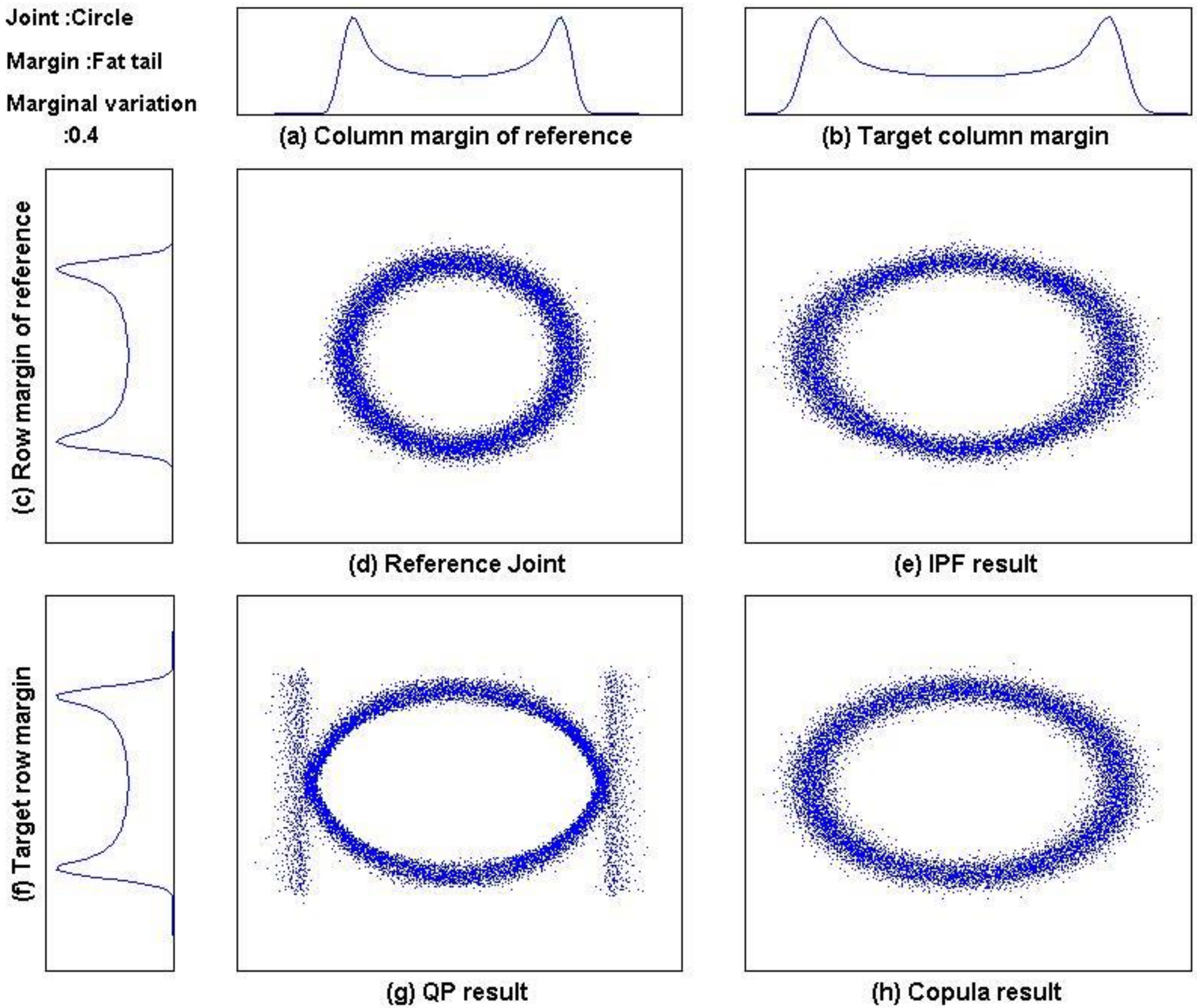

2-14-5. Reference joint distribution : Circle, Target marginal type : Fat tail, Marginal variation : 0.5

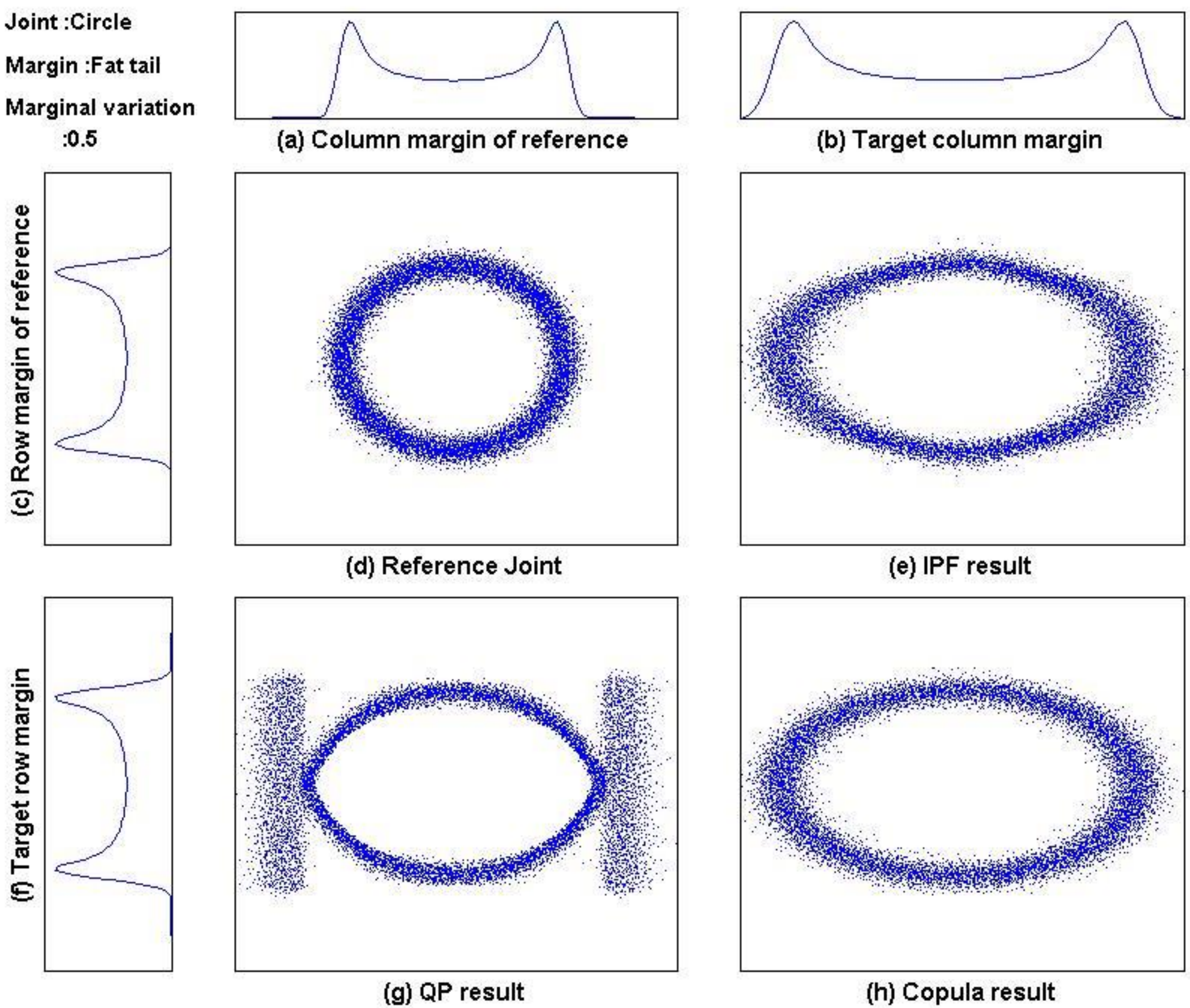

2-15-1. Reference joint distribution : Circle, Target marginal type : Thin tail, Marginal variation : 0.2

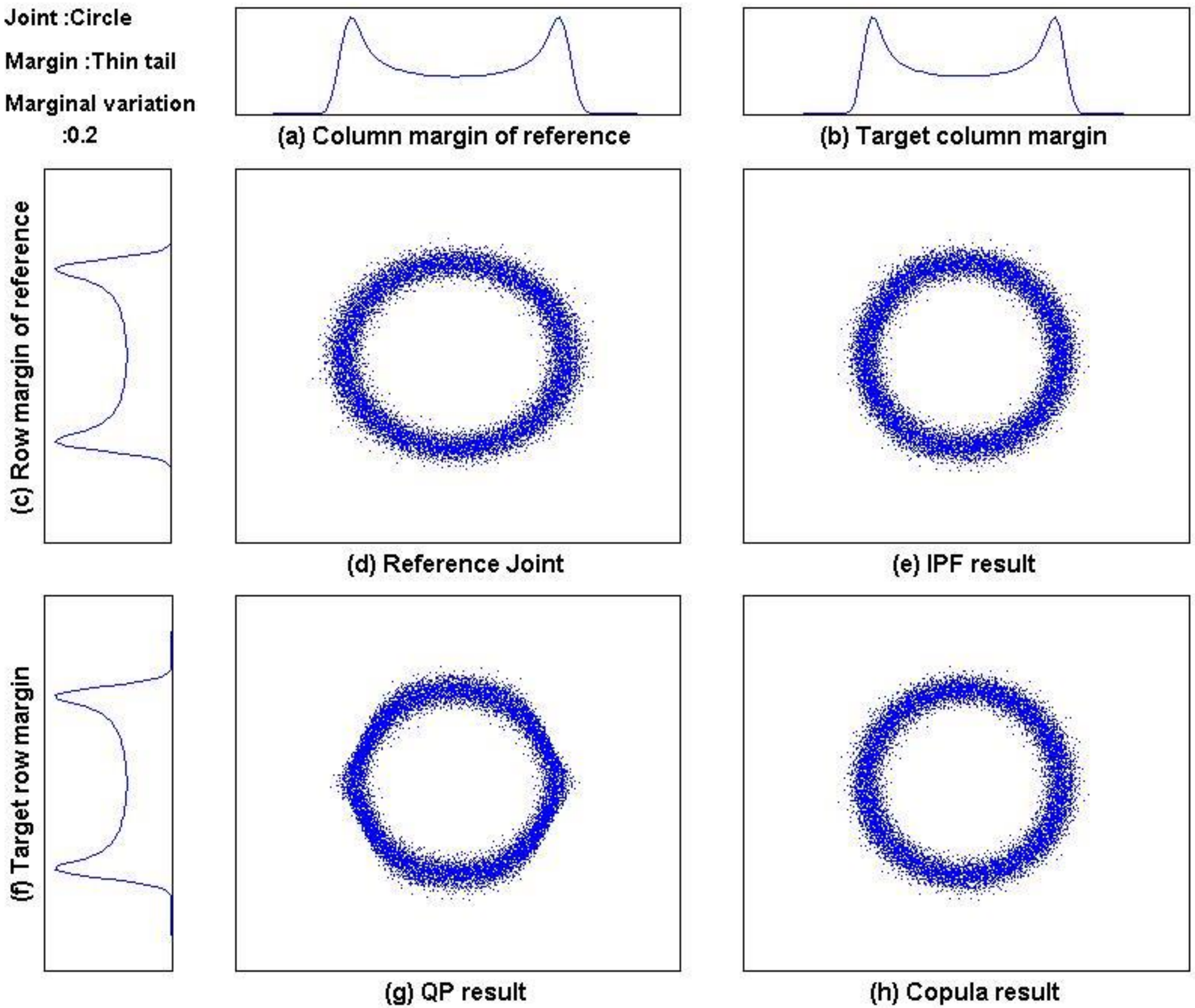

2-15-2. Reference joint distribution : Circle, Target marginal type : Thin tail, Marginal variation : 0.4

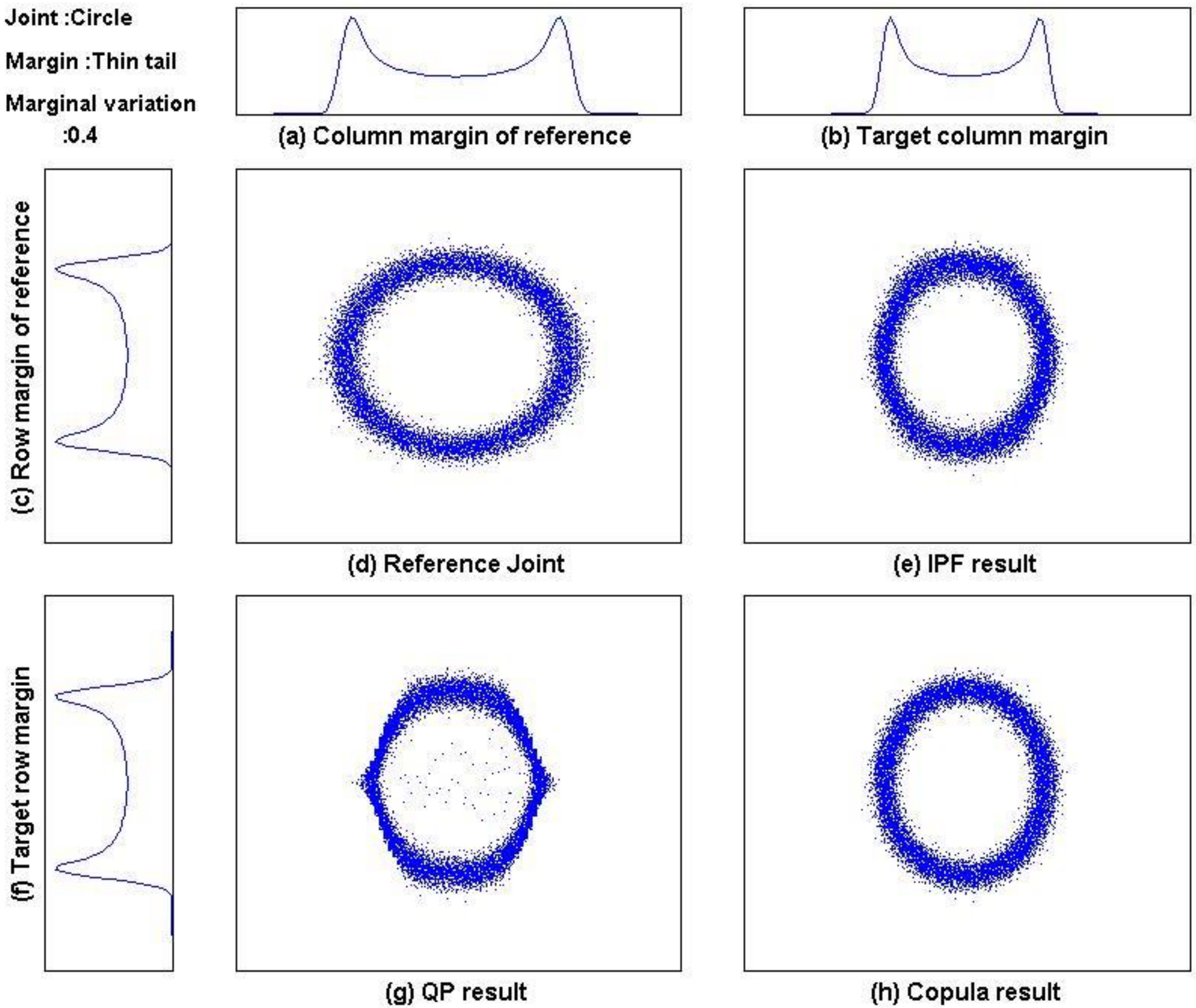

2-15-3. Reference joint distribution : Circle, Target marginal type : Thin tail, Marginal variation : 0.6

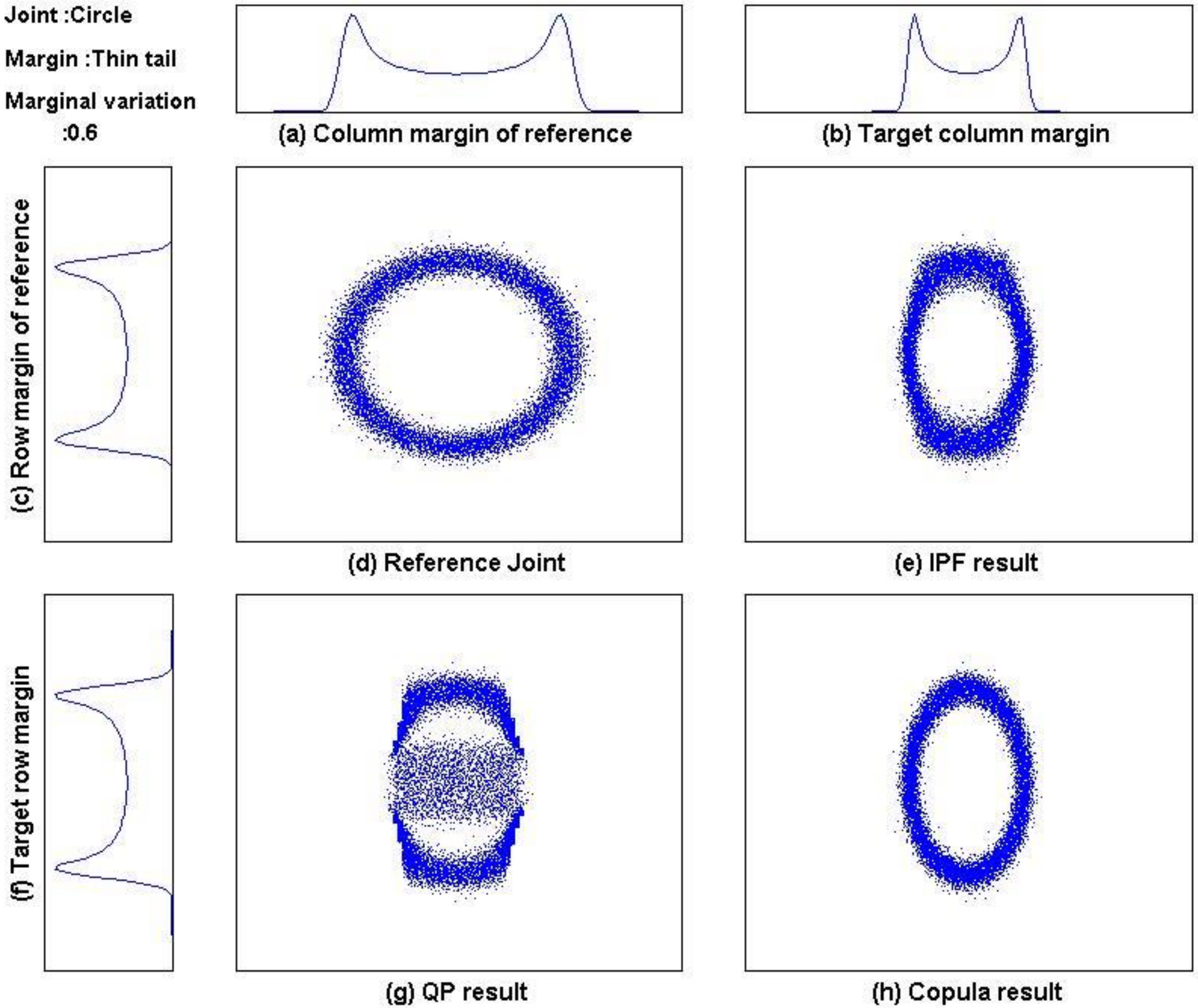

2-15-4. Reference joint distribution : Circle, Target marginal type : Thin tail, Marginal variation : 0.8

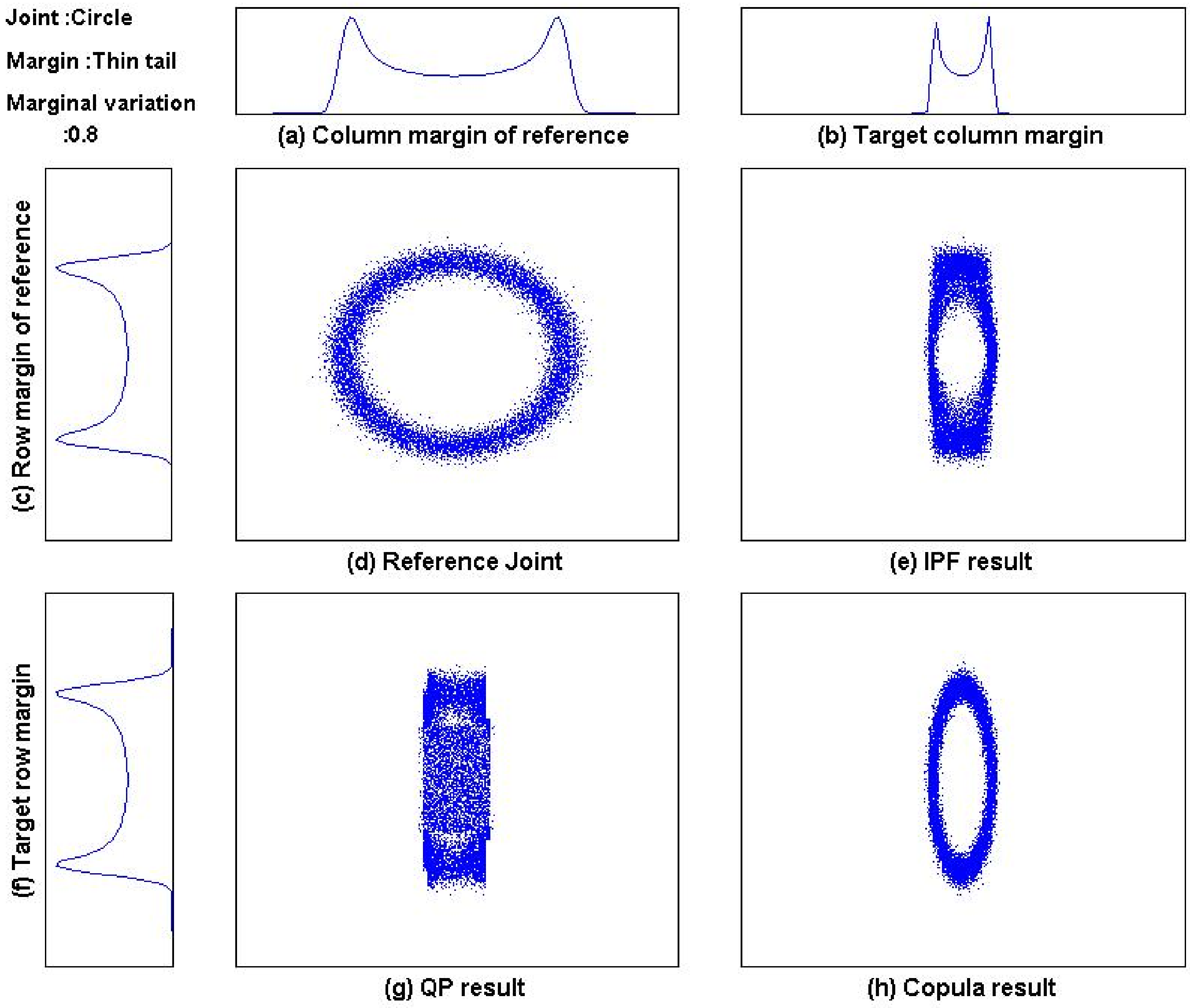

Supplement: S2 Fig — (PDF) [file pone.0159496.s002.pdf]
