# Supplementary material for: Copula-Based Approach to Synthetic Population Generation
Source: PLoS One. 2016 Aug 4;11(8):e0159496. doi: 10.1371/journal.pone.0159496 (PMC4973930; doi:10.1371/journal.pone.0159496)
Supplement: S1 Text — (PDF) [file pone.0159496.s003.pdf]

S1 Text. Proofs for Lemma 1 & Lemma 2

$s(u) = \dfrac{u - u_i}{u_{i+1} - u_i}$ and $t(v) = \dfrac{v - v_j}{v_{j+1} - v_j}$

$\mathrm{C}(u, v) = (1 - s(u))(1 - t(v))A_{i,j} + (1 - s(u))\, t(v)A_{i,j+1} + s(u)(1 - t(v))A_{i+1,j} + s(u)\, t(v)A_{i+1,j+1}$

Lemma 1. $\mathrm{C}(u, v)$ is at least C^0 continuous at all locations in $[0,1] \times [0,1]$.

<Proof>

$s(u) = \frac{u-u_i}{u_{i+1}-u_i}$ is a piecewise linear function of u, which is C^0 continuous. So is $t(v) = \frac{v-v_j}{v_{j+1}-v_j}$. Since $\mathrm{C}(u, v)$ is a bilinear interpolation of $A_{i,j}$'s, which is obviously C^0 continuous with respect to s and t. $\mathrm{C}(u, v)$ is a composite function of C^0 continuous functions, and hence we can conclude $\mathrm{C}(u, v)$ is C^0 continuous.

Lemma 2. $\mathrm{C}(u, v)$ satisfies the properties (a)~(c) of a copula function

<Proof>

(a) When $u = 0$, $i = 0$ and $u_i = u_0 = 0$. So, $s(0) = 0$.. When $u = 1$, $i = m - 1$ and $u_{i+1} = u_n = 1$. So, $s(1) = 1$. Similarly, $t(0) = 0$ and $t(1) = 1$.

$\mathrm{C}(u, 0) = (1 - s(u))A_{i,0} + s(u)A_{i+1,0} = 0$ because $A_{i,0} = 0$ by definition.

$\mathrm{C}(u, 1) = (1 - s(u))\, A_{i,n} + s(u)\, A_{i+1,n} = (1 - s(u))\, u_i + s(u)\, u_{i+1} = \frac{u_{i+1}-u}{u_{i+1}-u_i}\, u_i + \frac{u-u_i}{u_{i+1}-u_i}\, u_{i+1} = u.$

Similarly, we can verify that $\mathrm{C}(0, v) = 0$ and $\mathrm{C}(1, v) = v$.

(b) Firstly, consider when $u + du \le u_{i+1}$. $s(u + du) = s(u) + ds$ for some $ds > 0$.

$\mathrm{C}(u + du, v) = \mathrm{C}(u, v) - ds(1 - t(v))A_{i,j} - ds\, t(v)A_{i,j+1} + ds(1 - t(v))A_{i+1,j} + ds\, t(v)A_{i+1,j+1} = \mathrm{C}(u, v) + ds[(1 - t(v))(A_{i+1,j} - A_{i,j}) + t(v)(A_{i+1,j+1} - A_{i,j+1})]$.

Since $A_{i+1,j} \ge A_{i,j}$, $A_{i+1,j+1} \ge A_{i,j+1}$, $ds > 0$, and $0 \le t(v) \le 1$, we can say $\mathrm{C}(u + du, v) \ge \mathrm{C}(u, v)$.

If $u + du$ belongs to the next interval (i.e. $u_{i+1} < u + du \le u_{i+2}$), $\mathrm{C}(u + du, v) \ge \mathrm{C}(u_{i+1}, v) \ge \mathrm{C}(u_i, v)$.

In this manner, we can show that $\mathrm{C}(u + du, v) \ge \mathrm{C}(u, v)$ holds for any $du > 0$.

Similar argument can establish $\mathrm{C}(u, v + dv) \ge \mathrm{C}(u, v)$ for any $dv > 0$.

(c) Again consider when (u, v) and $(u + du, v + dv)$.belong to the same interval $[u_i, u_{i+1}] \times [v_j, v_{j+1}]$. Then, $s(u + du) = s(u) + ds$ and $t(v + dv) = t(v) + dt$ for some $ds > 0$ and $dt > 0$.

$\mathrm{C}(u + du, v + dv) - \mathrm{C}(u, v + dv) = ds[(1 - t(v) - dt)(A_{i+1,j} - A_{i,j}) + (t(v) + dt)(A_{i+1,j+1} - A_{i,j+1})]$, and

$\mathrm{C}(u + du, v) - \mathrm{C}(u, v) = ds[(1 - t(v))(A_{i+1,j} - A_{i,j}) + t(v)(A_{i+1,j+1} - A_{i,j+1})]$.

By subtracting the two, we get $\mathrm{C}(u + du, v + dv) - \mathrm{C}(u + du, v) - \mathrm{C}(u, v + dv) + \mathrm{C}(u, v) = ds \cdot dt \cdot (A_{i+1,j+1} - A_{i,j+1} - A_{i+1,j} + A_{i,j}) = ds \cdot dt \cdot a_{i+1,j+1} \ge 0$. When $(u + du, v + dv)$ belongs to a different interval, similar argument as in (b) can be used.
